# Comparative genomics of the mimicry switch in *Papilio dardanus*

**DOI:** 10.1098/rspb.2014.0465

**Published:** 2014-07-22

**Authors:** Martijn J. T. N. Timmermans, Simon W. Baxter, Rebecca Clark, David G. Heckel, Heiko Vogel, Steve Collins, Alexie Papanicolaou, Iva Fukova, Mathieu Joron, Martin J. Thompson, Chris D. Jiggins, Richard H. ffrench-Constant, Alfried P. Vogler

**Affiliations:** 1 Department of Life Science, Natural History Museum London, London SW7 5BD, UK; 2 Department of Life Sciences, Imperial College London, South Kensington Campus, London SW7 2AZ, UK; 3 Department of Zoology, University of Cambridge, Downing Street, Cambridge CB2 3EJ, UK; 4 Max Planck Institute for Chemical Ecology, Beutenberg Campus, Jena 07745, Germany; 5 African Butterfly Research Institute, 0800 Westlands, Nairobi 14308, Kenya; 6 School of Biosciences, University of Exeter, Cornwall Campus, Daphne du Maurier Building, Penryn TR10 9EZ, UK; 7 CSIRO Ecosystem Sciences, Black Mountain Laboratories, Canberra 2601, Australia; 8 Muséum National d'Histoire Naturelle, CNRS UMR 7205, CP50, 45 Rue Buffon, Paris 75005, France

**Keywords:** Batesian mimicry, polymorphism, Lepidoptera, supergene, genotype–phenotype associations

## Abstract

The African Mocker Swallowtail, *Papilio dardanus*, is a textbook example in evolutionary genetics. Classical breeding experiments have shown that wing pattern variation in this polymorphic Batesian mimic is determined by the polyallelic *H* locus that controls a set of distinct mimetic phenotypes. Using bacterial artificial chromosome (BAC) sequencing, recombination analyses and comparative genomics, we show that *H* co-segregates with an interval of less than 500 kb that is collinear with two other Lepidoptera genomes and contains 24 genes, including the transcription factor genes *engrailed* (*en*) and *invected* (*inv*). *H* is located in a region of conserved gene order, which argues against any role for genomic translocations in the evolution of a hypothesized multi-gene mimicry locus. Natural populations of *P. dardanus* show significant associations of specific morphs with single nucleotide polymorphisms (SNPs), centred on *en*. In addition, SNP variation in the *H* region reveals evidence of non-neutral molecular evolution in the *en* gene alone. We find evidence for a duplication potentially driving physical constraints on recombination in the *lamborni* morph. Absence of perfect linkage disequilibrium between different genes in the other morphs suggests that *H* is limited to nucleotide positions in the regulatory and coding regions of *en*. Our results therefore support the hypothesis that a single gene underlies wing pattern variation in *P. dardanus*.

## 1. Introduction

Batesian mimics are palatable species that avoid predation by evolving resemblance to toxic or harmful models [1]. They constitute excellent examples of adaptation by natural selection, in which unrelated species attain phenotypic similarity in response to selection by visual predators [2]. However, as Batesian mimics increase in frequency in the local prey community, predators may begin to associate the phenotype with palatability and the benefit of mimicry becomes reduced [3]. This leads to negative frequency-dependent selection on mimetic phenotypes, which may favour the evolution of multiple morphs in a population that mimic different models [4]. The polymorphism is maintained by balancing selection, which prevents any single form from reaching sufficient abundance to lose its protective benefit.

**Figure 1.**
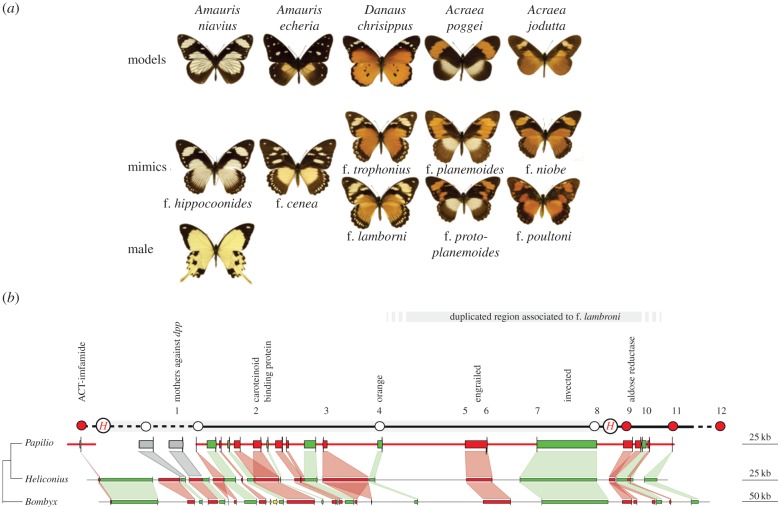
(*a*) Examples of phenotypes displayed by *P. dardanus* and their presumed models. The arrangement from left to right represents the female dominance hierarchy starting with the bottom recessive f. *hippocoonides* to the top-dominant f. *poultoni*. (*b*) A genomic map of the *H* region. Inferred gene products, including possible candidate genes, are mapped relative to the *ACT* flanking marker [17] (see the electronic supplementary material for additional information). White and red circles denote sequence markers used to test for recombination analyses in a f. *cenea*—f. *hippocoonides* laboratory cross [17] indicating co-segregation (white) and recombination (red) with the *H* phenotype. Bottom: homologous regions of *P. dardanus*, *H. melpomene* (www.butterflygenome.org) and *B. mori* (http://sgp.dna.affrc.go.jp/KAIKO/). Predicted protein-coding genes are shown by thick red and green lines, and their directions of transcription are indicated by thin vertical lines indicating the 3′-end of the coding region. The scale bar at the right shows physical distances in kilobase. Conserved gene orders in the three species are indicated by alternating red and green shading. Grey shading links several loci that are absent from the BAC tile path, but whose presence was confirmed by next generation sequencing (NGS). Numbers 1–12 refer to loci used in the analysis of SNP associations.

Among polymorphic Batesian mimics, the African Mocker Swallowtail, *Papilio dardanus* Yeats in Brown, 1776, has been a prominent study system ever since Trimen [5] recognized the diverse colour morphs to be members of a single species. Mimicry is limited to the females, which differ greatly from the non-mimetic males at most African mainland localities (figure 1) [6]. Laboratory crosses showed that most of the phenotypic variation is determined by a single Mendelian locus, termed *H*, whose various alleles exhibit a dominance hierarchy such that most of them are inherited without producing intermediate phenotypes [7–10].

Previous work has proposed *H* to be a large effect ‘supergene’ locus, consisting of a block of tightly linked, functional sites that each influenced some aspect of the wing morphology in the various mimicry forms. It has been suggested that in the formation of *H*, strong selection for linkage resulted in inter-chromosomal translocations that brought together unlinked wing patterning loci [11–13], although theoretical work suggests that such translocations are unlikely [14]. More likely, *H* arose due to ‘sieving’ of mutations whereby only those mutations linked to a gene already conferring resemblance to a specific model are used to specify the mimetic phenotypes [15], resulting in a supergene that gradually acquires allelic diversity at multiple linked sites in the process of increasing the resemblance with the mimicry models.

Despite the important role of *P. dardanus* in the development of evolutionary theory, the *H* locus has still not been characterized at the DNA level. Genomic approaches to the study of natural variation provide new means for the molecular and evolutionary characterization of the *H* locus. A recent study of the related polymorphic species *Papilio polytes* revealed that a single locus, *doublesex* (*dsx*), determines the different wing patterns, apparently through a combination of regulatory mutations and amino acid substitutions [16]. In *P. dardanus*, physical mapping identified a 13.9 cM region containing *H* and found the transcription factor gene *invected* (*inv*) to be closely linked to *H* [17]. The *inv* locus in arthropods is situated immediately adjacent to its paralogue *engrailed* (*en*) [18], which is functionally similar [19], and both genes have been associated with scale development and wing colour patterning in Lepidoptera [20,21]. Here, we use comparative genomics of wild-caught populations to characterize the *H* region covering *en/inv* and neighbouring genes. Molecular cloning and single nucleotide polymorphism (SNP) analysis of natural sequence variation delimit *H* to a narrow portion of the approximately 500 kb region analysed and support the hypothesis that mutations in a single regulatory gene could underlie the unique pattern variation of *P. dardanus*.

## 2. Material and methods

### (a) Bacterial artificial chromosome (BAC) library construction and screening

A BAC library was constructed using partially *Hin*dIII-digested genomic DNA pooled from several specimens of the Kenyan subspecies *P. dardanus tibullus* and screened with *inv* and *en* [22] probes. Eleven clones were identified, of which four were sequenced using Sanger technology. Gene predictions were made using KAIKOGAAS (http://kaikogaas.dna.affrc.go.jp) using *Bombyx mori* as reference genome.

### (b) Population samples and single nucleotide polymorphism analyses

For recombination analysis, the segregation of SNPs was assessed in a previously published pedigree brood (Brood 59 of [17]). For population genetic analyses, specimens were collected in 2002/2003 at Mt. Kenya. Additional specimens were obtained commercially, or caught in Kenya in 1998, 2007/2008 and 2010 (table 1; voucher numbers are given in the electronic supplementary material). DNA was extracted from small tissue sections with the DNeasy blood and tissue kit (Qiagen). SNP variation in 16 gene fragments was assessed using Sanger sequencing (table 2). PCR primers are given in the electronic supplementary material. All sequence traces were edited using Sequencher 4.6 (Gene Codes Corporation). SNPs and their allele frequencies were counted using the SNPatron Perl script [23]. Genotype–phenotype associations were investigated with the R package SNPassoc [24], using the genetic model that assesses the association of each allele with a given variable site by testing the homozygous and heterozygous state of the major allele versus the homozygous alternative state against the phenotype. The expectation of a SNP associated with a phenotypically dominant morph is that it only occurs in the dominant phenotype, most likely in a heterozygous state, and is absent from all phenotypes recessive to this phenotype. Only SNPs for which the major allele occurred at a relative frequency of less than 0.97 were analysed, with each response variable (phenotype) tested against those lower in the dominance hierarchy combined. The test is based on a likelihood ratio test against permutated data. Bonferroni corrections for multiple tests were applied. Linkage disequilibrium (LD) was calculated using the composite likelihood method first described by Weir [25]. This method can be applied on sequence data of unknown phase, as generated here. Calculations were performed with the package RxC [26]. Significance of the allelic correlations was obtained using permutation tests, and Bonferroni corrections for multiple tests were applied. A custom Perl script was used to generate a LD heat map.

**Table 1.** Number of specimens used in this study, their phenotype, subspecies and year of sampling. *Papilio dardanus dardanus* from Kakamega, *P. dardanus polytrophus* from Mt. Kenya, *P. dardanus tibullus* from Watamu, Shimoni, Nguruweni or Taita Hills. Full details and specimens voucher numbers are given in the electronic supplementary material.

subspecies (year)	*hippocoonides*	*cenea*	*lamborni*	(proto) *planemoides*	*poultoni*	total
polytrophus (2002–2003)	16	15	16	1	17	65
*polytrophus* (2010)	2	6	2	1	3	14
*dardanus* (1998)				3		3
*tibullus* (2007–2008)	10		2		3	1
commercial (2008)			1			1
total	28	21	20	5	23	97

**Table 2.** Gene fragments used for SNP analysis and tests of molecular evolution. No., number on physical map (figure 1); length, number of base pairs of PCR fragment; position, position of first nucleotide in fragment on the BAC tile path; missing, number of samples not sequenced, out of 97 in total; SNPs < 0.97, number of SNPs with major-allele frequency smaller than 97%. The asterisk refers to a physical position of approximately 3 kb outside of the BAC tile path determined by LR-PCR. Bold letters indicate genes that are not excluded from *H* by recombination analyses. McDonald–Kreitman (MK) and Hudson, Kreitman and Aguade [34] (HKA), *p*-values of the MK and HKA tests with *P. glaucus* (left) and *P. polytes* (right) of slash. For the HKA test, the unlinked loci were used for intraspecific comparisons and all *P. dardanus* f. *lamborni* were excluded. Jukes–Cantor correction was applied to obtain number of fixed differences between species. NA, not available. NP, not performed.

	no.	gene	length	position	missing	SNPs < 0.97	MK (*p*-values)	HKA (*p*-values)
*H* linked	1	**MAD**	145	—	33	**0**	NP/NP	NP/NP
	2	**CBP**	162	46 589	8	**9**	0.43/1.00	0.66/0.80
	3	**SCF**	821	94 342	1	**21**	0.13/0.49	**0.04**/0.11
	4	**orange**	129	133 078	7	**9**	0.37/0.37	0.46/0.88
	5	**engrailed (exon 1)**	513	192 833	4	**42**	**3 × 10^−6^**/**2 × 10^−7^**	0.34/0.17
	6	**engrailed (exon 3)**	177	208 074	4	**17**	0.35/0.37	0.44/0.80
	7	**invected (exon 5)**	191	243 505	2	**20**	0.16/0.07	**0.02**/0.57
	8	**invected (exon 1)**	335	286 121	1	**20**	0.52/1.00	0.66/0.85
	9	*AR*	192	311 476	0	**18**	0.10/1.00	0.68/0.67
	10	*CbpA*	140	319 618	3	**10**	NA/0.79	NA/0.88
	11	*CTD*	119	*	1	**6**	0.36/0.27	0.37/0.48
	12	hypothetical protein	202	—	1	**13**	0.70/0.33	0.75/0.89
unlinked	13	*dpp*	189	—	3	**3**	1.00/1.00	—
	14	*RpS19*	151	—	2	**6**	0.26/1.00	—
	15	*cdp*	214	—	2	**15**	0.46/1.00	—
	16	*wg*	314	—	4	**11**	0.12/**0.02**	—

### (c) Roche 454 and Illumina Solexa sequencing

Transcriptome data were obtained from RNA of wing discs that were dissected from seven individuals of each sex in the last larval instar or in a pre-pupal stage. Reverse transcription was performed using the PrimeScript reverse transcription enzyme (Takara, Otsu, Japan). Double-stranded cDNA was normalized using the Kamchatka crab duplex-specific nuclease method (Trimmer cDNA normalization kit, Evrogen, Moskow, Russia) and shotgun sequenced with a 454 GS-FLX Titanium pyrosequencer (Roche Applied Science).

Long-range PCR was conducted using Takara LA Taq on a single f. *lamborni* specimen, and amplicons were shotgun sequenced with 454 pyrosequencing. Raw data were preprocessed using Prinseq-lite [27]. Retained reads were mapped onto the BAC tile path (RepeatMasker masked [28]) using Burrows–Wheeler aligner (BWA) [29]. ShoRAH [30] (sliding window: size 150 bp, shift 75 bp) was used to obtain phased haplotypes.

Whole-genome shotgun sequencing was performed on a single male individual from subspecies *P. dardanus tibullus*, homozygous for the bottom recessive *hippocoon* phenotype. A 300 bp inset library was prepared from 3 µg of RNAse A-treated genomic DNA using Illumina TruSeq DNA Sample Prep Kit and SAGE Blue Pippin size selection system. The library was sequenced in a 1/3th of a HiSeq 2000 lane using 100 base paired-end reads (v3 chemistry). Raw reads were processed using RTA 1.17.21.3 and Casava v. 1.8.3. Reads were further processed using Prinseq-lite [27] and assembled using SOAPdenovo2 [31] and Abyss [32] using various K-mer sizes.

### (d) Molecular evolution

The McDonald–Kreitman [33] and HKA tests [34] were applied to test for non-neutral evolution and balancing selection. These tests compare the within-species variation to the between-species divergence using a close relative. Sequence data from Short Read Archives SRR850327 and SRR850325 for *P. polytes* and *Papilio glaucus* [35] were used as outgroups. Because these species were only distantly related to *P. dardanus*, tests of molecular rates may be affected by multiple hits at variable sites, which was corrected by applying a Jukes–Cantor model of sequence variation [36]. For *P. dardanus*, haplotypes were inferred using Phase [37] as implemented in DnaSP [38]. Diversity and divergence values were obtained using DnaSP, and McDonald–Kreitman tests were performed using Fisher's exact tests. Multilocus HKA tests that assess the greater than expected diversity among alleles compared with a set of reference loci were performed on synonymous sites only, using the HKA software package (Hey Lab). All individuals carrying the duplicated *en* allele associated to f. *lamborni* (see below) were removed.

## 3. Results

### (a) BAC sequencing and positional cloning of *H*

A genomic region in the vicinity of the *H* locus was analysed by sequencing BAC clones for a contiguous tile path of approximately 340 kb that includes the complete *en* and *inv* candidate genes [17]. The tile path contains 24 putative protein-coding regions, based on sequence homology with known proteins and annotations of two published lepidopteran genomes, the postman butterfly, *Heliconius melpomene*, and the silk moth *B. mori* (figure 1). The extent of the *en*/*inv* region is more than 90 kb, including long introns of up to approximately 40 kb in *inv*. The cloned region is rich in genes implicated in colour and pattern formation in insects and includes the genes for a putative Sanpodo homologue, orange, a carotenoid-binding protein (*CBP*) and two aldose reductase (*AR*) genes (figure 1). The Sanpodo protein regulates *notch* [39], which is involved in wing scale specification in butterflies [40]. The *orange* gene product is involved in protein transport and the tryptophan ommochrome biosynthesis pathway needed for the production of polycyclic orange and red pigments (although ommochromes have not been described from *Papilio* wings). A *CBP* in *B. mori* has been shown to determine cocoon colour [41]. The aldose reductases show significant similarity to 3-dehydroecdysone-3(β)-reductases involved in ecdysone biosynthesis [42], and temporal variation in the expression of this hormone is of key importance in lepidopteran wing patterning [43]. Furthermore, 3-dehydroecdysone-3(β)-reductase has been shown to be involved in cryptic pattern formation in larval stages of papilionids [44]. We found all of these genes, except for *CBP*, to be present in a transcriptome library prepared from normalized cDNA of last larval instar and pre-pupal wing discs, in addition to *inv* and *en* transcripts.

In order to test the hypothesis that the evolution of this region involved translocations of unlinked elements, we compared the *P. dardanus* gene order with *B. mori* and *H. melpomene*. The extent of this fragment is approximately twice the size in *B. mori* compared with that in the other two species. However, the gene order in *P. dardanus* was largely collinear with the corresponding genome regions in both species (figure 1), arguing against large-scale inter-chromosomal translocations in the *H* region, as has been proposed under the supergene hypothesis [11,13].

A mapping family (Brood 59 of [17]) was used to further delimit the extent of the *H* locus (figure 1). Earlier crosses had shown that *H* co-segregates with the first exon of *inv*, but fine-scale mapping was not possible within an interval defined by two amplified fragment length polymorphism (AFLP) markers (*ACT* and *Pd*) on either side of *inv*, thought to be up to 3 MB in size [17]. We studied segregation patterns of seven new markers in this interval in the existing pedigree specimens (figure 1) [17]. Two loci were located between ACT and *inv* (primer pairs Pd13–Pd16, Pd88–Pd89) and three loci between *inv* and Pd (primer pairs Pd15-1D8_F0_F, Pd52–Pd54, Pd32–Pd33). In addition, two markers outside the tile path were developed with reference to the *B. mori* genome; they are located on either side of the BAC tile path near ACT (Pd121–Pd122) and Pd (Pd227–228). Variation in these markers was analysed using DNA (Sanger) sequencing, restriction digests or size variation (in cases of unambiguous length differences of PCR product). Scoring these markers for parents and offspring localized a crossing-over event in two individuals between *inv* and a locus approximately 13 kb upstream of the 5′ end of the *inv*-coding regions (see white and red circles in figure 1), excluding five candidate genes from *H* (table 2). In the other direction, all loci except the distant *ACT* co-segregated with the *H* phenotype and hence no further reduction of the interval was possible. Based on sequence similarity with the two known genomes, it was possible to obtain and order all genes within the interval. High similarity was revealed between the sequence of *ACT* and the *B. mori* gene coding for the neuropeptide IMFamide (loci KAIKOGA050177, KAIKOGA050178) and a predicted *H. melpomene* gene [44] on contig HE670890 [78861..79874], located approximately 180 kb and approximately 90 kb from the tile path region, respectively. In both reference genomes, this portion contains five protein-coding genes. Illumina shotgun sequence data for a single specimen (approx. 50× coverage) was assembled to search for the corresponding genome region in *P. dardanus.* Contigs showing significant similarity were put in order to complete the genomic map beyond the BAC tile path (electronic supplementary material). Inter-genic gaps were closed using standard PCR and Sanger sequencing, with only a single intra-genic sequence gap remaining in the Myosin-Va gene (*B. mori* Gene001003). The resulting map revealed full gene synteny between the three species, and the region that defines the *H* interval therefore contains 24 genes in total.

### (b) Genotype–phenotype associations

The extent of the mimicry locus was further investigated by testing the associations of SNPs with particular wing pattern morphs. We used five female forms (ff.) from a single population of *P. d. polytrophus* (in order of increasing dominance: ff. *hippocoonides*, *cenea*, *lamborni*, *planemoides*, *poultoni*), which were supplemented with specimens from elsewhere in East Africa (table 1). SNP variation was assessed in the exons of 12 loci across the *H* region (10 of which were located on the BAC tile path) and four unlinked loci, including *wingless* (*wg*), *Ribosomal Protein S19* (*RpS19*), *decapentaplegic* (*dpp*) and *cell division protein* (*cdp*), for 97 individuals. Within 3484 bps of sequence across these loci combined, we identified 220 SNPs with a major-allele frequency of less than 0.97.

**Figure 2.**
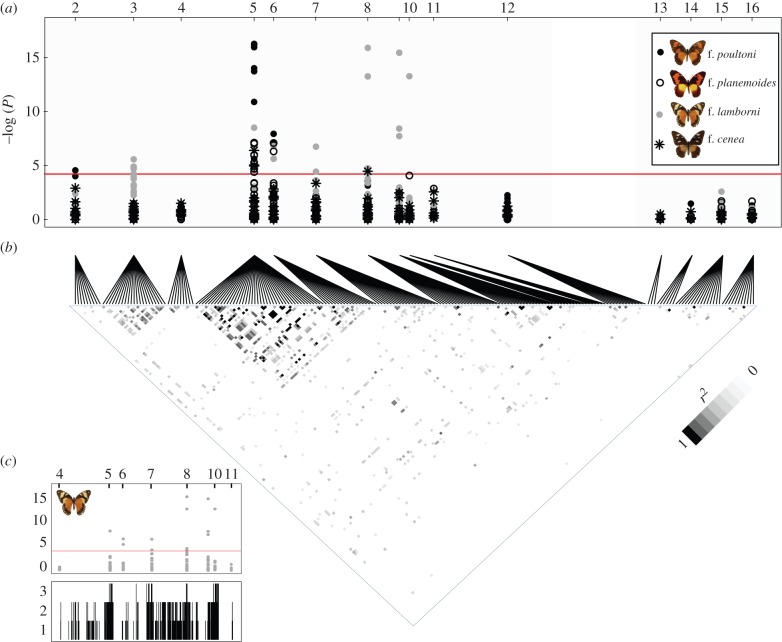
(*a*) Genetic associations of SNPs with wing pattern morphs. The significance of association for each SNP with a given colour morph was assessed separately for each morph against all morphs with a lower position in the dominance hierarchy. The horizontal axis represents 11 loci of the *H* region (2–12) and four unlinked genes (13–16). Locus 1 (MAD) did not contain polymorphic sites. The red horizontal line represents the significance threshold for association after Bonferroni correction for multiple testing. The grey symbols correspond to f. *lamborni* exhibiting the genomic duplication that is in perfect association with the phenotype. The SNP association therefore extends over the full length of the duplicated region. The extent of the duplication is evident from the presence of three alleles at certain nucleotide positions (see (*c*), bottom panel). Note that the full SNP association outside of the *en* locus is exclusively correlated to f. *lamborni* and likely correlated with the duplicated copy. (*b*) Heat plot showing LD (*r*^2^) of SNPs within and between loci. The 11 loci linked to the BAC tile path are given on the left, the four unlinked loci on the right. Only comparisons significant after Bonferroni correction are visualized in by grey-scale. In general, LD was low, with the exception of intra-locus comparisons within the Solute Carrier Family member and *en*, and inter-locus comparisons involving the two different exons of *en*. (*c*) Number of alleles observed in f. *lamborni*. Top panel: SNP association within the targeted region with f. *lamborni* as in panel (*a*). Bottom panel: the *y*-axis gives the total number of alleles observed in a 150-bp sliding window as inferred from 454 sequence data of LR-PCR products.

A likelihood ratio test of association of each SNP with particular morphs, taking the dominance hierarchy into account, shows significant association in all comparisons made. The strongest levels of association were confined to the *en*/*inv* region and the immediately adjacent *AR* and the Chitin-binding Peritrophin-A domain (*CbpA*) genes (figure 2)*.* This includes (i) full association of f. (*proto*)*planemoides* (five individuals) with SNPs in *en*; in addition the same SNPs were present in a single individual of the top-dominant f. *poultoni*, consistent with the presence of a recessive (*proto*)*planemoides* allele; (ii) near-complete association of f. *poultoni* with a cluster of SNPs, again centred on the first exon of *en*; (iii) significant association of the *cenea* phenotype to SNPs in the first exon of *en*, although none of these was fixed; (iv) full association of f. *lamborni* (15 individuals) with unique SNPs in *inv*, *AR* and *CbpA*. In addition, f. *lamborni* was in complete association with an 8-bp deletion in the first exon of *en*. This deletion was also present in two of the dominant f. *poultoni* individuals, as were the other fully associated SNPs, consistent with the presence of a recessive *lamborni* allele.

The deletion causing a frameshift in the highly conserved *en* gene is surprising. Using a PCR primer that binds to the deleted nucleotides and therefore does not amplify the truncated allele, we obtained two intact alleles that differed from the frameshifted copy, indicating a duplication of the region. SNPs in both copies showed association with the *lamborni* morph, but in the intact *en* fragments the association was not complete. The extent of this duplication was indicated by Roche/454 sequencing of long-range PCR fragments in fragments of 3–10 kb (from a approx. 200 kb sub-region in a single f. *lamborni* individual). When scored for the number of alleles detectable in windows of 150 bps, these sequences indicated that three alleles were present across the entire *en*/*inv* and adjacent *AR* genes, *CbpA* gene and Ubiquitin superfamily gene, but did not extend to *orange* on one side or *CTD* on the other (figure 2). Although we currently do not know its exact extent, the duplication does explain the SNP associations in the truncated *en*, *inv*, *AR* and *CbpA* genes with the *lamborni* phenotype (figures 1 and 2).

### (c) Linkage disequilibrium and patterns of variation

Reduced recombination and natural selection both disturb the random association of SNP alleles, resulting in more LD among loci than normally expected [45]. To verify whether population genetic processes or structural variation have affected SNP distributions within the interval, we assessed LD among SNPs using allelic correlation among loci [25,26]. Overall LD was low, specifically for inter-genic SNP comparisons (figure 2), with a possible exception involving the two exons of *en* that are separated by 16 kb.

To test whether any of the 16 loci investigated in the *H* region experienced balancing or positive selection, as predicted if a locus expresses divergent phenotypes, a McDonald–Kreitman test for the accumulation of non-synonymous (presumed non-neutral) changes at sites was applied. When performed against a baseline of between-species comparisons in the congeners *P. polytes* and *P. glaucus* [35], the McDonald–Kreitman tests were significant for *en* (electronic supplementary material), but not for any of the other 15 genes tested (table 2), which indicates that mutations within *en* alone deviate from the presumed neutral divergence. Long-term balancing selection is expected to result in a local peak of silent diversity in the neighbourhood of the target of selection [46,47]. This was tested using multilocus Hudson, Kreitman and Aguade (HKA) tests [34], but the result was not significant after Bonferroni correction for all genes, including *en* (table 2).

## 4. Discussion

The evolution of divergent mimetic morphs in *P. dardanus* and other *Papilio* has played an important role in the study of complex adaptive traits [48] and has fuelled arguments over Darwinian gradual change [49,50] versus macromutation [51,52]. We provide evidence for the localization of *H* in an interval that previously had been defined only by two AFLP markers of unknown physical distance. Here, this interval was narrowed down and sequenced using a combination of BAC sequencing and chromosome walking and was found to be largely collinear with two other lepidopteran genomes. The sequence information greatly narrows the physical extent of *H*. The region includes several candidate genes that have been implicated in wing coloration or patterning. Population data revealed a strong peak of morph-associated SNPs in a sub-region of approximately 130 kb that centres on *en*, the only gene for which the McDonald–Kreitman test revealed non-neutral variation. These findings make the *en* transcription factor the strongest candidate for *H*. The genomic approach therefore refines earlier studies that did not have sufficient resolution and suggested the neighbouring paralogue *inv* as probable candidate [14]. Functional validation, including expression analyses, will be required to further elucidate wing pattern determination and confirm *en*'s role as the *P. dardanus* mimicry gene.

Gene synteny of the *H* region in *P. dardanus* was found to be preserved with other Lepidoptera, which refutes the postulated chromosomal translocations of pattern-determining genes in the formation of the mimicry locus. The genomic architecture of *H* contrasts markedly with results in the polymorphic mimic *H. numata* [53], which revealed major chromosomal inversions over a 400 kb interval in its switch region, apparently maintained by balancing selection. Recombination within the scanned *P. dardanus* interval can readily be obtained in genetic crosses, e.g. for ff. *cenea* and *hippocoon* (figure 1)*.* In addition, we did not observe perfect LD (*r*^2^ = 1) between SNPs in different genes (except for SNPs specific to f. *lamborni*), which further supports *en* being the sole player underlying polymorphic mimicry in *P. dardanus*. However, our study of SNP association and LD is based on a set of distant sites representing the protein-coding genes only, which may have prevented the detection of perfect LD and genomic rearrangements on a narrower spatial scale, such as the regulatory region of *en*. A single-gene switch was confirmed for the parallel study of *P. polytes* that demonstrated a sharp peak in phenotypic associations with the *dsx* gene alone [16]. Denser sequencing coverage beyond the currently examined exons will also be needed for *P. dardanus* to determine the precise extent of non-neutral evolution and LD.

The *en* locus could transcriptionally control developmental differences between morphs, in accordance with findings in other mimetic butterflies, e.g. in *Heliconius*, where non-coding elements near the *optix* gene control the wing phenotype [44]. Variation in *cis-*regulatory elements of a single gene has also been shown to affect the positions of complex melanic spots on the wing in *Drosophila guttifera* [54] and distinct *cis*-regulatory elements of the *agouti* gene each control aspects of mouse coat colour [55]. In *P. polytes*, the detailed sequencing did not provide the resolution needed to reveal the functional mutations, as alleles are divergent due to apparent balancing selection and show clear LD [16]. Here, we also find divergent alleles, indicative of balancing selection, and notably the McDonald–Kreitman test demonstrated this phenomenon on the coding region, which may suggest the involvement of structural gene mutations, in addition to regulatory variation. The distribution of sequence variation in both species of *Papilio* is consistent with a refined supergene hypothesis involving multiple sites in accordance with a ‘beads-on-a-string’ linear array of functional sites envisioned by Clarke and Sheppard [11], but within a single gene. This perspective would reconcile the supergene hypothesis with Fisher's view [3] that a single locus could acquire control of discrete phenotypic variation by successive fixation of modifier alleles that gradually improve mimetic resemblance.

It remains to be established what would cause the reduced rate of recombination in *en*, as predicted by the supergene hypothesis, and which has been clearly established in *P. polytes* due to an inversion of the *dsx* region [15]. Structural variation within the *H* region remains elusive although the large insertion in f. *lamborni* that is in perfect association with the phenotype is intriguing. The insertion may alter the gene expression of the intact *en* copy, with the specific insertion site resulting in the *lamborni* phenotype, or the duplication may be altogether non-functional but it is maintained in the population as a by-product of selection on nearby sites. In any case, the apparent absence of such features in the other morphs does not preclude the existence of smaller scale rearrangements or other recombination-reducing features that would indicate the cooperation of multiple sites in producing the phenotype.

In conclusion, the exciting studies of *P. dardanus* of the mid-twentieth century lost momentum as classical genetics approaches were exhausted [48]. Genomic analyses now provide new possibilities for studying the molecular function and evolutionary history of the mimicry switch. The localization of *H* in the vicinity of the *en* locus suggests that a transcription factor might act as a developmental switch that controls the striking adaptive diversity of *P. dardanus*. Preliminary experiments [17] implicated the adjacent *inv* locus, but the resolution of that study could not distinguish between these two genes. Higher resolution of SNP variation may still show the involvement of regulatory regions of *inv*, and given their functional similarity and physical linkage the *en* and *inv* genes combined may have provided an evolutionary blueprint for generating phenotypic diversity that permit both changes of large effect and small additive mutations. The *en*/*inv* region may exemplify Turner's [15] ‘largesse of the genome’, i.e. the idea that certain genomic regions are predisposed to mediate integrated shifts in phenotype after multiple evolutionary steps at linked sites. Apparently, there is a wealth of such regions, as the same mechanism of mimicry switching in the congeneric *P. polytes* involves a different locus. Surprisingly, in both cases this mechanism applies to loci that are central to early embryonic development, and one might therefore expect them to be greatly constrained functionally, rather than being subject to accumulating high levels of variation for patterning of peripheral body structures.

## Supplementary Material

Electronic supplementary material

## Data accessibility

DNA sequences: GenBank accessions: FP243376, FM995623, FM955425, FP243362, JF299266–JF299270, JF299272, JF299278–JF299339, JF299341, JF299347–JF299409, JF299411, JF299417–JF299480, JF299482, JF299488–JF299551, JF299553–JF299622, JF299624, JF299630–JF299689, JF299691, JF299697–JF299760, JF299762, JF299768–JF299828, JF299830, JF299836–JF299844, JF299846, JF299852–JF299915, JF299917, JF299923–JF299985, JF299987, JF299993–JF300055, JF300057, JF300062–JF300119, KC747562–KC747569, KC747571–KC747614, KC747620–KC747630, KC988417–KC988422, KC988424, KC988430–KC988488, KC988640–KC988642, KC988644–KC988684, KC988689–KC988693, KC988695–KC988709, EF561094, EF561096, EF561098, EF560983–EF560990, EF561050–EF561056, EF561099, EF561097, EF561095, EF561101, EF561103, EF561106–EF561110, EF561037–EF561043, EF560938, EF560940–EF560942, EF560944–EF560946, EF561090–EF561092, EF561072–EF561074, EF561076–EF561088, KF114280–KF114310, KC988385–KC988947.

Wing disc transcriptome data: Sequence Read Archive: SRX014403. DNA sequence assembly of the *H* region is uploaded as the electronic supplemental material. *Papilio dardanus* f*. lamborni* mapped 454 reads (LR-PCR): Dryad doi:10.5061/dryad.s279c.
