# Supplementary material for: Comparative genomics of the mimicry switch in Papilio dardanus
Source: Proc Biol Sci. 2014 Jul 22;281(1787):20140465. doi: 10.1098/rspb.2014.0465 (PMC4071540; doi:10.1098/rspb.2014.0465)
Supplement: Electronic supplementary material [file rspb20140465supp1.docx]

**Supplementary Data**

1. Specimens voucher numbers
2. Oligos used for PCR amplification
3. Data used for McDonald-Kreitman tests
4. Supplementary information figure 1
5. Supplementary sequence
6. **Natural History Museum London (BMNH) voucher numbers of specimens used:**

| **Voucher nr** | **Subspecies** | **morph** | **Year** | **Location in Kenya** |
| --- | --- | --- | --- | --- |
| 746581 | polytrophus | poultoni | 2002/2003 | Mount Kenya |
| 746590 | polytrophus | poultoni | 2002/2003 | Mount Kenya |
| 746591 | polytrophus | poultoni | 2002/2003 | Mount Kenya |
| 746592 | polytrophus | poultoni | 2002/2003 | Mount Kenya |
| 746594 | polytrophus | poultoni | 2002/2003 | Mount Kenya |
| 746602 | polytrophus | proto(planemoides) | 2002/2003 | Mount Kenya |
| 746604 | polytrophus | poultoni | 2002/2003 | Mount Kenya |
| 746611 | polytrophus | hippocoon | 2002/2003 | Mount Kenya |
| 746612 | polytrophus | hippocoon | 2002/2003 | Mount Kenya |
| 746613 | polytrophus | hippocoon | 2002/2003 | Mount Kenya |
| 746614 | polytrophus | hippocoon | 2002/2003 | Mount Kenya |
| 746615 | polytrophus | hippocoon | 2002/2003 | Mount Kenya |
| 746616 | polytrophus | hippocoon | 2002/2003 | Mount Kenya |
| 746617 | polytrophus | hippocoon | 2002/2003 | Mount Kenya |
| 746618 | polytrophus | hippocoon | 2002/2003 | Mount Kenya |
| 746619 | polytrophus | cenea | 2002/2003 | Mount Kenya |
| 746620 | polytrophus | cenea | 2002/2003 | Mount Kenya |
| 746621 | polytrophus | cenea | 2002/2003 | Mount Kenya |
| 746622 | polytrophus | cenea | 2002/2003 | Mount Kenya |
| 746623 | polytrophus | cenea | 2002/2003 | Mount Kenya |
| 746624 | polytrophus | cenea | 2002/2003 | Mount Kenya |
| 746625 | polytrophus | cenea | 2002/2003 | Mount Kenya |
| 746626 | polytrophus | cenea | 2002/2003 | Mount Kenya |
| 746695 | polytrophus | poultoni | 2002/2003 | Mount Kenya |
| 746696 | polytrophus | poultoni | 2002/2003 | Mount Kenya |
| 746699 | polytrophus | poultoni | 2002/2003 | Mount Kenya |
| 746700 | polytrophus | poultoni | 2002/2003 | Mount Kenya |
| 746702 | polytrophus | poultoni | 2002/2003 | Mount Kenya |
| 746703 | polytrophus | poultoni | 2002/2003 | Mount Kenya |
| 746704 | polytrophus | poultoni | 2002/2003 | Mount Kenya |
| 746705 | polytrophus | poultoni | 2002/2003 | Mount Kenya |
| 746706 | polytrophus | poultoni | 2002/2003 | Mount Kenya |
| 746707 | polytrophus | poultoni | 2002/2003 | Mount Kenya |
| 746708 | polytrophus | poultoni | 2002/2003 | Mount Kenya |
| 746744 | polytrophus | cenea | 2002/2003 | Mount Kenya |
| 746745 | polytrophus | lamborni | 2002/2003 | Mount Kenya |
| 746746 | polytrophus | cenea | 2002/2003 | Mount Kenya |
| 746747 | polytrophus | cenea | 2002/2003 | Mount Kenya |
| 746748 | polytrophus | cenea | 2002/2003 | Mount Kenya |
| 746749 | polytrophus | cenea | 2002/2003 | Mount Kenya |
| 746750 | polytrophus | cenea | 2002/2003 | Mount Kenya |
| 746751 | polytrophus | cenea | 2002/2003 | Mount Kenya |
| 746773 | polytrophus | lamborni | 2002/2003 | Mount Kenya |
| 746774 | polytrophus | lamborni | 2002/2003 | Mount Kenya |
| 746775 | polytrophus | lamborni | 2002/2003 | Mount Kenya |
| 746776 | polytrophus | lamborni | 2002/2003 | Mount Kenya |
| 746777 | polytrophus | lamborni | 2002/2003 | Mount Kenya |
| 746779 | polytrophus | lamborni | 2002/2003 | Mount Kenya |
| 746780 | polytrophus | lamborni | 2002/2003 | Mount Kenya |
| 746781 | polytrophus | lamborni | 2002/2003 | Mount Kenya |
| 746786 | polytrophus | lamborni | 2002/2003 | Mount Kenya |
| 746787 | polytrophus | lamborni | 2002/2003 | Mount Kenya |
| 746788 | polytrophus | lamborni | 2002/2003 | Mount Kenya |
| 746789 | polytrophus | lamborni | 2002/2003 | Mount Kenya |
| 746791 | polytrophus | lamborni | 2002/2003 | Mount Kenya |
| 746792 | polytrophus | lamborni | 2002/2003 | Mount Kenya |
| 746793 | polytrophus | lamborni | 2002/2003 | Mount Kenya |
| 746797 | polytrophus | hippocoon | 2002/2003 | Mount Kenya |
| 746798 | polytrophus | hippocoon | 2002/2003 | Mount Kenya |
| 746800 | polytrophus | hippocoon | 2002/2003 | Mount Kenya |
| 746801 | polytrophus | hippocoon | 2002/2003 | Mount Kenya |
| 746802 | polytrophus | hippocoon | 2002/2003 | Mount Kenya |
| 746804 | polytrophus | hippocoon | 2002/2003 | Mount Kenya |
| 746805 | polytrophus | hippocoon | 2002/2003 | Mount Kenya |
| 746806 | polytrophus | hippocoon | 2002/2003 | Mount Kenya |
| 843035 | polytrophus | cenea | 2010 | Mount Kenya |
| 843039 | polytrophus | cenea | 2010 | Mount Kenya |
| 843040 | polytrophus | cenea | 2010 | Mount Kenya |
| 843041 | polytrophus | cenea | 2010 | Mount Kenya |
| 843043 | polytrophus | cenea | 2010 | Mount Kenya |
| 843044 | polytrophus | cenea | 2010 | Mount Kenya |
| 843034 | polytrophus | hippocoon | 2010 | Mount Kenya |
| 843045 | polytrophus | hippocoon | 2010 | Mount Kenya |
| 843036 | polytrophus | lamborni | 2010 | Mount Kenya |
| 843037 | polytrophus | lamborni | 2010 | Mount Kenya |
| 843048 | polytrophus | poultoni | 2010 | Mount Kenya |
| 843049 | polytrophus | poultoni | 2010 | Mount Kenya |
| 843047 | polytrophus | (proto)planemoides | 2010 | Mount Kenya |
| 843046 | polytrophus | poultoni | 2010 | Mount Kenya |
| MJ07-1156 | tibullus | hippocoon | 2007/2008 | Buda Forest, Nguruweni |
| MJ07-1157 | tibullus | hippocoon | 2007/2008 | Buda Forest, Nguruweni |
| MJ07-1032 | tibullus | hippocoon | 2007/2008 | Gedi ruins, Watamu |
| PM-46 | tibullus | hippocoon | 2007/2008 | Kipepeo, Watamu |
| MJ07-1152 | tibullus | hippocoon | 2007/2008 | Mrima forest |
| MJ07-1053 | tibullus | hippocoon | 2007/2008 | Pemba Channel Lodge, Shimoni |
| MJ07-1102 | tibullus | hippocoon | 2007/2008 | Shimoni forest East, Shimoni |
| MJ07-1037 | tibullus | hippocoon | 2007/2008 | Sokoke Forest, Watamu |
| PM-5 | tibullus | hippocoon | 2007/2008 | Taita hills |
| PM-223 | tibullus | hippocoon | 2007/2008 | Watamu |
| PM-34 | tibullus | lamborni | 2007/2008 | Kipepeo, Watamu |
| Strat08L |  | lamborni | 2009 | COMMERCIALLY OBTAINED |
| 740200 | dardanus | planemoides | 1998 | Kakamega, Kenya |
| 740201 | dardanus | planemoides | 1998 | Kakamega, Kenya |
| 847251 | dardanus | planemoides | 1998 | Kakamega, Kenya |
| PM-30 | tibullus | poultoni | 2007/2008 | Kipepeo, Watamu |
| PM-131 | tibullus | poultoni | 2007/2008 | Watamu |
| PM-264 | tibullus | poultoni | 2007/2008 | Watamu |

1. **Primers used for PCR:**

| **FOR:** | **NAME** | **ORIGIN** | **TARGET REGION** | **SEQUENCE (5' - 3')** |
| --- | --- | --- | --- | --- |
| RECOMBINATION | PD15 | BAC TILE PATH | CTD | AAYCCGTTACAAATTGGAATT |
| ANALYSIS | 1D8_F0 | BAC TILE PATH | CTD | GAGGACGTGCGACCTTACAT |
|  | PD52 | BAC TILE PATH |  | TCCGACCTTGCGAAGTATTCGGATT |
|  | PD54 | BAC TILE PATH |  | CCGTTCGGTTGTGTGAGTGTGTGTT |
|  | PD32 | BAC TILE PATH |  | AGCGCCTCGACATGGACTAT |
|  | PD33 | BAC TILE PATH |  | GCGGTACGTCTCCAGGTAAT |
|  | PD13 | BAC TILE PATH | ORANGE | ACAGCAAATAATAAAAGARACNTTYCA |
|  | PD16 | BAC TILE PATH | ORANGE | GCCTCSGCYTTYTGCAT |
|  | PD88 | BAC TILE PATH |  | ACAATACTCGCAACGGAAGG |
|  | PD89 | BAC TILE PATH |  | GAAAGCGGGAAGACAACAAT |
|  | PD121 | 454 DERIVED |  | GGAGATGGCACCGATCAT |
|  | PD122 | 454 DERIVED |  | CGAAGACCAATCAGCACTGT |
|  | PD227 | 454 DERIVED |  | CAGGAGCCGTAGCTGACTTC |
|  | PD228 | 454 DERIVED |  | CAGCTGATGCAAAACGGTAG |
| GENETIC | PD115 | BAC TILE PATH | CBP | CCATATCCTTCTGTGGTGGA |
| ASSOCATION | PD117 | BAC TILE PATH | CBP | GAGATAGGTCCGGGTATAGA |
|  | PD130 | BAC TILE PATH | SCF | TCGCTAGAAAAATCCGGTTG |
|  | PD131 | BAC TILE PATH | SCF | AGCCCCTTCTTCTTTGTGGT |
|  | PD126 | BAC TILE PATH | ORANGE | TTTCTGCATCTTATTCTGTTCCTG |
|  | PD127 | BAC TILE PATH | ORANGE | TCTTTGTGGAAACCCTCGAT |
|  | PD128 | BAC TILE PATH | ENGRAILED | GCTTTCGAGGATCGCTGTAG |
|  | PD129 | BAC TILE PATH | ENGRAILED | ACTAGGTCGGTCGCTGTACC |
|  | PD144 | BAC TILE PATH | ENGRAILED | CAGCGGCGGCACGCTCGAG |
|  | PD47 | BAC TILE PATH | ENGRAILED | CAATTGGCTTACTCGGCACT |
|  | PD48 | BAC TILE PATH | ENGRAILED | CACTCTCAGTTGCGGTGCTA |
|  | PD197 | BAC TILE PATH | INVECTED | TTYGCTGAGAACCGTTMCCT |
|  | PD198 | BAC TILE PATH | INVECTED | TTTCTCTGGCCTTCATCTCMA |
|  | PD9 | BAC TILE PATH | INVECTED | ATGATAAAGATTCAAGATCA |
|  | PD10 | BAC TILE PATH | INVECTED | ACTGGAGCCACGTTACCAA |
|  | PD111 | BAC TILE PATH | INVECTED | GGATAAGGATYATYTTGTT |
|  | PD132 | BAC TILE PATH | ALDOSE REDUCTASE | ACGCGGTATAGCAACGATTC |
|  | PD133 | BAC TILE PATH | ALDOSE REDUCTASE | CGCCATTTTTCCACTTGAGTA |
|  | PD200 | BAC TILE PATH | CBPA | CGTATTAGGATCGACTCCTTCG |
|  | PD201 | BAC TILE PATH | CBPA | TGTTCCTATTGGGATCTGTTCTC |
|  | PD227 | 454 DERIVED | HYPOTHETICAL PROTEIN | CAGGAGCCGTAGCTGACTTC |
|  | PD228 | 454 DERIVED | HYPOTHETICAL PROTEIN | CAGCTGATGCAAAACGGTAG |
|  | PD134 | 454 DERIVED | CTD | CTCACTTCGCCGAGTTCCT |
|  | PD241 | 454 DERIVED | CTD | AACACCGCGTATTTCCTC |
|  | PD235 |  | DECAPENTAPLEGIC | CAGACTTTAGTGAACTCAGTGAACC |
|  | PD236 |  | DECAPENTAPLEGIC | TTTCGAGGCCGCAACTAT |
|  | PD112 |  | WINGLESS | TTGCTGGATGCGCTTGCCGAGTTTCCG |
|  | PD113 |  | WINGLESS | CTTGTTYTCGGTYTTGTTRCCGC |
|  | PD262 |  | RpS19 | CATATGGATCTGGTGAAG |
|  | PD263 |  | RpS19 | ATCTGCAGAAGTGTGATG |
|  | PD264 |  | CDP | TTGACGAGTACGTCCAAWCG |
|  | PD265 |  | CDP | ACCGGTCAGCTGATTGAAGT |
| LR-PCR | PD100 | BAC TILE PATH |  | GGGTGGCTGGGGTTGCCAGA |
|  | PD107 | BAC TILE PATH |  | CGGTCTCAATAAACCGGACCA |
|  | PD73 | BAC TILE PATH |  | GCGTGCAAATGAATTTATGGCGGG |
|  | PD74 | BAC TILE PATH |  | TTGGACCCACATTTCAGACCC |
|  | PD82 | BAC TILE PATH |  | TCAAATCAGCGCACAGCCA |
|  | PD76 | BAC TILE PATH |  | CCGACCACGTAGCTGCACA |
|  | PD75 | BAC TILE PATH |  | GCTGGAGATTCTGCGCGGGAAAA |
|  | PD84 | BAC TILE PATH |  | CGTCCCAGGCAAAGACTTGA |
|  | PD83 | BAC TILE PATH |  | TGCAAGTTTCGGCAGCAAA |
|  | PD78 | BAC TILE PATH |  | CCTTGCCACTGGGCGTTC |
|  | PD77 | BAC TILE PATH |  | AAAAGCAAACCCGGCGGT |
|  | PD62 | BAC TILE PATH |  | TCGCGTTGATTACGTCCATA |
|  | PD47 | BAC TILE PATH |  | CAATTGGCTTACTCGGCACT |
|  | PD72 | BAC TILE PATH |  | TCTCGCGCAGAAAACGGG |
|  | PD70 | BAC TILE PATH |  | AGGACGACACGGGGACGA |
|  | PD96 | BAC TILE PATH |  | GCGATTGTATAGCGAGCGACAA |
|  | PD71 | BAC TILE PATH |  | TGGCTTAGGGGTTAAGCACTTG |
|  | PD44 | BAC TILE PATH |  | CTTCTTCGAGCCAATCTTCTG |
|  | PD54 | BAC TILE PATH |  | CCGTTCGGTTGTGTGAGTGTGTGTT |
|  | PD68 | BAC TILE PATH |  | CGTGGACAGGAGATGATCGGAGTCG |
|  | PD66 | BAC TILE PATH |  | TGTCTCCCGCAGCTCCGCCT |
|  | PD67 | BAC TILE PATH |  | TGGCCAGTACCGCACTTCGTTTGA |
|  | PD64 | BAC TILE PATH |  | CCAAACCCAATGTTTATTGACC |
|  | PD65 | BAC TILE PATH |  | AGGACTCGGCAGACTTATTCAGCATTT |
|  | PD58 | BAC TILE PATH |  | GGCGGTGACGCGGCCAAAT |
|  | PD59 | BAC TILE PATH |  | CCGCGTAGCGAACCAAAGTGGG |
|  | PD56 | BAC TILE PATH |  | TCGGCCTGTTCCATCGTGTTAATCG |
|  | PD57 | BAC TILE PATH |  | CGAAATCCATGATGTACGATGC |
|  | PD63 | BAC TILE PATH |  | AAGATAAGCCCGAAGATTACCG |
|  | PD54 | BAC TILE PATH |  | CCGTTCGGTTGTGTGAGTGTGTGTT |
|  | PD52 | BAC TILE PATH |  | TCCGACCTTGCGAAGTATTCGGATT |
|  | PD33 | BAC TILE PATH |  | GCGGTACGTCTCCAGGTAAT |
|  | PD32 | BAC TILE PATH |  | AGCGCCTCGACATGGACTAT |
|  | PD201 | BAC TILE PATH |  | TGTTCCTATTGGGATCTGTTCTC |
|  | PD200 | BAC TILE PATH |  | CGTATTAGGATCGACTCCTTCG |
|  | UBR | BAC TILE PATH |  | TTCAGTTTATTTAAYGGCATC |
|  | PD37 | BAC TILE PATH |  | CACCACCTTCCAGTTCCAAT |
|  | UBF | BAC TILE PATH |  | CGGAAGARGACTGTTACAGCA |
|  | PD262 | BAC TILE PATH |  | CATATGGATCTGGTGAAG |
|  | PD263 | BAC TILE PATH |  | ATCTGCAGAAGTGTGATG |
|  | PD264 | BAC TILE PATH |  | TTGACGAGTACGTCCAAWCG |
|  | PD265 | BAC TILE PATH |  | ACCGGTCAGCTGATTGAAGT |

1. **Data used for McDonald-Kreitman tests, including Jukes Cantor corrections.**

1. **Supplementary information for Figure 1**

Protein coding genes predicted in scanned interval, from left to right: IMFamide, Myosin-Va, Sodium nucleoside cotransporter, hypothetical protein, Mothers against Dpp (MAD), Zinc finger transcription factor, Uncharacterized zinc finger protein, Guanine nucleotide-binding protein G(s), 5 x Core histone H2A/H2B/H3/H4, Sanpodo, Star protein, Carotenoid-binding protein (CPB), Linker histone H1 and H5 family, 2 x Uncharacterized protein, Free Aspartyl-tRNASynthetase, Solute carrier family 39 member, Orange, Engrailed, Invected, 2x Aldehyde Reductase (AR), Chitin binding Peritrophin-A domain (CbpA), Hypothetical protein (Ubiquitin superfamily), Carboxy-terminal domain RNA polymerase II polypeptide A small phosphatase 2 (CTD), Hypothetical protein.

1. **Supplementary sequence.** Assembled sequence of *H* region used throughout the study.

LOCUS ACT_BAC 396207 bp DNA linear UNA 16-DEC-2013

DEFINITION Papilio dardanus BAC sequences, clones 10A24, 19F6 and 9M9.

ACCESSION

VERSION

KEYWORDS .

SOURCE Papilio dardanus (African swallowtail butterfly)

ORGANISM Papilio dardanus

Eukaryota; Metazoa; Arthropoda; Hexapoda; Insecta; Pterygota;

Neoptera; Endopterygota; Lepidoptera; Glossata; Ditrysia;

Papilionoidea; Papilionidae; Papilioninae; Papilio.

FEATURES Location/Qualifiers

misc_difference 56499

/label="START_BAC"

CDS join(59776..59846,60282..60388,60960..61074,62152..62224)

/note="hypothetical protein"

/label="hypothetical protein CDS"

CDS join(67630..67728,69716..69810,70162..70330,70886..70983,

71519..71633,72506..72636,72950..73118,73661..73858)

/note="putative Guanine nucleotide-binding protein G(s)

subunit alpha (Adenylate cyclase-stimulating Galpha

protein)"

/label="CDS"

CDS complement(74783..75181)

/note="hypothetical protein"

/label="hypothetical protein CDS"

CDS complement(75844..76224)

/note="hypothetical protein. Core histone H2A/H2B/H3/H4"

/label="CDS"

CDS 76674..76985

/note="hypothetical protein. Core histone H2A/H2B/H3/H4"

/label="CDS"

CDS 77969..78379

/note="putative Chain e, Structure Of The 4_601_167

Tetranucleosome. H3 histone"

/label="CDS"

CDS complement(80351..80716)

/note="hypothetical protein. Core histone H2A/H2B/H3/H4"

/label="CDS"

misc_feature 82217..83416

/label="Putative Sanpodo"

CDS join(86800..86875,88950..89098,89669..89827,90456..90509,

90968..91090)

/note="hypothetical protein similar to cholesterol

transporter BmStart1"

/label="CDS"

CDS join(100152..100358,101916..102071,102813..102917,

103068..103273,104690..104802,105668..105777)

/note="putative carotenoid-binding protein"

/label="carotenoid-binding protein"

CDS complement(109711..110421)

/note="Hypothetical Protein. Llinker histone H1 and H5

family. Expect 4.8e-24"

/label="CDS"

CDS join(116442..116449,116654..116685,116740..116860,

116928..117007,117116..119241,119387..119615,

120135..120181,120678..120851)

/note="hypothetical protein. (CV175329)

PAPILIOdpolyt_D07_1_059 Papilio dardanus cDNA"

/label="CDS"

CDS join(complement(124800..124888),

complement(124433..124596),complement(124171..124346),

complement(124006..124094),complement(123518..123938))

/note="unknown protein"

/label="unknown protein CDS"

repeat_region join(complement(130777..132327),

complement(127134..130718))

/note="putative Lian-Aa1 retrotransposon protein"

/label="Lian-Aa1 retrotransposon protein"

CDS join(136214..136359,136973..137150,137917..138091,

138342..138356,138735..138886,139886..140052,

140744..140901,141889..142043,142825..142936,

143332..143391,144316..144419)

/note="putative Free Aspartyl-Trna Synthetase (Asprs)

(E.C. 6.1.1.12) From Yeast"

/label="CDS"

CDS join(complement(151919..152326),

complement(150762..151834),complement(150439..150575),

complement(150261..150341),complement(149827..150014),

complement(149321..149467))

/note="putative solute carrier family 39 (zinc

transporter), member 10"

/label="CDS"

CDS join(188250..188318,188391..188482,188552..188663,

189556..189735,190094..190201,190932..191085,

191171..191439)

/note="ORANGE. putative clathrin coat assembly protein"

/label="orange"

repeat_region 236112..236627

/note="putative reverse transcriptase"

/label="reverse transcriptase"

CDS join(249309..250080,264700..264791,265289..265522)

/note="putative engrailed"

/label="engrailed"

repeat_region 274940..276604

/note="putative non-LTR retrotransposon R1Bmks ORF2

protein"

/label="non-LTR retrotransposon R1Bmks"

repeat_region join(complement(279114..279125),

complement(277539..278516))

/note="putative reverse transcriptase"

/label="reverse transcriptase"

CDS join(complement(341601..342678),

complement(341052..341132),complement(300465..300568),

complement(299974..300240))

/note="invected homeodomain"

/note="Bombyx invected (Bm in) mRNA, complete cds"

/label="invected"

CDS join(361752..361760,363153..363236,365646..365828,

366531..366647,367105..367272,367491..367694,

367969..368226)

/note="putative Aldose reductase (AR) (Aldehyde

reductase)"

/label="CDS"

CDS join(370141..370152,370885..370986,371672..371782,

372405..372575,372898..373095,373267..373375,

374139..374215)

/note="putative 3-dehydroecdysone 3beta-reductase"

/label="CDS"

CDS join(complement(377639..377714),

complement(376092..376318),complement(375779..375892),

complement(375550..375673),complement(375134..375272),

complement(374625..374795))

/note="Chitin binding Peritrophin-A domain 98.0 1.9e-25 2"

/label="CDS"

CDS join(378931..379933,380375..380442)

/note="Hypothetical Protein"

/label="Hypothetical Protein CDS"

CDS complement(396080..396142)

/note="similar to CTD (carboxy-terminal domain, RNA

polymerase II, polypeptide A) small phosphataselike 2"

/note="ORF1"

/label="ORF1 CDS"

ORIGIN

1 ggcccctcga cccgccgcgc gtggccacct cgcctgcagc tagaccctcc tacgtcgtca

61 aatctacatc cctttcccga caattccttc actggtatgg agctgtggtc tgttttagtc

121 atgccatgtc accgtcagtg tgtaaaaata tttatttttt ataaaatgac cgcaatataa

181 accaatcacc taagcgtttt aacgccttaa acatagacta tgttttaaca tcattgaaga

241 tacataataa aagcatttta ttgcgccatt atgtagtgat tttttacata agcattgtag

301 tcaatagata ggaagaactt tgttagatcc aaatacatac tatgttgttt atacatatat

361 tttgcgaaaa aaatatctca caaaatcaac ggattataaa gtaactgtgg gtttcgacat

421 ttggaaacat attgtctcag ttatttcaaa gagaatgaaa gggatagcaa taggtgtcaa

481 agcacagaaa ggcggtgtaa ctcgattatg tttatatgtc gcattagata taggattaat

541 tagcataatc atttaatagt gctaaaacaa ctgaccttat catgaaacag ataaacttgg

601 ataattagtt aactataaca gtaagtaaat aaggcaaatg gagatatata taacgtgacc

661 agacgtccct ttttaaacgg gactgtcccg tattttagtc atgagtcccg gtgtccccaa

721 tataaactct gggacgccaa attgtcccgt tttctgatat cgaaatgtcc cgttttgagt

781 aataaaaaat ggccacgtta gttatataac actcataata aaggatggct cacgataccc

841 catgtcccac ccgggatgta ccaagtgtgt caattcaata tatacaaatg tgacggacgg

901 agcactgaca aaaaatattt tgtatggata tggatacact gatggctcgg acggaacccg

961 gccgggacac ggtgaactgt acgtcatcct taacactctt cgttgagttt acgtcaatcg

1021 gcgacattaa tgtctaagtg gcgtaatcgt tgcaaaatta gtgattttat atagccaaag

1081 gtttgcatga atgtttgttt gaaggtatcc agaaatcgat ggatatcgac aaaagacaac

1141 gatatatgta ttttagtaga atagcaagta acgagtaaac gagtgtacgg acacgtttag

1201 ttcctaaact cgactaaaat actatcctgg ggtgttaccc aaagtatctt tggcgatgac

1261 tgacaatgac attcgatagg tcacgtgttt gccacgtgta agatatcgaa ggtcattgtc

1321 atttccatac acttttgatg ttcgttatca aatcgctttg tagcgcatgt actttggata

1381 atacccctgg tgcatacaaa gcttaacata ctataattac agatttgagt agcctcgtgt

1441 gcgaagaccc taataacaca tgcaatattt atttacttgt taacaataca tactcgtttg

1501 tttttgtaat atacaaaaca aggaaaccag acttgcttgc atttatttta actcttttgt

1561 aaatagagtc aagtttattt tattatacca caagatatta catgtccttt tttatatgaa

1621 acccttttaa atacttactg caataatatt aaaaaatctt accctacaag aaagtaattt

1681 taaaatggta tgtaaacgaa atgcagttgc ttggcaggag acagcgcaag ataggcgacg

1741 ctggcgttct ctcgtgtcgg aggccaagac ccactttggg tcgctgcgcc agcggagtaa

1801 gtaagtaagt atgtaaacga aattagggtg gataactaat ttaataatat attgtatcaa

1861 tgacgagttc agtcggacct ggataaccga gagtccaagg gaccgcaata ggattctcgc

1921 ttatagaggt taccctctca tccttgcctc gtctatccat gtttgactgt ataaaaattg

1981 aaatgcagaa aaaaacggta gtcgatttat aaaaataaat ttattatctg taaccacttg

2041 gtaatacaaa ctttaatatt aatccttata acattaaact tattagaata atctctaacc

2101 agttgacaac acaagcaaga acaaagtaaa acgagccaat aagactcaaa ttgcaacatt

2161 ttcgaaaaat actttgcctt ctatactaat attataaaga gagaaacttt gtatgtttgt

2221 attgaaataa ctcaagaact actggaccga tttcaaaaat tctttcacca ttcgaaagct

2281 agattatccc cgagtaacat aggctatatt atttaattgc acactgacgt agtcgcgcac

2341 aacggctagt ctcttagtaa ttgtatgata tacagatata caatacataa ctctcctgtc

2401 aaatacaaaa tattatattt tcacatttac attcacaatg acgctttttc gacactcgta

2461 aaagcgttga aatagaacaa acgaagttct atgtatctgt taataaggca gcgcttttaa

2521 atcttgggct tttttatcat atgcattctg agcactaggt aaacaaatcc agactacttt

2581 taaaccaatt caatgagcag taaaaagcgt ttgtatatac acagactata atgcaacgtc

2641 ttacattatg aagtacataa tatgtgttat tatttacaga tttaatttta tataaatatc

2701 aattaatttg aatataaaaa tccggtaaac acttcaaagt gatgattcaa tgatggtata

2761 gtttgaagtt tagtttttga ttttaatgca cccaaatacg tattgtctaa acaaaagaat

2821 attctcttca ttcttgcatg tactaatgcc attgaacaca tgaagcaagg ctctcttatt

2881 aaatagatat aataaccagt acataagtat ggacctagtt tattcttatt gcaatcactt

2941 gcactatcag ttttacttat gtattctctt gaaccaaatt taacactggg aaattttaac

3001 tttaaatatg tcaacaattc atcactaaca ctaactgtct cactacattc gtagtcactc

3061 tcaaaccaaa tgccaccgtt ttgtgtcttc gcaacattgt caatagctaa cattgcagaa

3121 tgttgaattg gatttttgtt acgattgtca aaagatacag ccactactga ctgtattgta

3181 ggatcgacaa ctatagctcc gttaatattc gtgtagacat ctccaataga attcgtagta

3241 tcagtatgta gtatgaacca tctggaaact tcaaaggcca tcttcatgta tgttttatga

3301 tagttaagtt cattagaact gaagaacttt ccgcaagtaa gtctctctaa ataaggattg

3361 gggtgaaagt tgcaagacca aatttttgaa aattccttgt tttgtttttg tattttaggt

3421 gcagtaagtg gaacctccat ctctttgaaa tactgaaatg catcttttag ttctatgaca

3481 gtgttttcga tgaattcttg tatagatgag acgccaacaa tataactcgc cggacacaat

3541 attacctgct tttttctcac tctcttcaaa tgctgtaact gtttcaatgg aactttttca

3601 ttcaatgttg taacaatttt tgataaatct ttagcatctt tggtgatacc gacaaacacc

3661 ttcagtaatt ctgcattctc actgaaatca tcacctaaaa ctggttctaa attatttaca

3721 gaattatcaa tatcttctat acatttgtcg atcgctttca tcataaaatc attgtcttgt

3781 aaaatcattt taattttttt cataggtggt tctattccaa ccgacatagt cattacgaga

3841 ggtcttgaaa tccacgggtg ttctgtcaca actgaaaaga gagaacaata ccactttttc

3901 atcacccact gtattttaat cactttcaaa cataaagcac gaaaaacaca tttctttaca

3961 attccgatta cgaaaaaccc gacaatacga cataaatatt gttaataaca aatgcaatac

4021 ctttaaatag ttgtacaata aaatcacaat tacgagagca tcaacaactg tccggctgtt

4081 tgtttgatac tgataacgta tcattttgta ctctccttgt gcacataacc tcaaggtaca

4141 ccactgaagg acaagttcat ggaattttat ataatcgaaa acaaaatatt atttactagc

4201 tctatttgat ggtcatttaa atacacttaa actgtacaat ttacttcccc ctgatactac

4261 agaagtacac catataaaaa tatctttggc caccacataa acaattgcct catgaaatct

4321 ttaattgcat acctaattcg tttcaacgat aaaataagca taaaaataag taatttgcga

4381 caaatttacc tcgcttcaga tttttcaaat cacaatagaa gacacgacgt aacaatttct

4441 ttaacaatag tcacacatta attaacatca agtggtttaa ccaccttgat ataatttatc

4501 aatacgaaac cgataaaaga atttaatttg ccacacatgt aaaacgccaa agaacagcag

4561 ccaccattag gcaatgctgt gtttccgatt acaaaaccat agaaactaac ttaattaaat

4621 attggcttat ttctgcttat ttgatttata tgtcaaatat caaccagtga tagacaagcc

4681 acagataatg taaataaatt tatgtaacct gtcgtaatag agaacagaca aacgcacgtt

4741 ccgttctacg tattgccgtt gacatatcta taataataaa actgtaaaaa gttaatcata

4801 acaaaaattg ctgctggtgc tgccaattta gaaattcatg ttcaaaaaca aataaagcac

4861 aacaaaatgc taaaaactga ttgacctagt tactttgcgt ttctggttta tataatcgtg

4921 aaattccact actgtaacac ttgtaggaat atatgtcgtt acctctattt gttcaaagaa

4981 aatgagttgg atgacaatac gttttaagaa ctgtatcgac ttggtttcga cggtggaaac

5041 ttacagtatg attttagtag gtttatgtat tgtattttgt aaattaataa tgcatttagt

5101 tgtagttaca acgatttatt taatcaagat aagattacag gctgccaaaa cttgtaggac

5161 attaaaactt tccttaataa gatacaaagg acaatatgcg caattttatt tcttgttttc

5221 tgatatttgg gagaaccacg tttggcaact ctcggaagcg atttcgcaga gcgctgacat

5281 ggccctctca gttgcgtcgt attctgaaat cataaagaaa cctttaagaa taaaaattta

5341 aacacttcaa aatatgtaaa aagtaaaatg actacgaatt ttgatgtctt ttaacaaaat

5401 ctagactaag tgatgcgctt aatgcaacct taaaagtctg ctgtgtggca gattgttgat

5461 gtttttaaaa aaatatgaaa atttttcata atagacaaat cacctgtatt tcctcgtttt

5521 ccaaagatgg atccattgaa aggtggcttc ctgtaggtgc cttcggtcga agccatcaga

5581 caaatgatca aggcgatgac gcagaaaaca acggctgcac gcatcttgga ttctgcaagc

5641 gtgtttatgt atttaaggag cattatttta aacaaaatta tatttgttaa aagtgcttct

5701 tattagtacg aatagagtaa ggtaaataat tttaaactag tacgtaccaa tgagaagtag

5761 gtaaagtttc aagcttttga tggtaacact ggaagatgtg agatgagtgt gatagcttag

5821 tattctagct cccagctttt atagcaaatc tcgagcgata ttttaattaa agtattactg

5881 agttagggca ccataatgga ataaaataca ttttacatgt ctggaaatat caaatttgta

5941 tttagtccca ctgcgccgcg aagaagaacg aactacttac actttgataa aaaaagtgga

6001 cgcaagtttt aaatgggaaa atcatgttta attctctctt tactgtgttt tcaaaaaata

6061 tcttttgacc atctcctatt ttatatgcaa ctatagacaa actcttatct aaataaactg

6121 ccattaagaa gggataatgc aaaagttcta agcatggttg gtttctatct aagttttaat

6181 cccctaaaga gtacggctct ttagatagcc aatatgcggt cagtattcaa taatccgtta

6241 attgaattta ctcttgagat gagaagtacg gacaacgttg tggtataaaa cggggaacag

6301 aaagcttctc agcatcagtc aagcttaagc ttccacagtg ttgtcagcct aaacacttca

6361 agttcccgaa acttatattt taaagtacta tattatttct caattcattt gtcttattgt

6421 aacatatgct taagctgtaa ttatattttc tagatggctg gacacgcgat gaaattcact

6481 gtctggttgg tttgcatgat ggcgatttta tttactgtga ttgaagtgag ccaggcaaat

6541 tacaaaaacg cgcctatgaa tggaatcatg ttcggtaaaa gggtaccttc tggtaaggct

6601 aaagatattt tcagatcata aaactctgta gcttgaatcg gtttttgcat tgactgtgac

6661 ttttaggatc tattcacact agtgtaagtc gaagttgagc ttacattact tagtataaat

6721 tgtgtgcaac taaaatacgt ttttatttca gaatacgaaa taaggagcaa aacgtttacg

6781 gcattatgtg aaattgcaac agaagcttgc caggcgtggt tcccttctca agaaaataaa

6841 taaaagttaa atagttaatt tacggtgaat gatcatacaa cggtttttgg aaatgaaatc

6901 atacttgaaa atgttataaa atatgtattt aattatataa atatgatttt aattatagat

6961 tcctgtacta tcatatttgt ttaactgcaa ttaagtacag tattatgttt tagaaccatt

7021 gcttaaataa agttacatac aaaaacttat atattgtttt attgttcttt acaaatcaat

7081 aactttaatt attattaggt atcgaaattt tcagagttaa gttgctttta tatggcaata

7141 tttttttcat aaaaattatt agatacagag taaacttatg aaaaggacag tataagatat

7201 cttcatttct taattaaaaa ttcagtgcag ttggacagtt aaaataaatt acaaaaaaan

7261 nnnnnnnnnn nnnnnnnnnn nnnnnnnnnn nnnnnnnnnn nnnnnnnnnn nnnnnnnnnn

7321 nnnnnnnnnn nnnnnnnnnn nnnnnnnnnn nnnnnnnnna aaaaaaaaaa actcattgac

7381 ttgataacct ccttctgttg gaagtcggtt aaaaatatta aattaaaata cgtgacacgg

7441 aattattgaa cggctggccc tgattcccta cttgacatat gtcaataaca atgcagtgac

7501 gtttcgcaat tcgtattgga aaaactttta taaggatttc gtatctacct tatttctaaa

7561 atgaattttc aaaggtctga acccgtggtg aaataatgta attgtgataa tttattaata

7621 tattaagttg gtgtattgtg aatctataaa aatgacgacg aaggaattat acaccaaggt

7681 aaaattttgt gatactgtgt tatgattgta ttttgtttaa ttttgttttt agatttatag

7741 acatatttaa gtttagaacc gcattaattt atgctgtgtg tttatttaaa taatattctg

7801 tttgaagtcg ttcattaaaa aaaatgatta catcagatga atactgcgtt ataatatttt

7861 tcagttaaat cgccttattt aagttttatc gtggctaatt aaagcttcct aacaataatt

7921 attcatttaa caactaaatt agtatcataa atataaaact taaccataaa ataacttata

7981 tatcatggca aaatcctatt cttatatcag atttgtaatt tatgtagcaa attcttacaa

8041 gacattcctt tcaaatagta ttgtttatga aaaggtgttt tttagataca gtatttgata

8101 ctgtaacatc aaagtaagta taggtaataa taaatattaa aaaaacatat tttttcttaa

8161 tcttaaaaaa aactatgaac tcaatttatc tacttttgaa atatgttgat ttttatacag

8221 cttattagat tcttattttg taaatattaa ccaaggatgt attggttgat ataaaatgtt

8281 atgcaaatca aacaggtttt tatgaatgtt ttgtgtctac attgtataga tatagaaggc

8341 ttgttttcaa cattttaaaa gttatttttt aatttggtag caaatggtaa tacagtcaaa

8401 cctgaataag taagacactt ctataagagt catcctctgg tcccaatgag ctacctccat

8461 aaccgagaaa ctcggattag agagagacct caataagcga gacaaatgtt acagtcctta

8521 ttactctcac gtaaccaggt tcaaatgtgc ttaaattcta ccctcaaaat gatttactta

8581 tttatcatgc ttgtcagact ttattattgc aaatatcaag ataacaatag aatgtgtcat

8641 aaagtttatg atactttgcc cttttataac tacccacatt attataaata ccatagagat

8701 atgtcaatat ttttaaaatc aataaaaatt ggccggcccg cccggtgaag taccacaatc

8761 acacaagaca ggcgtcaaat ggaagctatt acgtgttgtg tcacaagtgt ggtgttggag

8821 gcctaattta agtccacttt ctcttcctat aatttcctta tacagaaatt attggaaggg

8881 gaagcgaatt ggtggggagg aatatacata ggaagaggta ttgtcctctt gctgtgcatc

8941 tcctcctccg tagaataagg gtaggcaatg catctccaat agcagatgtc tatgggcagg

9001 ggtggatttg gatttttcag cgaatcccga tggccacttg ctcttgccgc tttgtattat

9061 aaaaaaaaac atctgtagtg tttcagcatg aatgctctct gtgtaacatt acaccatgta

9121 acatcagtac acagttagag ggttggtcac aattgtagta ttgtacttta caacatacat

9181 tatttgaatt gaattgattc atacatttat caatggatgt aacttgaatg aactatatga

9241 tttatctatc atgactcaat ttgtttatca ataatatgat acagtcaaac ttggataagc

9301 gagaatcatt ctttggaccc attgagctac ctccataagc gaggaactcg gagacacata

9361 ttgcggtccc ttggactcgc ttatgtaggt tcgattgtat tttataaggt acaaaaaaaa

9421 gatgtgtatg tattactttt ttttaacaaa ggaaaatgct ttaccttgcc ttatctttaa

9481 gagataaata taagttatgt tgggagtgtc acctgcccca ttactgtgga tttcaaatgt

9541 gaagtacaaa cacaattgtt ttttctctgt agtcaagggg tagcaaaatt cttttaacat

9601 atgttgcagt gcagtttcat aataaagcta tttgaagatg gaaataaact tgcacaattt

9661 atcttttaaa ttattcaaac tttctttaga catctttata aactaaaatt atgcggctaa

9721 aaattcaggc gctgtttcgg tgacggtccc gactagtttt caaccctcgg gggctcttcc

9781 taagggctca gagggacttc tgtcgcggcg ggtgtgagag tgtgagacgg gttccactca

9841 ccccggaaaa gagcccccga aggttcgaaa ctagtcggta gtgtcattaa agcaacgtgt

9901 gagtttttag ctgcttactt ttagtcaatt tatctttatt ttatgactta ctttatcgta

9961 ggatgccagg gtttgggtgg agcatcccgt caaagtatgg gaaggggcca ccgtgacttc

10021 ggattatcgt tccggagtgc tcattgtaaa gatcgatcag actggagaga tacgacagat

10081 taaggtaatc aatacatact ttgtgaaata gtttgagaac atttattaac atttttacac

10141 tcactgctat gtctgtcaag tatcattcta aaacaaactt tattatgaag atatagaata

10201 aatttatatt attgcaaatc tcaacaattt gttataaatt atatttgtag taaaaaatat

10261 attaattgat atctctttcc acagattaaa gatgaaaatc aaatgccacc tctacggaac

10321 ccttcactat tgataggaga aaatgacctc acatccctgt catatctaca tgaacctgct

10381 gtactgcaca atcttaaagt caggtaagta aacaaatctg ctacaaatct ccattgccaa

10441 tacacttgga taaaaatgtc ttatctttct cattctctat aaaagaacag agacaacgat

10501 atattttata caagttgctc ttctaagaaa ttgcagtttt gatttacaac tctatgcatt

10561 agagccgtta gtcctttttt ggttaaaact gatatagacg tggatgaaaa catttagtcc

10621 ctttgtacac agcgcaatta aaatcgaatt tactcttcaa gtggctcatg taatacaatc

10681 tcgtatgtcg gcgatcgctt agacactaaa atagatgatt tgcgttgcct tgcgtactaa

10741 ggactctact tacaagattc agaagagatg taactcttca ttgaatgggt gacgtcacat

10801 gttataggac atgtgacgtc attaaaaagt gattttgcgt aacttaaccg cggccacatg

10861 tcatcgcctt aactaatcct gtctaatgcg agtccaatga gcagcaactc atgaaggttt

10921 agttatttat atacgtttct tccattggta tcgattatgt ataagacgta aggtaagtat

10981 aacacgtgtt gtaacgattt attacattac aattgtctct ttcacactcg tggaatttta

11041 ttattgtgat atgcgacgaa tggttctgcc caaatcataa agccgagcgc ccatcgctaa

11101 agagtaatga gcgagagatg atgagctgag cggcctcaaa tgtgcttgcg tccccaccaa

11161 caaagagctg agctaagcga gcccaacggt acaacgcagc tgtgtcattt gacacagctc

11221 ggctcttttt gacacatctc atttcacttt atcggtggaa acacaagtat gcagtttcgt

11281 atgaaagtgg aaaatggggc cgcccagctc ccgctaggct ctttactttc gcagtgggcg

11341 ctcaccttta agctctgact acgacatata tattttcatc gtacatataa gctactagca

11401 gttgcccgcg acttcgtccg cgtggtgttt aatattaacc atgaatctta tctnnnnnnn

11461 nnnnnnnnnn nnnnnnnnnn nnnnnnnnnn nnnnnnnnnn nnnnnnnnnn nnnnnnnnnn

11521 nnnnnnnnnn nnnnnnnnnn nnnnnnnnnn nnngtaacca cggctctttg gggccatgaa

11581 acaatgtaac cagccgcata accacggctc tttgggccat gaaacaatgt aacctgggct

11641 ctttttggga ccacgaagca atgtaaccac ggttcttttt gaaccataaa acaatgtaat

11701 catggctctt tttggtgcaa tgaaacaata taaccacggc tctttagtgt catgaaacaa

11761 tgtaaccacg gctctttttg gtgtcacgaa acaatgtaac cacggctttt ttgaaccatg

11821 aaacaatgta atcatggtta tttttggtac cttgaaacaa tgcaaccccg gctctttgag

11881 gttataaaac aatgtaacca cgactctttt tggtacaatg aaacaatgta accacggctc

11941 tttttgaacc atgaaacaat gtaatcatgg ctctttttgg tgccatgaaa caatgtaaac

12001 acaggtctcc ttagacccat gtaacaatgt aaccacggat cttcggagcc atgaaacaat

12061 gtaaccataa ctcttttcag agccattaag taataaaact acggttctaa caattgtcat

12121 acaatgtaac cactgctctc ttatagagcc ataaaaaatg taatcaaacc tatacataca

12181 taatacattt gcacaccaga aatagtgata tttcttttaa aaaattgaaa tgtatctata

12241 ctctaatgtc tttctgtttt aggttactaa gtgatgtgag tcaagaaccg aagaataaga

12301 gtttggagta tgctcaaaat gagatcatac gactcaccac tgagtatctt gctctacagg

12361 tataaataga aagcctaata ccactagtcg ttgctttccg cttcgttcgc agtcatttta

12421 ttatttccca gtgagattga ctcctaatcc tgtttacgtg ttttcttttt tttttccttt

12481 ttaaagttat gttgtgttgt tagcaaaagg tatggctttt aaatgttata gttgttattt

12541 gatgctctat taatcagtaa cttaaccgct tcattagacc gatgtcacgc aaaaaaccgc

12601 cttgcgcaga agcatttttt atcgcgaact cggtctaatt aagccataag gtaatggaga

12661 aaaatttata gtatttgtta tgtatttctt aggaacgtgt tgacaagtta tctgagagtt

12721 gtcgtcggta caagaatcag attcgtttgt tggcaaatcg acttaaagag gctggcattg

12781 aagatgttaa tgacatatta gaagggaatg gtatgtattt gtatattgcg tactgaccga

12841 agttacgcga tgtaatgtta catgctacga tacgtcatga gcatacattt acatactgac

12901 tgcatgaatg tttgtcatgt aacatgttac ctatcgcgta acatcgtccc gcgtaacatt

12961 tataagaaaa catgaaatac aaaatcgaac atcttcattt gttacaattt gaatcaattt

13021 ctgaggggtt acccaagtac attcgctata aagcgaattt atagcgaacg tcgaaaaata

13081 tatgaaactg atattgagat tcgataaaat catatgattg tcacgtgtga tatcgaacat

13141 caatatcagt ttcatagatt tatgacgttc gttatagatt cgcttcgtag cgaatgtaac

13201 ttgggtaatc ccactcaaag aataaccatc caaaacatat ggttctgatg ggaagtttaa

13261 tatattcttt gtttccagtg actcttgtag acacatcagt ggcccagcag tcgatgtcta

13321 taatagaaat ggcgccggta actcgtaaga aggaaagaga gtatctcggc atgttcgaat

13381 ataagataca agatgaacct actattatta agaaacttat tattggtaag acattattta

13441 gtgtagtaat taaaagcacc gatagcctaa tgcttggagg gacggggtgc cgatctagcg

13501 gtcgtgggtt cgaatcccga catgaattgt cgtaaaccat atgataatac aaggtttttc

13561 aagcttacct agaatggtta aaaggaatat tagtaaatta acaaaaaact attaatattt

13621 acaaaaaaaa ccgagaattt aaaaagtttt ccgtcatttt gtgacagcag ccatcttgga

13681 tttacaagtg tccaatttat tatgattttt taaatagatt cgtcgactgt tttttttgtc

13741 tcagtagaga caaaaacatc tagtaagatg ttgaacgcga ttccgtctgc gtaacactaa

13801 aaaagtaaca actaataaca tagttaacac cgcctatgtg ttcttccaac aaacaaagtt

13861 tccaacagac aaagttcaac atctgtgtga aatttcatgg agacccgtta agccgtttcg

13921 cagatacgtt caatccaaca tccatccatg ttaacctaat tagtaaaatt tccatagatc

13981 taaagccgcg tgtcgcagta acccttttgc cgggtctacc ggcatacatc ttgttcatga

14041 tgctgcgcca catggaccac gtggacgacg agcccaagat gcaccagctc atgaaggctg

14101 tccgcactgg agtcaagaag acattgaaga aacggaccga cagtgtagag tataatgcac

14161 tctggctgtc taacatgttg aggtatggct tctctattag attagaaact ctatggtatt

14221 tattttaatg ttttatgagt gttaaaagat tgccacgaag ttcattgatt tattttataa

14281 aactaaaaag ttattagaaa tatggaactt ctgtaataac caatttgagt acacttaagg

14341 tctgctcgag ggatcaagta acgtctttgt gtgaagcaag ttgagcttct ttgaaattac

14401 tgttatgctt tggctttatt acaaaatgcc ttgagaaggc agacatgaaa aaattttctg

14461 gaccaagccc acccgagact gtaatgtctc agatgatgat gccttgactt ccttagccct

14521 cgttcacgat aaaaacggag atttattttt aacttaactc cgtttttatc ttgaactcgg

14581 tcaaatgact ccgaaatatt agggccaaat ttttaccgat catttttaga cccagaaata

14641 gcttgaaagt cttcaaagtt tgcttgtttc tcaggttatt gaacaacttg cgtcagtaca

14701 gcggtgacgc gatctaccag ggctcgaaca cgccgcgcca gaaccagcag tgccttcgca

14761 tcttcgacct ctccgagtac cggcaggtgc tcagcgacat cgccgtatgg atctaccaag

14821 gtattgatat gctaagtgta gcatgtgaat tatttccctt cttatgcata tcatctttca

14881 taaactaccc tccaccttta tagccatcca catataacac tttgcctgca attaaatata

14941 cataacgaaa tgttgcgatt ttaaaattga tttgctataa gcggatagcc ccgaatgggt

15001 tcgtgtttgt cagttgtcgg tcgtgtgagc tgtcaattgt cagagaaggt ttatatgtag

15061 aactagcgac ccgtatcagc ctcgcacggt tgtaatgctg atactaaata cactacagaa

15121 aactgtgaac gttcttttac gctataaagt gatgtgatgt ggtgtattta gtctcagcat

15181 tgcacccgac ccaggtcgct agtactatat aaaataatat gggaaatctc ctttaacttg

15241 ttaagcgctg gtacatctgt tggagcgtca actggagcgc ctgatcctgc ccgctatcct

15301 ggagcacgag gagatcaacg tgcactcgcg gccgtcgcgc gccgccgccg ccggcacgcc

15361 gcccggcccc gcgcgcctca tacacgagct cgccgccgtc agggaccatc tgcgcacctt

15421 caatgttgac actccactca cacttacgat atttaaacag gtaagaacga gaaagaacta

15481 aaaattatat atttgcaaat gtaatgtgtt tattcgggat tgggggcgtg gcttagaaag

15541 ttgaaggcgg gccacaaaga ggcggagttt taaggtcata aaattaataa aatactagct

15601 gtggtttgcg gctttgtaag cgtaattaag gctatatttt gcctatgtta ctccgtgata

15661 atgtagcttt ctaacggtaa aagcattttt gaaatcggcc cagttattga tttattccaa

15721 tacatacaaa aatacaaagc ttctctcttt ataatattaa gtatagatat tttctcttgc

15781 agttgttcta ctacatctgc gcgtacagct tgaaccagct gctcctgcgc aaggacctgt

15841 gctgctgggc caagggcctg cagatcagat acaatatatc gcacctcgag atatggatca

15901 aagaacagct cgctgaatac ggacagaaga gtgtaagtat aattttattt gtaaaaaatc

15961 ctggaaaaag aagtaaagga aataaaaaaa aaagaaaatt aagaaaaagg actataggaa

16021 agttactaaa ttaacagatc gattttattc ctgaagctat gtatgaaatt taaaaaatat

16081 tcatctaccc tctttaactg cacttctaaa atttactaag aaagtagttc gactcaggat

16141 gggctccatt ttcctttact ccacgacgag cgcgtatttt gcgtgacttc acacatatct

16201 ttgaatttct tctccgatat gtagtaatgt ttatcttgcc accaggtgga agagatcctg

16261 ggggtgctga agccgatcac gcaggcggtg cagctgctgc aggcgcgcaa gtccatgagc

16321 gacgtcgaca gcaccgtcga catgtgcgcc gacctcaccg ccatgcaagt ctgcaaggta

16381 cgtataaccg tacggcttca ttagaccgtt gtcacgcaaa aaacgacctt acgccgaagc

16441 aaaatgtccg ttttaaatgg aaactcggtc tgttgaagct ttcgggtaag ggacggtccc

16501 tcaaatatat actaaataat gttgttaata ataaatgttc gcgtcttctt ccaggtaaaa

16561 tttaaaaata tagcctatgt tactcgggga taatgtnnnn nnnnnnnnnn nnnnnnnnnn

16621 nnnnnnnnnn nnnnnnnnnn nnnnnnccca gtagttcttg agttatttct atacatacaa

16681 aaataaaaat attttatctt taataaaaac atataataat gtatctatac tatattataa

16741 agagagaaac gttgtatttt tgtatgtatt gaaataactc aaactcggta gttacaaaaa

16801 ttcttgcatc attggaaagc taatccccga gtgacatcgg ctatattttt taattccacg

16861 cggacgaagt cgcgaacaac aactagtatt aatatagaag tacggttttt tattttaaat

16921 tatttatttc aaagatcctg aacatgtata ctcccgctga ggagtacgag gtgaaggtga

16981 cgcgggactt catccacgag atacagaaga agatgcagga acgagctgga cctaagacca

17041 ccaaggaacc ggttagtatt agcttagaaa ctaggaaata atagaaaatt ctagtataat

17101 caaaactaat ttcacatatt ttttttggtt ctattattgt tatgctatag cttgattaag

17161 taaagtatgg tccttcgagt cgggcatttc gttttcctaa aagaatttgt ttatggtcgt

17221 tcgagttgga catttgtttt cctaaaataa tttgattatg ggtccttcga gcaggacatt

17281 tatttttttc taaagtaatt tgattatggt ccttctagca ggacatttgt tttcctaaaa

17341 gataactttg attatggtcc ttcgcgtcgg acatttattt tcgttaaata ttttgattat

17401 ggtccttcgc gtcggacatt tattttccta taacattttg attatggtcc tttgagcaga

17461 ttttttttta tatttcaaaa atcaacatat taactaaatt tctatacttg cagcaaaact

17521 tactaatgga tacaaagatg atatactcag tccaatttcc gttcaacccg tctccgatca

17581 gactggagga catcgaggtg cctcaagtgc tcgagttgga cggactgctc actaagatat

17641 aaatatctat tattctgtag acctactaat gttcatttta tctatttaat gttactttaa

17701 actagctgtt gttcgctact ttgtctgtgt cgaataaaaa tatagcttat gttactcggg

17761 gataatgtag ctttctatga gtgagcgaat tttttaaatc ggtctagtag tttttgagtt

17821 atttcaatac gtaaaaaata caaagtttcc tctttattta tggatgaatt cgaaagttat

17881 ttcttttaat ttagtcaaat atattagaat tataatattc agtggatagt tgcaaaatat

17941 tttaacttat ttaataatgt tgtttttact gtatttattt ttatttcaaa attatatagt

18001 atttatcact taaatatttc tctatattta taataaacaa aatcttatcg ttttcttaat

18061 tctgctgaaa gtcttcacag tttttttcat acttctaatt tagtgctttt ataatattag

18121 taactaactt actagggtga cataacgacc cgaatgtctt ggcctccgac aagaaatctc

18181 tcccccaatc gcgttcggag ctcccttaat ataacattag taggtctaca tatgttatat

18241 attatataaa aagtacttat tataatgtaa tcttgccctc agcatttttt atacataata

18301 attttataat tccttttcgt ttgccataca ttatagtgtg ccattattta tttgattgta

18361 caaatttgtt aacaatagtg ttttgttatt tgttggtatc cagtgagaat gttttatgaa

18421 gtacaaaatg tcataataac atggagatta atatatacta ctagagaaaa aaataataat

18481 tataatttga atgagttggc acgtgtaata cacgagtact cttcctgtgg acgtgccaac

18541 tgacaactga tttcgttacg gtttatgaat tttttatcat cgcgtacttc ttgtaaaggg

18601 gtaggcaggg cataatataa tactcttact gattagtcca ctgtatcttc aataaaatgt

18661 tcatttttct tactttttcc ttatacaaaa taaactaaac atttttattt tcattacaat

18721 taaaaaataa ataaattatt actttagtag cgatttcaaa acaactccat aataatattt

18781 aattaaaaaa tacccactca aagtcactat agaaaaaata ataaaaaagt ctattcaatt

18841 ctatgatatt tgatgtactc agggcagttt aatggtcact ttaacattcc cttagtgtag

18901 atttctaaaa ctgactttgg agacttctct gagtgtgaaa attaattaag ctctcactgt

18961 tattaattaa gtaaatgaat atatattata aaatatatat ctttatatat tggattttaa

19021 ttaataagaa aaacatattt gaaatgaaat aaactgatta gcttcaaagc tttattaaaa

19081 acatattcat agaaatatta gatattaagt gttgtactta atatagacaa atggcaaaaa

19141 tatataatgc ccagttaaca ttagtacatt ccagcgctgg attgcaactg gaaacacctg

19201 ttttcacgct gaatttatcc acagtctttt ttatgccagt ttgcatcctg tgctggaata

19261 atgtaaaaca gtaatctgat aaaatagaca tagtaacaat ggatatctta gtcaaatatt

19321 tataatttaa tttgttatgt ttacattatc cgaaacattt gtatgaaaaa tattgttatc

19381 tttgtctttt taattgaaat gaaagagatg gcagtagaat gtttcggcag tgtaaacgcg

19441 gtgtaatagt tttatttttt aaacaatctg caaacttatt ataatctcta tgatttaata

19501 tcagtattca tattttattt ttaaagaaac ctaagatctt atatccagtt tacattattc

19561 caatccagta aacctggaaa ccaaacagtg cattccaatg tccagcgctg gactgaaata

19621 gtgtgaactg ggtattatat ctgcttatgt tagacaagta tgtgttattg ctgtctctgt

19681 tctttttaaa ggcaatgaga gggacagcga tattataata caattgttaa tttcctatat

19741 atctagttat tcctgtgatt aatgtagcta taaatagctc attatataaa tggtgttata

19801 tagtctaacc tggataagcg agaattctct aaaagtgaga aaaatatcgc gaacctattc

19861 tcgcttatcg aaggttcgac tttactttgt ttttatatac aagtgttacc attcaggtgc

19921 ttttaaagcg tcataaaaat atattaaaat gtattcaaaa tatatatctt gtcataaaag

19981 tacttaattt ttatatatat tgttccttta gtatcaatta tgtttagtcc tagtatacgg

20041 ctgcagtgac ttgtattgtt ctctctgtct tttcaatgat aatgaaagag acgacaatat

20101 aaaccacatt agaattttac tatactaatg ataaattgtt attttatttg ttattttgcg

20161 tccaatcata ttttttagat tttaacaata attttatcta tttagggtct acattaaatg

20221 aaaaaatgtc aataaatttt atattaaaag aaaatatagc aaaatatata gcgcgttgaa

20281 aacttggtac taaatattat aatcaattgt aaagtaggtg ctcatgctct ttgttaatat

20341 atcaaattat tgaaaataaa taaaaaaaac tatatatcta tcgaaaaatc attattatgt

20401 cttttagagt acagttaata aagttataaa aaatattatt tcctaaaaat aaaaaaatat

20461 accaaaagct tctttgaggt gcatttaagc gtatttatga tttgactcca agcattacaa

20521 aaaaaataaa accgagcttt tgagttggca cgtcgcatac aagactaatg ttatgtttca

20581 agtgccaact ctagtgctac agcaaatttt ttatctataa tatttctttt ttacattcca

20641 ttcttatgca tttatagaga aaatatagat agataactac acatttttgt agtataaaaa

20701 cacataataa tttaaagaaa aaaccattat taattgatag acttttaatt atcatgattt

20761 tattttttat tttaaacata ttaagtaaaa aaaatgtaaa caaaaaggca ttagatatat

20821 taaaaatata tattatgcta tttatatttg ttattgggat atttttccgc ttagatattt

20881 taaattattt tattaattat cgtggttcaa agatttctat ggacatatat ttttccttat

20941 tagaaaatta ctaatgattt tttccttcct tttgtccttt atgtgtatcg aatggtttta

21001 ttgtatttta cattgttaat tcatatcgtt gaaatgttat acttttcaaa atatatgtta

21061 tatgaaaaaa aatctacgtt tttttgaaat tatatatcaa aaatattata gaagcaataa

21121 ttttgcatat cttttgattg ttaataatta ataattattc cataatttat gacattccga

21181 gttgttttaa tacattaagt agaaaatcaa atatataaaa gaaatactgt gttagttcca

21241 tagatatgtc attatttcgt ttatttatta ggcaatatgt tagataattt actatttcta

21301 tgtatttata attttttgat acttattgac ttaaacatca ttcatcgaag tagatttgaa

21361 atttgtagca taatttattt agtattattt taccgaaata gttttaagag aaatgatttt

21421 aaaataattg tattaataat atatcccttc catttcttat gagggtaggc aaatattcac

21481 tcgtcagatt aatgttgttt gaaaaatgag ggtatttgca ataaatatca tacctcatgc

21541 caccgtttcg tcgtgttttt gttgaaatta aatgtttgta atgaaatcaa ataaatatag

21601 cagttgacct tattccacgt tttattttac caaatgtgtt taattaattt tagtttaaaa

21661 gcctgttttt tctcgaggta cccgcatctt gctaactgta aaaaacaaaa taagtaagta

21721 tttgatattt cgttctatca aaagctgtta ttttttacaa aaatactggt gcaatttttt

21781 cttaaattgt cgcaatgtac ttaatttaat gatcctcatt ttacttaatt tatcctttat

21841 aatgttggat gctttaaaac ttagtaaaaa ataatgagac aggaaaatga caaaaactgt

21901 gtttcacgtt ttaatattgg atgcaggttt aaaatacgaa taacttttaa gtaaaagata

21961 tgatattcgc tacgtggtaa taaggttgtc aaacaacctt cgatcaaaac ccagtattcc

22021 gcgccaagcg tcgcagaaca gacgttacta attcgcgcct ttttacaacg tgacgtgtaa

22081 cattttattt ctataaataa ataaaaaaat actgtgatgt aaaacgtaga gttaaaaagg

22141 tgagaaaatt cattttaatt attattgctt ttttaaataa ttttctaagg aattaagtta

22201 acaaaatatt aataaactac ctaaatcgaa cctgggggtg ttaccccaag tacttgcggt

22261 acaaatcgat tcaataaaga acatcaaaaa tgtatgtaac tgacaacgac gttcgatatc

22321 acacgtgaca agcatatgac ttatcggaaa tcattgtcag tttcgtacat ttttgtcgat

22381 ttcatcagcg atatcgccaa tcgaaagata ctttgggtaa tccccctgaa tcgttaaaaa

22441 cgaagtgagc agcaagtccg gtccaattat gttacagtga aagtgatgtt tcggtctcta

22501 ccggtaccgc tcgtattcct gtctgacaat aacatgcatg accttatata aagagaaaaa

22561 ctttgtattt ttatatgcat taaaataatt caaaaactac tgagccgatt tcaaaaattc

22621 tnnnnnnnnn nnnnnnnnnn nnnnnnnnnn nnnnnnnnnn nnnnnnnnnn nataccacgc

22681 ggacaaagtc gcgcacaaca gctagtattt gataaaataa catttgttta gtaaaacgta

22741 tacattaaat tatacatatt atgtattaaa agaaacaaac tatgttatct gcaggaaaag

22801 gcgataaaga caggtttgta tttattaatt aaatcagaca gattataata aaaccttttt

22861 cgttttgttc ctgtccacat gtaactatta gtagcttgtt ttaaattttt atcacttgaa

22921 tatcttggat tttttgaatc gcaaagacgc aatacttact aggaaaataa caaagataac

22981 aaagcactga aaattaatta tttaccaatc atctatacca atattataaa gactctttgt

23041 atatttgtat gtattgaaag aactcaaaaa ctactgagcc gatttcaann nnnnnnnnnn

23101 nnnnnnnnnn nnnnnnnnnn nnnnnnnnnn nnnnnnnnga aagctacatt atccccgagt

23161 aacattttta attccgcgca gacgaagtcg cgagcaacag ttaataaaat tataaaataa

23221 cagagcacta taaaaatgag ttaacgattt ttcgaattag tttcttacta acttaagtaa

23281 gtataatacg tacatacaca atatatatgg caaacggctt acatttcaac ttatgcaaat

23341 attgttttat taagactaaa taacacgcct tcatacgcca ctgaaacaaa acactttaag

23401 ctaatattaa tttgtacaca aaactgggat ataaaataaa gagtgatgta cacggacagt

23461 cttaccacgt ccgcataatc cgtgtacagc cttaagtaaa atacttcggc ttagaaacat

23521 tatttcataa gacactaata ttataatatc atcataatta ctaatcgata aattatcgga

23581 tagaaaaaac tggcttaatt caggatttgc ctttcatata tattggtcgt aaacactaaa

23641 taaaatacca tctagacaag cactaataag tggtaccaaa tacactatta ccaaactata

23701 caagtggcgg cccttggata gtttggattt atcccctcga aatctgacta cagtatagtc

23761 attacagcga ttgtacaatt gacatggtcg gttcaatgtt tgtcctccct caacccctag

23821 accccctgga gtaattgtaa tattattaat tgcaaaaaca aataatgatt aaaaaaaaaa

23881 gttaggagat aaaataaata acataaaagg arctaacgtt aaagatacca aagatgctgc

23941 attagcaata gcagcaggaa cgacagaaaa aagaaaaaaa ataaaaccct agaccccaaa

24001 gtctggataa atgcttgcat ccaatttcta gaataatcta aatgaaaatg attactgaca

24061 tctgtcatca tatgaatatg tttactacta aaatgtcgca acacaaacaa ttggtgataa

24121 ataaataaat attgcatgtt taacgatact aaacataata ctgatagact agtcatatta

24181 aaacattaac aaatcaacat actgattgga gtttgatata cttaatgact tatgacattt

24241 tgtataacag aaaacataaa tgctttgaat agtttctgag aaggaattag taaacgcttt

24301 tttcactaac cctacggcac cattagacag agttcgagga aaaaatgtaa actttgcttc

24361 ggcgtaaggc cgttttttgc gtgacattgg tctaatgaag ccctatgtta ataggtacca

24421 taagtgttgt tcaaaatgga tttccatttg gcacacacct ttcgcctctt taaatcttat

24481 tactatttgg ttaacacata taaagattgt accagttaga tcactttata acattgagat

24541 gataagccaa cctaaagtca acggcgtgga gttattgttt atttgtctta aaaacgagaa

24601 gtttaatgtc aaggttgatc atgtttggtt ttttttatta aaaaatggtg cgtatctaaa

24661 gcggcatctt tgtatttatt gagccaattt aagaattaat tgtttcgtgg taataaatta

24721 tgtctacgta tgtcagcaat taataattcg gctaacgacg atattatcgt ttctaagtat

24781 tgagtgctaa attactctaa gttttgaatc gacaactttt tgtacctttt ccaattaaag

24841 ttcgcgctag aatatggttc acatcaatgg tatgttaacc cgcgaccgcg agatctgtag

24901 taacctgttt atgacgtttc gaaattttga taaaatagaa aatgttgtac ttaagtaagt

24961 aagtatttaa aggcaaaaag cagcaaattt tgatcgttat tttagcaaaa gtccttgtgg

25021 attatcatat tacgatccga taaaaaggta aattataata ctaatacgag ataagtcgag

25081 taattatgtt tatctttaca tactacataa tgataaacat tgtatataga gaatattaga

25141 atacatctct tctagttgca gattgttgtt aaaatacacc atagcaccat aatgttttgt

25201 taaactctat taaatatttt gtaaagtata acgtatacta ttcataatag aaagctttga

25261 tttaaatata catatacatt caacggaaat atttttttat tatattatat tttttcccac

25321 aactccctac acacggaatt aaataattct actgaagtta tttgattaac atggttaggg

25381 aaaactaact tgtcttgcaa gtgttatagt taacctcata aaattgttat gtaacctttt

25441 tacttgaaat aagttaacca aaagaagttc gtttccgaaa aaaggcgaag gtaagtaagt

25501 aattaaaact tctagaagta agtactcaag taatgtatac tttgtgatcc tgaacgttaa

25561 gacgagtaaa taaatctttc ttatcacaca atatcttatc ataacgtcga atttagtaag

25621 ttcagtacta aattcaacat atcggcggac tgtcgtttgc ttctcgctta atatatttgc

25681 gaatgttttg gcgcttgtgt ttgtgtttgt ttttaattat ttaattttaa tagaaaaata

25741 aattgtttcc tgcgattatt aagtcatggt aagttagtta atcattataa agattatttg

25801 attaaaagct atgaaaaata cagtgcagtt attgaaggtt tgaggaaaaa gaaatatttt

25861 ctattacgtt gactttaata ttattaagaa tcgtaagtag tatgttttgt taacagtacg

25921 atttttaata tttctattta ttttcttcaa taatctatac taatattata aatagagaaa

25981 ctttnnnnnn nnnnnnnnnn nnnnnnnnnn nnnnnnnnnn nnnnnnnnnn nnnnnnnnnn

26041 nnnnnnnnnn nnnnnnnnnn nnnnnnnnnn nnnnnnnnnn nnnnnnnnnn nnnnnnnnnn

26101 cgcggacgaa gtcgcgcaca acagctagta attaatattc gtgcagaaca aagaacatta

26161 atttgtttaa ataactcgaa atccagggca tacaagyawk nacrywgntm wtsgywacrk

26221 tggrmntttt tngcactttc atgcaanttc tttagcaatt taaccgttag tgataaaggt

26281 tattgcctta tgtttcgtgt taaatatttc cgctaatgaa aaatgttatt cgaatttaca

26341 tgttacgttt ctaaactaaa taaataaggt aaaggtacca gtaattgata ctatagtggc

26401 ggaaaagcgc tattttcttc gtcactttta atccattgct agtagaaact tttgtaaaag

26461 ttactaatat taaccagtcc ttcaagcaac ttataatata cctcaaatta ttttaattca

26521 tcttttaata atttttatga tttattttcc aaacctctga aaatgtagta gtaattgata

26581 ggtgtctttt tgtttgcagt agtaattgac aaggcaaaat cgatacagtg gcccagtaat

26641 tgacaaggcg attgctctta ttttaaccaa cttaatattg ttatattaag aaagattgaa

26701 gtccaataga tattatattt caatacataa taaacataag tatgcttgtg ataaaatatt

26761 atacaaaaca taaatttgtt caaaacaaat cttcatattg gtaaatataa tataggcaaa

26821 aaatgtttta tatagcggga gcgctccata gtattttgat agcatgcaac tagtaagggg

26881 tatcaattac tagaaaacga ccaattttgg ttctagtaat tgataatcgt tataactaaa

26941 taaataattc aaaaatcgat tagaaaatta gacaatagag agtacgtcta taacaaaatt

27001 tagttctggt aatatttttc gattcaaaag tgctggttcc atgctaaaca ctcgctaagc

27061 gatacgctct cttaacaaaa cacgtaagaa aagtgacaaa cgaggagcgc gcactgccgt

27121 ataatgcaat aatattaata aataagtatt atgaatagaa atcactgatt ttgattttaa

27181 taacaaatta caggtagttt tattataact ttatgtgtaa atagcatgaa taaaattaac

27241 agatatttta ttataagctg tcaaactcca aattatcaat tactgggtca ctatcaatta

27301 ctggtacctt taccttatac cttttgaatg aaaataattt taaataaaac acatgaaaca

27361 tgtaagttac aaatgcaaaa ataactcaat aagtaactac gtttagttta atttggacaa

27421 aaatgttaca gctatatctt tataacctaa tgatataatt taatcaagaa tagttaagat

27481 aatagttaat taatataaag gtatcaatat cgatagataa gtgatttttt tctgattact

27541 tatatcgcat tttatgacac ggtgataaaa tgtttgggga tttacaagtt aattgtcact

27601 aaaatatcgc tatatcaaat aaccataacc agttcacttt gattaataat tatcaattaa

27661 tattgtaaaa tgatttcaaa ccctagggct tcattagacc gaattcgcgg aaaaaaagga

27721 gattttgttt tgtcgtaagt ccgttatttg cgtgacatcc gtctaatgaa gcctaaagaa

27781 ggataaagta agtatgaaaa agtgttttaa ttatgggaga atctaatcta atattaaaat

27841 cagctaatta gcaaattttt atttataagc gcgcgacatc tagttcataa taatggtagt

27901 aatttatgat tatttaagta tcccaggctt caactatttg tgtttgcggt cgggaatgcg

27961 atttgtaaat ttacacaaac acaacaatag ggataaagtg agtgcacgcg gtatcttatc

28021 ataacagcaa ttacctactt tactatttgc aacggaaaat aaagaatatc ataggtagaa

28081 tatgattgtc aaaaaaaacc ttaaccttat tatagtctct catttgatgg ttgcttgcca

28141 atcgtttagt tagtaactaa gtaagtactt agttaaacga aataactcgc aagttaagta

28201 acccacaaag cggaatcatg tcaacatcac tgacgactaa tatttgcttg gtttgtttca

28261 gataaaatga tttgtgagca gtctataatc gagaaatcca ttacaaaacc gaccaggaag

28321 ctgttgaata tttaagagcc aaaaaaaaat ataaaaaatg aatacaaacg gaacaacaca

28381 aatgcgaaat aatggcgtaa gttaaagtac ttagttattt ttataacatt atttcaaaag

28441 gacaagacaa gacgaaattt aatatgttaa aaaaacaata aggaaaagaa acaaaatgtc

28501 aaaaaaaacg ttccaaaccg gttttcagag gggtatttgt caacaaagag gaatggtcca

28561 gtttcacact tggtgggacc agttacactc gttttagtat aaaaaaactt taaccatacc

28621 ttaggttata aaaattactg cattaggttt ttcaaactaa aataaagcat ttttttcata

28681 tttatagact ttcctcaaag gtcgagtgac cattgtcctt gnnnnnnnnn nnnnnnnnnn

28741 nnnnnnnnnn nnnnnnnnnn nnnnnnnnnn natagacttt cctcaaaggt cgagtgacca

28801 ttgtccttgc agattctaaa aattgatgta ttgcgtttat caaactaaaa taaagcattt

28861 ttttcatata tagactttcc tcaaaggtcg agtgaccatt gtccttgctt gtagttcgtg

28921 tttaagcagt gtaaacgcac acttacattg gtaaaagcaa ttcgcattca taaccagtac

28981 aaacagcgat aaggtttagt gttgtcgtta aacttgtaat acgcaacttc tagttacaaa

29041 gtcataatca aaatgatttt ttattactcg ttactgataa catctagtat taattcttgt

29101 tactagataa ctacagtcaa atctggataa agccttgttc gcaccaggat agtattttag

29161 tagtcattta tccgaacaaa ggatttacta ctgttcggac tcgtttaagc ctaaaactac

29221 gcactagggt tgtaatttag gaactaaacg tgtccgaaaa gcaacatttt agtagattag

29281 attagtatat cctttgttcg gatactagtt actttctagt cgatataaat actatcctgg

29341 tgcctacaag gcttaaacga aagtcattct gtgttcctga cgagctacct tcataagcga

29401 gataaacgga ttagagagaa aagtctataa acgacacaaa tatagcggtc ccttggactc

29461 actcacttaa ccaggttcga ctgtaattac atttttgttt agtttattaa tatgtagatt

29521 aacactagtt ttagcgtaag aagttaaaac actgtcacct tatcaattca ctaaaaggga

29581 agcttagaat tagtgcagtt gcactaggtt tttcacttac gaaacagcta tataaaaaca

29641 tatcaacaac ccgctctttc ttctagaaaa aactgcgata acaatatgtt tccacactgg

29701 ttacgacagt gaaaacatcg tcgtacattt taatatctgt ggcagaaaga caatgtgtca

29761 tttgctattg tttgtgatag aatgtacaac aaaatgtaca tcgtaaagtg caatcaacaa

29821 taaaaataaa ggtgttgata cattagcgat atagttaaat tacaatatta attatggcac

29881 acagaaacgt tcaatgatca ccaagagtct agcaaactag aagagaaaac ctagcttaaa

29941 gctgttttct tctttttgaa taaatttagt tcgacaaatc tacacaaagg ggcctagtga

30001 ccaattaaag actctgcgtg cgtcagtcaa cccttaaaga aactgttaaa gaaccgttta

30061 atgttcaaaa tacaatgatt caagatttta tttcgaaact tttaaaaggt gtgtaactta

30121 actaatgtag aatgtttaaa taatcgataa ttttttatca aatagataag atattggtaa

30181 tttaatttag gtagttaaaa tataaatggc gagtgcgtcc gcggccttat tgagttgttt

30241 actaacacag catagccgcg gtgtgcattc cgccgacgtc tgttactaac gtctgtagtt

30301 attggagtaa aatcgggtcg tagttaaaaa aacagttttt tttttaatat ttgaaatatt

30361 agttcgtgtt cgtaaatcag tcaaaattgt gttaaaggta aatattctcc agtaatccat

30421 ggtttgataa cttaggcatg aaaaagtttg atgtgtttta attatcaaac atgtccggtt

30481 gtgttttagg atttgtttgt gattgtacaa aattcaaatt gaaccaactg gatttacata

30541 aaaaattgaa gttattttac gaattttcaa ttattttata caataaaatc taaaattaac

30601 atttttatag aaaattaata aataatttcg tttatagaca tcagatggag ccttcgtcga

30661 aatggacgaa gtgcagaaaa aagacgcgga cccaaatgga gaggtaagtc ttataataac

30721 ataaaaaata tcataataaa taataagtaa gtataacata taaaaaaata tgtaaatata

30781 atatcaggta ggtatttaat attttttggc ttgtaaatca atttctaaga acatttaata

30841 ttaagaaaat ttaagttatt tcactgttta aacttccgtg agacattatt attgataaat

30901 aaaaacggct gcactcacgt tattaagatc cggtaacggt accgactagt ttcgagcctg

30961 tcgaaggctc tcgaggaata tttaagttgt ctaatgagca gtgtcaatat tgagaattat

31021 gtgactttag tttaaaagac taaagtttaa gagtaaaaga agagaggaat atactaaaca

31081 caatagagaa tagaagagta gagtaagagc atctgatacg acacgacgac ctgataatcg

31141 aaattataga aggcaaaatt gaaggaaaga gaaagagaga aaaaccgagg ggaaattata

31201 cagatcaaat aaaaaagaaa gtggacgtcg tgtcgtatca gaagaccaag atcttggcac

31261 aggaaagaga gggttggaaa gaactccacc gacaaggtca aaaaccttaa attaaagaaa

31321 aagaagtttt aaaaactata agtattaata aagttgtatt agtctcctca tgtgtattaa

31381 ttcagcatct gaagtatttt ttcttaggct gtttttggtt tatattaatg tcctcggctg

31441 ccgcacatag cttctgtttg ttgccacatt tagactactc gtataagtag tacatttcta

31501 aaagcgtatt gaataaacaa acgattcatg agacaaaagg tcttatactt tgacacaatt

31561 ccgtaacaca gttgtcctta tagccttttt agtacaataa caagctaatt ctgacacaat

31621 tgaacactaa tagatcggga tccgaaaagc agcagacctc agttatatcc aaatacnnnn

31681 nnnnnnnnnn nnnnnnnnnn nnnnnaattt atatacgctt agataggact cgtatgtact

31741 atcagtgaat ggacattagt tatcctgtcc agttgcgcag tttcgcacaa tcacgcgcta

31801 aagttgcagt aaatattact aaagtttact tacattcaca aagctgattt tcacatannn

31861 nnnnnnnnnn nnnnnnnnna agcaaacggc ctaccagaca ctttaaatgt ccacgcggnn

31921 nnnnnnnnnn nnnnnnnnnn nnnnnnnnnn nnnnnnnctc atagcgctaa tgcttataaa

31981 gtcaacttaa cttatttttc accgcttgat tttcatatac aatgctcaag gtctcctagt

32041 catcctagtc tcctagtcat agtcttatgg taataccccc tgagagacag tagtctcttc

32101 cttcctttcc ttttgcagaa aagaatgtgt tacacaggat cctatagata tttaataaat

32161 tataaaacaa agcaattgac taattagtaa tttaaagaga gattaattaa aattgattaa

32221 tcaaaatatg tcaaggagaa tattgaagtg tatattttac tttctaatta ttctcagttc

32281 gtttgtattt ataacaaggt gacagtcatt gccgaaaata attcgttcaa tacctacttc

32341 ctagttattc taaaatagat tgtcatgtga tttttattat ttattgagta cttacatttt

32401 tcttgaggac aaagaaaaat agaaaacaac cctaattgta gaggtattgt aatttttgtt

32461 cgttatttct ggactttatc taagtatatt atgaataact ttattatcat ttggaaatta

32521 agtaggtaaa taattatgta tcttataaca atagttaact atacatttca tatagtttgg

32581 ggacaatttt ggtgtaatta agtacatttt tgcaggaaag tatatttaat ataaaagtct

32641 ccaataagtg acgtttaaaa tttgtttttt tttgttcaga tagtccacga gaagctggag

32701 tatgagccga gcggctggtt cgagctgaca ctgaccaaag ccggcagctc cactgaggac

32761 ttcctcaaga ataacagatc cgctatgaag actgtcgccg tctttgtgct gaatggtctc

32821 gttgttggct tctttttcgg atgcctatat tactggatga atcatagtaa gtgttcaaac

32881 tttgtaaaat tctatttaga agctgctaag acgacgttta tttcctactc aactcggtga

32941 aatatgattt gagattctcg ttgcaaataa taaagtcttt gggctctatt ttccgcaact

33001 ccacgactac cgcgtatttg gcgtgacaca catatctttg aatttcttcg ccgatatgtg

33061 caggttgcat cgcgatgttt ccttcaccgt aatagtgttg ggtaaatatg tacatatgaa

33121 aatcgaaaaa cacgttggta catggcgtat ttcgaactcg ggtcctgcag tttacaagtc

33181 aaaaaccaaa cccctgatcc accgacgctc ttatagtttg ttttacgaca ttatttaagt

33241 taagtagtta cacttatcca gaaatcttat tcattaaccg attgaaagca agttaatgct

33301 tttgtctctt gtttcgcagg caataagcca ttagaattat gccatgggtt tggaagtctg

33361 atagccttcc tcagtatagt ttacttcttc gtgatttact tcctcgtggt gaagcggtac

33421 ttcggggcgt ggttcgagaa gacaatatgg acgagagtcg agacgatagc taatttttgc

33481 tggaaaatga tgtaagtgtt aacaacattt ttttttcaaa tagtagaaac agccatacaa

33541 taaaaaaatg tttattactt ttattataac aactatagtc aattgtttca tcttttctgt

33601 tcaatcctgt ggtctttatt tcccgttctg taaaacggat gaagttctta actcttctat

33661 atcttctttg cttattttgt atgcagatgg ttccgctggt gtttctcagt gtgcgtgctg

33721 gccgcgatag cagtcttcct ttacgttgac accagagacg ctcctgaaag actgatctca

33781 cttcttggtc tcatcgttct attactttta ggttagtaga ctgtttcgat tcaatctttt

33841 attaattaac tgcatacatc ttaagttgtt atgaaattaa tatatttgta ataaataaat

33901 gtacattgtt ctaggttttg tattctctgc ccaccctggg cgcataaaaa agcgcacagt

33961 gagcatgggc ctgttgatac agtttatatt cggtctaatc ttcatcaggt gggatgctgg

34021 caggctcgcg ctgcagtgct tctcagataa ggtataactt ttcgaacgca agtctacgat

34081 tttagtctct gagtgactta cgtaattgtt tcatgtcgat atcgcgtatc tgtgtatgaa

34141 tcactactga gtaagccact taagccttgt tcgcaccagg ttaatatttt agttgagtag

34201 caagtatccg aaaaaagaat gtatcccatc tactctacta aaatggtgct gttcgaacac

34261 tagttagtta ctaaattact tgctactcca ataaagtact atcctggtgc gcacaaggca

34321 ttagagactg aagtccctga tttgcgaagc tttgtatgcg caggagctaa ggaccatcat

34381 acacgaacag agtcattgtc gctatcgtcc gaatgtcaac tattttgttt aattctccag

34441 gtggccactt tcctgtctta cggtgtagat ggcgctgcgt tcgtattcgg tgaacttcta

34501 gtcaggaccg aaggcgtctt tgctttcagt gtaagtacat aaatttcttt atttcataga

34561 atgtagtaaa tcccctcccc tctcccgtgc ttccctatgt aaaatggcag actgttgtat

34621 taatgtctaa ggaatggtgc accgcagacg ttgccggtga tcttcttctt cagcatgctg

34681 gtggaggtgc tgttcttctg gggagcgctg cagtggttct gcctgcggct ggggcacgtg

34741 ctgcgctcgc tcacctccac caccgtctgc gagagcgtca tctctgtcgc taacgtcttc

34801 ctcggacagg ttggacacct tcatagttga cttaatactt agccgtcaac ctgttatttg

34861 tccagttgtc aactctttaa ttgacaagat cctttgttca tacactaacg atttttttcc

34921 tgtcagtcgg aatcagtgct gataatcaag ccttacctcg cgctgctcac cccctcagag

34981 atacatgtcg tgatgtcgtc aggcttcgca acggtatctg gcaagtcttt gcttttcatt

35041 taaaataaca atacaaaacg aagaccatca gactctgaac agctaattat ttttgcgtaa

35101 aagtatttct ctaaaccagc gattcccaaa gtggttcagg tggatcctct tggatccaca

35161 gaagccccgg tggttgtacg atgacgggaa ataatcctaa cttgaagaag aaacatagca

35221 atcattgcag gctgaactct ccgatcgcta tatttcttta agtcttttct ttcttctttt

35281 cttgggtgaa cagttgaggg ttatcctatc ccttgtatcc aggtacgatc ctagcagcgt

35341 acatcgcgtt cggcgcggag ccggcgcact tggtgacagc gagcgtgatg tcggcgccgg

35401 ccgcgctctg ctacgctaag ctgatgctgc ccgagacgcg ccgctcgcgc accgccgttc

35461 acaacctgca gccggttgaa atgttagttg acattggaac atatctatgt ggtcgtcact

35521 atcatacttt taggaaaaaa tacagacgac cgggtatttt aacgacgata tgttgtacca

35581 ggctcgtaat taggtgataa atataagtaa gacagaaata atagaagggg taatagtaac

35641 ttagatatta ggacgtgtgg gtccctaata gcgtgtctat gccgtcagcg aagaccaatc

35701 agcactgtcc gcggcgacgc gcggagccac caatggtgtg gcgctgatca tgaacatcat

35761 cgctaacttg gtggcgttcg tggcggtgat cgcattcctg aacggcgtgc tcggctactg

35821 cggcggcctg ctcggcaacc ctgacatcaa cctcgagtgg gtctttggca agatctttat

35881 acccctctgc tggcttatgg gtaagaaaaa gtagacaaac agtgagcaag taggcttctc

35941 aattttgttt ttcctcgaag cagtaggtac gtcgcgatcg aacgcccaag tggatgtgat

36001 gtgtttgatc tgcaggtgtg ccgtgggagg agtgcgagca cgtgggcaca ctggtgggcc

36061 ttaagactgt ggtcaacgag ttcgtcgcct accagcgcat gggcgaaatg aaggaccagg

36121 gcttgctctc ggtaatatca gtttaaaaag cggcacttgt tttgagtcag ttgcttttat

36181 cgttctgaag tttgattaca gaggagttaa aaactatcaa atatattttt aagtaaataa

36241 acccgctgta ggctatggtc tggttgtctc gataatcaca ccatgtcgac agccccgaca

36301 gatgtcacgc ccggcgagag gcggtgcgtg ttgggtacga ttatttacat ggtgtcacta

36361 cactcctagg actggaacgt gtcaccggcc aataaataaa atattgaaat taagtaatta

36421 tgattgcagg ctcgtgggga actgatagca acgtacgcgt tgtgtgggtt caccaatccc

36481 gcctcggcgg ggatcatgat tggtgccatc tccgccatgg cgccctcaca gcgagaaaca

36541 ctgtccagcg taagtgtgac ctatactact tacctacaca tacacacaag ggtcatgtta

36601 cgatggtaaa tcagttaact atttgacttt ttccaggtgg cggtgcgagc atttttcacc

36661 ggatgcggaa tctgcttcat gacggcttgc atagcgggta acttttgttt taataaaata

36721 gtaaaagttt attaattcag tctataagaa gaaactttaa tttgttcatt attttctttc

36781 aggtcttctg atgccggaag gttcattcgg ttaaacttag aaataagtaa tgactgtact

36841 taatattagt tgaataattg tgtatatttt ttatttatga ctaaactaaa taaacgataa

36901 tatttgtcat aaacttttat ttttcgaaat cagcacatgt taaaacccaa ctttgattag

36961 cgcgattatg ccatagaggt taaagcaaaa aggagctaag accagcaaag acctccatca

37021 gcagatatca gaggatcctt accccttgcc ttcgaaatga caataatgaa tctactgtcg

37081 aatactgttg gaagcagggt taagatctct gatagttatt attgtaaact ataaatttaa

37141 taaatcgttc aaatggctac actgattatt gaatgcttta tttttaacga aatagttaaa

37201 actttattaa ctaagaagtt tataattctc ctgcataaat actgtatcaa ccgttctttt

37261 agagaaataa aatagaaaag ccggtttcgc gttgcaattg tcagaaacgt cagagcacaa

37321 tgcactctat cgggaagtgt ttcgatttta taaaaaacct acgcaaaatc ttttgaaaaa

37381 gtgccttagt tatgataccg tatcattttc aaatttacgc gagtgtgtcg ttctttttcc

37441 tttgtaaaat tatttgttac ctttcaagct tcatatcaat gttggataag aaagttccga

37501 cattgatgag tgactccgga aaggaagaga ccgacgaaga tgtgtgcaaa tggataccta

37561 agaagaaaca cgaggtcctg aaggccaagt acaaatgtct caagaagttg ttccagatat

37621 acgacgcgag cgtgatcggc atcctgccgg agccgtacag cgcggagggg cgcggcgagc

37681 ggcggcactc gcggcgccgg cgctcgcacc gcaccaagac ggacgcgtgc gccggcacca

37741 cggagtccgc cgcgccgccc tcgcccgccc tggcgcccgc cgagcgcagc tcgctcacca

37801 cggccgtcat gcgcgacctc aagctgcagt acacctccac ctcgacgcag gagtcgctcg

37861 tgcgccgcga caagtacacg caggactaca aggacgccct ctccgtcacc acgagcgaga

37921 aggagtgcga gtgcacccag acctcggaga tcctgcacgc ggcgccattc cttatgatga

37981 aggactcgca accgaagccg acgcgatttc agctcttcct ccaacgaata cttggcataa

38041 gacgagagaa gtccaacaac ggcctcccct ccccccgcat gtacgcggcg agcgacaaca

38101 acatcagcaa tcgctacgag aagcggcggc gacgcgggat gcgatttcgt cggcttcgcg

38161 cgaagaaagt gtactcagag tccgcgctca gggagcgcca gagccccgtc atcctgagct

38221 acgtgcagtc cgtgcagagg aactgcttga tggacactac gccgcggcag tgccccttca

38281 tggggtgtag aatgatcttt tacggtgaga atagttattt tgtagaagtt cagaaaactt

38341 aggcttgaaa cttatagttg tataattttt taattgtaaa taagtagtga tactttattt

38401 attgtcccta ggcattatta actacaacga ccacatcaat ctatgccact tcaccgatcg

38461 caagttctcc tgtcactact gccacgaggg attcctgagg gagcgcgaca aattgttgca

38521 cgaaaatgaa catataggca tttccaaact gagttccaat ttcacatcta cattgtctgg

38581 tcatcactcg aaagtggcca acgtgacgca gaccgacccc gaacctaaac agatcaacga

38641 ggaaaagctc aagaagatcg tctcattctt tgacaagatc gaggatcccg aagaaatcat

38701 tgcggagcta aagaagagtc gtcactccgc gtcgaatatg aacctcagtc gtcatccgtc

38761 taaagtggac atggtcacat ccgggcccaa caggtccggc tccaactcgc ggaggtcgag

38821 ctgcgtccac ctgcagaaga cggacagaaa gtccagctct tctgtggaat ctgacacgtc

38881 gtcagtgcac acgtcagggt cgtcattcag atgtcaaata tgtggactta agtttgatca

38941 catgtgagct attatttttt cttatttatt gagcgagcct gttctcagta acgttcttgg

39001 cttcgtgcag atgcaaagat taggtagcta gatcaagttt ctgattttgt agctcctatg

39061 caaacatttt tgcagacagc gtttaaatat gcacgtgagt gccgagcatc gaagtggtag

39121 ccggtgctct gggcagagcc gggccgagcg gccgccgagc cctccgactg ctccgagcgc

39181 gccctaccag ccggggccgc cgagctcccc gcactaccag gacaactcgg acgacgagga

39241 gcccgcctcc gtgctcgcta acaagagcta ttcgacttta catcgcgtta atacatcgtc

39301 aacttttact cgcttcacct ccgaagagaa gtcggccatg agctacgacc catccaccaa

39361 cattatatat tattcttcat cggagtcggt gaagaacccg tcggtgatgg gcgagacggt

39421 gaagcgaagc cgcgccggct tcaacagcta caagtgggaa cctggaacca attctattcg

39481 tgcatgatcg ttttgtctat tcacgttata ttttcttttg gtttaaatat aaatgaaaaa

39541 tccattccgt tatttcattt ctgccagaag tcgaagtctc catttactga aaaaattggt

39601 taatgggttc atccttttac ttgtatcatt caacaaactt atttagcact ttacagtcga

39661 cgctggtgtt gctaataatt gtcccagttt cctttgaata agtaaatatt acgaattgtt

39721 tttagtttaa ttttagatac gtctagactg gtccactact gtaggggaac aaataaaaca

39781 actcttatac aaaccacaca tgtttattgc aggttttatt atttctacaa actgccactc

39841 gacgcgtgag atcgcaacag caaaataaac aatggaagcc acttttcaaa tctccctcct

39901 acactcgtcg ctaaaaaaac ttataaacca ctaacataac cttttctata tcaaatgtat

39961 gtaaaaccaa atgtatataa tatactttaa atggtaatat attcacctat aatatcgtat

40021 cgcttagtaa aataactaac agtgattaaa agataaaata acaaattcaa gccaatataa

40081 catgcatgga gtgttgccat ataaaatatt tttaagccga atacagttta aataatattg

40141 tgcttttcat atgtaatagt ttcaataaca aaattgaacg tacaacattt caaattgtgc

40201 aataaaaaca agtaacattt ttaatttcat atattataat acattataac tgacttgtaa

40261 tataatatat aaaatagtac agagattttg agggtagttt gaatttttta aaactaccca

40321 ctataatatc acggggaaga aaatatgttg ctataagaca aaattaaata tataaaaaat

40381 tgaccacccc tgtaataacg gataacagcg aacatgtcta taaaatgata atgtaaaacg

40441 aagataaaat gcgatacaaa aattttgaac atcatatttg gtttccattg ccgctatagg

40501 gttccgaaaa ccgtgatact catgtaaacc ttgtcatttt ttttgttgta tttcgtcaat

40561 ggaaacccaa caaatcgtga gcgatctcac agcatcccat ctttccctaa tcaaccatca

40621 catagcaccg caccatgata ttaaaccttc tcgagaatgc attaaacctt tgtaaaaaaa

40681 acatatcctg ttctatgact agcattttta caagtgcagt ttatattaca gaaagatgta

40741 taaaacgtta cagatacaat attgttagta cagtcgagaa aatccaaggc gttccacttt

40801 tagaacaatc cagacatttt aaaaaaataa tcaaatagca gtgagtttct taaactatag

40861 tataaaaagc atttcttgta ccactatgtg tctctttatt cctctttttt gcttttttgc

40921 agacaaattt aaatacactc tttccctatg gcttcattag accgagttcg cgataaaaac

40981 ggacttacga tgaagcaaaa tctccgtttt tatttcaaac tctgtctaat gaagctgtgg

41041 ggttataaaa aactaagtct cattggtctt tgttcccaag aaacttacaa gcagatgtct

41101 ctttttgttt agtacacctc cagtttcccc gactgtacat ttgtgtaatg gaaacagagg

41161 ttaacttaaa gaggtcggca gtgaaagtca acttcatttg accaatcaag gatataaaac

41221 atcgtataga caaaagacag aaaatctaca aaaaatatta ataattacga tgaaataact

41281 tttaacatga tgacgaagta aatatatttg cataaaaact aaagtaaatc gaaacttaca

41341 atttaaaaaa aatcactgca aagtctatgg atttttttta caattttaca tttatatttt

41401 gtgataactt tggtcatgaa tgaaaaatga acaaattgtt tttaggcaat atgtggagtt

41461 acatacattc ccattgataa aatttcatta gaacttttga aaaatataat aaaacggcga

41521 cactaaacac taatataatg cctttccata gacgtttcgt cagtaccaac attccaacac

41581 taataatcta tctctttcta tcgtaaaacg tttccaaaat aacctctctc tttcttaata

41641 tcaatttcgt tcccccacag ataatataag tgcacactac tgggttacaa caataatttt

41701 tgattgccaa gaattatgga attatattac aattgcagtt ttaaaaagtt tttttgggaa

41761 aagatgtatt atacaagagt caaattaata ttaagcaatt atcagacgcg tcatgcgaat

41821 gcatcaatgc tttaaaatat attggtttat ctacagaatg tagggaaatt attgtttcaa

41881 aatttgatga aaatagcaat aagtgcagat aatactgatg agttatcaaa atttcacaac

41941 ctaatatttc ttgaaactag attcagaaat ggaaatggtg taaattagga aattaaagcc

42001 aaaaacaatg aaatattttt taaatatttt aaaaacaaaa tgatcattct gatcatatgt

42061 agaatgaaat atttttcttt ttaacagcac gcagttacac gccctgacag aacctatatt

42121 aggggaaaat gaacacaaca gcagaggaac caaatacctg aagaaaatat tttagtttaa

42181 ccaaaatctt cggtaaaaat catccttgtc catcactaat gtcatcctat gtcttcagta

42241 gacagagttc gcgatataaa cgaagatttt gtttcggcgt aaggtcgatt tttgcgtgac

42301 tttgttataa aggggcctat aatcaaatcg tcttttttat atgaataata aaatattgtt

42361 ttgtaataaa aatagttttg ttttttacgt atttgatcca tttttataat taatacaaat

42421 acaaataatt attccataac tcttagcaat aatgataatt ttaatttaaa cattagccaa

42481 ttttaaattg atcaatattt ttgaagtaat acctatcatg tccttacgac cgattataca

42541 tactattcta atgtttataa ctagggttgc cacaaatcga ttacattttt atacacaaat

42601 tataaaaaaa atgacatccc tgtttttttc tttacaaaaa tactatcaat tagactttaa

42661 atattttctg cttagaaaca ttttatttga tatttaactt ggcttttacg tatattttaa

42721 aaatagccaa aataaatcat gatgtggcaa ccctacatgt cacctaacct aagcaacctt

42781 acgttcgcaa actataaaat agtgaaacaa agaaagaagc cctatacata atgtaatatg

42841 ctttaaaact atcaaatata aaaatctaat tactgtagtg acttgtaaca attctatata

42901 actgtaaatg cttacaatgg cgagataata aaaaattgca gtttatttac atgtaaaaat

42961 atttaaatat gggtaaactg tcatcatatt gtcatctctg tcatgctttg aaaaagacag

43021 ggacaataat acaagtgtaa aagcagttga aatacacgtt tatgtcacga caaacactat

43081 ttatacaaca ttttttgagt attgtttaaa atagggttgc ctacagtaag ttattaatta

43141 ataaaaaaaa ttacttaaat ttgaaaaatt acaaaattca ttagatatca tgaagctaac

43201 ttaattaaaa atgtttatcg gactagtaaa cttgttttct ataaaaaaaa gcattcctaa

43261 taaaaatcat tatcattatt ttatcaatca tatgcatttt tttatatcac taatgtgaaa

43321 tagtccatta attttatatt cattaatatg attaaaataa aagtttttcg tcttattcta

43381 agatgaaata tgtttgcgat gtaaacttat aaactattat aaaacaatgt taagactcat

43441 tttggaggta attgttttga gctacccttt ataatttccg tacaaaataa atggccaatg

43501 taccggggaa acaaaattag tttctgccac aatatctgaa aatacaaata ttaaataata

43561 aataactatt ataaaaaaaa tggaaaattt ataagagtca tgtcaaagac ttgcaatata

43621 tgataaactt aatatacgat atacaactaa aattctaaac acatatttta tcattttata

43681 aaaaattgca aattattgag ttacgacatt ctacaaatat ttgagttacg tcattttaca

43741 aacttttgag ttaagtcatt ctaaaaactt ttgagttacg aggttccaca attttttgag

43801 ttacgacgct ctataagttt ttgagttacg atgttctaca aatttttgag taacgacgtt

43861 ctacaaattt tagacttagt atatttccag aggggtcatg taaaatggtg ttgtatgtac

43921 ctatgacatg gaggagcagg gcagacgcgg gctgcccatc tgcgtgagca cgcggtccag

43981 ccactgcagc ggcccgttca ggtgcagctc gatccagcac ggagtagacg tcaccgtctg

44041 acgtctgcac acgcacaccg cattacaaat taaaccatca accgtagaca aactataatt

44101 tacgtaaata atagtttgtc tttggtaaag tctatggtta aatctcgtta actccccaga

44161 gttggacatt tgtaacaaaa cagtttgtct catttgagta tatacattac tcatatttta

44221 caatatcttt ttattttaat tcacaaaata acttaaaaat gtcaaatgtc gaaatggctt

44281 atggcaaatg tggtaatgga taaggggtct taagacctag gtcttaggtg tcattggaaa

44341 tgggctgtta aatcctagat cttaggtaac accgtagaag gggtcttaat tcctagattc

44401 taacactgga aatgggctct taattcctag atcctaggta acaccgtaaa aggggtctta

44461 attcctagat cctaacactg gaaatgggct cttaattcct agatcttagg taacaccgta

44521 gaaggggtct taattnnnnn nnnnnnnnnn nnnnnnnnnn nnnnnnnnnn nnnnnnnnnn

44581 nnnnnagatt ctaacactgg aaatgggctc ttaattccta gatcttagat aacactggag

44641 aaggggtctt aattcctaga tcctaggtag cactgtaaaa tatatcttaa gacccaggtc

44701 ctaggtcctc atacctatac tcagctcccc atcccttgac gaagctcatc cgtatggtgc

44761 acatgcgcgt cagctggaac accgcctcga agccctgcga cactgactgc gacagcagcg

44821 ccgcgaactc ctggttgttg aagatcttca ggttgcaacc tttgtttaca agaaatatac

44881 atttaaattg cagaaaaatc taaatattac atctttatat ggacgttatt aaaacttaag

44941 ttagatacaa aaattatgtt ttttttaggt taatcgtgtt cgtcttacgt taacataatc

45001 cgtactatac tattataaaa aggaaaattt tgtatttgta tgtattgaaa taactcgtac

45061 actactgggc cgatttcaaa aattctttta ccattagaan nnnnnnnnnn nnnnnnnnnn

45121 nnnnnnnnnn nnnnnnnnnn nnnnnnnnnn nnnnnnnnnn nnnnnnnnnn nnnnnnnnnn

45181 nnnnnnnnnn nnnnnnnnng gtaaaataac cataatgacc ttgagaatga gaaaagatgg

45241 gaaatctaat agaaacattg aagtgtccac tatggtgagc tgagtggtgg tccttgtggt

45301 aagcagcatg ttgtggtgac aactcacacg tgcacagtgg tattgccagg gacctgctga

45361 gcgagcgtgg cctccaggta gagcgcgctg tgcggcccca cgcccaccac gccgccgctc

45421 tgcatcacgc ccagcgacaa actataaaca aattgcatat atttattcct ataactaata

45481 accgataatc caaagtgatt ttgataagac aaaaagtttc atttttctaa actaaaagta

45541 tattgttcat tagatttatt gactatatta tgttttgcaa gcgacacaga ctgtaaagaa

45601 ctaaaagaat agcttatgtg ttcttctaaa cttgttctat atttcaccaa gatctgtaga

45661 gccgtgttgg ggataccttc aaacaaagag cccttcatct taactttccc atttataata

45721 atagtaacac attaaatacc caaagtacat tagctatgaa gcgattcnnn nnnnnnnnnn

45781 nnnnnnnnnn nnnnnnnnnn nnnnnnnnnn nnnnnnnnnn nnnnnnnnnn nnnnnnnnnn

45841 nnnntcacgt aacttatcaa atatctatgt cagtttcata cattttttgt cgattcnnnn

45901 nnnnnnnnnn nnnnnnnnnn nnnnnnnnnn nnnnnnnnnn nnnnnnnnnn nnnnnnnnnn

45961 nngggtacct ctgtattata aacaataatg tcttacacgt cggtatggtc gtgatgcggc

46021 tgatgatagt cagtgtaggg cggcggggcg cggatctcgc cctccgcgca acgcggcacc

46081 aagataggcg gcaaagctga acaaaaggat ttaaaacatg cttattaaan actttaaaaa

46141 gatctaatta atgcctcctt attagtactt atatggtcga acatggatag agtccaaggg

46201 gctgttatat ttaggacttt ttcgcttatg gaggtcgtct atcgcgagca tacaatagct

46261 ctcgcttatg taagtttctc cctagaccag gttcaactgt aataacaatt tgcattgggc

46321 caacaatgct gactatgctc tggtcacccc taacttagag taggtaggtc cttaatagag

46381 atgtataaga ccaaacacat cttaatatca tattacaata tttacagtta ataataacaa

46441 aatattagct tcatattctt accgggcgag tcaatcttat tgtagtgata aggattgatg

46501 cacacctcgt cctttttcaa ctgatatgca taaatacagt ggtcgactgg ctttaattca

46561 tgctgactct gtaaaaataa acatttttta tttgtataca taaaacctaa aacaatgctt

46621 ttaagttata tactatgttc taatttatgt aaaatttcat taaaatccat tcagctgttt

46681 tgaagatacc ttcaaatatc cctttatcca ttttttcaag gtttgttttt agaggtttct

46741 ctataatccg attttctcgc ttattgaggt agctnnnnnn nnnnnnnnnt tacagaccat

46801 agaattaccc taacttatcc agacttgact gtagttgtaa aagtatgaat actaaagacc

46861 agttcccatt tcaatgtcat tcatataaat tgagtttaaa aaaatggtta agatacattc

46921 aaacacaaag acgaccttgg agtatgatta ggttgactaa tacccaaata cacaatgggt

46981 tgccaagttc aaaaaaactt gtataacttt acaaaatcaa caaagttcag atttgttagt

47041 ttaatataaa gatatttatt actatctaat ttaaagccaa taacaataaa ctctaaaatt

47101 aaattcagca agtaatctat ggttttaatc accatagaca tgttttaacc attgttgatg

47161 gttttgggtg tactcacttg tagctgcggc cagcgccaca gcctgcagta gatgacatgc

47221 ggcaacccct ttctgtattg accattcaca atgttatcat tgggtttcgc tctgcaagcg

47281 aacatgtaca aatacttcaa ttaagttgag aacaattata tctcggttgt agacaaggcg

47341 attggaatct agaactttag tctcttagta acttactatc tcatagaggt ttctacacag

47401 gtaaaactgg tgtattacaa tatattgcat aagccacttg gagactaaaa ttgcagattt

47461 gtttagcctt gtgtatgaag agattaaaat tagcagttat ttcaatgcat aaaatagcag

47521 ttattttaac ggttcaggat tttttgctca aaataattat gtagatatat gcattgtggt

47581 aagtacataa cattacatgc agatagtaga atattttaca ttgtcacatg taattttatc

47641 actctctaaa tatttacttt atctattaga taacacacag acatttttat gttaaggtta

47701 tttctaacta ataaaccaat ccattagtgt tttaaaaaca tcatagtgtt annnnnnnnn

47761 nnnnnnnnnn nnnnnnggcc actgtaattg caaacactta aaagcacaaa aataaaaaag

47821 aaacatcaca cattattcac aaaatattca atataacact caaaaattag caagagccag

47881 agtaaaaaac cacaattctt ttaatcttaa ttaatgtttg catcctatag ataacattta

47941 acacttttca ataaattgaa ttacgaaaac acgtgcgtaa attgcaaatc catttgttaa

48001 tcattttgat tacacttcag attgcagtca cataattcta ataagaaaag gtatagaaac

48061 acagggtcaa taaccggtaa agaattacat ttagtaacca aacaagaaac taataagaaa

48121 aatatttcaa acgtttgtac gataaattat gcttcaatta tgaatgaata gttcaaatcc

48181 aaacaaacat gctttaggca ttttattacc tggattatag acgaaagcga gtaatgtcag

48241 tacgtgaaat aaaatcaata aactaacatc ggtgttcaac cattaaaatt gcacattttt

48301 atcgtaaatt agcgattatt ttgtgtgaaa actaaaaagg ccgaggtccc aaattcgccg

48361 ccatattgat aaagataaaa aagcttcgat tgttttgacg gcgatttgac cttccttttt

48421 ggattttgcg tcctgtctat ggttatgtaa ctaaaatacg atggtcattg cattctgagg

48481 ggtcattgtg cttttggtac tttgcttttt agcaaggtta tattgtatgt aaaaaaataa

48541 tgattaattc aagtaattaa gttattatta gcccttacaa atgcctttat ttattgttca

48601 tataaaagta tgaatttaat actctaacta gaatatgtta tcattgtcta tattcctgtt

48661 cttaacgtca taattggcgg caatctataa taattttttt ggaactgttt ataattcgtg

48721 tctatcacag aatattaatt tggtaataac aacgacaaaa gcaatttccg ggtatgaaaa

48781 aaaataacaa ccatcaagaa aataaaaaaa caaggtctta tcagcataga agtatttaaa

48841 agttgaagac cggggaaaat ttaagcaatc ataatcaagg tcattgttag atatcatacc

48901 aaaacgtttg ttatcaaaat aaaaataaag caatcaataa ataacctggg aattgtgacg

48961 catttagtat gactactttg actagtaata gccttttcta actcctcgag agcctcactt

49021 ttctttaatt tcttaactaa actttttaca gccttttctg accatttatc ttctgcggca

49081 gatgatcctt cgggaccttt tttccactct agaagtcgtt tcactactgg cggcgttcgc

49141 ggaaacattc cgccaaattt cactttttaa gtcattcacg gaaactaatt tacattagat

49201 tactaacaat acacactttt tatttaatgt cttcatacat attatattct aatttaaatt

49261 aacttatcgg ccattacaat ttgtgaatgt gattaacaga ataggtagaa aaatgacagc

49321 gtagttgcca atcacaataa tttttcttac aacatttcct ttacttataa aacaataatt

49381 caattttaaa taagttttga ataaatttat tttcctttat ttatcataat attctgaaat

49441 acaaccaagg aatttgcata ctacatagga annnnnnnnn nnnnnnnnnn nnnnnnnnnn

49501 nnnnnnnnnn nnnnnnnnnn nnnnnnnnnn nnnnnnnnnn nnnnnnnnnn nnnnnnnnnn

49561 nnnnnnnnnn ntcaaaagtg atccatttcg tggtcataaa tttgtttact gattatgata

49621 cgtaaatcta aacagactat aatagaagga cgcatataac gaaaacatat gttaaaggat

49681 ctgtaatttt agtgtaaaaa aatattttgg aaattgggaa aattctaaac gatgctgaca

49741 acactagaaa gggacaacgt gcttcagttt ttgtgttact gctcggaccg ttttcttagt

49801 atgcagtgcg cagtccacta taacgtcctt gaatacaaca aggatttatg gcttacatat

49861 ttttttatat ttatctgtca gattttattt tttgtaataa aaaggtccta aacattttat

49921 ataatttaaa aagtgtatct gtttataaat tgaaactttt agatgtgcaa caaaaacttt

49981 atacattttt aacagaatta gaaaattaaa taagcttatt gatcattcta aacaaaaaaa

50041 aaattggtaa atgtttattt tactgcttcc ggtattttca gttttcaata gcaacaattg

50101 ttgtctatgg aaaagagtgg tagatgttta cagcatcgct tattttacaa cattttaagg

50161 aaattaaaag gaggtaacag tttccattct aaacatgcaa taacgtcgta caaaaaaggc

50221 ttttcattat aaaacagaat ttcatggaat aatttatagc atatctaatt atctaatctg

50281 tatcttttaa nttacaaaac cgcccctact aaaagactac tgttattgac tatttaacaa

50341 cgagtgccct atgccaaaac gtcaaactta ttggactttg gtgaatacat aattaggata

50401 attttatttt atctgtatgg taacactgtt tttgttttga aattaaaacg ccatccggtt

50461 ttgtttgttt tgtttcaatt gttaattttt gatgtgccag ttgctgaaaa ttcatttgct

50521 atattacggc aatatattat ttggttagta ttttagtttt ggcctttata atagtgttat

50581 taacctgatc tttaaaatgt attcctactg ggtactgtta ctggccatca ttgttattgt

50641 agtggcctgt cacaaaatta tattttaatt ttcccgcttt gacttttata gcttggctgg

50701 aaaatgcatt ttcgttttca atttaagata aatatttttt ttcttaattg atttcatttc

50761 tttttattat tcaaaaaatg ctatattttt attttttttg tttaatttta ttaaaaaatt

50821 atcattataa aatgtttgca ttaccatttt ataataacca atgctctgat atttaatatt

50881 ctgattttct aataaaatta aagtaaacga aaaaattata aaaattgatc actattgtag

50941 tgaaaatatt tttttacatt tagctaaaac agatatagtt ttcttaacat atcttgttag

51001 tcattaaaca gcattgttat tgcttaatat ttcttgtaag agtctaaaaa tgatgaccta

51061 aggtcttcac tgagcagctc ttttagccga agcattcaat tatatttttt cagtcaaaaa

51121 tattggaaaa ctaaatatat ctgtgaaaat attacacacc tgtcttattt ctttttacat

51181 ttaccgagtt tgaagaaatg tgaactcttc atatgttgtt aaaatatata aaatataaac

51241 tagctcacta tacatgctta ccgagaggct cggagcctat cctgtgtttg ggatgacaaa

51301 gccttttatt taataattct atataattta actatatgtg ggacattaga aaagcaaata

51361 aatggtctaa gggacaatga tagtcgctta tttctacaat aaaaagtaaa ctatgttctt

51421 tcttttaagc aatctgcttt gtattgtttt gttacatact atgtctcaat atttctgagc

51481 attgtgataa attgtaatat aaaaaatagc atatagagta tattattaat aatagaataa

51541 tagacaatga aacttgtata aacaactttt actatacagc tatcctaagc atggaaccgc

51601 agcttccacc gttactactt tgtatgcaga atatgaatgg agaagaagta tcacttagta

51661 tcggacctaa tgatgatttc caaaccttcc tggataaagc caagtacctt tcctttatcc

51721 ttttatcctt ttcttataaa ctacatgact cctgaccctt gcgtgtgtct tgtacagtgt

51781 tacatatcac ctaagttatg acatataggt atgaactttc aattaataac tcaaaaacta

51841 ctgaattgtt taataaaacg agacctactg atacgcatta cctttcgaat gaaaaaagaa

51901 tgatcaaatc ggacaactag gtgccgagtt atcagtgaac acaaaaaaca cgcacagacg

51961 aattgactac ctactccttt ttggatgttg gttaaaaaca gtatatcttc ctttagaaga

52021 atataatggg aaatctgttt tcactttctc catgtttagt gataaacagt ggcacttttc

52081 tatttcagat caacgctagg tttcgacgta gacataaact cgattactgg cgaccaaaca

52141 gtagcattga acgaaaacat ttatcattac ctcctaaatt ctgaactaaa ttatcaaact

52201 gatccacaat taggtactga aagtttccag cgtacaccag aacaaggtgc acatagtccg

52261 gacggttttg tctacgtttt agacgacggc acgcagatac gagcctcgca gatacatttt

52321 gacaacgaag atccccctgt agaccttaca gcggaacaaa taccattcgt caaatacgcc

52381 aatgatagtg ccgatgaaca cgaagtcgaa gatcaaagta tagaaaatac gaaaaaaata

52441 aacatcgtcg aaagtcctgt taaaagatgg gaaagtagaa cttccagtcc tagatgcagc

52501 tttgttaata gcctaccgtt caaactagtc tgttctaaca cgtccaattt cgaagctcaa

52561 tttaccaaat atttagaatc gagtatgaca aaaacattta caactctcag tcctgttacc

52621 aatagaaata aatcccctaa aagtttaata agtgataatt acaagaatta cgatgacaac

52681 tatcaggctc agcgaaacaa agatgatttc gcgagttata cacgcgaaga aatactcaat

52741 atgtttaaag actcgcccgt cgcatcacta ccttatgaag agaaccaaaa tcataacgag

52801 aaaagacgcc acgtacgtaa aaccgaccca tcccgacttc ataagaattg gagtaataga

52861 ccttacgtag acgtaaaacc tggagagaaa ggaagacaaa actgtttcat ttgtggtaaa

52921 atcgataata acgtggaaaa attgtattta ttcgataacg aggaccaaaa gctacataga

52981 tgtacatcgc cgaggaatta tccgactcaa cttaaaataa tatgcgacca ttgtttggct

53041 gataacttta aaccgagtcg aatgaagagt cctacacaat ctctcagttc ggatgagttc

53101 ttagttatta agaacaacca gcaattcata tttcaaaagg ttattgattt taaagtggct

53161 cctatttcgg aaacctcgga taaaagggat ctcaaccttc tcgaagattc ccaagagagt

53221 gataaattag aatttgttaa agtagagata ggttctgatg gagaaattgt aacgaaatgt

53281 attgacaacg atactagatc ttcggatgac gttataattg tgaaagacga gaaaaaggat

53341 agttccagtg atgtggaaat aatagagaat gatccagata ttgatgatca cattatagat

53401 aacttggagg aagctgatga ggatgttaag gcgtttttag ggaaatatca ttgcgatgat

53461 gatactgaga ttaaggaatt aaaatgcagg ttcgtttagt ctctttccac gataataatc

53521 ggagtcagat atctttattt aatcaataaa acagttaaga agttattaat attttattaa

53581 gaattcaatt gtacataaaa gcgaagcgta ggtcgataat atcaagatat ttccaggttc

53641 tgtgaccgaa tatttgaaga aattagtcaa gtcatagacc atggtgagga gcataagcat

53701 aacttagatg atggagaagt attcccttgt ccgttgtgtg attatggtat aaaatatttt

53761 ctttaatagt gatatgatta caaatatgta aatgatttcc tgtctagttc taatccaaca

53821 ttttggcaaa gtattgtata ttttaaatgt ccctaaattc cagtaaactt tgggcttaag

53881 gtattgatgt taacatatag acttaagaag ctgttttatt ttaggttatt ctaattttaa

53941 atggttgaaa ggtcatctta aagcagctca tgataagaca aaagttaccg aaataaaaga

54001 cgaagaccac gagggtgaca aaggtgagtg aaagactact gatcttttgg taaaacatgt

54061 aaagtttgca aaggcatata aaacgatgat tttataatat aaatgcaggt aaaaccacac

54121 catcgtcgtc gccagtcgcc aagagaacga gaagtgcggt aaaaaaagtc gagacagaaa

54181 acgataaagg tctgaatatg aacatgttaa tgagtcttac agatgtttcc gtggaaactc

54241 gcggttattt atctattagt cgaacatccg aatcataaaa ttactgcttc tatattggaa

54301 ctactattaa ctttgccatt tctaagttag ttggatgttt cccttttttc gtggttaaat

54361 aaaatacata atattaacat taattttttt cttaaaaaat tcagcctctc cagagaagat

54421 agaacgcacg aataatataa ctacgaaact tgaatctgtt agtatatgac attaagtatt

54481 ggtaaagccg gctacaaacg ttcagcttta actgcgcagt tttaactgta cagttaaaac

54541 tgacgtacgc gtannnnnnn nnnnnnnnnn nnnnnnnnca atttgaactg cacagtcgaa

54601 ctgtacaatt aaaactgagc gtctgtaacc ggctttagtt agttaaatca gatctttagc

54661 ggttgcagta gattagttgc tagttactac taaaaatagt tctcaactaa caatggcatt

54721 caattaaatt aatgaaataa atgtagaacg ggtcaacatt gactgtgaac acggaggtga

54781 agcaggaatg tctagacagc agtgatgatg aagccatctg gatcgtgcag actgtgggcc

54841 acgaaccatc agagcagctg gagaacttgc tgaaagtggc tgcggtaagg gtctcgttag

54901 ttttcagata tcttggcgcc tctaagcttc gtgcgtgtgt taagcgcaga acatgtgaat

54961 tttagatgta gcgaggatta gaatttacaa atttccaaat ataaaattag ggatcattcg

55021 ttagactttg aggaactttt agcattgtct ggaaatgcta tattcgaccg cgtaaaatta

55081 ttcaagtaaa aactaaatta gctcggactg ttgttgatga ttacttggtt ttatcatata

55141 caatacaaat attcgaaact aatttccagg gtgaagacaa agatgagaaa aatagaaaga

55201 aacataaatg taacaactgc aggtataagg atagattagt atttgttatt agatttgtat

55261 attttatnnn nnnnnnnnnn nnnnnnnnnn nnnnnnnnnn nnnnnnnnnn nnnnnnnnnn

55321 nnnnnnnnnn nnnnnnnnnn nnnnnnnnnn nnnnnnnnnn nnnnncgtca aatggaagct

55381 attccgtgtt ttgtctgacg agtgtggtgc cggaacggac aggaacagga cgcacnnnnn

55441 nnnnnnnnnn nnnnnnnnnn nnnnnnnnnn nnnnncctcc gtagattaag ggtagacaac

55501 gcatttgcaa ttgcagatgt ctatggccag cggttgcttc gctttttcag cgattccagg

55561 agaccgatgg ctctaacttt tgatagaaac gttagttctt ataatatagt ttatttttag

55621 tttttgagtg taattttcca gtcaaatatt cccaaccgcg gagagcctaa tctcccacaa

55681 gtgtcgtcgg aggggacgca agcgcagagg cccagtcaaa gacgactcgg tcatactcgt

55741 tccttctgaa gaggactttc tgcgggtact aaagaaaaac tttaatttgt caataaatat

55801 actgaccgtg aatttaaatt gtaacatgta taagagcact attgttgtct ctgttttatc

55861 taaaaagtaa gagggatggc aataaaactt ctatgtatcg atttattttt cagagggctc

55921 aaggaagacc taaacaaaag cagtcaggtg ttgacaatga tttactggtt attgtaagta

55981 aataaaaatt atttttgtat aagactgcat aggatttttt aaaatattta ttcctcttta

56041 tgcagagacc tcgtaagaga aagaaccgtg agccgacttc tgaccctcaa atagtgacct

56101 gccacaactg caatgaatcc tttacatcta aagttagact caagtttcat atgtaagcgt

56161 taatatcaat tttaaattat ttaattgcta taaaataatc tgaaaaaaaa aacaaacgtg

56221 tatttcccta ggcaattcca cgacgcgact aaccttttga cagccgaagg tcagtactcg

56281 tgcccggagt gcgagggcgc gcacttccct actgaaagcg aactctttga tcacgtgcat

56341 ttccagcacc acaagcaaaa gcgctggcag tgtccagtag aagattgcgg aaagacattt

56401 tatctaaggt atatcacaga atatacaatt tcttctttat ctactgattg ttttcaaaaa

56461 aaacctttgt aatatatgca ctctgtattc tagtgtctaa gctttatttc agagcaacgc

56521 taacaaaaca cagcagaacg catacggata cgcggcgata cgtgtgcgta acgtgtggga

56581 aacggttcct tgacaagcag actcttgacg agcatggagt cacacattta caggtaggtc

56641 ataaaacacg tggctgactc gccttgtgag gcttgcgagt ttactttgat actttcagtg

56701 ctactgaatt gccaagtaaa ttcataatgt ttcatttatg tctcgctatg ttcaaatgct

56761 tgtcctagat aaagccgttc cagtgccaca tctgtttgaa gcagctgacg cgacgatccc

56821 gtttacgtat gcatctccgc gcacacgagg aagaactcgc gccgcggctt gtgctcgtat

56881 gtgccgcgtg cggccgggcc tttcgcgaca ctaatgacgc tcaggtaggc atgtggcgca

56941 cgaagggttg atatttaacc tacacgacta acgaaatcaa gacaaggcgt agtttatatt

57001 aatttaatga aggtcataat ttttttttac tcactgttgt tcgcgacttc gtctgcgtgg

57061 aattaaaaaa tatatcctat gtgactcggg gataatgtag ctttctaatg gtgaaagaat

57121 ttttgaaatc ggcccagtag ttcttgagtt atttcaatac atacaaaaag ttttcctcat

57181 tgtaatatta cagtggttac ttataggagc acgctaacaa atcgaaggag tgcatagagc

57241 agttaagtcc gtctgtgaaa gaagagaacg aagtgtcagt actgctctcc cccactactg

57301 gtttagtttc acatactgtc gcggttgttg gtgagtttta gttctacatg atgtgttcaa

57361 aataattacc tgatgatacc gaaaattatg tgttcgaata tactaaatat gagaactagt

57421 tagtccaaga gacagctgtt tttatctcgt ttatagaggt ttctctctaa tttttcgctt

57481 ttggaggtag ctcgtcagac tagagaataa ttctcgctta tccaggtttc acggtatttg

57541 acaaactttg ataataggca aacggtaaaa acctacatgt atgtgttatt tctcataatt

57601 ccgtatatct ttatttacat atttattata taaaacattt cagtttaaaa attaaaaaaa

57661 aaataaaaca tattacataa aaatagtgca ccaccagctg cggaatccga tgactcaagc

57721 aaccagtggt cagggctgca gagaagaaac ctcctcacta tacgtgcggt ctttaatatt

57781 ttatttctca tattataata gctaaatccc cgcaaatgga gtgcggtccg gagggagacg

57841 cgctgctcgc gacgctgacg gacttcgcca agatcatcat acgtgtggta gagatcgaga

57901 aggccttccg ttgcgagtat tgtgaagagt atgtatacgt ttacattgat gaaacatata

57961 caaaaacatt gtggacccag attttcaata gcttttgttt gagtcctttg ggaatacaaa

58021 ccctcagtta tttcatttta caagtataga atcaaaactt tatgttctat acgaaaggta

58081 ttttgtatta gcgtttcctg ttgggccgtt cgggcaaaac gaatggccca acaggaaacg

58141 ggggtacaga gcttaataaa cctagtgatg tatttagtct tttaattatt gatgtgtctt

58201 tcttgcagtg ttttctattt agaagaagca ttgaacagcc atcgggttat ccacaaaggt

58261 gttaaaaatc ctttcacttg tcacatttgt aaagtcagct ttgctactta ttccaggtaa

58321 ataggtttcc ttcatcttga aatatataaa taatttgtat gatgtatttt tgtaaaagtt

58381 ttctgcaata gaaaccaact attatagtct aagaacaaac aaattaattt aattatacaa

58441 actaactcaa atgggtatcc ttaactctgt ctacctcctc ttaagttatg atataactgt

58501 atttcggccg attaatattg atactttttt accaaaatta ctaataacac tttctatttc

58561 tacgtagcac gactgatacg ttctgatact tgatacctaa gcacttattt agatattcag

58621 gctaatatac atgtgccgat ccccatagga gcaaaggtag gtaatttcga catatattac

58681 agatgtacaa cacacaaaac tacccacggt ttttacaagc gttctctagc agactgcaag

58741 aaggctgcca aatgtcccgg cacttcggaa gccgggcctg ccgctactgg cattttaggt

58801 tatggtggct ttcctgtggt taaacatttc ttatgcgagg tatatagcaa gatttttaag

58861 ccttaatcct tttttccact agtgagattg tccgcgcgac ctagctatga ctgtaattta

58921 gttcaacaag gatgtctgaa acggcaaata aggtgctggt tgcagttgca tctataggaa

58981 agtcagaaaa catacttaat aacagaaaat gccataaaat aaataaaaac agctgtacta

59041 cgacaaagat gtttagattt tttttaacat tggcagggtg gcgctcggtg tcataatttc

59101 atttatggtc accgggtcag gtttccttgt agatctagta agaaaaactt atctggcctg

59161 ggccagataa gggcctgtta ctggtaacag ggcctactta ctgctgccaa ttaagctcgt

59221 cgcactatgc atttttgtgg aaatccactt aatgtagtgc tacagcagta gtgcggttaa

59281 tcgcgtagtg gaataagggt ctatataaga aattacgtat ttgtcaggat tgcggacgtt

59341 cctatctgca ttggacatat ctacaagtac atcgacgtat gaaacatgcc aatgagaact

59401 ttctatataa gtgcaaccag tgcgatctga cattccctaa caggtaacta ttatattaat

59461 aggggtcatg atatactggg aaattggagg aaactagaaa atttaaacta gctgttgttc

59521 gcgacttcgt ctgcgtggca taaaataata tatcctatgt tactcgggga taatgtagct

59581 ttctaatagt gaaagaattt ttggaatctg cccagtagtt cttgagttat ttaaatatat

59641 acaaaaataa aaaaatctta cctctttata acacccacaa acgcgacaaa aatgttaccc

59701 tcctgaattg gattataatc tttttttata caaatgtgcc ggaacagttg gagcgtggca

59761 taccatagga agaagatgca cggcaagagt ggacccgagg acggcggtgg taccaccaag

59821 atagccagag atgactatag gtacgggtaa aatctataaa cagtcgaact tggataagca

59881 agaatccaag ggaccgcgat atttgtatcg cttatagagg cttctctaaa ccgagtttct

59941 tgctttctcg tagctcaacg ggaccagaga ttgactctca cttacagagg tttctcactt

60001 atctaggttt gactgtatga ttgttaagcg tttcttataa gaaaagatag attttgatca

60061 tactttgatg gattttttgc cttcagatct ataaaataat aataattaaa gaaaatcgtt

60121 caactatatt gcgttttaga atattaatcc cgccagaata ttctacaaat aaatcaaatt

60181 gaaagttaaa ttgagcaaag tgtaacggta acatgttata gtgacttttt ttatagtgta

60241 caggtgattt ttaacttaaa aatatgtttt ttttatggca ggataccttg tagagattgc

60301 agcatggtgt tgccgaacaa aacagcactt tatgcgcata gacagaagga gcatagtgat

60361 atgacacttt ctatgagcga tgaacaaggt gcgtggtatc cttatcctta catagtataa

60421 aacaataact ctttttcatg tttgtcccta tatatgtttg aatctttaaa actacgcaac

60481 ggattttgat gcggtttttt aatagataga ctgattcgta aggaaggttt tcatgtataa

60541 tacattcaca atatagtaag actgataatt cagaggtttt taatatgatg taaattaaca

60601 catttttagc gctgatattg caaattgcag tatttagtat cagcattata ccgatttgca

60661 ctcgtgcgaa gccggggcgg gtcgatagta tttcttaagc cgtacgtcta tcgcaaaaga

60721 gtaatgagcg gagatggtga gctgagcggc ctcaaatgtg cttgcgtccc aacaaagagc

60781 tgagctaagc gagcacaacg gcataacgta gctcatttga aacagctcgg ctctttttgt

60841 tggtagacac aagtatgaag tttcgtacga aagtggaaaa tggggccgcc ctcccgctca

60901 gctcttgcta tataatagtg tgtacttatt tatactatta tttttttctt ttttaaaagt

60961 aatgtttgga ataggtgcag atgcgcaagt gcgtacacac gcgccgctag cgacttcttg

61021 cagtaaatgc ggtcataact ttaccgagcc cagcgcgctg cagcgacatc tcaagtatgt

61081 tattgcattt taaaacatta tcacagaaaa ataatgtttt taatggatgc tttgaacgat

61141 ataaaatgcc ccgcgtactt ttattcactt tagtaatgtc agctgatgtc tgatgtacta

61201 ttgtttattc aatgacgaaa gttaggtaaa ttatgaattg ttaattcgtc tataatatga

61261 caattttgcc gctccctgaa tttgccctcc ctcaaccttc taaatcgagg gctttatgca

61321 cgagatccct aatttagtgc aacactggcc cactcagtgg accttatgat aaagctatgc

61381 ttaggtactc cctacagcct tgtttgcacc aagatagtat tttaattgag tagcaagtta

61441 gtaaaaaaac tagcccgaat aacaatttac caatctactt tactaaaata ttgttgtacg

61501 gatatgttta gttacttact tgactaataa ttaaaagata tgtgtattca cgcaaaatac

61561 gcgctagtcg tggagttgcg gaaaatggag cccatcatgg gtcaaaacct ttaacctttt

61621 ttttttactt gactaaaata ctaacctggt gcgaataagg ctttagtgat cagtctgagt

61681 agagtaaggt gtgttagaga ggtgcacggc cacgagggct gcgggcgcgg ggagcggcgc

61741 gcgcacgcgt gcgcggtgtg cggccgcgcc ttccgctccg cctccgtgcg caacgagcac

61801 ctgcgcgtgc acaccggcga gcgcccctac ccctgcgacg tctgcggggt cgccttccga

61861 cggtatctta ctataaacct aaggcttcat tagaccgatg tcacgcaaaa aacggcctta

61921 cgacaaagca aaatctccgt ctaatgaagc caagtcagcc acctcactta tagaggtgtt

61981 tttctgatga gtttctcgtt tatgaaggtc ttccgtccgg accaatgact ctcttataga

62041 tgtttctcgc ttagctagat tcaactgtat tttataattt cactagttgt cacccacgac

62101 tccgagcgga attaaaaaaa atctttgtga ggagacaatg tcttcttcca gactgtattc

62161 tacgtctgtg caaaatgtca acatcggttc agctatttta gagatacagt cgaacctgga

62221 ataagattaa tttgagattt ctctaattag gtatttcgct tatgaaggtt gtcccccggg

62281 aacagagaat tatagatatt tcccacttat tcaggtttaa cttcattcaa acatccagca

62341 ttagaaagat tacgtttaaa tgtattacaa agtacccacc atggtaccag gtcgacagca

62401 atgcgcaacc atcgtctgat ccactccggc gtgcgcgcgt gggcgtgcgc gcgctgcccc

62461 aagcgcttcc gcatacgatc cgacctgcgc acgcacatgc ggctcaagca tcccacttac

62521 atgattgtat tcgaggtaca ctatgctata acattattta taagaataaa atatagccta

62581 tgttactcgg ggataatgta gctttctaat ggtgaaagga tttttgaaat cggcccagta

62641 gtttttgagt tatttcaata catacaaaaa tacaatgttt ctatctttat aatattaata

62701 tataatatag aaaaatgatc taataaagcc ttcgggtaaa acagtaacgg gtagtgatag

62761 aaatttcaat acacgaaaaa tgtagataga tagatcgaca gatgtagatc gtagatgtat

62821 aaatagatgc acgaaaatac atataagttt ttccatttaa attctcagat gaaaggttta

62881 agcccaacct atgaagagat aatgcaacac ataagtgaaa ataatatcac tcacggacgt

62941 attatagaga tcaccaagat gacatttgaa aaggtaagtg gacaaaacat gggcgtcacg

63001 agcagttcaa atacataaag acttttatta cagtttttga cccctttctc ccccttgtca

63061 tcagaagtaa gcaacataac cccctcccat ggtcaggtaa acattggtac tcgatttaaa

63121 ttcattgcct tgtgtcatat attgggttgt tcggaaagtc atttcgtttt tttgatggaa

63181 attatagaca attctgccta tactgacaat agtgctaggt ctgggtatgt cattgtatcc

63241 cattcaagct gtagaagcta ttggcgagtg acaaaacttt attttgtatt tatcagcttc

63301 aaccagcctt caaaacttga aaactcttcg acataaaaat tgccggatgg aaatttagcg

63361 aaccagttct ggcactggcg tactgtcaac acaccttctc cattcacatc ggtctatttc

63421 tttctgactt caacagcatt tttatccttt ctatagcaaa acataaaata agtcgaaatt

63481 tttgcttttc gctctccata tttaaaagga caccaaccaa aaactataac gtagaattaa

63541 ttgaaacttt tcacaaacta gccttgttat atgagctttc aaagtatata ataaataatt

63601 aaacggctta agtgagaatt tgggtgcaaa aaattcaata aatcctctat cgggaaaaaa

63661 cgaaatgact ttccgaacaa cctaatagta aaaaaacgta aatcaatgtc ccctgtctcc

63721 ccatatacgt aaaggtgagc gcccaccgct aaagaggaaa gagctgtgcg ggagttgatc

63781 ggccccgttt tacactttca tacgaaactg catatttgtg tctgcaccga caaagtgaaa

63841 tgagatgtgt caaaaagagt cgagctgctt caaatgagac atcagcgtta tagcattggg

63901 ctcgcttagc tcagttctct gttggtgtgg acgcaagcac atttgaggcc gctcagctca

63961 ccatctcctg ctcattactc ttttgcggtg ggcgcttaac ttaatacttg aactgccgtt

64021 attgcgtttt tgcatttagg gtacaagcag tatagtgccg accacagcgc gagccctgtc

64081 tttactcggc cacgtgccgc gcaccaagat cacctgcgac gcgcccacgc ctgttaattt

64141 aggtatgttc atcatttcca ctacctcaac ctactcccga agtgtcagta caccaggtta

64201 ttgtcctcca cgttaatcta tcctccgtca tgtcagtcgt cacacccttg tcctataaca

64261 tcttttacgc aatccatctc ttcctaggcc tacctaggta cttaagtaca ataaaaccaa

64321 agtccagtta aacccgaatc tcaacattac gcatagtaag tgatatttac aatctgacag

64381 acgttataag gcatcttaga gtagcgtggc catatcatga agctgtatag aaatgacctt

64441 tcatattctt ataaagggta cttttaatat tctctaaagt aacatatatt ttaattacta

64501 aaaaaaatga agtttcatat attatttcct agatatgttc caaccggcgc gccgcgggcg

64561 tggtatagcg aagaatccta aaagaccaaa gatcctccaa ggcccgctcg gcgatgctga

64621 ccccaatgcg tactccgtgc ctatcgcttg cggctccaat caggttactt tataattatt

64681 aaattattgc tattcatgtc attttttaca tacagacgga caaacttatc tgacccggtg

64741 agaaattaga caaaataatc actcattaac gaccaaactg tctttttaca tcttgtatgt

64801 aattttctat acttaagtgg gatttatcca atttttagaa tagttaccgc gcctcttacc

64861 aacggaaatc cttatttacc tacatgcatt aaactttact aatattataa atgccaaagt

64921 tacgatggct gtttgtttgg gtcttggtaa acttttgcac agatgtagaa cagtctgaaa

64981 gacataggga acatatagat tacttattat gttgttagct ccacgcaaac gaagtcgtcg

65041 tcgtgtaatt tttgaaagta ccctcaattt gcaggagttg ccagaattaa atgtgcaact

65101 gttgagctcg gccgcgctgc acggccaagt ggtgcagata caacttgacg ataatatctg

65161 gaataaaaca caataaaaca cacacacaaa cacatagact tggggccctt gcttttataa

65221 cttcacctgt tgtttgttgt attgtttatt tcatggataa agttaggtaa atgatgagtt

65281 gtcaattcct ttataaaatg ttaattttgc cgcgccctga acttgtggcc aggggcacgc

65341 accccggctt gtctcctcta gatccagggc tgaacgtggc cttccgctgt ccaagtctga

65401 caataattgt aaaagttgtg atacttataa ttctctgata acaatgtcac agattacttt

65461 taaactctat tttgatattt aacagtagac aaaataatcc aaaatattgt attcttgcat

65521 tactttggac tgtttttaac aaataacaaa aactatttat aataaaatac tgatattctt

65581 tgtaatgata ttgtaatacc aaaaactacc aaaagtgtcc aattttttta aggtttaaac

65641 aaaaaaatac aataattata agtttagttg ttacataaaa taaactgttt tagtatgtat

65701 ctaagtatta attttaactt tttaaaaaca aataaaaata tataaacgca aaaagtattt

65761 ttcctcttac gaaatatgtc tctgtcttgt cgaagagaat gaaagagatg ataatatata

65821 actttaaact ttcgtgattt ttactaacat tttgttcgtt accttacgac ttcattggac

65881 cgatgtcacg caaaaaaacg gccgcacgcc aaagcaaaaa ctacgttttt accgcgaact

65941 ctgtctaatg aagtcatacg gtaaggaacg ggattcttac cacgatttag ctgcttgagc

66001 tcccgatatt gagtcagccc gtagtcatgg cgcatgcaac tgtgccgata tatcgtgacc

66061 tcacaacaga agactggcgt gaaatggaag caattccgtg tttcgcttgg tgagtgtggt

66121 gccggaggcc taattttagt ccattttccc ttcctatcct tttttataag gaaatgatgg

66181 gaaggggaag tggacatggc gggggagggg acgcatagga agataaatta tcctcttgct

66241 gtgcattgtt gtttcctcct ccgtagacta agggtaggca acgcatctgc aactgcagat

66301 gtctatgggc agtggtcgct tcgctatttc agcgaaacca ggtggccgct tgctcgtttg

66361 ccactttata atataaaaaa aaacgaaatc gtggcaagaa tcccgtttct tacgaacgag

66421 atgataatat ttgtcacggt ggaaacctac agtaggtttc cactgtgatg ttccatagag

66481 ttaagcccta aaataccata gattagtaca ctattgttaa ataatactca gttttaagtt

66541 attagtaatt atctttaatc gaagtaaaga taatgtttat ctgtcattga taagatggga

66601 aacacaccga caatcgacca gattttagta aaaatataac tcgtttttgt tttggacgtc

66661 ttaaggtaat caagtgtttt ttttttaatt tagttaattt gtttattaag tgtaatttag

66721 tattaatttt gtagtgttac atttcaaata acgtttaaat tattcatatc gattaaaata

66781 ttttttttta attatttatc aatgcgaaga aatatgcctc atttcttgtc tcgtaaaaat

66841 gaagttattt caagagtaat tgtcaataag aaaatcacct taactgtgtt tagtttcaat

66901 atgtgtttta tgtttttttt taatatttag tttgattaat tactattcaa taaaaagtta

66961 ttctagtaat ttacgtagta tgtatacctc tgtatgtatt cttaataatt attattttat

67021 tattattaga agattattta ttttatccat aattaaatag attatttttt cttattagta

67081 aaagattttc tccaaacttg gcgatcttgc gccatcccaa cccaatcgca gacgtggagc

67141 tcccttatgt ctttttcaac ctcatcgatc cagcgatacc tgggacggcc aaccgggcgc

67201 cgcccactcg gccgtcccaa atatgcgcgt ttaacactcc gatcatcatc cattctttct

67261 aggtggccga gccagcggag tctgtgcgtc tttatctctc caatgatgtt tggctccgcc

67321 accagctgct caatttccgc attctttctt gtcctccacg tcccatcctc tcgttgcacg

67381 agtcctaaaa tttttcttag gattttgcgt tcggtcacca gatttcttgt cggaggccaa

67441 ggcccacttc gggtcatcgt gtcaccctag ttagttagtt agttagtaaa agattttatc

67501 tctaacgtat ttagtaattc ccctgatatt agtatacaaa aacatagtca tcattcataa

67561 aaagatatat tatcaaacat gtttttaaca gtgtaaacgt aaagcaacaa taacaattgt

67621 ttttcagaga tgcgtctcct accttgtata aagtcatatg acgaaggtgt cgatcattca

67681 aagcagatag acagagagat taaagaatgg attaaaacgt acaatgaggt aagtttataa

67741 ttatttattt taatatcttg ctgttgctcg cgacattgtc cgcgtggaat taaaaaaata

67801 tagcccatgt tactcgggga tagtgtagct atctatgagt gaaacaattt ttgaaatcgg

67861 ccgagtagtt tttgagttac atcaatatat acaaaaaata caaatctttc ctctgtataa

67921 atgacttttt tctagccagt acagtagtat gtatactgta tttctcgtta aatatacact

67981 tagtacaatc ccttagtaaa tatttaacga tttttattct gtatctatac tctaatatta

68041 taaatagtaa tattataaag agagaaactt tgtatttttg tatgtattga aataactcaa

68101 ctattaggcc gatttcaaaa attctttcaa cattagaatg ctacataatc cccaaataac

68161 aaaggctata ttatttaatt cttcgcagac aaagtcgcgc acaacagcta gtttaaaata

68221 aattaacgta agctgtttac ttagagttat agtaactact aggcggtact ttatagtagt

68281 taagtgggca attaaaacaa aagcataata agtgctactt tacatttatc agaataactt

68341 attacagtaa gtgtaggttg atatattttt ctttagaacc taactggcta ccctactacg

68401 agtagacttt tttttttttt ttaaacgtca ggaaatgcat ttaggcacct ctacgccggg

68461 cgtgcaatgg cgtagagagt gtgggactcg ccggccacag aaggtgaccg taatacccac

68521 taaaacctga cggccctcac acgtattacg gcggggtcgt gggcactgtt taacgtccac

68581 gacaactacg agtagactac gttaaccgag aagagaatta cacattggtg tacaaaatca

68641 gttaagattt ttttaaatca cactatgtaa tagtagaacg gataaataaa aataaaataa

68701 aacaccattt attaaccaag aaacaataaa ctattacaat aatgaagtaa ctaaacaaca

68761 aaagaaaata gtttctggtt aaaggggaga ggctcagcta atcctacctc catattacat

68821 ccctccttcc taatatacag gtgtttcaaa tatggatatg cattattttt ttttatcgtg

68881 cattcacgca aaatacgcgc tagtcgtgga gttgtttttg ttctatttta acattatttt

68941 gaaagtcaaa cattcttatt taaaacttat tatacaaatt aaatataata atttcttcga

69001 aatattattc attaaaactt ccttgaaata ggaaacaatt taaggattcg gttaaaattg

69061 attgtttagt ttgatttata atttatcaac gtcggcatcg ttgtataaag tgcttttgat

69121 agcggttttc cgatggtatg ataatcataa cgatctgata gtaaataccg ctttgtgata

69181 atgatgcgtc atgtccatga taatattaat aagcgccgtg atgcaataca tttcaaatac

69241 ttccgcctct tgatcattta taaaggttta gagagagacg ccaggaccta taacctattc

69301 ccaacgattt ttcaacgcgc gctaataaag cgttgacaaa caaatgtatt tccatgttct

69361 gttctacctg ttcacacggt gccgctttgt attatcgcgc atcgttggat tttgaacgct

69421 ggacaaacga aataagagtc attttacagc gatggtatga tcgaagacgt aaagcgtccg

69481 tgtgaatgca ggcttacaaa ttacgcattt attaatgact aactaatgtt cgcgacttca

69541 tacgcgcgga attaaatgat atagcctatg ttactcgggg ataatgtagc tttctaaatg

69601 tgaaagaatt tttaaaatcg gcttagtagt ttttgagtta tttcaataca tacaaaaata

69661 caaatctttc ctctttatga tattagtgcg actacaaatg tctaaaacgt tttaggcaat

69721 caagctgcta ctgttgggca cgggtgaaag cgggaagaca acaataataa aacaaatgaa

69781 gattctccat gtaaaaggat ttagtcacag gtttgtatag tgttgttcca acttaatatc

69841 ggctcggaac ctctcaagag caaaacaata agatatgcaa gcagtgcatc tgagcggccg

69901 acaagattca aatactatgt accacaggtt ctatttcaag acacgttata gcacttatta

69961 ttgtattttt agtgctttgg acccttgtca tcaaagtaaa caaagccttt cattattaca

70021 taaacattgc taagtaattg cctaactaat ctgtttttca atttttcaac agcagtcatc

70081 aacctattca aatcagtgac ctgccttacc cccatagtac ttaaataata cttgaacggt

70141 cccctttttt atgttttcca gtgaaagaac agcaatgatc ccacacatta ggttcaacac

70201 acacgaggca atctatgaga taatacacaa catgcctgtg ctcagtatag gcttgcagaa

70261 tcctaaacac gtgcgcgcgc aagagtatct actgaaggtc ggtcctgaag gacctcggga

70321 gtacactgag gtatttactc ccttaattta aataaaacaa actaatttaa ggtgagttaa

70381 agatttctct ttataagtaa tggctccgag ctaagtaaaa tatggtttgt gtcaaagatg

70441 tgctgtaaaa agggtaaatt tagaaacggc taggaaacta tggaggagta catactgtgc

70501 cgaccctcta tagggacaag ggcatgatga tttttttttt atatgagaag ggggcaaacg

70561 agcagcggat cgcctaagtt aggtaatccg acgcccacgg acagctgcaa cgccagagga

70621 accgcagatg actccggact tatcattatc taagtagggt aaaatgagtc tcaaaggttt

70681 caatagaatt actttttctc ttctttggtg tagttttgtg tatgtttata ccagttttgt

70741 tgatattgac atccttaata gctgtacagt aatgtttttt gtacaacaac aaaaaacata

70801 cagacgtttt gagaaccctc ttttggaagt tgaaatgtac acatgttttg gcagtggaat

70861 ctcagactta cattgtgttt ttcaggagta tttcgataat gtacgggacc tgtggcagga

70921 cggcggagtc agagaatgtt ttagaagagc aaatgaatat caattgattg atagtgctga

70981 atagtaagta tcattttcaa aatacccaca tttttattag agtaggaagc aaacgatctg

71041 ctgacgccta tggacatttg caacacttga ggaatcggca atacgttgct ggcctttaag

71101 aaatagatag gctcgcttct tgagggacaa tactaacttt ctaaaatacc ttccgattcc

71161 taagttctta tatgcgctaa aacaaaatat atcatattaa atacattcta gcgcaatctt

71221 gacattttaa taatttttta actatatttt atatatttta tctatatttt acatgttttt

71281 tatctatttc atgttttttt tattgataag ggggcaaacg agcagcggat caactaaatt

71341 aattggtccg acgcccatgg acatctgcaa cgccagagga accacagatg tgttgccggc

71401 ctctgagtac gctcttttct tgaaggtccc taagtcgtaa acgtttggaa ataccgctga

71461 ggacagagtg agtgatgtga ggacttttat gtattctcag tatatttatg ttttccagtt

71521 tcctagatag aatagacctg gtgtgcaagg agggctacgt gccgtcggac gcggacatcc

71581 tgcgctgccg ccgcaagacc accggcatac agaagattga gttcagagtt aaggtgattt

71641 tgttttatgt attcaaactt tgccggcgta cataaacatt gtttaacctt aacccttaaa

71701 tgcctgattt acgaaagtga ttttttttta atttagttga aaagaatatt atacaacagt

71761 aaaatacgta cagcgaacca ataaaaaaaa ctaatagtaa tgtgaacatt tggtttaaaa

71821 gattggtatc aaaattgata ctctgggtta ttaggggtta agatcagggc ctcgcaatac

71881 aggttgcgcc tagtgggagg acgctgaaca cttcgtccta tgtataacag tttgctaata

71941 aactttttta tttttagcaa tatttttaat gttttttttt gttttaaaaa tatttctaca

72001 agtttcgcag ttttttaatt aaaattatca caaaaatgtc tcgtatctat taataacatt

72061 agttctgtat atccgataaa atagtgatat attgcttttt ttaattctta ggttccaaaa

72121 tcaatgcacg gtggtacaca agacttctgg atgtttgacg tcggcgggca gcggggggag

72181 aggaaaaaat ggatacaggt taattttaca atatttttgg tacaatagat ggcgctgtaa

72241 ctaatatatt gtttttaata tttaacaagt gttcttaaaa atgtgacatc atatcaattt

72301 tatttattta actaatagct gctcgcgaat tcgtccgcgc gggattacat aatatagcct

72361 atgttacacg gggataatgt agctttctaa tggtgaaaga atttttgtaa tcagttcggt

72421 agtttttgag ttttttcaat acatacaaaa atatattttt ttcttcttta taatataagt

72481 atagatataa gtttttattt tgtaggtatt cgaaggtatc cacgcgatct ggttcgtggt

72541 ggcgtgcagt gacttcgatc agacattacg cgaagatagt tcacaaaatc ggttaaaaga

72601 agcactcatt ctctttgaag atgtatggca aagcaggtta ttatttcatc atacttacta

72661 cttttgtata agaattagcc cgcgaggaat taaatactgc ctaataatta tagcatacaa

72721 aatcggtagt aaaagttttt gaattttttt taatacatac aaaaacggat atatttcctc

72781 tttataatat tagtcctact actacttcca atttagttga actaaaaaaa aaactatgac

72841 aactaaaaaa ttctcaaaca agaattttaa tacagaaggt catttcatgc tttttgcagc

72901 aatttatgag aaattactaa ataattctac cattttatct attttccagg tttctcctag

72961 aagctggtat aatagtattc ctcaacaaac aggatctgtt ggagactaag atcaaacaag

73021 gcaggagtat agctccgtac ttccctaagt ataatcattt cgaagcttcc ggcgacgaat

73081 atacgagaac aaagttattt attagaagtc tatttgtggt aagacacctt ttctatagcc

73141 aaagatattt ttaatatttt ttctttgctt ataacagtaa atattttgct tatctgtggt

73201 atttatcaat gcttcaaata taagcaatac aaacattgca aaataaaata tgaaccatac

73261 ttattttcat acaaaataat aaaaaatatt tctattctta tcaattagct gtctcccaca

73321 aaacttagta agtagcccat gtgttctaga tccttctaca tccataccaa atctcaccga

73381 aatccgttaa ttcgttctgt agatacattc aaacggacat ccatccaaac ttcgcattta

73441 tattattaga ctagctgttg ttcgcgactt catccgcatg gattaaaaaa atatagccta

73501 tgttactcgg ggatgtagct ttctaatggt gaaagcttta ttgatatcag accagtagtt

73561 tttgagttat ttcaatatat ataaaaacac aaatcttttt attataatag tatagattag

73621 tagtagcgtt tcgatttggt agatatttag ttatttgtag aatttaacaa cgaagaagcg

73681 tgaacgtcgt cacatcatag cgtcagcgga gaggttcgtt gtactggagg cgagtcggtc

73741 ccgcgaatgc tacttccatt tcaccaccgc aactgacacg cacaacgtgc gcaccgtctt

73801 ccgcgacgta catcatatga tacttactca catactcagc aatatcggcg tctactgaca

73861 gtctattagt ctgacaccga cttggtctac tgaaatactg ttaacaccga ctcggactat

73921 caacaatgta ttggtctact gacataaacc cggtctactg gtctactgac ctactattaa

73981 caccgagtcc gtctactgac atcgatcaaa tctattggtc gactaacact gacttggtct

74041 actggtatac tgacactgaa ttggtctact gacttgctat tattacagac tcggtccact

74101 gacacaaacc tggtctactg acaccaacta gatctacttg tctactgaaa ccgacttgat

74161 caactggtct actgacaccg acttattcta ccgatccgac atattggtct gctgacaccg

74221 actacgactt ttgcactgtc tactgatcta ctgatatctt gcactggcca tttacatatg

74281 taaaataaaa atctctgact agtttacttc tcccttattc ttaaatctga ttgttaatta

74341 tagaaattaa ttataattag tgataattta agtacggtat aattttctat tgtaatttag

74401 ttttataaat gtgtgtttga atgttatttt ataatagata gaatagtact gaaaacgatc

74461 gctcactttt agcaatataa aaagatgacg tagtttgaaa atttagttta agtaaataaa

74521 atatttttgt acaataaaat aaacatttta ctaattcctt tgaatttatt tttgataaaa

74581 ctaatggtac ccttaaatct ccttcaaaaa tgaggaacac tcaaataaat catgtagtat

74641 tttttgttta aagtgtctat tttttgacat ttattaacat catgaattta caaatatctt

74701 aaagttaaaa agtcatacaa cacatcacta acaaataaat ttaaaatagc aatataattt

74761 aaaaaaaaat agtaaatggt ttttaatgtg tacggtcagg tgcttcctta ctgtcatgat

74821 atgctttaat agctttacta ccctcaatca tggcgtggtt agctaattca ccaggaacta

74881 ataactttat agcagttctt acttctcctc cactcaatgt attcctcttg gagtggagga

74941 ccagcctccc agcttcttca gctatcttct ctatcatatc gttgacgaag ttattcataa

75001 tcagcataga tttcttcgaa ataccaatat tcctgtcttc gacaacagat ttcaacactt

75061 tgtagatgta actcgcgaaa ttacgactgt ttttctttga ttttttattt ttcaatatcg

75121 gtttctcgat cggtttgtcc atggtcttta gagcgttctt cggttttttc acgggcgcca

75181 ttttgtattt tgaacggttt ttcataagta taaattatct tgcctgctat ttataattta

75241 aactacaatg taactgatga gggttttaaa cagttagtaa ttaaaaaata gtagtaggta

75301 gtagtagttt tttttatttg atttattttt ataagaggct ttgtcgccaa aagcgaccat

75361 atcgccccat aaatctaata cttaattcta tacttaaata actataaatg atatcttaga

75421 ctaatatcgg ccaacttaag caccatcctg gcctcgtccg aagacgggta ggccagaata

75481 gtgtttatcc gaccgacgac cgacaaacga ctttcttaaa ataactattc ctaagatgtt

75541 cgctccggac acattcttag tagtagttgt taataaggtt ttgtacatat acggcgattt

75601 tcttattacg agatagtttt aataatgaag taacgaaata aatagttcat atttaaatta

75661 ttctttaaat taactttagg attaccttgt gattatttgt ttatcagcac ttttaactat

75721 agttatgtat ttttagggtc tataaaacaa gtcttaagat taggtcaatt taattaatcc

75781 ttaagtataa tttagttcat tatttacaca gacataataa agttaaacat aaatttgaat

75841 caatcagtgt tctttgctat cattatacgc tttaagcgct tttgctcctt cgactaaagc

75901 gtgttttgct aactctcctg ggacaagaag tttgatggct gtacggactt cggcactgcc

75961 caaagtactc ctttttgaat ggacgacaag tctcccggcc tcttcagcta tcttctcgat

76021 catgtcattc acgaaattat tcataatcag catagacttc ttagatatac cgaagttttc

76081 ttttaaaata ctcctcaata atttatagat gtaaatagaa aagctatgat aattcttttt

76141 tttcatcttt gtttttacaa tcggtttttc gatcggtttc tccatcgatt tcaataaatt

76201 cttcggtttt ttaactggcg ccattttgga ttttaaaaca gagtcgggta ttcgcagtca

76261 atcgtttttt ggcgtagttt ttgggtatat tttattgaat ggtattgtag ttaaaggggg

76321 ttataagacg cgtataaacc aatttttttc ccaaacattt taatcttttt ttatttgtta

76381 ttaaatatcg gtcatttcaa tgtggttcag attcattaaa cgtaagtaca taattttata

76441 atatgcatta agacatcgac gatttcaaag taaatgcatt taatatcatt attctaagta

76501 cccgaggggt ataaaccgac tatcaaatta ttgtcactaa tttaaaggta gtaaaaataa

76561 aacatacata cttcaacctc gaattaccca caaatatctt tattatcagt ataaaagcga

76621 acgcgacgaa atatagtacg acactatctg tctgtcatcc gtgcgcgcca aaaatgacag

76681 gtcgaggcaa aggcggcaaa ggtctcggga agggaggcgc taaacgtcat cgaaaagtac

76741 ttcgagataa tattcaaggt atcacaaaac ctgcgatacg aagattagca agacgaggcg

76801 gagtcaagag aatttccggt ttaatttatg aagaaacgag aggagttttg aaagttttcc

76861 ttgaaaatgt aattcgtgac gccgttactt acaccgaaca cgcgaagaga aagactgtga

76921 cagcgatgga cgtcgtgtac gctttaaagc ggcaaggcag gactttgtac ggttttgggg

76981 gttaagttaa taaataaaat acttaaataa atatatgaat tgaacttgaa gaaaagacaa

77041 ggttctttta agaaccgcaa aattatcttg gtttctattg agatatgtga tggaggcgtc

77101 agttgttttt tagttgcatt atgaaattaa tgttttaatt gagtttttgt aaagttaccg

77161 ataaaactaa tggttacgcc cttaataggt tatgaatatg agaactagaa ctttaaatta

77221 gaaattaatt aaaaaccaat taattagaaa ccacaaatag cagcggtcca aaaattagct

77281 atacaagact gagatttttt tttgtttttt tttttaagta tattatgcaa agaggtaata

77341 tacatagagg gaccgctcca tagtaatttg acgttaaaat gcgtgcctcg caagatatta

77401 ttaaaaaaaa atcttgcagg tatcatgaaa aaaattccca agattgggaa aatgtgtgtt

77461 ttttttttaa agtaaaataa tagaataaac taaaacattc attaaaatgt taattatcgt

77521 ttagcattat tattatttta tttaaaacaa ttcaaatcca aacaattttt aacaaagact

77581 atttttgttt tatagttgtt ataaatttgt aattagcgtt ttaaaaatgt ctcgattaat

77641 tacaatatta aactttagta aatactatga aataaaattt aaatatgaga aaagaggtta

77701 cattcgttcg actttttatg tcggcaaatg taatgtaaat gtcaacacta ggggaaactt

77761 ccctacgtga gacctcccga ggtcaaagaa aatgcctcac ttttaggctt cgaatgtttc

77821 atctatagta taatatatct tttatattat gtttaaatcc cctaccactt actctcagta

77881 agatagtgtg gccaaattat ataaagccaa acctaaaaaa atactcaact tttacgattg

77941 ttcttaacaa caagatttga catcaaaaat ggcgcgaacc aagcagacag ctcgcaagtc

78001 caccggcggg aaggcgccaa gaaaacaact agcgacgaaa gctgctagaa aaagcgctcc

78061 agcgacgggg ggagtgaaaa aaccgcatag atacagacct ggtacggtcg cattacggga

78121 aattagacgc tatcagaaga gcacagagtt gttaataagg aaattaccgt ttcaacgtct

78181 tgttagagaa atagctcaag attttaaaac cgatttaagg tttcaaagtt cagcggtaat

78241 ggcgctgcaa gaagccagcg aagcttatct tgtgggactc tttgaagata caaacctttg

78301 tgcaatccat gccaagagag tcacaataat gccgaaggat attcagcttg caagaagagt

78361 gcgtggcgaa cgcgcttaat tcacatttgt tataataaat actaataaaa atatattaaa

78421 atgttataga taattatggc ttgtaattaa acgataacca aaaataataa atttgacctc

78481 caaaataaat atttattatc agcaaaaaaa agtaaatcat cacctaaatt cgatccacat

78541 tttatataaa aactaccacc aaaatatttg tagtctttca atcaaaatag aaaaactatc

78601 accaaaaaaa tcattttgcc atcaaaaata ataaagttgc acctaattta taacctcaat

78661 ttaatgcaaa atttcactca aatattttac atatttttgt gctaaaatag taaaatataa

78721 ccaatttaaa ttattgttca cctattttat ttttttagta tctattttag ctgacgtatg

78781 tgggagtgac ataaggggcg tttcacgcat aacctagact ttgatttaat tttaaatatc

78841 ctaaacttag cccaacccat acaggttttt gttgcagttt attactttta tcggtaacaa

78901 ggttgcagta ttaatactta ataaccgtta aatgtgacca attgattact tttagctctt

78961 attttgctga ttatttattt atttatttat ttatttaaat tttatttgat ctgcaatgaa

79021 agttactaaa caacaatgat ttcatatggt ttggataaca ataatggtta aaaattatga

79081 ataatattca attaattacc attagcgtaa cacatatttg gattaagcgt atacgaacat

79141 aggacctgaa aagacctata tcattatttt ttatgaaggt tagtttttag tttataattc

79201 ttgtacgtta ggtatcattc tggggagtct ccagacgggc gactatgtgg gcagctttcg

79261 gatgactgtt ggcacacgcc agtcctgctg gatggggcgt gcaaggcgag agtccacctt

79321 gtgcttgatg tttgttcgtt gagacgaaca attcttaatg tttataattc gattgatagt

79381 tttttttata acttacctgt ttgttttttt tattttattc aacatgttct aaacataacc

79441 ttacctatgc agttggtacg cgtttttaat agacatgaat ttaaattaaa cgatgtaaaa

79501 caatcttcat aattagtaaa gtaaatagaa aaatttgtat attagtgaga tctgcatatt

79561 ttttacttta gaagcacttc agtcaaccat caagcgcaac gtcacagctc acgaataaca

79621 aagagcggcg cagagagtgc gtccgtgccc tgcagtaacg gacggataaa atataatata

79681 atataatttg gtatcaatat agtttatttg gttatatgca tacattttta ggttgatttg

79741 caatatattt gggtataaaa gcacaaaaat tagatgtttg aaattaggtg ctaatttatc

79801 aattttgatg tgaaaatatt ttttctgaag gaagcgtgat ttaaataggt agatgaattt

79861 taatttagag catcatatgt ttggtacagt tttatttttt ttgttcttat tatatttgtt

79921 ttgggtaaat caatttggtg ttacgtttat tttttttggt ggtcgtttta tattttcccg

79981 ataattatta catcgacaaa gcccttttca gggctgaaat taatctatgt ttcggtagaa

80041 tgtgaacaat atttaaaaat aatttattag aatcacctta tttgataggg aatccgtaaa

80101 taagaggata ataataaatt ataaaaacaa tacctaccag acatctgaca agccgtgttg

80161 gacttaccca tggtcatgaa tcaaatcacc atttgggttt atttactgtt tattttgtta

80221 tgtaataaaa ttcattaatt atttattaaa acatttattg aaaaaaacat tttatataat

80281 tctgaaacat aaatagcaac ccatcaaggc gttgtttaaa aattacattt gtagcattac

80341 atgttagcag tcattgctct tgggacatat tcttcgtggt cttcttgggc agtagatcag

80401 gatgtatcgc aggtatcacc ccgccctgtg acacgaccac tccgctcagc atctggttga

80461 gctcgtcatc atttcgtatc gcgaatagga tgtgtcttgg tatgaccctg gacttcttgt

80521 tctgtttagc agcttccgcg gccagttcca gtatttctgc tgctagatat tcgacagcag

80581 ctgccaggta tatcggagct ccgcttccta ttctttgtgc gtagttgcca gattttaata

80641 tcttatggat tctcccaact gggaacgtga tgtttgcacg agaggaacgc gatttagctt

80701 tgctcctcgg tttcattgtt tttcttcgtt tgtcagctct ctatcacttg tgtactctgc

80761 tggaatttga catatttaga ggggtttagg tagctggggg tttaaaatgt gttttagtta

80821 catttatccg aaagtttaca tagtttctct tgtactttta tatacttagg tggaaataat

80881 gtcaacgatt tctctgacga aaattgcaaa gataaaaaac tataaaaaca ataagtcaag

80941 gtcttaatct attttttcat acacttatta tgtcgaagtt aaataatgct ggtctataac

81001 gaccggtgcg caggaagtta tcatcaacat ttttttttag acttgataaa aatttacttt

81061 ggtacacata cctacacaaa tccaaacgat ctttaatatg cgcattggga aaaacatagc

81121 ataaaagggt attccgtgta taaattcagt aattccctat gaacatgaaa gaacaaacta

81181 gttaaatctt taaactctgt cctcccttca agtgtgaagc gtattgccaa atgttatgta

81241 cccctgtccg cagctgtaca cgcagctgcg agagaccgtt acgcgcggtg agcacgtgcc

81301 ttgccctacg cccttcccca cctcgtctct ctaatattat tattaaaaac gtttcaagta

81361 cattttttgt ttatttacac ttagttataa tcatagacaa ttttttactt taacattgca

81421 ttgcagtatt cttaactaaa agttactttc taaatattca atcgatcggg tatgcctata

81481 tgtacttatg aaaatattca attttaaatt catgttattg cgtcctatag tacctggtat

81541 tcaaatataa gaaacagctt ttacattaat acattatttt cagttcatat ttagcaagta

81601 tgataacatt gaactatagt ttaacttaaa agaaaattgt taacagaaat tcatccactt

81661 taattcaagt caacatcgac cgtttcccat tgtcggaatt cattcagtaa ttgtgcaaac

81721 gtaaattcat ctctgtatta aaatgtgttt tgtaatttaa gccttttgat taacaattaa

81781 ggcttaaaat gctttgaacg ttatatttaa atttggaatg cgtagggcgc gtgctcgtct

81841 gtttactagg ggttgcgttt gcgccatttc cgaagccagt ttttttttca acgttatccc

81901 ctacgtcagc tcagttgacg gttgatggtg gaacagttag gacgcagtct cgctaataaa

81961 atataacaga atttaattaa atttctagaa gctattttaa agttttgtgt gttttaagtg

82021 tgtgaaatat tttttttcat agttgtttct caactataac aaatgtagta aaacctgtgt

82081 aaactcaagt taaacgagga tggaaaccgg aacgccgaga cggttccgct ccaagacgca

82141 cccttaagac aggaagaggt cagccatcct gtttataatt caagtctaga cgccggggaa

82201 agtgtacgct tacaatatgg attcgccaaa aataatttac aagtattact caaatcccgc

82261 attctgctcc caaagcgaag ttgtatttgc acaaaaatca tgtgtcacca agctggtcac

82321 gccgagtcga aggatgccac cgttaggagg gaattcaccg aggaagaaag aaatcgacaa

82381 cagaatagta agaactgata tatcagacaa tcctctgcga ttgacgccgg ctggaagaat

82441 gcgggatgaa ccgccggagc tcccgccgaa acctccaaag tatcaacgcc agttccaaag

82501 gcaagcttca ttaatatgca agccaacaag acagatcgta cgatgtagga cacgcagtga

82561 ggatctagaa atggcacagt taagacaaac ccagacgaac aaatacgaaa gaaacgccga

82621 gagcgaggac ggtctagaag acagcagact gaaacacagg tatgaagtca ttagagatat

82681 agaggatggc ttagattact caccaacaaa gaagagaatt atagaagcgg atgcgagtga

82741 aatcgaagag tacaatgtag acgggcctga tgaaaatgaa atgaaagaaa taccaacgga

82801 aatagtgaaa acggttaacg gcaagacgca caggtacgcc attgttcctt cagatgatga

82861 ggaaccgagg aaaatgaatg tcaccttctc atcaccgatc atgtcacaga agaatctaat

82921 cgcgactcag aaactccacg agctgctgtc gacgccgaga aagttgaaga gttacgctag

82981 tcaaccatct gttagaataa caccaaataa acaaacaagt ccgaacgtat acgtcgatac

83041 tccgaagagg tttatatcga gcacgccgac gcaaggagtt actccatcca agtcctgtgc

83101 caatttatct ataatgagaa gcggaacatc acccatttca ccaaaagttc cacagaaatt

83161 gaactacggc gcacctgaag aattcgaaag acaggacgtg ttcgttacta gtttcagaga

83221 caagagtcgt gatagaagct tcgatatgga cagaagagaa gcgagtagag aaagcagaag

83281 aaactacgac actagaggaa gagatagtca aagaagagat aaaactaccg cgatcattat

83341 gcccaggtac atatatttag tgcaaagctc tataaatgaa ttcccgcctt tttatccact

83401 cggtctacac tcttaattta actctttata aaacaccgaa acacttttta ctttcatcaa

83461 aaacaataaa ttattcacat agtaaccatt agatgcgcac gcgcaccttg tcaataatta

83521 aaggcagatg gcgtaacagg tgttttttgt tagtaattta agagaattac gaactcaaca

83581 tatcgtcaca cagttgctta gtagaggtat actcagtcaa ttgttatttt ttattttttt

83641 atttaattgt cactttataa cacagggtag cgccacagag tccgtcagta tactcagaag

83701 agacgtacaa atctctgtcg acactgaaag ttgtgaacag ttcctttgcc tcgctcacca

83761 ttgccgctct tatgttgacg ttatgcggcg gtctcactac tggactgagc ttctatatga

83821 tgtataccgt aagtatatat gtactcaaac tcgtttttcc taatcgctat gttccaaagg

83881 cctggaactt agcttagcaa ttcacaataa tggatatacc atgttcgttc tcactaacaa

83941 gtccttagcg gttataatca tgcaatatat tttcaggtgg gtcgccgcta ctacctcgat

84001 ttcggagtcc tgtccggctt cacgtgtttc ctgctaggaa tgctcggttt aaggtcgcgc

84061 aggaatcagt tgctgccgaa caggaattat atatctggta aatattgtta gcactagttc

84121 gttgctgaat aggcatactt aggtagattt attttacgat caggtacact gtgagaaatg

84181 attttatgac caaataaaac tagaatggcg ggaagacgtg cccgcatgct tttcaaccgc

84241 aacgcttaat gtgcacacta acgttttatt atattgtctg cattactaac ttaaagtttt

84301 atatttttac ttaagtaatt attacttact tacttaaatt attgcttatt tttaacagtt

84361 ttttttgttt caggttacat agtgctgtcc tcattctctc ttctcagtgc tttcggtctt

84421 ttaattttat tatctgtcca acccctgccc ggtacagccc ttaacgatat tacttctgga

84481 gctgtgtgca gtattagcat tctctcatta tctctcgcta ccataggtgt tttagcttca

84541 tattgttgcg caagagatcc acccgataat agagtgggca caacgaggtg gtattaaaac

84601 tttttagtgt tttgacattt cagttggctg cacgattcca atgacctttg cctttttttg

84661 aaaagaacaa tgtcgtttta attttaacga atttgtgaca ttcgtacaaa taaagttaaa

84721 aaaatgttag ttttaaaata aaattgtata ttatttaagt ataggtaggt atttattgca

84781 tagaatttct tgtattaagt tttaaggaaa aataattaat taattaatgc atttactatc

84841 tgaaatttta agatgcacta gtataatgaa ccaaagaatg gaatttaatt tatatgctta

84901 attttctgcg tcatagttat gtaatcatca ggtaaatgat atctagcaaa taggtcatat

84961 cgacgttaaa tactttaata agaaatgtat ctttgtatgt agatattttg aagtcgcaaa

85021 acccaatgaa ttatattttt atactataaa cctagggctt cattagacca agttcgcgaa

85081 aaaaacggaa attttgtttc ggcctttttt gcgtgacatc actgtaatga agccctaggg

85141 caagtgccaa aatgtactag tgtacatttc tgtgttgtac aagaatttcg gcagtagtct

85201 gccgaaattc atagaaagaa taaggttctt tcttagtcca tgctgtgtaa aaacaaattg

85261 tgatccctct cattattaaa aaaaaaacag atagttaagt tttgtatgat gcaagtttcg

85321 gcagttggaa cactcaaaga ttcataattt tatgaatgtt aaaatctgta ataaaaatag

85381 caaaactatt tgattgtttc ttttattagc ggtacccttg acacctaaat agcgagacgg

85441 ttgaggttca aaatcgagga gcttgtaact aaacatgcaa cattgtacgc ttgaccgacc

85501 attcaacgga aggacagcaa cctgttcatg aaagcagtga tgctaaaaag gtttaccgac

85561 ttatttttag tggatcgtta taaaataagt aaaatagagt gaaaatctag tttaaaaatt

85621 ttctttgtct ttccgcaagc cgcgttgtct ccagatgtct cagccggaca ctgccgattt

85681 ttacgggact tccaatgaaa ggtatatgta ctgttatagg ccactatttt ttggtcctga

85741 ttctgtgttg acgaagccgc aggcaagcgc tcgcattttt tttaaattat tgttctgaat

85801 ggaatatttt agaagtctgc aagtattgga acgcaaaact tttttgaaaa ctataaacgt

85861 cacttatcta ttgtcatttt gtcagttgtc aattctaatt ctataataca agttaaagtg

85921 tatttattta attattgttc tagtttgata acataattaa aagtgtttga taacatgtta

85981 tgatcgaagt acagtatgct taaacaatag ttgttttact gcctttaata gtctataaat

86041 aagtgattgt gaaatcagta atgtttttca attaataaca aataatcaga gtaagtcttt

86101 gtttgcattt ccttacaaat acaatcaaac acaccttttt tcatagaata ttaacaaatc

86161 ttttaaaaca agtttagaca gtcaagaatt ctggaaataa gtctcaagag agtaattaaa

86221 acttttttca tataaaaata aagatttatt acaaaataac ttttatctca aaccacgcgt

86281 atttagtact ctaaagtgcg cgttatttag ttcaaaactt aattttatta attgttttgt

86341 tttgtgaatc aacatgaaac atttgtgatt agcctcacct tttggttaca ttgcaaattg

86401 gttgttaaaa tatgtttcgt aatatgtcaa ggcttttata ttaatatagc tctatatata

86461 aaaatggttt aattagctaa gtcattcgct aagagaaaga tgagaaaggg agcttactat

86521 ttgttatttt tttaattaaa ctttacaatt aaaagtattt ttcaaggtta tctattgttt

86581 attaccaatt ataatgaaaa agataacagt gaacaatata gttttaaacc attgtcttgt

86641 acacatttct taatagtaaa ataaacaata cattaataca gtcaaacctg ggcaagcaag

86701 aaacctccat aagcgagaca aatattgcgg tctcttaaag tcttgcttat tcatgttgac

86761 tgtatttgaa atgtaataag aatatattcc agggtcacaa tggctgacca tcacatcagg

86821 gaagcagctg aatctctatt ggagagcgtc tccaatccac agtatagaaa tgtttgtaag

86881 tagttttaca aagattaaac caaaaattgt atatataccc acctgatact aatttatgtg

86941 gtagaacact agtatgtgtt aacatcaaaa tctgttgagc cattattgaa tattccttct

87001 aacaaccatc catacaaact tttgcattaa tattggtttc attcgctatt tgagcatttg

87061 ctcttttgct atattacaat aacaaatagt atgatgagtg tttctttata cagggtgtcc

87121 caaaattcaa cgataagcca gtgccagtgc atcagcagat cggtacctgc atagggaaaa

87181 ttaataaaaa aaatctaact cttttagttt aaaagttatg ttaacttatt tgaatttaat

87241 tgctctttga tacccgaccc cattcaaata cccactgtct cttgagctat aaagatatcc

87301 ctgctttttt atttgtaatg ggtagcttac taacatagct aatagttgca gttgaaaacg

87361 ggacctaaat atctgtagtt taagtacaaa aaaataatta ctttaactgt ttgcttgaaa

87421 ttttccaata tttcaacttt tcatcaactt tgtcatgcta ccgtgaaaaa aaaatgtatg

87481 gagggtaccg tggcatgttg ttacaaaagt tggcgcatct catcaagatt ctaaaatggt

87541 acaatgttat tgtagaaaca tgcaaaaacc ctgttaatat ttttttcaag tactgaaaag

87601 gattattttg catagtttca aagaacttta ctaactgttt aaacaagttt taagttacag

87661 atttgtgtat aattaccttt atttacacgt tttgaggtac gagcgtgtta gaaacctaat

87721 attaatacga cggagtgcaa catcatcatc accatcacaa tcacagtaga gaagaaacaa

87781 aaactaatta tttcgattaa attctgtcta gttggtggtg gtgatgctca tgatggttta

87841 aatgaaactc ataattgtta ttttatttca ttaaagttaa gaaacgaagt accccagggg

87901 gtccacttat tatgacttta ctaccggttt cactccgact tatcaatagt atctaacacg

87961 ctcgtacccc aacacgtgta aataaaggta attatacaca aatctgtaac ttaaaacttg

88021 tgtaaacact tagtaaagtt ctttgaaact atgcaaaata atccttttca gtacttgaaa

88081 aaaatattaa cagggttctt gcatgtttct accataacat tgtaccattt tagaatcttg

88141 atgagatgcg ccaacttttg taacaacatg ccacggtacc ctccatacat ttatttttca

88201 cggcagcatg acaaagttga tgaaaagtta aaatattcga aaatttcaag caaacagtta

88261 aagtaattat ttttttgtac ctaaactaca gatatttagg taccgttttc gactgcaatt

88321 attagctatg ttactaagct acccattaca aataaaaaag cgaggatatc tttatagctc

88381 aagagacagt gggtatttga atggggtcgg gtatcaaaga gcaattaaat tcaaataagt

88441 taacataact tttaaaccaa aagagttaga ttttttttat taattttccc tatgcaggta

88501 ccgatctggt gatgcactgg cactggctta tcgttgaatt ttgggacacc ctgtataata

88561 taacctattg aacagattaa gaaactatag tacctttctt ttagatgtca gagcccacag

88621 tatacaaata aaagaaagtt aaataggttt tatgaaattt gtagttggct atgactgcta

88681 gattttatat gtttgctctt gattataaaa tacatacaaa gcaaaataga accttttaat

88741 gatggaataa agttcaattt tgtttgtgat aatgaatgca agtttaatct aatattctat

88801 agaattattt tagcttcact tgtaccatat tgtttttata tcatttgtta ttttttttta

88861 ttaacctgac acaattattt tttctttaaa agacatgtaa tttattatca ataaattatt

88921 tgtatttata tttatgtttg atctttcagc tcaaaatcat tcagtgaacc taacaactga

88981 agacctctta gctgggtacc gaccgtacgg caggatgtcc tccgtgagaa ggttcttctg

89041 tctttttgtc acattcgatc tcctcttcac aagtctcatg tggctgatat gtgttatggt

89101 aagggtaatt ttttgcttac tagctgttgt ctgcgactcc ttctgcacta actaatgaac

89161 ttagtaagta ccctataccc tacagtagaa cttctaccag attatgtttt ataattaaac

89221 ccagcaagac gttgcttgcg attttgtctg cgcagaatta aaaatatagt ctatcttatt

89281 tggaaatcat atagctttct aacagtgaaa gaattgttgt aattgcccca gtagttcttg

89341 agttatttca atacatacaa aaatacttct tctatccttc caataaggaa ggatgagaag

89401 gggaagtaga tttttccggg ggaggggaca cataggaaga ggaattatcc tcttgctgtg

89461 agtctcctcc gtagattaag ggtgggtaac tcatctgcaa ttcccgatgt caatgggcaa

89521 cggtcgcttc gctatttcag cgaatccaga tgaccgatag ctcgtttgcg actttataat

89581 ataaaaaaaa caaaacaatt ttataggttt actcacataa aaaaaatcca tctctttcta

89641 ctattctcta tttaatgttt gtttgcagat gaagggagag tcattagtga tgatattcaa

89701 cagagaaata gtacattaca atataaagat atctttagtt gacatagtgc tggttgctgt

89761 actgcggttt ctattactta tactgttcta tgctgcattt tatattaata attggagtgt

89821 tattgcggtg agtatacttt ttaattatct tgttcagact tttggtcgaa ttccgaaacg

89881 acacatggca gcgtcttaaa taagaccaca agattccaaa ttctatatag aaattttcat

89941 atctacttga tgtttattag atttaaagca cccctataaa ccaaataaga taaatcgagt

90001 gggcaaaagt catatcaaaa tacgacttca cttaggtcat atttgggttc agaaataaga

90061 cccaagcaaa tgtgacgtat catattatac tatagtggcg ctccaagcgt attttcggtg

90121 aactaactac tcatatgtcc cagccaaagg cttaagacat gatgttattg gttttaattg

90181 taatgtgatg cacacacaac atacctatga cgtgatctaa ggcatttttt gtccttcacc

90241 gtatatgtca attctatatc tttctttttc tatgttttac atattttcta acagaactaa

90301 acagaatagc agcgtttgtc acactgggcc agagatcctt gtttgactat actttaagat

90361 tcgatacata atttggctgt tggtcaattt acttattaaa gtattcaatt attttgttgt

90421 tagatatata tacaatagtt ttaattttat ttcagctgtc gaccggtggg acttgtgcct

90481 ttttaatagc aaaagtgttc gtttttgatg taagtatgag gtaaataatt cgcctttaaa

90541 ttatattatt tgatctgtat aatacaaata aaaaaatata tattttacgg gtattttccg

90601 gagggccgat cgggtcaata ggaaacaaac cattatactt gtgatcttta aaatcaaact

90661 tgacattgat gacttattcc cattcacacg agcgcttttt taaggcgcgc taaaaaagct

90721 ttccaacaat ctgttccaac ggtgacgctt tgaattttta atcgcgttgt attttaagag

90781 gtggacgaac gaaacaagag tcatttttat gaacgagggc gttaaagcgc caacgctaaa

90841 gttaaaaaag cgtacgtctg aattcattat tacaaagtac aataatctag aacttttatg

90901 tatagttatt aagttacgaa cctggacaag agagtccaag tgaccgcgat atttgtctcg

90961 tttacagagg tttctctata atccgaattt ctcgcttacg gaggtagctc gtcgggacca

91021 gagaatgact cgcttacaga ggtttctcgg ttatccagat ttgactgtat ttgatagttt

91081 caaagtgtaa ttgtagtggc cgaacgcatc ccagccggtg taccaagtgt tcctcatcct

91141 gacgtcgttc acgctggcgt ggggcgaagc ctggttcctc gacttcaggg tgctgccgct

91201 ggagctgggg gccagtagtc agtactacct ttattcactt ttctatctaa gtatagctat

91261 gtcacgtgac ttcgtctgcg agtaaattaa aaaaatatag cctagtaaat gtggaacaaa

91321 aaaacaacat agtaagtgac ctatttgttt ttcagactat attctacatt cctactgaat

91381 tttaccaaga tcctttaagt agttttggag gtaccttcaa atatccatcc atacttttgc

91441 attaataata ttagtaaaat taaaattact gtgtggcaat aatttacaca aaaagtcacc

91501 tatagtaacc caattaacta tcacctagta cacaataaaa acggagagtt tgcttcggag

91561 taaggccgtt tcttgcctga catcggtttt atgaagccgt agggtaatag tagttaaagt

91621 taccgattcc taccaggtca tcgaccatca gagcgtaccc cgctgctgca ggcgaggcca

91681 gcgccgcgtt ccgcctacgc tgagtccact gtcaactggt tctcacccgt agagaccccg

91741 gaggccagtc ccaggccccg cctgcccggg gaacaggtta tactgacgca ggagttggtg

91801 agttatttac taagttatat ttatactagc tgttgcgcgg gacttcttcc gcgtggaatt

91861 aaaatgatag cctatgttcc tcggggataa cgtagctttc taatggtgaa agaatttttg

91921 aaatcggcct agtacttgag ttatttcaat acaaacaaaa atacaaagtt tctctcttta

91981 taatattagt atagattaag tctgtttgtc tgtgtaataa atgtagataa tcttaggtca

92041 ataacactcg ttttgatatt aattctatac taatataaag aagaaaggtt cgtatttctg

92101 tgtattgaaa taactcgaaa gactcctcgg tcgtcgcata atgtagacat ttggcaacat

92161 acagttttat aattattttt atacagatct aatatttata actaaaatat gataataacg

92221 tacataataa gagttgagaa tatatttata tttaaagaaa tagcgctcgt atatcaatga

92281 atacaaaatt acggagacgc ttcttttaac tataaacttg tcgaccgtca aataatgtga

92341 aacgtggtat gtattgggca atgatataca tttaagggtt aagctaataa aattatattt

92401 ctgattgaga gcaggtttat tttttcatag attttggaat ggattcagtt taatagtgaa

92461 aatagcgcca tctagattgc gtcatgataa aatgtgtttt aagaatgata tagttatcag

92521 cgctgacgct tgaagttgat tatatttgtc cgtaaagtct tgttcgcaca cagataatat

92581 tatagtcgag tagcaagtat caagtatccg agcagaggat ttactaatct agtctactaa

92641 aatgttgctg ttcggacacg tttagttact aaattacttg ttaaaatact atcctggtgc

92701 gtaaaaggtt tcaataaaac taatcttagt gtagattatt caattctgag tacggcatta

92761 ataccgaact aagattcgaa aagtatatta atctataagc gtacagtcca acctggataa

92821 gcgagagtcc gagaaccgcg atatttgtct cgcttacagt ggtttctttc taatcagagt

92881 ttttcaccta tgggggtaac ttgtcggaac cccggggggg ctacccgatg tacttttcga

92941 ttggcgatat cgctgatgga atcgacaaaa atgtatgaaa ctgacattga cattcgataa

93001 gtcatgtgct tgtcacgtgt gagatatcga acgtctttgc cagtttcata catttttgac

93061 gttcgctatg gaatcgcttc gtagcgaatg tactttgagt aaccccctga gaatgagtct

93121 cgcttatcca ggtgtgactg tatataatat tttttattca cattatatta gcatacttta

93181 tacatataca tacttcttat cgttgcattt atcttaatta caagattaaa cgtagcttat

93241 ctaagaataa gatataatta atattgcaca ttattttacg tatttcccgc gatttttaaa

93301 gatttacata tattctgatt ttatttcctt gttgtttgta caaaattgaa acggcagttc

93361 ggcttataca gggtgtcccg taagtcgacg ccaaaactga aacaggtgat agaggaagtc

93421 ctaagagaca ctagaaaaat attaaaaaaa atctatgtct aatagtttat gaactatagc

93481 atgttctaaa ttttcgaaaa aaagtacacc ctgtgttggt tattagcctg cagtagctat

93541 aactctttac ttttttaaat ttttattttt cttgtctaat cttaaatagt gtttgtataa

93601 cctattgttt aaaattataa gctaactgct acagattaga agataatgcc acttttatcc

93661 gaactataac ggaacgatta aatattcgcc caactttaaa tgactgtcct gaaaaaaaaa

93721 atgtaccaac gctagtatgg cacgtcgcgc gaaaagttgg cgcatttaat aaggattcta

93781 aaatggtata gcacttgcta cattatttct tacataacag ggaaaaaaat atttttcttc

93841 aaaaatcgca atttttaaaa aatcttttta tctactaaaa gtgattttgg agtaacgaat

93901 tgtattgtat tgcaacttct agtttaaatg ggaggaagag gcattccatg aagttcgagt

93961 aagacagata ttgacgcttt cgagtgtttt ttttttcttg ctataaaaat tactgggatt

94021 aagattttct ctatctctgg agccgttggc ttggtcgagc gatgtctttc acgtcggtct

94081 gttcgtggcc gtcatccaga tcggcagttc ttacactgct gatttactca gctgctttaa

94141 tgtgtgttca aagtgtactt tgtaactttg tatcgttata acgatatgcg aaccggtaat

94201 ttttgagaag atgttagttg tattggagct gaaggaaagc gaacatgctt ttatatcatt

94261 tttagcatcc caattataat tgtttaaata atatggaaaa tttatagcga aaaaaaactc

94321 tcgaaaacgt cagtatctcg ctcatgatgg aatgcctctt ccttccattt aaactagtag

94381 tggcattcaa tgacattcgt tacactaaaa tcacttttag tagataaaaa gattttttaa

94441 aaattgcgat ttttgaagaa aaatattttt ttccctgtta tgtaagcaat aatgtagcaa

94501 gtgctatacc attttagaat ccttattaaa tgcgccaact tttcgcgcga cgtgccatac

94561 tagcgttggt acattttttt ttcaggacag tcatttaaag ttgggcgaat atttaatcgt

94621 tccgttatag ttcggataaa agtggcatta tcttctaatc tgtagcagtt agcttataat

94681 tttaaacaat tggttataca aacactattt aagattagac aagaaaaata aaaattttaa

94741 aaagtaaaga gttatagcta ctgcaggcta aaaaccaaca cagggtgtac ttttttcgaa

94801 aatttagaac atgctatagt tcataaacta ttagacatag attttttttt atatttttct

94861 ggtgtctctt aggtcttctt ctatcacctg tttcagtttt ggcgtcgact tacgggacac

94921 cctgtataca tttattcgtt ggtgacttat actttaactt taactttatc ttagctttat

94981 acatatttac aattaactta aatatcttta catcactaca tccaaagata tattgtacta

95041 tagatggaac actcgaagcc taaaagtgag gcattttctt tgacctccgg aggagcctac

95101 ctcggagcgt acatcacgta cggaaatttc ccctagtgtt gacctttaca ttacatttga

95161 tgacataaat agttcggtcg actatgtaac ctcttttctc atattttatt tcatagtatt

95221 tactaaagtt taatattata attaatctag acatttttaa aacgctaatt aaaaatttat

95281 aacaaccata aaacaaaaat agactttgtt aaagattatt tggatttaaa ttgttttaaa

95341 taaaataata ataatgaaaa acgataataa acattttaat gaatgtttat gtttattcta

95401 ttgtttatta aaaaaattaa aaaaaataca ttttcccaat tcaaatatct ctttgactac

95461 atcgtcgttg ttaacacttc gacttcccgc gcgattctct acggtttttt cacctaactt

95521 ttttacgaat aaaaggcaat ggtcttaagg ccattatgcc accaaagagt tgcgatattg

95581 actgaacatt taagaacttg aatttctact actaatatag aatttcatcc accaatgtgt

95641 tgtccctctt actcattccc aaagatatag agataactaa atatatattt tttacatgtg

95701 gttcgcttca attaccagtt ctctaattta tttctaatat taaaactagt ttattataaa

95761 tagatatccg aatcgaataa tagcatttcg cttcctacgt gacgcttaca atctgacaaa

95821 cgacaaattt ttaatgtaat ttgaacttta agatattttt tatatattat aatgtgacta

95881 tcgagcgctt gctcgttcgc caaattggcg aagcgaccgc tgtgcaaaca tctgcaaaag

95941 cagatgcgtt gcctacctta atcgacagag gagaggcaca gaaagaggat gtttcccctt

96001 tctatgcgtc ccttcctcat caaatccaca tacctttcca cgggaaaggg aactgaactg

96061 ccgttagtct acaattaagg actcctttag cgacatattt cctccttatg ttgaggacca

96121 ataacttgtc gattatacga ttctggagga cactcggaac taaataatag accaggggtc

96181 tcattggacc aaattatgta tcataagaag taccaggaac aaggatggtc tgtattttgg

96241 aataaatttt atgaatgtac cagtaccaaa cgtagaaagt acaacagtaa taatgaggaa

96301 taaagcgtcc tcgtttctaa ataaagcgga ctgtctctgg atgttactta tagtatattt

96361 gactatataa acaagaccta ggtcatccaa tcgattcgtg atcatacctc tattgaggtc

96421 aattgaccaa caattggtgt caatcagccg ttagaccgca cgtggattag tcaactaata

96481 tcggcccctt tgggtttgaa ttaactcgga tcaagttcat attaataatt attacgaccc

96541 ttaagccttt ttcgcaccag gatagtattt tagtcgagta gcaagtagtt gtatccgaac

96601 aaaggattta acataaagaa gtctttacct gtttattata actttgtcct tggttattcc

96661 ataatagttg ttattcagtc attagcctct aaaatactgt ttaattgtgc gtgaaatatt

96721 accaaaaaca taactacaga gtctgtttcc taaataaaac aatttgaata aaatccgttg

96781 gtatattcag cgtttcgtag aacttcagtt atgtaatttc tttgacacta tagtagaaca

96841 agcttatctg acccggtagg cgtaagtgaa tctaaattac tatttattga cgcccccctg

96901 tcaatctttg ttaaggagag cgctcaccgc aaaagaataa agagctaagc gggaactggg

96961 cgaccccttt ttctactttt atacgaaact gcatacttat gtctccacca acgaagagaa

97021 ataagatgtg tcaaaaagac ccgaaatgag cctgagctga gctgtgtcaa atgagacagc

97081 tgcgttatgt tgtgctcgct tagctcagct ctttgttggg acgcaagcac atttgaggcc

97141 gctcagctca ccatctcaca ctcattactg ttttgcattg ggcgctcaga tttaaactgt

97201 atagtccggc aaacttgtct gacctggcga ggtaaagatt aggtaaaaca ttacataaga

97261 aactcgctaa atattatcag acagctacaa ttcgatgtgt atctttggta ctagcccacc

97321 aagccagatg agtttattta aaaaaaaagt tagtaagcct gtggtgacac atgacgatat

97381 gaaggttaaa ataataagat ggtattaaaa ttaaaacctt tcaatgtatg ccagttgtct

97441 tactcaagtt taattttaaa tcatatctcg ttacaatata gttaattaga ttaaaatctt

97501 ctgtatagat atctataaga acattttgtt tgtccattat ttttttaatt tatatatgaa

97561 ataatttgct ttattgaaac gcagagttaa taagtccgta gattactacc taatgcctag

97621 ttcgcattgg cttagtattt tagtcgagta gcaagtagca tttatccgaa caaaggattt

97681 actactgttc ggactcgttt aaacctaaat ctatgcacta gggtagtaat ttagtaacta

97741 aacgtgtccg aacagcaaca ttttagtaga atagattagt aaatcctttg ttcggatact

97801 tgctacttgc tactcgacta aaatactatc ctagtgtgta caaggcttaa aggcttgtaa

97861 gaaagcaagt cgtcccactc tttctaacta gaaacacaag gtgtagtaca actttcacag

97921 tttttctata gtgcatacag tattataatt gcacctgtgc ttagccaggg cgttgtcgtt

97981 agttaaatca tgacagaggg cgctagggag ctgttataat atagttccac ttatgtccac

98041 cgggtcacac atcgttgttc gtctatactt gatactgaag taaacgtcag gaggcaaagc

98101 tagtatgtac cagatggcat tcattgcaat taaagtaaag ccttaagtat tatactcgtt

98161 aatgtaatga agccttcaac gcccccgtaa tacatacacg tgcgtattaa gatagtttac

98221 attgctgtaa atgtagcagc tgtgcaaaca catatacgta ataccggcct tctttttatc

98281 actacatagt ataaaacaaa atcgctttct ctgtccctat atgtatgctt aaatctttaa

98341 aactacgcaa cggattttga tacagttttt tagtagatag agtgattgaa aaggaaggtt

98401 tatattttat tttattaact agcgacgtac ccggattcgt acgggtgcaa tttttattac

98461 tatggctgta tacaacgttc acagtttttc tgtagtgtat ttagtaccag cattgcaccc

98521 gtgcgaagcc agggcgggtc gctagtttat tataaattaa tagagaagca agcatgtctt

98581 gcgacacagt aaatatgggc ttctgcagct cataatgatg ggcaaaatcg ccggcaaaag

98641 acgcgttgga agaaaaagaa agtcgtggtt aagaaacatc cgggagtgga ccactatcgc

98701 cagcgtcgaa caactttttc gcttggcgca ggatagagat gggttcgctg agctgacggc

98761 caacctccag taatggagag gcattagaag aagaacaaga agagaagcaa gcggtcacct

98821 agattcgctg caatggcgaa gcgaccgctg cccatagaca tcagcaatgg tcgatgcgtt

98881 gtttaccttt aatcaacgaa ggcagagaaa gaggatattt cctaagcatc cctacctctg

98941 ccaaatgcgc ttgttcgtac atcttttctt tgttagaaca tggtaggaag gggaatacaa

99001 cttaactgaa ctttgcgtaa gcgtagatta tttgatcaaa caggatgaat gaataacaat

99061 aaattaacaa caaaaacaac ataactcacg cccgtaaccc agagggggta ggcagagaca

99121 ctcgacctct ggcacgattc tgacacacct ctctcgcttc tacattcata catctacgca

99181 aacacgacgt ttacgtgctc ttaataccgg tcttcttgag taattcctca atttgaccgg

99241 tatgagtccg cctcgggcga ccccgtccaa cacttccact cactttcgca ttataaatcc

99301 tctttatgaa ttaacacaat caaaaattat agatatgacg tttaggtatc ttctacacag

99361 tagttaaact aaggtcacgt tttcattatt cgctaaggat aacgataatg cgttatcata

99421 ataaataatg cgtttaaaga taataaatta ttatcttaat atacgcattt tataaataaa

99481 aactgcctaa tttttatata gatttattaa acacggacag tcgcggataa ctgcacttaa

99541 gtgacatttc atgtgcaaaa cgttagtcaa ttatcaaagg tgattatgac gtatccctct

99601 gaaagcctct ttgttttatt tgtcattaaa cgacataatt cgatacatac attcaaataa

99661 ggggttttac aacgcgcgct aaaatagaac aaattcattt taatttatct gttcacattg

99721 caacgctttt aaattttaac cgcgcagcgt tggatttcga acgctagatg aacgaaacaa

99781 gagtaatttt acaaaaacaa aggcgttaaa aaaaacgtcc atgtgaatgc ataataagag

99841 taaatactct ttcatttcat aataatttat tcaacatttt attgaaagtt caaaaggttg

99901 aacaactaaa ccattaggcc ttaaattaaa gaacattaac tttacagtaa aatgttaata

99961 aattagttca cgactgtaca ttactaatta gattattatc ctgtctgttc gataattcaa

100021 gtcgaaatat tagattgaag acaaagactg ccagttgcaa tccaacgttc ataccgaacg

100081 gacgcgtgtg tttaaacttc gataattaag aattaacatt ttcgtaatat taaagtgtta

100141 tatttaaaaa aatggagtca ttaataaagg agagagcgaa taagataacc gaagaggaat

100201 ttaaaaggaa tttaaacgaa ttggagaatg tggaaactca agaagagatg gagaagaagt

100261 tagatagtct gatgaagggt ggcttcttct tgacgaactg gatgaagagt ttgttcaata

100321 agagtagtaa tgaaccattt aacccggtct ggttgtctgt aagtatgaat aaaatataaa

100381 aatacagtcg gacctggatg cgagggtccg agggaccgcg atatttgtca cgctcatata

100441 cgtcttctct ctaatcaggt ttttcgatta tgaaagttcg tcgggaccag agaatgagtc

100501 tcgcattttt ctcgcaggtt tctaagctta tctacgtttg actgtatatt tatcggtata

100561 caaaaaaaat atcacaaaca tatgtttttt attagattat ttttaaatga atctttttaa

100621 aatcaaatgc gatatttaag tagttttatt tataaattaa aattaatgga aggatatgca

100681 aatagcactt ttgcgatata atcgatagtc atttttatgt ttcacaaaaa tataaagaaa

100741 caaaacaaaa cgttggaaac aaaggagcac tcatacggaa cgcggaaaca ctttcacttg

100801 accactgtct ttaaagcctt gtacacacca ggatagtatt ttagtcgagt agcaagtatc

100861 cgaacaaggg atttactaat ctattctatt aaaatgttgc tgttcgaaca cgtttagtta

100921 gtaaattact accctagtgc gtaggtttag ggttaaacga gtccgaacag tagtaaatcc

100981 tttgttcgga taaatgctac ttgctactcg actaaaatac tatcctggtg cgaacaaggc

101041 tttattgcac ctagctggcc attcgtgcaa gtaatactga ggattctaaa aaaaaatgtt

101101 aatatagttc aaattacaca cgaatcaatt aaaaatatta ctatcttaca aaaagtttgg

101161 ttgtatgaca tagtttggaa aaacagccta cagtttcttt tattttcttg cggatggtgt

101221 cgccggcgac aactagtgct aaataaaaat ggaaattaat acgctataac ataattttct

101281 tcacgatatg acaataaatt ttcacaaagt ctattgtctg tataggtttt aaattgcaaa

101341 atatttgctt gccaacatta cgtgttatcg tttactaaac tgaatatcat attgaaaaaa

101401 aagcaaagat tttattgcaa tcatatgaac aggattattt aaaacaaaat caaatggtaa

101461 tattctaagc actgctttaa gtatgaatgt ggaggtacac cggtagaggt aggaggaaga

101521 gattgcgtga aaggtgatat gataaacaag ggtgtgacaa ctgacatgac ggagcataga

101581 ttaacgtgga ggacaacaac ttggtgtcct cacactacgg gagtaggatg tggtagtgga

101641 aattattcat tatttcacct attaactgag ttactgctcg attacaaatt tgttttttat

101701 gaaatgagag gaaggtaaat tgctttgtat tttgtaagta ttttgtttac ggttacagca

101761 tgtttacggt tagaagaaga ggataaatta acagaaaagt atactaatgt agcaataatt

101821 tatctatatt ttacaataga tgtcaatggc acaaagaagg ttttgcaaag ataaaaatgt

101881 atttattaaa agaaatcttt gtttttttaa ttcagataga tgaatacaag cagcaagctg

101941 aggaaagtct gcagacggct tggcggatac tcaacctgcc gacatggaga ctggagaagc

102001 gaggttcaca tcggggcgat gtggtcgaat ccgtcaatgt ggaccagctg ggcaaggtct

102061 acaggttcac tgtaagttga cctctttgca gatggagaag gaataaacgg cttctcacag

102121 catacaacaa tgcccagtgt cactctacaa ggtttaatat cttggtactc cgggagatca

102181 ctagcaacat aaaagggctg ctagggttct ttggagagct agtctgtgct agagtgatcc

102241 aagacctgac ctcacgcaaa acgcgccagt cttggagttg cggaaatata gccctattac

102301 tactactact aaggtgacca catggacata aaccgtagta tttggtgcca attcgaacct

102361 ggatagagag tccaatcgac cgcgtcatat gtctcgctta agcctagttc gcaccaggat

102421 agtatttcaa tccagttgca agtaaattta gttactaaac atatccgaac agcaacattt

102481 tagttgaata aattaataaa tccattgttc ggacactttt ggttatcaaa tttcttggtt

102541 ctcgactata atactgtgcg aacaaggctt tatagagatc tctggcttat agaggtttct

102601 ggattatcaa gtattcactt atgtaggttc gactattcta ttttcgtcat tttgacattg

102661 aatgtagcag ggttaaggtt ccaattagta aaataggatt gttagtcata gatgtattta

102721 agtggaaaag aagtgagagc tgtcatttct aaccacctat ctatttgtag agattagtga

102781 agttcttggc catgattatt taactttttc agggtgtagt ggaatgtcca gcgaagttcc

102841 tgtacgagga gttcaagaac aatatggcca agttgccgga gtggaaccct actatattaa

102901 agactgaatt cattaaggta aaatattttg gttaatttaa ctatagtagg aatcttgtaa

102961 atcttggtta acgattttta tttttattaa ttaaataggg gggcgtccat taataataaa

103021 cccaggttga ttgtatgtat ttataaccaa ataaacttga tctccaggag ataggtccgg

103081 gtatagacct gtcgtaccag gtgacagcgg gcggcggccg cggcatcatc gcgccgcgcg

103141 acttcgtcat cctgcgtcgc accgcgcagg tgacgcgcga aggccgcgtc acagacgagg

103201 agccctactg ctatatgacc agcgggatca gtgtgcaagt gcctggatat ccaccacaga

103261 aggatatggt taggtaaata ttaaaaaaaa tattgtagac tttattcact tatcattaag

103321 gtaacaaatc gaatgacatt ttacttcaaa atagattcag tcattgaaac atgacaatgg

103381 aaattacccg ggaaataata attttagttc acttttcctt cctatccttt tcttataaag

103441 aactgatgag aaggggtagt ggatttggta ggggagggga tgcataggaa gaggaaccat

103501 cctttgtctg tgcgtctcct cctccgtaga ttaaaggtag gcaacgcatc tgcatttcag

103561 atgtctatgc gcagcggtct cttcgctatt tcagcgaatc ctggtggccg cttgctcgtt

103621 tgccactttg taatatatat taacattacc tttgtcagac cgttaatagt tttttacccg

103681 ataattttat tttttgttca attttgtttg tattttttta aagcattgtg tgtaaaaaaa

103741 tttcgatacc tttttatatt cgtagtagtg ctgtatttct gcaaatctac gactgtcgcg

103801 ttttttacgt aagaaaattt aaaaaaatgt ttttatatta gtaattttgc tgtgtttaat

103861 ccaaatctat atttatatcg agtatcattt taatccctta ttaaggttaa agtaaggata

103921 ttattatcat tcagatatta gataaatgat atcgaatgca tcgagtcatt ggaactacaa

103981 tgatagtgac ggttattttt aataatcaga acaaaaacat agtaaagtag catatatcat

104041 catcatcatc accctggtct aggagcctac cctatacgat atagaatata tatgccggtg

104101 ggggagatat ttcttcacca gtggagtcca tttttgaatt ataaaattaa cagtaacgtt

104161 aggttcgcaa tagtttcaac gtccgctcta gccgacagtc gcagaagaag tgttgtgtcc

104221 tgctaccttc ttcgctcggc cggaaacgtc gactctatca aacgacgaca ggtggcgctg

104281 gcgttcctta cgttctgcag tcccgactaa caccaccaat ggcgctagta ttgcgacgat

104341 tgcgacatat agatgtattt tgccccggct acgcacgggt gcaatgctaa tactaaatac

104401 actacacaaa agctgtgaac gttgtatata acagccatac taataattgc actcgtacga

104461 agtcgggtac gtcgctatta gtacatatta accttcctct tcaatcactc tattaaaaaa

104521 actgtataaa aatccgtttc gtagttttaa agatttaagc atacatatag ggacagagaa

104581 agcgactttg atacatgtag tgaagtaaaa tttaactaca ttttttttta gaatatttta

104641 ggcatttttg caatacaata caagggtaaa ttttaattta acttcgcagg ggtcataata

104701 aagtgggttc gtgggtgatg aagccgaagt ctacgcagag cgccggcggc aagatagagg

104761 agtgcaccat attccactgg ctcatgtgct gtgatcttaa aggtattatt aacttactca

104821 tttagggggg ggagccccct tatctatact ggcatcaact cctataattt ccctataagg

104881 aaatgatggg aagggtaagt gtattgacag gggaagggac ccatattaag aggtattatc

104941 ctcttgctgt gcgtctcctc ctccgtagat taagggtagg caacgcatct gcaaatgtct

105001 atgggcaggg gtcgcttcag ttttggccac ttgctcgttt gacactttgt attataaaaa

105061 atgtattttt gtatatattg aaataactca acaactactt tgtcgatttc aaaaattctt

105121 tcaccattag aaagctacat tatctccgag taacataggt tatattttat aattccgcgc

105181 gaagtcgcca acagttagta ataatataat actccacaat gtacatgtgt gggcgtcgcc

105241 tagttttgat cagcacccct atatcctatc cggttgacgc ctgtataagt cttggtattt

105301 tgtttatatg aaaatcaaat gactaaaaag ccattgtgtt gatagtgagc gaccatattt

105361 atactcacct ttcttaatag tgttaaaatg ttattattaa ataaaaaggt aatgttacat

105421 acttaaagga gaaaaagtgt tttgcttaaa aatatttttt ttattttcgt gttttagcat

105481 aaattatact ttgtaattgt actagttaag ttaaaattaa ttataatact tcggcttcat

105541 tagaccgagt tcgcgataaa aacggactta agccgaatta aagtgtccat ttttatcgtg

105601 aactctgtct aatgaagcca aagtaatata attaattttt tatacatttt atattattta

105661 tttgcaggta aaatccctca gttcgtgtta gacgcggcgt tcgctacagt gatgttggac

105721 tacatagtgc acgtgcgcaa attcgcggca gaatcgaagg ctaaaggact cttctaaagg

105781 ttaattggtg cttatagata ttgcctattt aaacttcgta atataaaatt gtgtacaata

105841 ttatgagcgc cttataaact tcggcgccca tttttgggca attctgaggg gtttttggcg

105901 cctttttcta tccgacgcct aaagcggtcg cctaaccgcg ccacccgaca ccgatgcatt

105961 tcaaataaga atataattat aatttcgatt tgttaaacgg taaatgaaat ttcaacacat

106021 attggtattt ttttataatt cagggattca tggacaacta gcgacccgcc ccggcttcgc

106081 acgggtgcaa aacttatact acactacaga aaaactgtga acattgcata taacagctat

106141 actaacgata attgaacccg tgcgaaaccg gggcggctcg ctagttatat gtaaaccttc

106201 cgtacgaatt actctatcta ttaaataacc gcatcaaaat ccgttgcgta gttttaaaga

106261 ttcaagtaaa tatagggaca aacataaaaa agcgttattg ttttatacta tgtaagtata

106321 aggataacaa ctgaatcggt aggcgtgtca aactaagtaa tgacagctca ctagcgccga

106381 cctgtcagtg tcaaaaaatg tagctgtcac tttttctttt ttgtcatttt ctatgtcaag

106441 gttttacact tagactatta atatatgttt tctctgtcta cggttttaca tatctgtttt

106501 gtccgaacta atcatgatag cctctctcac cagaccacct tgtttaccta taaactatta

106561 ttagttaagc tataaactat aatttattac taaaatatcg attttgttta ttgattaatt

106621 tgacagctgt aatgtaacaa tatggcgcaa cttttttcta atcttatttg aaatgcatac

106681 gacatacaaa caccctctca cggtgaagga taacatcgtg atgtctgcac atatcggcta

106741 agaaattcac agatatgtat gaagtcacgc aaaatacgct ctagtcgtgg attggaagaa

106801 aatggagccc atcatgggtc gaaatacaat acagtacata cccgttatac atattttagg

106861 ccttttgtac acgataaacg caataaagta gctcggagac taaagtatca gaatagcgac

106921 gcctttacgc gaatggcagt tttcatttta tgacaataat tgttaaaaaa atattgcata

106981 atcataaatg cgttgattta attgggaact ataaaaatac acaacgcaca ataaaattgt

107041 ttaaaaaaca tcaggtgata tttaaatata ttggaattaa attaagcaaa taaataacaa

107101 ttcgctttta aatatttaat cataaaatta tttcaattta tacattatta gtcttctttc

107161 tcttcccacc cttttcttta aggaaatgat gggaagagga agtagatttg gcggaagaga

107221 ggacgcataa gaagggagaa atcctctgtc tattggtccc ctccttcata gtttaagggt

107281 aggcaaatac atctgccctt gcagatgtct atgggcagcg gtcgcttcgc tatttcggcg

107341 aatcctggtg gccacttgct cgtttgccac tttatagtat aaaaaaatac attaacaaaa

107401 ttaaaaacac aacttttatt aatattccgt tttaaaaaaa tattgacatt taaaatggcg

107461 ccaaaaacaa gcatacgtca tcgatctgat aaaagtacat acctaagtca aagccatgac

107521 agcattttag cactcctaga cattagagca gtacacgtac agactagacc acaattacta

107581 ttgaatctac cttaaattag acctaaacat agacattaca atagaccctt tgatattaaa

107641 attatgagaa aaaaacttat ttttaaaaat aaaaaaatac taaaatagta gaaccatttc

107701 attatggcaa caccaaattt aattcttttt atgccaaata ataattcttt tcatatttaa

107761 tcatatttta tttaaaaaat gggaacattt agcttcatag aaaagatgtg tattttttca

107821 aactattttt taaatttatt attgatattt aaattggtct tcctaaatta tgattataaa

107881 aagccaagaa ccaccgaaaa aagtgtaaaa agctttttaa cagacttcaa aatcgtctgt

107941 atgatttttt tcaaagtagg gtagtagaga atttaatatt ggctcaaaaa tacaaaatat

108001 atgtcgactt tattttagtt ttatttccaa tgaatttatg taaatctact aactcttaat

108061 tactgtgggt tttcacggtc gctttcaatt gtcactttga acctaaggct tcattacacc

108121 tatgttacgc aaaataacgg ctttacgccg acgcgaaatt cccgttttct gtgcgaattc

108181 ggtctaatga agtcgtaggg ttagaaaaca aaatgattta caaccactga aaatctacct

108241 tagaaatata aaccaatgca ttaaaagtac atataagtat aaaatgttac cgttttattt

108301 tgcatgcatt ttattaattt tattatattc atagaaatgt aagctatcag tcgttgtgta

108361 aagggactat tataagtaaa gtattttttt atcaattaaa gctgtaaata ataaattaag

108421 ttaataaatg ccttatttgt atgaattttg ttttttttac aacgtacctt tgaaactaga

108481 ataaagaaac aagtaaaaaa tggtgatatt tttaacaagc gccctggtat taaatcagat

108541 tcttgcagcg cccctagcgg gcgtaaagag cacctaaaat atactattga caatgacgat

108601 acgaagcggc tggtcgcaaa tattctccaa agacccacag atccacgtaa tatatccaat

108661 cctctccttt gtccatttat gggaagagta caacgaacct aaaaagataa aactattact

108721 agctgcttcc cgcgcctccg tctgcaagaa aaaaaatcat agtaactagc ctatgtgttt

108781 tagactatgt ttctttatcc atacatttca tcaagaacaa ttgaatggtt tctgagatac

108841 cttcaaacaa acatttatgt gtcgtaactt ttacatttat aatattacta gctgttgtgg

108901 gcgacttcgt ccacctggaa ttaaataata tagcctatgt tactcgggga taatgtagct

108961 ttctaatggc gaaagaattt ttaaaatcgg cccagtagtt ctgaagttat ttcaatacat

109021 acaaaaatac aaagtttctc tataatatta gtatagatta agatgcaaaa ggccccatta

109081 ctataagcct ccctgtaacc tttatcttaa aaatcacctc taaggttaga aagcagacaa

109141 agggaagatt gaaaagcaga aacatgaagt acgtacaatg cttgtaacgc cattttttaa

109201 aacataataa ctacgtattt tacgacaaac tcaggagtag aatccactga gatgccgtgc

109261 tgttagtcgt ctttttaatt gcacaaatag tactggtaat acaacagtga aaattgcctg

109321 gattttttca aattatacat gttaattatt gaaacaaaaa ttaacgcgca tgacagataa

109381 accgctcaaa ttgcgaaata gctttagttg cctgggttac tggatttagt ttttaataca

109441 gtcgaacctg gataagcggg actctaaggg accgtgatat ttgtctcgct tatattggtt

109501 tctctctaat ccgtttatct cgcttgtgga ggtagctcgt cgccatgttt gactgtagtt

109561 aattagtgtg taagaaaaac tcttttaata ttgataatta tttaatttac aaatataata

109621 cagtttaaca ataaattaac tttcgagtac cgcttaacca tcaaggtcac aagctacgat

109681 atgtttgaga agttatttaa tttttaaccg tcaagctttg ggcttcattt tcggcggttt

109741 tataatcgag ccgatgcttt ttcttttcga cattgtagct ttctttttaa gtggagtttg

109801 tactttcttc attttcgact ctttagttgt tttaattcta acaactttta cttttttatc

109861 atcgtcggtt gcttcgactt ttcttttaat accttctttt ttctttattt tttcagtatc

109921 tgttactatt ttgtcgcttt ttttagtttt catgtcagac tttttagttt tggtattttc

109981 tttattggct ttcttttttg gtttttcatc ttctgtcttt tcggtgattt tcttttcttt

110041 tttcatagtt ttcggtttgc ttttttgttt gtcagcggcg ggagcgagtt tgaaggaacc

110101 agcagcacct attccgttca cttgcactat aataccggct tctacttcgc ttttaataag

110161 tttttttatg atataattca ctttagcgac atctacgtgg tagttttcat gcatatattt

110221 tttgatagca tacaatgaag tgccctttct gcttttcaag tcggttaacg cttcgtggac

110281 cattgttttt actgttaact tctttggtgc ctcctcattt tctcttactt cttcgtttga

110341 tggtcgtttt attatagttt tctttgtttt tttgggtggc gacggcgttg tctccatttc

110401 atcctcgtac ccgctcgaca ttttcgtaat gttttttctt caatatcaaa agttttgact

110461 gggacacgca ttagtgttca acataagtat tacacaaact ctaaacagta ttttttttgt

110521 ttaggaagaa gcttttttgt ttaggatttg ctgtatttta actggatcat gaggtgcttg

110581 tgcgcaggga gtgtttaaca gaaacttctt tagtagcgac ttgtgaggta tctcaaagtt

110641 aggtaacatc ttagatatta actaattatt gtacatattt aataatttga aatcggctgt

110701 ggtttcattc cgtttagccc aactatcccc ggggataaat atgtaacaaa ttccagcttt

110761 agttcaaaaa tggtttagtt tagataatgc catttttggt gatttcttcg tttgcaacgg

110821 aatagtagtc tcgaatagta gtattgaacg gacctaaata ttttaagtaa gggatgtggg

110881 ggcttaaaca tatttttttt aattataaga ttttagtgac aacggtaagt aactctttat

110941 aaaaactacg taatattttt tagtttaatc tgacctgtta cttaattata tagttatagg

111001 agtaaatgat tttatttcca caatacgaga tgcgataata ttattaacta cgatcaaatc

111061 gaaaatctaa attaaaaatg ttaattgata atttaattac tacccatgtg cctcctgatc

111121 gtaaatttat ttgctaaatt tgaagttaac cataatcgtc aagacaaaag catagctaaa

111181 aaacacgatg aaccgttcga aagaataagt aaaaattatg taattaatac aaatagttgc

111241 ataaaaaaac attttatttg caactcctta cggtcacaaa aagtacttta ttgaccggga

111301 aaattgaggg aactcaattt aatagtattt aaataggttg agtgaactta ctaatgtgac

111361 cactactatt taacatttaa ctatcattat gttacgctgt gttcaacaca gaggtccgat

111421 tttggcaacg aaaaatttct ttgccttaag ttggtaaaaa agtgctgaat tttctaacag

111481 tggcacttcg aaaatggcct ttatttcaat aaactcttat attaaaatct taggcttgtt

111541 ataatcgttt taggtgttgt gtattaaata tgaacgtata agaaattatt acgtaaaatt

111601 agcagaacat ctaattctat ttgacgcgta gcgccttaca gattataata gcaataattc

111661 attcacacct taaactcgta aataatgcct gtaatcatag ttatctcaaa ttgtggatta

111721 ttaaattgta ttaatatgaa ataaatactt actacgttta ttatgacaac aaatattgtt

111781 attcaagcat acagaacctt tgaagagcgc aaacttagct tttgtaagct atgattgagt

111841 cactgtataa tttggaaaca aatgtcttca atagtaaaga aaatacagaa atacaggttt

111901 ttaataactt tatgaacttc caagtgcaaa ccaaatttaa aaatatttcg aaataatcat

111961 tttgatgcat agatttatcg gaaaacattc actggtatta aattcgtaac tattctaaat

112021 aaacttttta ttaaaaaata agttacttaa ctcgcttaca gggccaagat aaccatttta

112081 agcgccttgg tgcgaaaata tttaggcgcc ccgatttgta ataactccat tagctgttcc

112141 cacaaagccc tgctctgtta ttttattttc ttgaaaatag tttggggttt atgtttttga

112201 tttgataacg tttatagtag aggtatttaa tatattctaa catcctaaag ggcttaaaaa

112261 ttccactcta tacagttcac ttttgcgcat taattttaac tgttttttga tattcatccc

112321 ttcgtgctcc ttgatatggg ttacacgtac aatatattcg tttcacgtac acgtaacatt

112381 agcagttaat cttcctacaa atatacaaaa gtaaataaaa actgtgcaat tgaagcttaa

112441 gccggctaca aacgttcagt tttttaattg tgcagttaaa acttaaaact gacgtagaca

112501 cggagaactg gacagacgaa ctgtacactt ctcagttttg cctacacacg tcagttataa

112561 gagcgcagtc caactgtaca gttaagcaca gttttaagtt aattagactt tagcgttgat

112621 aatcatcttg ctaagtataa agcctagtct tatttcaata attttactta cgttaatcgc

112681 ccgcagtcaa actcgtcacg acaacacttt ttggactaac ataacaatca tattttgtta

112741 ataaacattg tcggttgcta tttcctaatt atcttaacag tgagtttcca ctgcagaaac

112801 agttgtataa aacatatctt tatccctgtc tttcttcttg aagaaccaga ttacaataat

112861 tatttgttta tacaggcctt aaccgtagaa tggccaacgt tccaacaaat aaaattaaat

112921 acagaaatta ttccccttta aaactaccaa tatccctcag tgttataaaa ataaaacagt

112981 gcaaaatcgc gttagcactt cttaccttga aaaaaaagat ataacgtgtg tttattatta

113041 gctaattata ttctaacaat tttctttatg atgcttacag caaaacctcg acaaaccaat

113101 caaaatctaa cctctctata tctataaccc tcttaagtct gcaatataaa tttgatatgt

113161 aaaagaatga atgatttatt gtgactaaat tgagctcaaa gtatttcgtt gacgtgagca

113221 catagaatcc atacaataca gctgatgaca tcaccaaaga ttaataaata cgtaacgaag

113281 taatgatttt ttactcggct ttaaatattg tctttatcaa gatggaatac aactcaatat

113341 tccatgcgat caagcaacaa aagatatttt attgcaagct tgccgttcta agaatttata

113401 ctctcgctca tttctattga aaaatggcac gagctagaaa gagataaata tacaaaactc

113461 gtttacaatt gaattaacga gagggtattg gggtcacagc acgtgtttga atttcgaaac

113521 acgtttcatc caatacggtg cgttcgtaac cagcggttta tcattggtta gacttaaccg

113581 gtaccataaa acgtcgagtt ccagttaaat cgatagcgtg caccgcttct ccatacaaaa

113641 ggagcgatac tgtcgcaact ttgttatcgc tggcggtcgc ttggtactga tgtcgctcac

113701 tggaggatga atcttttacg ttattcgtgc cgccatttcg tcgcgaaaga tgtccggagt

113761 gatcgtattt atggcgttct gaattcgttc tccgtggttt tttgtgcatg ccgagctgtg

113821 ttttgacgag atttcacaaa ggtaagtgat ttttgttgaa tttgatatcg aacttccgtc

113881 cattttgatt cagcttttat tgtgacattg tttacgttgg tgttttgttg cattacacgt

113941 cacgtagcta atatcgtttc aaaatttaaa cgagagtgca gaatttcttc gaaatgtata

114001 tttggaacga taaaggtata actttgtagc gttatggttg attcgatatg atttttgctt

114061 tcacctgtat tacttacggt tgtaattttg gtagttctta gtaataatta atgtagagag

114121 taatgtaaac atgctgtctg taacttgata catgtttttg tatattgtgt catatgacat

114181 gtatgtgatt tcaagcagca tagtatgtta cgctggaggt tcttattttg atagtgataa

114241 ggtcgaattt ccagtgttgt ttatacgact gccttactta attagtttgc ttagggtgtc

114301 tctgtccgac gtgccagcag tgccggggca agtttctctt tctttcatgt gatttattta

114361 taggacgagc gagggacgcg ctgcccccca gggcgctcta tatttgtttt agtgttgggg

114421 ttgtcgataa aaatgggctc atcgatatgt tttgcatgca aaatatttat tttaattgta

114481 tgtagtcaat taaaatgaat tattaattta acagcaaatt aaaaccaaag taattacagt

114541 cagttaacta caattacaca tttttgtaca aaattaacaa tatttattgg atttttggta

114601 ttggattttg atattttttt ttacgaaaat cagtttgcgg ggtagtaaaa tgcaaattat

114661 ttgataatca tttaataaca aacacagata tacttatcaa tctgtttgat aacatacaaa

114721 catgtgccaa tgtgtggtgc cataagtcca tcccatttcc atcacttcat atcctattat

114781 aatttaactt actctctttt tctgctttaa caaacgctgc caaatctatc cgaattctcg

114841 atgtttagga atttcgatgt ggacaagtgc tcgcataact tcgaaacgtt cgctttatag

114901 attctcttct catcttctgt cttgtcatct ttatttgtac ctattacctt tttaacataa

114961 ttacacaaca taatgtaaat cgatctatct gcgatactta tcatttattt ctatgaagta

115021 agttttttaa tgatttttac atcatgtata atttaaagta ttttaatatt aatcaacgat

115081 aatgtaaaaa gtcattacta ttaaattaaa tcatattaac aaatatcgat aaataatgaa

115141 acggtacagc actagcataa aaaaatgtct gccgttgttg aggcaaaggg gactactgct

115201 tgacgataat aatcgcagta aacaaaaata tattctacgc tatatgcaat agagtacaat

115261 ttccaaacca aagaacgtcc ctttgaaata tttcccgttg caatggcaag ttaacttcta

115321 cctaatgcgc gaggaataaa gtacaatttc gcttggattt ttttatcaag ttgtaataat

115381 acaaatttaa gatggagaca attccatagt tttttttgtt gcgttctata caataattta

115441 tgtggggtga aaaatggagt ctgacggtaa gcgccaatgg ctcgatgtgg ggagcgatgt

115501 tttgaagata ttataagtga gttgtaaaac tggtaacgat tttattactt gcggggaaat

115561 gtaaacaaaa cgctaatgtg ttagtgacag gtcaagcatt accatgtgtc tgctgacctt

115621 ttgtaacact ttttttcgca cccatctttt tcattcaaaa tgtaattaaa tagattattt

115681 aatataaaca tacattttac agcttaacaa tatttataaa aaagtctctt tcggtttcaa

115741 tcggatgtaa cttttgaact cagttgtttt gcatcttttt ttattaactg acggctgtca

115801 aaacggcaca ttttgtaatc taagacgggt tgtttggttg cgaatttttt taatttttga

115861 tcgagcagac attttcatgt tagaaaggtc ttaataatag gcctagagtt cattcaatat

115921 aggggaattg attcgctata acatttacct gaaaaataat ttgggttaca actaaaaata

115981 taaccgacat taccgcttac cgtgtcttct ctggatgccg aactaggtgc gtaggctctt

116041 cggccgtcat gctatttttt atattaggag acgatcagtc aagtgttgcc ggacgcattt

116101 cacgctgata ttcggtaagg tcatttttat ttaggaagcc gtgattttta gtctaagaaa

116161 attttgatac ccttaccata aaagcatttg aggccatttt cgcgctgttc gacaatctgc

116221 aagagtgata gagggataat tttggcgcgc atttttattc tgcgtcacag attgttcttg

116281 gtttaggcta aattattttt ggtgtcatac gatacgcatt catcaccgaa gtgtcagatg

116341 cttttcgttt ttttttattg catttttgcc cattgttaaa ttgcactttg ttttaggatt

116401 aagtccatta tgttaccgta gactggtccc ttatgaataa aatgttaccg taagttttaa

116461 cacttgaaga aaaacatact gttatctatc ttttttttaa agataatatg agatatgcta

116521 aaaaaatttc gcatatgtgt ttaggaaatt gaatctcacg gttaacaaca accttcaaag

116581 gatgtttaca attactaata ttacaataca aaacagtaat tattcttata aaataatttt

116641 ttttttatta cagatgcagc agccaattta tcaagaccag caagggtaag aatcatttaa

116701 ttcttgtaac actttcgata tattttaaat attttataga atttgacgta atactttttg

116761 tttttacgaa atttagtact gaaatgtcaa aaacttttac cttttactgc aaatatcatt

116821 gttctgttgc tacctggaat tggcatataa acagcaccaa gtagcgatag gcctaaatgc

116881 attttttctt ttttacggaa ctaataaacc atttcattta tttccagcgg gcagcagggg

116941 gccggtcaac agatgctcct gtgccccgtc agattagtat acgagacaca aatcttggtg

117001 caacctggtg agtatctcaa caaatgactt ctagtcttct tcttattcat gccatttatt

117061 tgtcgcgtct gtcttctgtt tttttggtta tttgataatt taatttgttt ttcaggtgaa

117121 caaattcaac cgaatcaaac gatattcata aatcaacaca acccgccgcc atggatacag

117181 aacagaccac agaacaacgt gatgtacgtc caacagatgg cgcccaacaa cttcatgccg

117241 caaattcagc agcagccggc gcaaaaccca atatacatcc atcagaatta caacaacata

117301 cagcaaatga taccacaaca aattatatct caagcgcaac ataaagaaat gcgtaccgca

117361 aatgtacaaa tgacaaacat actgcagcgg aaccctgcgc aacagttgca gcctaccccc

117421 agtccgtaca caacgactgt tcagggcgag gctgtacaaa gcactgcaac tcccaactac

117481 ggagtaatac ccgcaaatta cttgcaaaat caaattaccc aaaaccaaat caacttcata

117541 ccaacaaata ctaataaaca aataatccaa aacgtacaaa caccacccag accagctgta

117601 tcaacaacca atgttataaa ttttcaaaga aatattgaaa ttcagcaaaa tataccgaga

117661 ccggtagtgc agcaaacgta ccagcacgaa agtaatatga tacaaacatc gttaccacag

117721 tcaataacga tggtcccaat ccagacaaca caaccacctc aaagacctaa cgatacacac

117781 gatcaagtca cgcaggtacg accaatgcaa acgttggtcg ccaacaaacc aaccttacca

117841 ccaacagtca gcacaatttc taattcaaca cacgaacgaa tagttaaaaa aattgcagca

117901 gtcacaccaa agggtaatat tacagtaaac acattggtta atacagtacc taaaaatgtt

117961 ccatcgtaca gctacagacc tatccaaccc agacctcagc agcagagaaa tatagcacct

118021 aatatcatac ctacgaatac ccagacacaa ttcaagggcc cgcccaagat gcagcagcct

118081 tacaacccaa accctctaac ttatgtgatg tcatcgaatt atactaacac tgaccaaagt

118141 atgtttaata gaaagagaaa gagtgaatcc cctgatgagg tacaaaagaa aatgacgatt

118201 actcacatac cacaaaagaa ctctccagtt gctgtggtct gctcggtgca gacaaacact

118261 gtcaatagta ttggtgtaaa tacgatgccg atgcaaagga cacataaaat gcctccaaat

118321 attaccagag ataatagaaa tcttattaaa atacgtaacg aggaaagtat taaagaaata

118381 cagcaaagag tcgaaattca aatagccaaa gaatcatcat cgccgagcga aacagagaaa

118441 ttattgagga acactgtatt cactcaggct aggaatagag ttttagcgga taaacaagaa

118501 ataataagta caaatgtaat aaaagtcgaa actattacta gtgaagtaaa atctgagtca

118561 atacttcaaa aggaacttga aaaggaacca gccaaaaatt tggaacaaat taaaaaagat

118621 tcagtcaaaa acgaagagga aaataaaatg gttttaatga aaaaggaaga ggaaatcaag

118681 aaagatttaa ttaaaaagga ggaagatatt aaaaaggaat tagtcaaaaa agaagaagaa

118741 attagaaaag atttgattaa aaaagaagaa attagaaaag atttaattaa aaaggaggaa

118801 gaaataagaa aagatttaat taaaaaagaa gaaatcaaaa aagaattatt taaaaaggaa

118861 gaagaaataa gaaaagattt aatcaaaaaa gaagaggaaa ttaggaaaga tttaattaaa

118921 aaagaagaag agatcagaaa agacttattg aaaaaggaag aagaaagtaa aaaagtttta

118981 aagaaagcgg aggaagataa aaaagaatta atgagaaaag aggaagaaaa taaaaaggaa

119041 gcaatgaaaa atgaagtcat taagccagta gttgttcaaa cgaaggaaga aaagataggg

119101 aaagattcta atgatgtgtt aaaaccaaac ataagagaaa taaatgttct tggtaatagt

119161 aaatcaaatg acaagaatgg atttgttttg acacatgtac ttgatggtta tgttatacaa

119221 gaatctaata ttgctttccc tgtaagtata tctttttaat tttcattata aagtatctta

119281 tattatagtt aagaagttac ttgaatttaa attcatcttc atggaatggc ataactagtc

119341 tttttatctt ttgagactaa actaaatctt ttaaatttta ttttagatac gaagaccact

119401 aaaagagaaa acactgccac caaacacagt cacagaagta aaacaagatt ccaaagaagt

119461 taaaactgag aatagcacaa aggtttttaa tttatccaat ttgaatttaa atgatgcaga

119521 agaaaagaag gatgcagaag ataaaaagga gaacgcagaa gaaagactga acgaagaaga

119581 aaataaagac tgtacagata ctgttagtga taaaggtaag aatagtggtt attttttaac

119641 tgcgagtttg tactaacaca gtttactaaa ttcaatacaa ttagataaat gcattttatc

119701 ccttacttat ttattaattc acaaataaat aataaaatag tacaataaaa gtgttacggc

119761 agtgcaataa ttttaaactc ccgaattctg aatcgattta taaagcttaa ataagcggtg

119821 cttaaaattt agtgaacgtc tgctagtgtt tggagtttaa gcgtggtctt ataatttaag

119881 acgttgttat atgaccacga aattctgaaa cgtcatttaa atatgtttaa atgtcgttta

119941 gaatttggcc gtaaaaaaaa acaacttttt ttaatatttt taatttgaag cattttagaa

120001 taatgcgtag ctatctacgg cggacggttc ataaaagttg ttacatttgg tctcctccta

120061 attctttgga ttattttttc ttagaaatct ctaaaattca taagaagaag ttcaccatgt

120121 gtgtttcttt acagataatc ctttcgcgga actgagaaca gagactgtaa aaagctggac

120181 ggtatgtatt tcgtttaatt atatcatacg cccaaataga tccagtagac ttttagatag

120241 gcttttatta tatttgatat tcaaaagaca cttagctgtc attactgtta ggaacacatt

120301 tgcactatga atgactatta atagtgcaaa tgtattttta actatgacat actctagggg

120361 aaataattag aatgggtaca ggaaataatc tttgggtatg gaccgcattc tgtggaaggc

120421 attggggaag ggctatgcct tctacaaaat gcatgtccag cagtgggtag agaacggttg

120481 atgttgatgg aagattagaa aggagctaac aagccaaata ctgaaacaaa tcttaaatta

120541 gctctgccca caatctaacg aacatcaaaa tttgtatgga atttggaatt atagtgtgtt

120601 ctttagatgc tgtaacatat ttgacgtttc cgtattcgct aaattataga atgtcactaa

120661 ttaatgacat ctttcaggta aaagaattga cggaacatct tgtgaaatac cattgggatg

120721 aaacagtgtc agttttactt gaacatgaaa ttgacggaga atcattatat ttagtttcaa

120781 agaatcaatt ggttagtatt ggtattagtg aggaacatgc tgatattatc tgtgattttg

120841 ttaaaagata aagtgatata gtacagacaa aagtgcaaaa cttatatgat aaagtgcttc

120901 aagaagtggc gtacggacag tgcaaacaaa tgtatataaa ttaattttaa gtttttaata

120961 tgtgacaaga tggtgttcaa agtgctacta tatctaactc tatggcttca ttaaacagaa

121021 ttcgcgataa aaacgtagat ttttcttcgg agtaagaccg tttcttgtga aacatctgtc

121081 taatagagcc gtagtgtaat gatgaaattc tcatgtgaag atattgtttc aatatcttta

121141 aatgaacaga attagccttg tgtacgtcag taaaataagc aatgtaaatc tgcaatttta

121201 gtatctaagc aacttttaca atataaagca aggcttacac tacaccgtga catggctggg

121261 atatgacatc caggccagcg ccgtcagtgt caatacaaaa tatacaaatg tgcccgagcc

121321 atgtgacgta tagcatagtc aaaatatgac actgactgca cgggattgga cgtagctgct

121381 cgccccgggt cactgacctt aacataggtt cgtagacaag caacgcgacc acagaatagt

121441 gtagcttaga gaatgagggg cgtaaggaca ttggcagtaa aatttcatag taattaatta

121501 acaatatttc tttgaactga ctttgatgtg ttcattttca tatttttaag atcttggata

121561 tagctcaaat aaagcattca ataataaatt taaactacac atagttaaat agaataaatt

121621 acattgcaca taaaatattt aaattattag tcttgattct aaaataaatg tactgaaata

121681 tattgtggtc tttgttttta aaaaggacaa gatgcaagtt gccagatggt caaggtttta

121741 tctaggggga gggtggttag taaaaagatc ttttaatccc aagggtaatg cttattagct

121801 atgcaatgaa aaactcacaa gtagtttttg agttatcagt gtgaatataa ggggattgca

121861 atcatcttag tatttcaaag tactttatac atctgtatgt acattttgta acttatctaa

121921 ggctgccctc tctcgcctga tgacctgaat attcctgtac atgtgtttta tgcagggatc

121981 tattaaatac atcaaatatg ctatagatat ataaaaaact gaatatggga agccctaata

122041 ttctcctaat aagagatagc aattgtatac aatttgtaaa ttaaaaataa tttgtaagct

122101 aagttaatgt ataaaattac tgtgtaaata aattatttac cctacagctt cattagaccg

122161 agcacgtgca aaaaaaatgg aggtcttgct tcggcgtaag gccgtttttg gcgtgacttc

122221 gatctaatta agccttaggc ttagaaaaac ttgtcgcctt ttgatatttg gcgaaagttc

122281 aatagaaatg taataaatta tttttggtgc actattgtac atttaaagta ctttaagagg

122341 tacattttaa attaaattta gaattaacat tgagttcaat ttctctggct caataaatcg

122401 cgtcctttgt atgaagtgtt gttgacattt tttttgtgtg atctcaaaaa aagttaaaga

122461 caaattaaaa cattgtgaaa agagttggtt ttggtatttt atattcaatt tctaccttat

122521 aagagaaagc tccaagtaaa atttctaata tgaataaaac gagtcatgct tgaataggaa

122581 tattagattt ttttgttctt taaagtttaa attgcaataa actgagaaac aaatgaatta

122641 aagtattttt tttgatttta gtttctatct ctctaatcta ttaattatac ctaaaagctt

122701 aaaatattga taagtattta ctaaaatacg taccataata aagttcaatg gcagattatt

122761 gacaatgaca acaacatgac atttgggttt ggctgttagt ctatggttta tttgtgtcta

122821 tggttaatgt cgttcttttt taaacttgaa gaaatcctac aaaagtcttg gtattagtaa

122881 gtctaaaaaa atattgctcg gcacgtacaa ataggaataa attataatag aaataaattc

122941 tgtgataagt gttagtagcc accaactcag ttcaacacag caaggaacaa tttaacttca

123001 accaagacgt tgaggtagtt cttaaaactt cgttataaaa ctgtctccct taaattagtg

123061 ctaacataaa gcttttaatc agtgttaatt ttatagtatg gtcacttgcc actatattgt

123121 cggcatggtt gtacagtatg tcggttttac aaaattaaag ttattctgca taaatggtcc

123181 tttcattcac tgcatatgtt gccgccgtgt gaatgtaaaa atattcgatt tggtggtcgg

123241 aaaaatatac gaaaaacgtt cggagcaaag agcaatggtt tctgcgataa aataaatgat

123301 gtttattaga cgcttgatat ttagattgca atcaaaccgg caaataaaaa aaatattaga

123361 caatccaaca gacgagaaga aaatctaaac acatttttaa ttaaagttta caacaaagat

123421 acataaaaga taaaaaacaa ttgtacgtaa aaaataaaat gtagtcatgt ataaaaaaat

123481 taccagtgat aaaaaaattg tccacggtga cgcataatta gttcctcttt gtttttccca

123541 taaaatttac cacttgggct agtggcgcga actttcctcg gcgacatctt tttttcagca

123601 tcatttccgg cacttcttcc gactcgatca tggacgaatc catggttttg gagacgagct

123661 cctcggaaaa atcctcggtg tcgttctttt cgctatcaga aatctcccta tattttttgc

123721 tgtactctgc tttcgcgcgg ggtggcacct tgtcgcgtct acatttgggg tcgtgtacga

123781 attttgaata gtccggcgcc tggcacatta aggtttcata aaaaaaacga tccgctcttc

123841 cttccgcttt ccctcttcct ttaccgtgtt cttcatctga actttcgttc gaagagtgaa

123901 gggctggcga gccggttaac ttcttatgcg gtcgtggact gaaaagagta tatatttcac

123961 gattttacga tatgtcctgt gtggttcgag tagataggag cttaccatct cttgttatct

124021 ccgggcgcca tattaggtgg tgctcgtggt gattgcatcg gcggaaaccc cggcccagct

124081 tcatcaccaa caccctgatg attgttattt ttgtagttcg taattaaatt gtaaaacgtg

124141 atgtaacaat atgttttttt atcaacttac tttatacccg aaatggaaat aacagtgtgt

124201 gactttctcg acgtcggccc aggcgacgat gtaccgcgcc atgttgagcg ggattttcgg

124261 caggaagttt gaatggaact tgccttgaca atatatcgtg ataacatctg tatccttcac

124321 ttcaaagact atcctaaata tcgtacctaa aaatacgaca aaatgactaa aggcttcttt

124381 ggatttttaa ataatttaag tgtaataaag attaaatcgg ttatgaacta accaggtaca

124441 aacatgttat agttagtatc gtagttaccg ctgcctatcc agataccgtt tttccttgac

124501 agagaaataa tattgtcgcg atggaatctc acatcgaaat gcagcaccac gtcccttggt

124561 tcctcgcctt cttgagcgca taagttaacc gacatcctaa aataaaacgc ccatattatt

124621 tagaataagg gccattgagt acgagttttt gttcatttgg ggagcttcaa tggtaattat

124681 attggtattg ttataggtgg gaaccgtgga aaatagtatc agttcaaata ttccatttta

124741 gataggttcg gaatcaaatt tgagtcatcg caagctcatt gaatttcaat gcgacttact

124801 ttctggcgtt gtccttaatt tttccaccaa tttcaatttt gtcgccgact ttaagcaaag

124861 cgggaatatt tgccgtaaag acaggcatct tactctagga tttgcaccga tactataata

124921 tttcacagca gtcgtatcca gattagtgta aataatatct tttgacattt tgaacaaata

124981 atttaaacat aacagcgatg tttatgtctt ttgtataatt tattaatcaa taaatattga

125041 taccgaatgt gtaaaaatgt gacacctgcc acaaatcgta ttaaactata cgaaaaaata

125101 ttttctttaa acattaatca ttgcgaataa attcaataca aaaatagtgt tttgtatcct

125161 tgtcaatgtt tcgccgtttg aaattacgag ttgaaaatat tatacaatga aaatggcacg

125221 ccataactta aagttgtaat tttttagact atttttaatg attttgaaag aagaaaacat

125281 aaaaaattgg aactttttca gttggcagaa ttttaacgga atgtgtcaac tccacgtaaa

125341 tgtcaatgtc aaaaagatca cctacttatc aaacataacc tataaaaatt aaatactaaa

125401 ctgatacggt acaagtacaa ctcaacagtt taaatattgc aatcatctta aaaacatatt

125461 tatacgcgaa acttgatagg taagacgctc aacaagtatt ttgtaaaatt accaaccaaa

125521 gttgatttga atcacgttcc cggattcata cttttgtaga gaaataagcg attttaaaac

125581 gataaatgta ttattttctg ctaaaaaact gcgaaaaaca taacaacaag gtttttatat

125641 attagaaata attaaatgga tccaaaatga cgcaatatac atgtaaattt ttgcattaaa

125701 tatttctgac ttataattca attattttct ttaattgttg cctaaatagc caacttcttt

125761 attataataa attctcttcc ccatttttcc ttcttttgcc atacgactgt gcctgattca

125821 ttagtcttta atgaaattaa tttaatttta tatatatagt tgtgtttgca tatagggtat

125881 agagttatca gggaactgtt catttataaa tatttgctaa ttaacctact gcatgcataa

125941 aacatacaat caactgacac tgtgacagga tatttataga ctactaccac tttattctga

126001 aacgagaatc agcattatta ctgttgtact ttctacattt ggaactggta cattcataat

126061 atttattcca aaatacagaa catccttgtt cctggtactt cttatgatac aaaatttggt

126121 ccaatgaggc ccctgctcta tcttgtttag ttccaagtgt cctccagtat catataatca

126181 acgatttatt ggtcttcgtt tatcacagta cttcatacat tttatataga tttattataa

126241 tatactagcg tttgttcgcg acttcatttg tatcgaattt aaaataaatt tctaatatgt

126301 aacccatgtt actcaatgat aatttccaat ggttatagaa tttttgaaat tggacgtcca

126361 cgtggaatag tccggtaatg tgctattacg aggtttttgc tgttatgcca ttttcggcat

126421 ctacagacac taatacacct ttgatttttt ttccagaatt ttcctacgga tgtttacgga

126481 gatattataa aaatatactt gtttgttcac tcactgttct attgaagaag atgaacgttt

126541 ttttgctctc ctgtaaaatt ttaattttgc gattaccgga ttattcggtt tgggcgtcga

126601 attggtctag tagtctagtt ttcgagttta ttcattgcat ataataatac aattttttca

126661 tctttataat agcaaaggtg catattatta tgcaaagaac atagttactt tgtgacaatg

126721 tatagcctga ccaggaaaac ataaaaatta tgataagatg ccacaataat cataaatcac

126781 attaatatta aaactatgtg gcgaaaattc tataatattc tgaaacctgg ttaggttttt

126841 attttatatt ttttattctt gatataatgt aatatcaagt actgacctat acatgtttat

126901 attatgataa gtgtacatag cttatttaaa ttgataagat atatttatta atgacagttc

126961 agaccaatat atattttgct tgtgaaaaaa attgttgcaa tttagaattt tatttttaaa

127021 atactataac tgtgtttcat tgaaaaggct agttagttag ttagttagta gtaggggcta

127081 tttttccgca actccacgac ttgcgcgtat tttgcgtgag gacaggactt ggatcactcc

127141 agccaaccca gctctccaaa gaaacccagc agccctttta tgttgccggt gatctctggg

127201 agcgatcccg gcgcgccgag atgtttaacc ctgtaatcgg acactgccga gcagtgttgt

127261 ataatatgag aagctgtttc ttcctcctcc atgcacgctc tgcagagagg actatctgta

127321 atgcctagat taaataggtg tttattgaag tggccatggc ctgtaagggc agccactact

127381 atgcggagtt gtgctttgca caatttgagt aacctgatag agatacgtct attgattttt

127441 ggcagggctt ccttggcctg cctgcataca ctcaagttgt cccagagctc gctatgttga

127501 gcgttgctcc tgtttgttac ctgcgacctt agaaatcctg gtggtaatgg aacgatgggt

127561 tctggaccga ttgctcgggt gctggaacct ttgcgggcca gctcgtctgc tgcatcgttg

127621 cctctcgagc cgctgtgacc ttttatccat tggaggttga tattattggt tgttcctaaa

127681 tcattaagga ttaggtgaca ctcatgtatt aggcccgaag tcattatgtt actcttcagt

127741 gcctgaagca ctgccatact atcagtaagt atacggatgt tttcacccct aacttttctt

127801 gccgttatgg cgtgtgctgc taagatcacc cccatgcact ctgcttgaaa gatggtgttg

127861 tgaataccta acgaggtgtg tatttttatg ttcagatcgt ctgagaagac ccctgaacct

127921 gaaccttgat cagttttgga tccatctgtg tagatcctaa ggtcatttat tgaatgccta

127981 cttgtgtgtt catcttctgt gaggtgtatt ttgtactttt tatcgaaaat gtactgtttt

128041 ggtatcaggt cgttctttgc atctataatg ggtattgcct ccttgagtga gtccaaaata

128101 gatgcgtgtg ggttcctgct gtgtttccaa agttttagtg tacgcagcct taacgccgct

128161 gaggttgcta tttgctgtat atgaatgtgt aggggtgtga gatttaacat tgcttctagt

128221 gcagctgtag gagtagttct catactgccg gttatcgcca tgcaagctag tctctgacac

128281 ctttggagtt tgttacaggc ggtattttgt ttggtcttgg tccaccacac taccgctccg

128341 taagtgatga ctggcctaat gacagatgtg tatagccata gcaccgtttt cggtgttagc

128401 ccccatcttt taccaatcat tttcttgcat tgccagaaaa tgatgctggc cttgttaacc

128461 ttgttttcaa ggtgcttatt ccaattgagt ttgttgtcta atatcacgcc gaggtatttt

128521 acctctgttg atagttgaat tttggtgccg aatagcgatg gtaggttata ttcaccgagt

128581 gttctcttgt ttgtgaacat tactacctct gttttgtttg ggttgactgt tagttcggat

128641 tccttacacc agcgttctac tatgcgtaga gccgcgcgag tgaggttgca agcgatgttg

128701 gccgatgttg ttgtgatcag tatcgtcaaa tcgtccgcat atcctactgt gtaaaagttg

128761 ttcttgttga gaattgttat taggtcatct atcaccagat tccacagtag aggcgagagg

128821 acacccccct gggggcagcc tctcacgatt gttactatct gggagtcatt ggtaatgatg

128881 attcttttgc ttaacatttg tgtgatccat ttgaagattg ttggatccac cccatgattg

128941 catagggctt tttcaatgtg tacgaagctt gttttgtcaa aggctccttc tatatcaatg

129001 aaggtgccaa tgcagattga tttcctgtcc aaagcgtctt ctatcttgct gactactaga

129061 tgaagcgctg aatcagttga tttgcctacg ctgtacgcgt gctgtaatgg gtgtagaggt

129121 ttatgtttta ggtctacttc cctgatgtat ctttcgataa gtctttccat cgtcttaaga

129181 ataaaggatg ttagacttat cggtctgaag gctttggcat tggtgtaatc acctttgcct

129241 ggttttggta taaatataac tttgacttcc ctccagctct taggtacata tctgtatgcc

129301 aggcacgctt tcataagttc ggccagcctg tgattcaata gtttttctcc ccactgcagc

129361 agggctggaa agattccatc tggtcccgct gctttgaatg ggtgaaagct atcaatggcc

129421 catttgagtc tggaatttgt gattacttgc tctgcaagtt cccaatcatg ggtggtgggt

129481 gtgttggcct cttcagacca ctccctagtt ctacttactt tgcagtctgg gaagtgtgca

129541 ttcagtagaa ccagaagcgt ttcgtctggg gttgttgtgt acgtgttgtc gttgttgcgt

129601 aaggtttgtg gtccttcaag ctgttggctt gatagaacct ttctcacacg gtttgcttgg

129661 gcacagtttt ctatgccgtt acagaactta cgccatcctg ctgtccggca tttcctaatg

129721 tgctttttgt agtctgattt cgctattttg taagcgtccc agtctggatc atgggaggtg

129781 ttcatggctt tattcagagc tcgcctaact ttcctccttt ttgtgttgag ttctttgcac

129841 caccacggtt gttgaccggg ttttgcggtg aagattttct ctggacatgc ttcgtgatag

129901 ctgttgatga tgcattcacg gatgtcatga gcttgttttt ctatggattc tagcccagac

129961 cagtactttg gctgagtaga ttccaagttg tttagttttg cggctaggag cctagagtac

130021 aaactcttgt tcgttttcct agggttcctc ctaggttgtg ttgttgttgt ggtgtttata

130081 cataaagtga agcagatcca cctatggtcg gagcatgaag cctcatcgga aacacgccag

130141 tttattattt ttgacttaag gtggtctgtt gctaaagtta ggtctataat agtcctactc

130201 cttctagtga caaaggttgg ttcgttacct acatttaaaa tgttaaggtt tgtcgtaaat

130261 aagaaatcaa ctaagttttt acctctatcg ttgttcctgt ccatgcccca caattgatgg

130321 tgggcatttg catcggtgcc gattaccact tccaaatttg tacgttcgca gtggttgatc

130381 agcctggcga ggtttgttgg cggtgggtcg tctgcctccg ccatgtaggt tgaggcgacg

130441 acaaaaacac caccctgtgt gtcagtcact ttgattgcgt agttatctct gcaacagaaa

130501 tccgtcagcg gaagggcctg tatgcccttg gatatggcaa tgcaggccct tggctgatcg

130561 tgatttgaaa agaggttaag cttacccata atttttgata ggcctagaac cttgtttgcc

130621 ctaacccacg gctcttgtat tagggcaata aaatttgtgt ggttctccat ccacttccgt

130681 agttgagccg tggcggtttc gctgtggtgg agattgatct ggatgaagtt tgtagggtta

130741 ttggacgaag gggacgccat ttagacgctc ctttacatcc tcttcctcgt caatgtagag

130801 tcccgccagc attgcttcgg cgtcctcacc ctcggagagg gaggcacccg acagtgtgcc

130861 gagtgactct gctcccgcag gcgacgacac ttgttgcaga cctgacgggc ctggtttgtt

130921 ctcagggggg tcagttgtca tgtcttcccc gctgttgtgc tcggtagttg agctggtggt

130981 cggctcattt gatggctgcg cttgtgtgct tgagcaagtt gcctgatggt ctggcgttct

131041 ggtctggggt gtttcataat atttgccacc cttgcccaag aatttgatgt agcagtgata

131101 gattgcgcat gccagtcttc tatcacgctc taaaaggatg gggatatgcg cctcggggat

131161 ggtcagtttg acgaaccatc cgtccttctg tttctctcca tcgagggcaa gccactcttt

131221 gacatcaacc catcggttct gcctgaaaat taggcgccct atgtcgcgtg tgtctttgat

131281 gatatcatac tcatctggga cgaacatacc acatctgacg cgccggggaa gctcggcctg

131341 tggtttgacg atgaactgat ggccttggca tacgtgcgga tcttctgcca tctttttgag

131401 ccatgtcagc gtgagttgat tttcgcaggt aaccctgatg gctccctcga cgaaaattgg

131461 tttagtggca agtactggta attgcgtatt gtcgccaccg tccggtgtcc aggcggagtc

131521 cagcattgcc ttcattatgt tcttgatgaa ggtggtggct acctctttag tgaggctgcc

131581 cggtcctgcg ctagtaactg ccaccctgag gtcggaggtt accgctgcgc tatagctccc

131641 cgctgctggt ctgcggggtc gctccacctt ctgtttcttg tagtcacctc ttggtgacgt

131701 tgaggagttt agggctcgtt tctcggcgcg ggtttccttg ccaggtcgct gagtcgccat

131761 ttccttcaca ggtaccgttc ccttgccggc gacagcggtt ggctctggag gtctacgacc

131821 ggaaccagat accattagtc cactggggcc ggcttggggc ggcccttgat cgggggtagg

131881 cggctctgta accctaggca tctcacctct ctcagccctg gtttttgcag ccctgagcct

131941 ttgagtctta gttggccgct tccatttctt cgccttagaa gcaggatccc tggaagaacc

132001 agtgggagtc gactggaaag tcctcttcgg cgttggtttt ttaccatgtt tagggaccac

132061 ctgccaggca gctgacaggt cgtcgtctat acgtggcggt gatggcggtg gcgacggcag

132121 cttaggtgtt ggtggtggtg ccgccatggc agcatcggtt ttcgacgcgc ccgctgaagt

132181 tgctggtgat gatgcagccg gggctttatc atcccttgtt tgggtagagc ttttgctcga

132241 gcctgccttg gcaggttgtt tggttgaggc gtgggatgaa ccagtcgcct ttgtagtttt

132301 tggtttattt ggccttttgt tatccattag cctgtcttaa aaagttcgtc cccccagctt

132361 cccaccctcc atggagcccg cacatctggg aacgccttac tcaaaagaaa ttcgggcgtt

132421 aggactactg gggagatata ggggggcagg gttggctcac agcgcgacat cgggagctcg

132481 atcgggttgg cagctagatg aaaaagatcc agcttgacca tttgcacaac cgtcacttac

132541 cccgtcagca tgacctttac cgccgctagg tgctccgtaa cacctagcgg accccaccca

132601 cctcgcctgc ggccctctga cgggacgcgt ggccatttcc ctgcctgtgg ctagcaaaca

132661 gggctaccgt tttgcttgaa atccggacca aagggggagt gttgcctaac gccactaaat

132721 aaacccctgt gacgctcggc tcagcccccc aggactcctt catccctgcg tacggcgctc

132781 gactgcgcag gacctagcgc aggtgggttt gtatgcaccg ccggttaaac ccacaatcac

132841 cgacggcaat tcgagtggtc ctctcggccg tgactagaga gtaggtaatg gtaccagtct

132901 ctagccacgt tgcccggggt gcaccggggc agtgggtatt catcttgttg gggtgttaag

132961 tccgcgagga tcaataccca ccacccacgc gaggaggtgg aacacactaa tagcccccaa

133021 aaggctatct atattctaaa gtggaaagat ttgtaatttt gcatgtttgt atttttatat

133081 gtattcaaat aactcaaaaa ttactgtagc aatttcaaaa aatctttcac cgttaggcta

133141 tattttaatt taggctatat tttttgattc cgcgcaaata aagtcacaag caacagttag

133201 tttaatgtat aaaaataata attatttata ctttggttgt ctctagtgat acaatatggt

133261 ggtgtcagaa gaaacacaac ccactgtaga aggggatgca gctataagta agaaagcggc

133321 aaagaaagca gccaaagctg cggagaaaca acaaaagaaa acagagcata aggtaaaatt

133381 aaacactttg taaacatact agcgacttca tttgtatata atgaaataaa aaatataatt

133441 tatgttactc gaagataatg tagccttcta atggtgaaag aattattgaa attgactcaa

133501 tagtttccga gttatttcaa tacatataaa aacacaaatc tttatttaaa atattagtat

133561 ttagtataga tatagattat tactactatt actccgctgg cgcagcgacc caaagtgggt

133621 cttagcctct gacacgagag aacgccagcg tcgcctatct tgcgctgtct cgtgctcaac

133681 taaaggcagt tgatatagat tattactgac ttataatata cagatgccta ttttatgatc

133741 ccaaataact gtggtctttc accttttact cgggaaggtc tgagagcctc tgtaggaaat

133801 gtttaggcta aagagaaaat agaatgtctt attaacattt tacataaata gcaaggtata

133861 tttcaagata ccttgctgtt tctattgctt gatatgttgc tgtcctctga tgccatttag

133921 caaaaaataa acggcagatt tgaacccaca acctttacta gttatcacac taaactacta

133981 ggacatcagt actactatgt ttttgtagca tttttatgta tcctcttcgt atccaggcag

134041 catcaggtca gcaacccaca gaagcacctg aatccgactg ctcggaaggt cggtatggag

134101 tccgcaagct gatacaatca tcaggggagc accgtgacag agtttacact gatgtggtgg

134161 atcttggagt tgcgttggat ggacaggatg tatgggtcag aggtgaggag cttatatttg

134221 ctgatggcct ctccggagac agggggtgtt ttcaatggtt gaaattattt taaaaaaatt

134281 tcttcttgag ggctacagtt agaattttta tttattttaa actagcgttt cctcacgact

134341 tcgttcgctc gtaattcaat aatacaccct ttgttattca gtgataatgt agctttctaa

134401 agccttgttc gcaccaggat agtattttag tcgagtagaa agtagcattt atccgaacaa

134461 aggatttact actgtttgga ctagtttaag cctaaaccta cgcactggga tagtaattta

134521 gtaactaaac gtgttcgaac agcaacattt tagtagaata gattaacact agaaagacgg

134581 gaaaaacgcg ctacctacaa agacggccga tggtcatatg acacttcagc ataacaaaga

134641 ctctaattat gtatatgttt atactcaaat taaataatat ataacagatg ataggaaata

134701 tattgccgat tacagatcaa ataaaatatt tttcgtattt atgtaatttg caataaaaaa

134761 taaatatttg aagttatgcg gtatcgtcat atttgcttag ttgccactgc tatagttgac

134821 tttctgaaga gttacaatct ttgtttttcc tatttttaca atgtctttga tgacttctgt

134881 catctcacct gcagcagcat tatgcagtga taaaaattac agtatagttt cagcagtgga

134941 attctttgaa aaatgccttg agtactacct ataatagcta aacaaaattt tttctttagt

135001 cttttttgtt tatttataag tacatataaa gtttttacac ttagaccttc actccagaat

135061 aacattagat aagggtatac aaaagcaaaa aatatatatt accacgcaat aagatctcac

135121 cctaacaaat tttgacttct gttcggcctc gtatgaccca ttttgaagta tctatttatt

135181 tctagaatag accacagggt tatatagttt tattgtgaaa gctattatta taacctgata

135241 tactatcacc aggtacagtt cttcactaaa acgattttca ctagtttatt gttgagtggc

135301 agctaaagac ctggttactc actgaacggg gctaagaagc tacagaagta tctgaatttc

135361 tatccctaag tttttatggc aatgctacat cttcaataat aatacctaat tggttttctg

135421 ccaacaattg tagttatatc aacattagtt ttacacaaat acacgaggta acttttttta

135481 gaaatatgaa ctataaacag aaatattgat acaataaaaa gtcataagcc cccaaaatat

135541 aatctgaatg atgtatctaa atgtgaaaaa cgatctgtta ataaaattaa tgtaaacatt

135601 attatcctaa ttacaataac aatacatacg tctgtcaagg tgtcttatga ctggcgccgt

135661 ctttgtaggt gaatgttatt ttaattcata tttctataaa caaagtacag tctaaatatt

135721 tttgtctgca ttacaatcgt aattgattaa aagtcacgaa atattaaact gtataaacaa

135781 atagggacga aattatcagc gtttcaattt caaaacaggg tcaaatgaca ccggccgtct

135841 ttctagtgtt aaatctgttg ttcggatact tgctacttgg tactcaacta aaatactatc

135901 ctagtgcgta ctaggcttaa tggtgtaatt tttgaaatcc tcccagtagt ttttgagtta

135961 tttcaataca tacataaaat agatatcttt cctctttatt atattagcat aaactagctg

136021 ttgttcgcga cttcattcgc gcgaaattta aaaaatacaa cctatgttac tcggtgataa

136081 cagcttttaa atggtgcaaa ttttgatatc gacccagtag tttttgattt atttcaatac

136141 atatagaaaa tagaaaccat tcctctttat aatagtataa ataaggaatt atgttatgta

136201 ttttctacat acaatgttgt tagcgcggct gcacacgtcg cgtgctaaag gcaagcagtg

136261 cttcgctgtg ttgcggcaga actccagcac ggtgcaactg ctggtcagcg tcaatgagga

136321 gaagaatgtc agtaagcaga tggtcaagtt tgtcggcagg tgagtacatt tgtgaccctt

136381 cggcttcatt agactcagtt cacgcaaaat accttagtca aagctaaatc gttttttttt

136441 atcgcgaact cgttttgatg aaagcataag gtaaaaatat atttaactag ctggtgttct

136501 tgacttcgtt gttgaattta aaaatatagt ctatgttaat gtttaatttt tgaaatcggc

136561 ccattagttt ttgagttaat taattttatt aagagttagt taaggtgatt tcaatgcaga

136621 cataaataca aaatattcct ctttataata ttagtacaga tatattagta tgttttttta

136681 acttaacaaa agtttcgatt ttctttgtgt tttcgttaag tttcaaaatt taattaaaat

136741 tatatagtcg gtacaatttt ttttttattt ttccatctaa cacatttaca tacacaataa

136801 gtattttaac acattgttga acggtaacga gttaattggt gtttgtagcc acgaataaaa

136861 tcgaatctta cctgaattgc cgatcaacaa ttttcaacaa ctttcgttaa tagtattttt

136921 tatttaaatc tagataagat tatatcaaat atgcgaacgt ttctttccgc agtataacaa

136981 aagagtcaat aatagacgta aaaggtcgtg tggtgacgac tgccaccccg gtggagtcgt

137041 gcagcgtgcg cgacgcggaa ctgctcgcgc aggaggtgtg gcaggtgtcc gcggcgcgcg

137101 cgcagctccc tctgcaaata gaagatgctt ctagaccgga gaagaatgac gtgagtgtcc

137161 actaactcta gccaggagaa gaataatcgg agtacggtag gagaggactc ttaggtcaaa

137221 gatatttggg tatatcctta catagtataa aacaataacg cttttttatg tttgtcccta

137281 tatatgcttg aatctttaaa actacgcaac ggattttgat gcggtatttt aatagataga

137341 gtgaatcata aggaaggttt tcatgtataa tacattcaca atatagtaga gtaacactga

137401 tcatttagag gtttttaata ttatgtcgta aattaacaca tttttagtgc ttacattgca

137461 aacgctgcag tgtttagtat cagcattata ccgatttgcg cccgtgcgaa gccggggcgg

137521 gtcgctagtt ctttataaaa ctcacttatt atattttaca agaagtattt acattgaagg

137581 gcggctctct aatctctcta agatgtatgt caaacttcta aaagaattta aacaatagaa

137641 ttataaattt taaattttct ttataaaaaa actaaataca aaagaggtaa catccctaca

137701 gtacagttaa acctggataa gcgaataatt ccctgatccc gacgagctgc ctccgtaagc

137761 gagaaactcg attagagaga aacctctata aacgagacaa atatcgcggt cccctggacc

137821 cgcgtttatg caggtttgac tgtactatac taaacaaaaa actgtcaaaa ctttactact

137881 aatactattt ttctagaaac tatgattttg gtttagtttt ctatgaggac aattttatgt

137941 ctgcaggacc cggaggctct gaagatcagg gtgaaccagg acacgaggct ggacaaccgc

138001 atcctggacc tgcgcacgcc ggccaaccag gctatcttcc gcatcgaggc cggcgtctgc

138061 agactcttca gagacattct catcaggaaa ggtcagcata ccttatccat ttgtattaat

138121 aatttaattg gtttttttta tattataaag tagtaaacga gcaagcggcc acctggattc

138181 gctgaaatag cgaagcaccg ctgcccatag acatctgcaa ttgcagatgc gttgcctacc

138241 cttaatctac ggaagaggag acacacagca aggggataat tcttcttcct atatgtctcc

138301 ccccggcaca tttacttctc cttcccatca tttcttttca gaacatgata ggaagggtaa

138361 gtgaactaaa atgaaacctc cggcaccaca ctcgtcagac tgtgcacgga atagcttcaa

138421 tttgacgcct gtcttctgtg ttgtggtatt tcaccagacg agtcggacaa ttcgtacaaa

138481 gatgttgaaa cggactctac cactgttaaa attattatta ctgaagtaaa acatcgtgat

138541 gcctgtacat atcggcgaag aaattcgaag atatgtgtat tcacgcaaaa tacgcgctag

138601 tcgtggagtt gcggaaaatg gagcccatca tggctcgaaa cctttttttt ttaatattct

138661 tcatattaca gtttaataca gacagataat tatcgtcgtt ataacaggtt tctataatta

138721 taaaataatt gcaggtttcg tagagatcca cacgccgaag ataatatctg cggcgtcgga

138781 gggcggcgcc aacgtgttca cggtgtcgta cttcaagacg tcggcctacc tcgcgcagag

138841 cccgcagctc tacaagcaga tggcgatcgc tgccgacttt gataaggtat atagttgtaa

138901 caaatttaac ccttaaatgc catgttgcca aatgtataca ccgccaaaga tatattatac

138961 tgtagatgga acatttgaag cctaaaagtg aggcattttc attgtcctcc ggaggtcttt

139021 cccctagtgt tgtccattac gttatatttg ccgatataaa aagtcgaccg tatctaacct

139081 cttttctcaa atttatattt tatttcatgg tatttactaa agtttaatat tatgattaat

139141 cgagacattt ttaaaacatt aaaaaaaaat tataacaacc acaaaacaaa aatagtcttt

139201 gttcattgtt tttttggatt taagttgttt taaataaaat aataataatg aaaaccgata

139261 attaacattt taatgtttta gtttattcta atgtttattt tataaattaa aacaaaaaac

139321 atattttccc aaacttggga aaattttcat tatactggca aagttttgat tttttttgta

139381 atttcttacc cggcacggat atagctgtca aattactatg gagcgctccc tctatgtata

139441 atatctcttt gcataccacg tttcacgtta tttgacggtc gacaagttta taaagttaaa

139501 agaagcgtct ccgtaatttt gtattcattg atatacgagc gctatttctt taaatataaa

139561 taaaaatatt ctcaactctt attatgtaag ttattatcat attttagatc tgtataaaaa

139621 taattataaa agtgtaagca aatgtctaca ttatgcgttt gttgcatatt atgtgcaata

139681 aaaaccatgc atttaggggt taacccttat tttaaaaaaa gggtatgcag ttaagcatac

139741 caccgtggct cggggaaaga cacaatgggt atgtgggact ccactctgcc cgtgagactg

139801 ggatactcac aaaaccaatt gtccaacgag acttggtctt ggatggtgtc gtctaatgat

139861 accccctttt aataattgtg tgcaggtgtt cacggtgggc gcggtgttcc gcgcggagga

139921 ctccaacacg caccgccatt tgacggagtt tgtaggtctg gatttggaga tggctttcaa

139981 gaatcactac cacgaggtgg tgttcaccat cgctgataca ttcacagaga tattcagagg

140041 actgcaggaa cagtgagttt gttttttata gtactttgtt tttttttagt tttgatctaa

140101 ttttttgtat tccgttctgt actatttact tatgggtaaa tttaatagaa atatgcaaaa

140161 aagttaatat tttgaaattc ccagttatta atcactaggt gtcgcccgcg tctccgttta

140221 tgcggagttt ataaaaacat agtaaactat aatctttcaa attatgtttt atatcagtac

140281 caaatttcat cgaaatcctt taagcccttc agataccttc aaacaaacat ccatcgtaac

140341 attcgcattt ataatatgac tagcgaccag tcccggcttc gcacgtgtgc aatgctgata

140401 ctaaatacac taccagaaaa actgaacgtt atgtgtaaga gccataatgg gtccctagtt

140461 atacatataa accttcctct tcaatcactc tattaaaaaa accgcatcaa agtccgttgc

140521 gtagtttaaa gatttaagca tacatatagg gacagagaaa gtgactttta tactatgtaa

140581 tattaaagtt taaaggtttc aatccatgat gggctccatt ttccgcaact ccacgacaag

140641 cgcgtatttt gcgtgaatac acatatcttt gaatttttta gccgatatat gcatttcacg

140701 atgttttcct tcacggattt tgatttggca cgtggctaac aagagtatta gtctatccgg

140761 tgtgccaact agcatatgta tatagatacg cgcgcgagat agcggttgtg gggcagcagt

140821 tccgcgtgga gccgttcaag ttcctggagc cgccgctgcg tctggagttc cctcaagcta

140881 tacagatgtt gaaagaagct ggtaaaaaca aattatattc tttgcataca ttttagatcc

140941 ccctatataa tcatatgtat tttgtagtta atattagtat agaagataca tcttaccaaa

141001 gagatattat acatagaggg agcactccat actaatttga cgttaaaaag cgtgccaaga

141061 aagaaattat taaaaaaaaa cttgccagta tcatgaaaaa aattcgcatg tttgggaaat

141121 tgtgtaattt tctttaaatt tttaaaatat acagtaggat aaactaaaac attcattaaa

141181 acgttaatta tcgtttttaa ttataattat tttatttaaa acaatttaaa tccaaacaat

141241 ttttaacaaa gactattttt gttttatggt tgttatacat ttttaattag cgttttaaaa

141301 tgtctgtttt aattacaata ttaaacttta gtaaatacta tgaaataaaa tttaaatttg

141361 agaaaagagg ttacattcgg ttgacttttt atgtcggcaa atgtaattta gaggttaaca

141421 cttagggaga tttccctaca ttggacctcc ggaggtcaaa gactgtctca cttttaggca

141481 tcgagtgttc catctacagt ataatatatc tttgtatctt actttaaaac ccccctccct

141541 ggaggtttct ggacctctct ccgttttcaa aatgtttagt ttattaaaat atagtcgaat

141601 taagataaac gcaagtcaaa gagacagtga tattttcctc gcttctagat tttggcttat

141661 ccgaggttct ccgcttatag aggtcgtcca tcaggaccaa aagtataaaa cttcaccaaa

141721 ttgcacgggt ggtgaagtca aaaaactaaa aaaacctcac gtaatggacg cccctttatt

141781 tacattgttt gctaatccat attcaactgc atttgactat aaatttcgac tattggccct

141841 agttaacata atttagccct tttgaattaa aaaaaaacat tatatcaggt gtgaccttgg

141901 gcgaggagga tgacctatcc actcctgacg agaagctgct gggccgcctc gtgaaggcaa

141961 agtacgacac cgacttctac atcctggaca agtacccgct cgctgtcaga ccattttaca

142021 cgatgcctga tcccaacaac ccggtaagtt gtcaaataaa acatcatcat agtaaattgc

142081 tatttttttg ttactggtaa cacttttaaa cagtattcgt acagggtgtt cccgtattgt

142141 ctgtttattt ttttttacca cagattcctt gggcgaaaac aagatgattt tacccaactt

142201 gcctctaggc ttgaaaacaa catgttttaa tttttttcaa gtatctcgaa aacccttaga

142261 ctttattaat attgaaacca ttatttttat ttgataaact acaccttttt taaaacaaac

142321 cattgttata aaatatacag ggtgctcaaa aacacgaata caatcatcaa tcatattttt

142381 gcaaccccct gtataatcgc agtcaattat ttgctccatt agaccgaatt cgcgataaaa

142441 atgatgagat tttacttcgg tgtgaggccg tttttagcgt gacatcagtt taaactattg

142501 agaactgaag ttgaaccaaa tgataaatac gttatttatg gttaattata gttgtaaggg

142561 cataaataag agttttaaga atttttttaa tatatttaaa aatatttgac aaactttttt

142621 tactaggtca cctcttggta ttagcgacaa ctatattact ttatttacac taaaattgtt

142681 gaaactttcg tgagacatta atatacttta tttatttaaa acggctaaaa actcacgtgt

142741 aattttatgt gaacggtatc gactagtttc gaactcttcg gataaccgaa gagtataatg

142801 tctctcttat ttacactgtt tcagcgcgtg tcgaactcgt acgacatgtt catgcgcggc

142861 gaggagatcc tgagcggtgc gcagcgcata cacgaccccg acttcctcgc gcagcgcgcc

142921 ctgcatcatg acatcggtca gtacatatac agacaaacct ctataagcga gaaactcgga

142981 taagagagaa acctctataa gtgagacaaa tatcgcaatc ctttggacca tccaggttca

143041 aatattaccg ctggtatgta tgacactggc agtgatattc gataggtcat gtgcttgtca

143101 cgtgtaggat atcattgtca gtttcataca tttttgacgt tcgatataga atcgcttcgt

143161 agcgaatgta cttttggtaa cccccaggtc ttcctaaatc catggttaca agttgaacta

143221 aattatgaca gctcactagc gtcccctatc aatactaaaa tatattaaaa cacgtattgt

143281 aaaatatact aaaatatgaa atataattgt tgttaaatgt tatcttccca gacgtgagca

143341 agatcgctgc ctatatagaa tcgttcagac tgggctgtcc gccgcacgca ggtaaattat

143401 attctaccct acggctacat tagacagagt tcgcgataaa aacggagatt ttgctactgc

143461 gtaatttcat ttttatcggg tactctgtct aatgaagccg aggcctaaaa gattttcctt

143521 aaccctactg tttcattaga ccaaaatcgc gattaaaata gagattttgc cgtaagtccg

143581 tgtatatcgt gaactttgtc tgatgaaatc gtagatatga gactaatctc ttaatccggc

143641 tacaaacgtt cagtttttta aactttccag ttgaaactga cgtgcacact cgaactgtaa

143701 gttctcagtt ttgcttacag tcagttttag ctgcgcagta gaactacagt taaaactgag

143761 tttccgtaac cgtcttaagt aatatctggt attactatta aatatgttaa attcacagtc

143821 aatatgaatg ttcaccctta agtgtcttaa cactgaatgt cgaaaaacgt atgaataatt

143881 aggaaaagtg gaataattta aaattttgaa tatattaaag aaggaagcaa gtgccttgac

143941 ttctttttaa tggaggtttt agtcagcatg atgactagaa actaacggct tcgttacagg

144001 ataggtttaa agatttcgac ccatgacggg ctccattttc cgcaactcca cgactaccgc

144061 gtattttgcg tgaatacata tacctttgaa tttcttcgcc gatatgtgca gacatcacga

144121 tgttttcctt caccgtaaga gcgtcggtaa atatgcacat atgaaaatcg aaaattgaaa

144181 aacacattgg tacatggcgg gattcgaact cgggacctgc agtttacaag tcaagagcta

144241 aacccctgag ccaccgacgc tctatataat cgttacagaa ataaatattt aatacttctt

144301 aaatgtattc tccaggcggt ggtataggta tggagcgcgt cgtgatgctg tacctcggcc

144361 tcgacaacat ccgcaagacc tcgctgttcc cgcgcgaccc caagcggctc acaccttgaa

144421 cttacttata aaatactaac tacccgcccc ggcttcgcac ggctgcaatt ttaatactaa

144481 atacaataca gaaaaactgt gaacgtcgta acagccatac taataataat agcgcctgtg

144541 cgaagccggg gcgggtcgct agttatacat ataaaccttc tcgtcaatca ctctattata

144601 aaaaccgcat caaaatccgt tgcgtagttt taaagattta agcatacata tagggacaga

144661 gaaagcgact ttgttttata cactatatta ttatgtagtg atacaataat aaatgtttta

144721 tataccttta caaagtcttt ttatttataa cctaaaatat tacaatttta agtataaatg

144781 actcctacaa agtatgtctt ctgttttttc aaactcggtg ggtatgcacc gcttctgtat

144841 tgcaaaaaat agcaatatgt ttttaaattt ggctaataaa taaaaaaaaa gagtgatatt

144901 aaagaatata ttcttgtctt ccgacaggct cgaaactagt cggtaccgtt accggatttt

144961 aataacgtga gtgcagccgt ttttatttat caactagcgg tcgcccgcga cttcgttgac

145021 gtggaattta gaagttcaaa aaagtttttc tgaaaattgt aaaattctta aatggcatag

145081 ttaatttatt catgctgaaa agcatttatt ccaattttca tgcaattctg aagaaggaat

145141 aaagttgctc tatatgaact ttggaccctt atttcggacc ctaaggtgtt gaatttcgta

145201 aaatcctttc taggagcttg tctacgtggt aacagaaacc cctgaccaaa atatcaagtt

145261 tctaagtctc gtatctacgg cgtaattttt tttttagatt ttatcccctt tttttgtctc

145321 ccaaagtaaa aacttgtaaa attcttaaat ggcaaactta tttattcatg ctaaagacca

145381 ctcattccaa tttttatgaa actctgataa aggaaatact tttcttcata caaactttga

145441 acccccattt ctgaccctta ggggcgaatt tcgtaaaaat ctttccttta ctcacgccta

145501 cgtcataaca cccacctgca tgtcaaattt cagccagaac cgtcctgggg tttaggctgt

145561 tcgttgacag atatctatgt cattcagtca cctttgagtt ttatatacag ggtgtcccaa

145621 aattcaacga taagccagtg ccagtgcatc accagattag tacctgcata gggaaaatta

145681 ataaaaaaaa tctaactctt ttagtttaaa agttatgtta acttatttga attgaattgc

145741 tctttgatac ccgaccccat tcaaataccc actgtctctt gagctataaa gatatccctg

145801 cttttttatt tgtaatggat agcttactaa catagctaat aattgcagtt gaaaacggga

145861 cctaaatatc tgtagtttaa gtacaaaaaa ataattactt taactgtttg cttgaaattt

145921 tcgaatattt taacttttca tcaactttgt catgctaccg tgaaaaaaaa atgtatggag

145981 ggtaccgtgg catgttgtta caaaagttgg cgcatctcat caagatttta aaatggtaca

146041 acgttatggt agaaacatgc aaaaaccctg ttaatatttt tttcaagtac tgaaaaggat

146101 tattttgcat agtttcaaag aactttccta agtgtttaca caagttttaa gttacagatt

146161 cgtgtataat tacctttatt tacacgtgtt ggggtacgag cgtgttagat actattgata

146221 agtcggagtg aaacaggtag taaagtcata ataagtggac cccctggggt actttatttc

146281 ttaactttaa tgaaataaaa taacaattat gagtttcatt taaaccatca tgagcatcac

146341 caccaccaac tagacagaat ttaatcgaaa taattagttt ttgtttcttc tctactgtga

146401 ttgtgatggt gatgatgatg ttgcactccg acttattaat agtaggtttc taacacgcgc

146461 gtacctcaaa acgtgtaaat aaaggtaatt atacacgaat ctgtaactta aaacttgtgt

146521 aaacacttag taaagttctt tgaaacgatg caaaataatc cttttcagca cttgaaaaaa

146581 aatattaaca ggctttttgc atgtttctat cataacattg taccatttta gaatcttgat

146641 gagttgcgcc aacttttgta acaacatgcc acggtaccct ccatacgttt ttttttcacg

146701 gtagcatgac aaagttgatg aaaagttaaa atattcgaaa atttcaagca aacagttaaa

146761 gtaattattt ttttgtactt aaactacaga tatttaggtc ccgttttcaa ctgcaattat

146821 tagctatgtt agtaagctac ccattacaaa taaaaaagca gggatatctt tataggtcaa

146881 gagacagtgg gtatttgaat ggggtcgggt atcaaagagc aattaaattc aaataagtta

146941 acataacttt taaactaaaa gagttagatt ttttttatta attttcccta tgcaggtact

147001 aatctggtga tgcactggca ctggcttatc gttgaatttt gggacaccct gtatagattg

147061 atgatgtgtg taggcataat aaaactatac agttaaaaat acaataaaac cagccaccga

147121 cgtttcgttt tttaactgtg cattttaaaa ctaatgcccc ttctgcctac acacgtttag

147181 ttttttagtt gtaatctcgc ttacattgaa gtaagacgtg cacggaatca aaaatttaaa

147241 agaaattagc aattattttt tgttttattc tacacaaaga aaaaagtttt attttaaata

147301 attacctaac taaataaagc caaatcttct ataactgtaa agttaaaaag taagcgtcat

147361 gtgtgcaaaa ttttatcgag atcttttcag aaattatgca gataccttta aacatccata

147421 catcgtaacg ctcgcattta gtacttagta gttcgaccaa tgatgggctc catttacctt

147481 cattccacga ctagcgcgta tcttgcgtga cacatatctt tgaatttctt cgccgatatg

147541 tgcagacatc acgatgtttt ccttcgcagt aagcgtcggg taaatatgta cttatgaaaa

147601 tcgaaaaaca cattggtaca tgccgggatt cgaactcggg acctgcagat acaagtcaac

147661 agctaaaccc ctgagccacc gacgcttcac tctcgcattt tattattaaa gtttattttt

147721 aagtaggaaa aggagtttca aggagataca ataaattatg cacaaattaa tatattttta

147781 ttataaatcc tattaattaa gtaaccgtta catgattaag taagttaatg cttaatttac

147841 aaggaagttg agcactgcct aagtacatca caatattact ttttaataaa gaatcaagta

147901 tgtgaaataa tattcttagg aactgcttac caaaaaaaaa ttaaactaaa atagtgttca

147961 ccagaggacc tatcacgaaa agcgagaaaa tatacgcatc acacacacac gagcgttttt

148021 ttaacgcacg ataaaaagcg atgagataga tgtatttcca cgtatctgtt cacacggcgg

148081 cgttatttat tgtatatcgc gcagtgttgg ataagcgctg gtcgaactaa acaagagtca

148141 ttacgtgaaa gcgataaaaa agcgccaaca ccgacgttaa aaaacgtccg tgtaaggcta

148201 ataagtaaat ataagttttg tcggtgttat actttctcaa acattttcgt gataggcgtt

148261 ttaagcatag aagtcacgta gctcatatat aaattatagc attcgctaat gtttacataa

148321 aacatgtatt actattattt aaatacatat atagatagac atacatccaa acatgtaaaa

148381 aaaatatatt tttagtaaaa tttttgatgt ttccaacatt ttttaaaatt ctgccacaac

148441 aattaaaatg tctaacacct acggcttcat tagacctatt cacgcaaaaa acggctaaac

148501 gccgaagcaa aacctgcgtt ttttccgcga actcggtcta atgaagccat agagaataat

148561 tacatctgat ccatatattt taattattaa gtgtttaata ctatggtgtt atccaccgag

148621 gggttcagag ccagctctag ttagataaaa taaggccaaa ttgtgaaacg tcatttataa

148681 agataacagg gtcttaagac cacacttcta atgtctttta aattataaac cacactgtat

148741 atatctatat ttgtatttaa tgtctgtcaa tgattgaaaa ttatattaaa aatagatata

148801 gacactcaat ttcttgtact taacacgatt cgattatttt attcgattaa cactttttat

148861 atcctagggt caatatgacc agtccgtggg ttctaagacc ccaatattga agttaggaaa

148921 aaaatcactt acattttaaa ttcgtcgtac atattcactt tgagtgcgtt cactttagtt

148981 tatatggtta tataatttga cactggttta ggggaacatt tacacttata acacaaaaaa

149041 tacacctgtt gtctttacca acaaagttca gactgcacca acgaagttca gactgcacca

149101 acaaagttca gactgcacca acgaagttca gactgcacca acaaagtcca gcctgcacca

149161 atgaagttca gactgcacta cataggtcca gtctgcgcta acaaagttca gactgcacca

149221 ttgaagttca gactgcacta cacaggtcca gtctgtacca aaaggttcag atcgcaccgc

149281 aaaggtccga acttccccac tatcgtcgca ccgcacatgt tcaggagtcg ccgaagaggt

149341 tcttgaggtc gtgctcgtag agtgcgatga cgagcatgac gccgatgccg gaggcgagcc

149401 cggccagctg cagcacgcac tggcacagcg tgccctcctt gctgtgagac gtgctcagtt

149461 ccggcatctg gttagcgcaa accaaagcgt aaacattcaa atataacata tttgttatcg

149521 agacttaacg caaatgtatc tagtggtcta atgaccccaa ttttcttaaa ctatttagta

149581 gattcttgta tataataacc atagacataa taatagtgtt cgtgcgaagc tggggcaggt

149641 cgctagttat acatttatat gtataactag cgacctttct ttttaatcac tctatctatt

149701 aaaaaaccgc atcaaaatcc gatacgtagt tttaaagatt tacgcataca tagggatata

149761 gggacagaga aagcgacttt gtcttatacc atgtagttat atgttctatt aattgattat

149821 atttaccata tcaacgagtg cgatgtacaa gaacatgccc gcggcggccg cgaagagcca

149881 gcgcgtggcg gagggcgcgt ggcccgccag cacgccgcac accatgccca ccaggcacag

149941 cgccgacgac agcacgttgt accacaccgc gcgccgagcc gacatgcccg ctttcagcag

150001 gaccgcgaag tcacctaaga tatagatatc cacacatggt aaaagccatt ggtaccgaga

150061 atttcctggt agtttgggtt aatgtttaac gcttctagtc ccgagagtcc cttgtcctga

150121 gaatgcgtct ctacccacaa atgttaagcc cctgtagatg tagaagcttg agttgtgaag

150181 tatggcttgt ttccgtgtcc aatccaatgc tagttaacat acaaagataa aagataatta

150241 acattaagtt gaggtcttac ccaactcatg aggcagctcg tggcagagca ccgcaatcgc

150301 agtcgagaaa ccaccggcga tgtttgaaga gaacgccgca cctgttacaa ttagttcata

150361 cagatttaca ataccaactc gaagaaccaa tttattcata tcccgtatta ttcaaaaaac

150421 gttctaaagt aatattacct atcgccatgc cgtcagtgaa gttgtgcaag ccgtcgccca

150481 tgatgaccat ccaggctact gaagacatgg aggacggcgg cgcgtgcacg tgcccgtgcg

150541 cgtgcgagtg cccgtggtgc gcgcgcgagt gctccctacg aattacataa acgttactta

150601 aagctgtgca tacaaaatat atcaccatca aggggtcttt ttctaccttc tctttctttc

150661 attctaatat cataatcaac tcaatgtacg tcaccaccga ggtatcggag gctaccctta

150721 gttattggcg actaaatgta tcgaatgact ctttcactca cctcaaaatg acagtataac

150781 tgtcgccatc cttcaaatcc ttagcccctt cttctttgtg gttgatatta ttcatttcga

150841 cgaccgcggg attttcagct tttagtagcc agtctttcgc cgtcggctcg ttctctttac

150901 cttcacgatt gagtgcattc gacgagacaa cgctaacgga gttattgtgc ttcttctggg

150961 ccttcggact cggggggaga ccatctctca cgtgatgctc atcgtgggtg tcagtgtcga

151021 tctcatcata acagtaagga tactccgaat acttgtgctt acatgtcttc attgttggat

151081 cacctttctc gtccctcttg aagatcttca tgatattatt cgatgtggaa ttagatctcg

151141 gtttcccatc agaacattcc accggctcgt cttttagcac ccggactctc gacggcagtt

151201 tgtcctcttc caacttctgc ctccttttcc tccactccac gacaactgtt agaccttttt

151261 ccgtgaaata gaagaataca acgcccaaca tagccgcaag ccccttccac atgccgtcgt

151321 cgtgcgaaga tctgagctcg acgtcggttt ccccgtggct gtgtaattcc tccatcgggc

151381 tcattgcgtg tggcatcaag tgtaagagag catccccaca caacgtgccg acagctaacg

151441 cgactaagaa ttgtatcaga tgattgtaat aagtcttatg cattatagga ataaccgcca

151501 ctcccagcaa cccacaagca ctgatcgcga atatacttaa agatgaatac aaccaaactg

151561 tgtacatatt tttggaatac gcgttctcta aaggtttgag atctttgttg tctcttctat

151621 aaggtatgtt agcttctttg acacaaccgg atttttctag cgaggtgccg gccagcttct

151681 gatagagtag aatgggacag atggcttcta acgtgtcctg tgacagggag gtctcggctt

151741 ggtgttcaga ggcgccgtgc tcgtgttcgt gctcgtggtc gtgaggtttt aaccttgtgt

151801 ttactgtcgt cactagctct tcactgctta cacactgcaa atagaaatat tgaatcattg

151861 accgagccta gaagattgtt tcaagtatag tttaggggat tttcgtatca atacttacat

151921 taacaacctc ctctttggtt tcagtctcac catagaagta caatttctgc tcgactttct

151981 gcacaccacc ttcaactaac ttatgtaggt ctagatcttg aagcattcta ttaaaaccag

152041 tcatattcat acttgttgtg ctaccatcac cgtatatact gaagatctgt tccacatagg

152101 tacttgggtt tacatttctg acagctctct ttcttctgat cttgtccaat ttaaattgat

152161 acctctctct tgtgtctatt tgtttgccga ggttagttgt caggtgttcg ttgtgtttga

152221 catctggtgg atgcgtggtg tttttgtgtt catctacgtg ggagccgcag acgtgcgccg

152281 cgcagagcag gcagaacatg catactgcca ctagatggtg agccattgtt accttctata

152341 cctgataatt gttgcagttt tatttcgtta tggattttaa tccatcggtt tgttgaaatg

152401 ttgaagcgtc acgatgttaa catagctcga tctgtggaca agaaaaataa ctttaagtat

152461 tatttaatta ctgattgagc tattatcata agtcgtttga aattactaat cttatgatct

152521 atgctagtcg cacttataga ttaatttggc ttagattaca atcgcctaga tgcatgtgaa

152581 cattttcctt cctgaacaat aatcaatcta ggccaatatt ataaagagag aaactttgta

152641 tttttgtatg tattgaaata attcaacgac tactgttatt taagtcgcgc acaaagtctt

152701 cctataaatt aaatttataa cccaatcaaa tgatataatg atatctctgt attaagaaat

152761 tccgagactg taatacgata tgtcgccgcg taaaaatcag acaacgcttt atataaactg

152821 gaacgttagt gtcccgatat ggaaacttct cgaatgccaa cataggtagt gtctgctgaa

152881 acactgtcac gaaacatagc cttccttctc acactgcttg tgataattga gatagcgata

152941 tgtttctgta tatgttttaa aacagtggga attctgttag tagatctaat gttacgacga

153001 gggccacaca ttgcggggta ttatgtcaat tgctgaatat gcgatatgga acattcatac

153061 aagtaatagt tatgatatct ttatctttat agttcaactt gaaacctggt atagtctgac

153121 tcaggtagaa aatataatac tattagatga ttaaaggttt catattttga ttatatcgcg

153181 aacaagatac agtggtagag cccgctttaa catcttcgca cgaattgtcc ggctcgcccg

153241 gtgacatacc acgaccacac agaagacagg cgtcaaatga aagctattct gtatttcgat

153301 tgaggagtct ggaaccggtg gcctaatttt agtccacttc cccttcctat ctttttctta

153361 taaggaaatg atgggaaggg taagtggata tggcggggtg gggacgtata ggaagaagaa

153421 ttatcctctt gctgtgagtc tcttcctccg tagataaggg tacgcaacgc atctgcaatt

153481 gcaggtgttc gtcgcttcgc tatttaagcg aatccaggtg gccgcttgct cgtgcatcaa

153541 caagaagtat agacagagat cagtggtgaa tcaaagtaaa gtacaaaata ttccaaacag

153601 ttacaggaag ccggttaaaa atcctatgtt aaaacaaata tgatttcatt atgtctgtcg

153661 gtcacctgtc tgtcggtctt ggtgatataa caaaacgatc acgtctgtcg cttgtcaatt

153721 ttaacgagct caagtaacgc acattataat aaacactatg cacagacagc catattgctt

153781 tgttccacta tgatctaatg cgtaggactg ttaaatctgc tctgccttcc cccggtctgt

153841 gtaaattgtc ctttaagcct tgttcgtacc aggatagtat tttagttgag tagcaatcaa

153901 caagtatccg aacaaaggat ttactaatct aatctactaa aatgttactg ttcggacacg

153961 tttcgttact aaattacttg ctatttcact aaaacactat cctggcgtac aaggctttag

154021 ggtgattgtt caaaaaactt agcctaaggg ggggggtccc ttactcctcc tacttagagg

154081 gctacatttt ttggtgaact ttaaacattt caataccaca atttcattta aactttatac

154141 aatatttact tgaattttct ttctcgactt tggaaggttt taatttgtac cctactaatc

154201 ggtgtcaagc ggaacacaac aatgtctggc gggttaccaa tcgatttgtt ccgtcgctga

154261 ttgtcaacta acgagccatt actacaagtg agtgatacga acatgagaca catttttata

154321 ttgacaccga aacaacaccg ggccacatac caatgacaga ttatatacca ctgctgaaca

154381 tataacaatg atttatctat actaatatta taaagaggaa aactttgtat ttttgtatgt

154441 attgaaataa ctcggtaact actttaacag tggtagagcc cgctttaaca tcttcgcacg

154501 aaatgtccgg ctcgcctggt gaaataccac gaccacacag aaaacgggcg tcaaatggaa

154561 gcaattccgt gtttcgtctg aggagtgtgg tgccggagga ctaactttta atccgctttc

154621 ccttcctatc cttttcctat aaggaaatga taggaagagg aagtggattt tccggtggag

154681 gggacgcata gggagaggaa tgatcctctt gctgtgcgtc tcctcctccg aagaataagg

154741 gtaggcaacg catctgcaaa tgtagatgtc tatgggcagc ggtcgcttcg ctatttcagc

154801 gaattaaagt ggccgcttgc tcgtttgcca ctttttaata taaaaaaaag gtcgatttca

154861 aaaattcttt caccattaga aagctgcatt atccccgagt aacataggct gtatttttta

154921 attccgcgcg gacgaagtcg cgagcaacag ctagttttag atacgtttaa agaattcaga

154981 tatgacgcct aagaagtatg gatttgctgt cgcccgtaat ttcgtttcat cgagattcgt

155041 aaagccgttt cggatatacc ttcaaacaaa catagtaaac gcatttataa tatttatttg

155101 tgtaaaaaag ttaaattttt ctattttttc atacatttta catgcaatac tcatacatat

155161 ttatgaatat ttcgacagta gaaactcgta ccgctttatt ccgaaacaag aattagcggt

155221 aatactgttg tactttctac gtttggtact ggtacattca taaaatgtat tccaaaaaat

155281 acagcacatc cttgttcctg gtacttctta tgatccataa ttcggtccaa tgagacccct

155341 gctctatctt atttaattca gagtgtcttt cactatcgta taatcaacaa cttattggtc

155401 ctcgtttatc gcagtacttt atacattttt tcggaataag ggttaagaga ctaatgctat

155461 atttaatatt ttcatatcga ttttagctca cgtttaacct tcgtatggcg atgtcactac

155521 acgtctctag agattgtcat gttctataca tattgaacca cgagcgtgta attttatttt

155581 ataactagcc gttgtccgcg attttgtctg cgttgaatta taaatatagt ctatgttact

155641 cggggataat gtagctttct aatggtgata gaatttttga aatcggtcga gtagttcttg

155701 agttatttca aacatactaa aatacaaatc ttttctcttt ataatattag caataagcat

155761 agataagctg ctgtccgcga ctttaggtga caaacgagca agcgtccacc taaattcgct

155821 gaaatagcga aacgaccgct gcccatagac acctgcaatt gcagatgcgt tgccaaccct

155881 taatttaagg aggaggcgca cagaaaaagg ataattcctc tttctatgcg tcccctctcc

155941 cgccaaatcc actacccttt ccttatctta taagaaaaag ataggaaggg aatgtggact

156001 taaataagaa gtcgggcccc acactcatga gatgaaacga ggaatttctt ccgtttcacg

156061 cctgttttct gtgtggtcgt tggacttcac cgggcgagct ggtctattcg tgcaaagacg

156121 ttaaaacggg ctttaccact gtttttcttc attggtcccg cctaattaag attatattaa

156181 aaaaaaactc taaattcata gctaaaactt tcaagattca gccatatcaa tgtaagtctt

156241 atattattat ttcaataact atcagttatt gataaacatc cgccggaaac acgacccagt

156301 gggtaagagt aagagcaaat atttttcgcg acaaataacc acctttctga taataatatt

156361 gatttgctaa aaataactta acacgtgaca ggaaagtcta ttaaatactg attactcgtg

156421 acttcgttcg cgcggaatta aaaaatatag ccattaatac tcgcgaataa tatagctttt

156481 taacggtgaa aagtagcatt gtatatgaaa atcaagcgtt taaaaataaa tatagtccac

156541 taaatacact caagaaactt tagccgcgcg gacatttaaa gtgtctggta agccgtttgc

156601 cccatccaaa gtgtccgact gcttgcaggg agtattgtca tagtcccctc gagagtcata

156661 tgtgaaatca gctatgtgaa tgtaagtaaa cttcagtaat atttactgca actttagcgc

156721 gtgattgcgt gaaacttccc aactggacag gataactaat gcccactcgc ttatagtaca

156781 tacgagtcct atcgttcgga taaatgctac ttgctactcg actaaaatac tatcctggtg

156841 cgaacaaggc ttaacggaag accagaaaat gacactcgct tatccaggtt cgactgtaga

156901 tagtatagcg tcggtggccc aggggtttag ctcttaacaa caactgtagg tcctgagttc

156961 gaatcccgcc gtctaccaat gtgtttttcc attttcatat gtacatattt acccgacgct

157021 cttagggtaa agggtaaatc atcgtgatgt ctgcacatat cggcgaagaa attcaaagat

157081 atgtgttaag tcacgcaaaa tacgcgctag tcgtggagtg aaggaaaatg gagccaatca

157141 tgggtcgaac tatttttttt tactatagat aaaagataat tactaggtca gtagcacatc

157201 attatcgact acgtacggta aaactaggcg acaaacaata ttttcgtaac accttccaaa

157261 ttgataacta tcaagatcca aagtataacg cgaccacttc acttacatcg ccagatgata

157321 agaattgttt acttgatacc aatgcatgta taaagtacaa acaaatttaa tccgtatgtc

157381 gctggttcaa atcctactcg aaggaccatg caggaaatta taatgggact gttaaaagtt

157441 gcgaaaactg gttaaaagga taaataagtg agaaaaacat gttgatcgta ccgtacattt

157501 aattagtaaa gagcgaacaa tgacagtgtt gccagaatgt aaaaaaatat aaaatgaaat

157561 attgccgttt cccaacaagc gctcatcacg agtgtccaaa aatatcaagt ctgcaccaga

157621 ttattaccac ttattaccac attatcataa cttgatcctc aaaccgtaaa cttcaaaagt

157681 tccaactctc aacttttacc acgaaacaaa caaaatcact aacaatagta atcccattta

157741 ggaatagaac tccgactcgc attgtattac actaaggctt cattagtcag agtccgctgt

157801 aaatacagtg attttgtttc ggcgtaaggc cgattttagc gtgttaagcc ttgtacgcac

157861 taggatagta ttttagtcaa gtagcaagta gtaagtatcc gaacaaagga tttactaatc

157921 tattctacta aaatgttgct gttcggacac gttaaactag tctgaacagt agtaaatcct

157981 ttattcggat aaatgctact cgactaaaag actatcctag tgtgtacaag gctttagagt

158041 aaggcgaacg gccccacaat ccacacaaac ctcaagcgat acattttaat cgaacgtgtt

158101 ataaccacgt tctggttgca atttccacgt atacctatat ttgcttttgt gattttaaag

158161 tgtaaaattg cgcaacaaac cacgcgcaca gtttattgac ctagattact ttaccgcttc

158221 ctatatttag cgtggccttt cacaaacagt acataaatag tagtcaccaa ataagaccta

158281 acactaacgt gaccagacgt cccgttttta acgggatcgt ccgttttaca gtcatatgtc

158341 ccggtgtccc cgaaataatc tctgggacgc tatattctcc cgttttcgga aacggcgcga

158401 aaactgtgat agttaagccg gtcgagcctg gcgtgacgat ccggccggtg caccggggct

158461 gaacgcggac ggtgtgcgag accgagactt tgttgactag ttttttcttt cgccctgttt

158521 acgacatttg ttacgagatg ggctaaacac gtgattggtc gatacgttgt aatcacggct

158581 tgcacaaacc cagggtgggt tagattagat tccctagatt ctgaaaatta ttgttatccc

158641 gctttgagta aaataaaaaa tggtcacctt acataccaaa ggacctctct ttgactgtta

158701 ttgattactc actaaagctt tgttcggact aggacagtat tttagtgaag tagcaagtat

158761 tttagtaaaa aacgtgtgcg aacagcaaaa ttttagtcga gtaggattgt agcagttgtt

158821 cgcgccagat tgtattttag tagagtagca cgtaaaacgt gtgcgaacag caacatttta

158881 gtcgagtagg ctataaaaac actttagtcg agccaatcca ttttactcta tttttacggc

158941 aagtagcggt ccgaacagct tgttttggcg agttcattag tttgccgctt tgtcgtaaac

159001 atgacgtgtc ggtggagcga ggaaactact ttaaaatatg tctccaaata tgtgggacat

159061 taatgcttgt ggaatatcaa gcaaataaat tagtctgcat accagaatgt gcaatagaat

159121 aattattaaa catgttgtta aaattggtaa ctcaattttt taaatatttt gtgaacaggg

159181 tttgaataaa taaaaatatt tgattatttt tattaagaca tcaaaattat ttttttaggt

159241 aaaacattta aacaccacat taattataat ttataatgaa ttcgtatttt gcagtgtttt

159301 cgtttcggtt ttggtaatgt attttctcaa acaaattaat tgggtacaca aacaatatcg

159361 ttccagcgcc atctttgggt agaccgagtg agctcgatcg ccgcgcggga attacaattt

159421 aatatcaaat attacccatt ttgttttaat ttttaaattg tttgaaattt taaaagtttg

159481 aaatttgaga ctttcaggga aacacatagg catttatgtg aacacatagg aggctactca

159541 gtaagatttc tataaaccac gagcgagagc tagttctaca tgttcattct ccaaaacatc

159601 acgcacttgc gtacagtgca gcagtactgc gacgactatg cgaatgagtt ggttttggag

159661 tggggatcgg agtagggatg gtgagtcgtt atcgcttttc gcgtcaacga aacgaaacgt

159721 ttaatttgct tttgccaact tgttgaaaaa ctagttattt ttgttaacca gttaagcata

159781 tattcaacaa agaaagatca aagaccagcg gcgaaaaaaa aaccgaccag tgacttcctg

159841 tatagatata cgttgaattt aattattcga aggaaaaaca gtcaacggtt gatttaacta

159901 tgtcgttgta caacgcccat ccctaatttg gttacttgct acttgctact ctactaaaat

159961 actaagatag tgcgaactag gcttaaggct atcccttagt gtagcttaat ttctcaagta

160021 tcaaatcaga gtacgttaga aagtacagac acgaggcttg actgttttaa tagacttcca

160081 aaaaaagaga aggttctcaa ttcctctgaa ttttttaaat agaaattttc cgtttcgtat

160141 atacgaattt ctgaaatcgt aattccaagt ttaaaaaaaa aaaacatatt tttaattaaa

160201 atgtaacaaa tcgatatgtc aaacaagtag gtaaacgtgg atacaatctt ctaatttcca

160261 ggcttgaaaa aacattttaa gcaaatataa gtacaaataa cataaaaagt taatactcac

160321 gtaattcagc taacggataa aacttcaagg ttgaagcgat aaatacatta ctaatgcaac

160381 gaaggccctt gaatgttctc cgttagtcgt tacacggcgt tataatacat cggcctccgt

160441 ttgcctctaa cgaaagacaa ggacaaaaaa caaaaccaat ttaagccgac caaagatcaa

160501 ggtaaaatga accttaagac atacacatag agcatacgtt atcaacacag tcgtgatata

160561 tgtatttgta attcatcaca aagataaaac aaacgctatg ccaagcaatt atgaacttta

160621 tttctatatc aacaaagttc acatcgatag tcttgcagtt acaaatacac gagttgcaac

160681 tgtatatggg ttaagcgaaa ataaacttta ttacattaca ataaaaggtt cgttcgctgc

160741 aagttaaacg tataggaaac ggaatggaaa cgtgcctaca tttataaccg cgtgactcag

160801 ctctcgtata gcgagataac gaaataatga tgaattcgtt actgcgggga atatactgaa

160861 ttccaaagag atatctatat atataaaact ctaacgtgac tgactgacat agatatctat

160921 caacgcacag cctaaaccac tggacggatg tggctaaaat ttggcatgca ggtgggtgtt

160981 atgacgtagg cgtgagtcta tatatataaa actctaacgt gactgactga catagatatc

161041 tatcaacgca cagcctaaac cacaggacgg atgtggctaa aatttggcat gcaggtgggt

161101 gttatgacgt aggcgtgagt aaaacaaaga ttttttcgaa attcaacccc taagggtccg

161161 aaatgggggt tcaaagtatg tatggaaaaa agtgagtcca ttgtcagaat agcatgaaaa

161221 ttggaatgaa tggtctttag catgaataaa taagtttgtc attcgagaat tttacaattt

161281 tttattttgg ggggcaaaaa gggggtcaaa gtacgaattt aaaaaaaaat tctcgcagcg

161341 taggtacgag tcctagaaac ttgaaatttt gcatagcggt ttctgtcgaa attcaacccc

161401 taagggtccg aaatgggggt tcaaagtttg tatggagcaa cgtgaaccct ttgtcagaat

161461 tccgtaaaaa ttggaatgag agctcttcag cacgaataaa tacttttgcc atttaagaaa

161521 tttccatttt tttacatttg ggggcaaaaa gggggacaat cttcaaattt ggtagggagc

161581 aacgtgattc ctttgtcaga aatccatgaa aattggaatg agagctcttc agaatgaata

161641 agtaactttg gcatttaaga ttattttaaa tttttacatt gggggcccaa aagggggcaa

161701 acttcaaagt tcgtagggag cgaagagatt cctttgctag aattgtatga aaattggaat

161761 gagagctctt cagcacgaat aaatactttt gccattcaag atttatttta attttacatt

161821 gggggcaaaa aagggagtca aagttctaat ttaaaattac tttttacagc gtagatacga

161881 ttcctacgga cttgaaattt tatatagggc tttctgttaa catgttgaag tgcactgata

161941 aatgaattta cgaaattcaa cccctaaggg accgaattgt aggtccaaag tttgtatgga

162001 gcaacgcgaa aatgaaaaac tttgcaattt tataaatttc ttcttttggg gataaaatgg

162061 ggtcaaaatg cgaatcataa aataattttc ccggtgtaga tacgagtccc agagtatgga

162121 catgttgcat atgggtttgt attgcttact tcttcaaatt tattttataa aggttttacg

162181 cggacgaagt cgcgggcgcc cactagttgt atataaaggg agcgctccat agtagtttga

162241 cagcaatatg cgtcccgcgt aagaaattat taaataatca aatctttccc agtataatcg

162301 aaattttaat tagtgtttta aaaatgtctc gattaaatat aatattaaac tttagtaaat

162361 actataaaat aaaattaaaa tatgagaaaa gaggttacat tcggtcgact ttttatgtcg

162421 gcaaatataa tttaaagaac aacactaggg gaaatttccc tacgagagac ctccggaggt

162481 caaggaaaat gcctcaattt taggcttcga atgctctatc tatagtaaaa tatatctttg

162541 ctgaattcac tatcgaaaac ggaaaacgaa tggttttttt taattagaac tcttggtaaa

162601 taaaaataat aagaggaaaa tccaagaaag ccacgttgtt ctaagtgtaa taattatttt

162661 gataagcaat acacaagtcg gcctacaagc ttaccataag catactttgc tgtctacatt

162721 ttaaaatttg tataaaggct cgaccctaaa ttcatttgct ggggttccca acaagaggct

162781 gttgtttgcg gacccctgtt ttaccaaaca gttcgggtcg cattcgcaga cgaaataacc

162841 acaggcggcc ttcctttggt acgttgaacc gaaccttggt tcgtggtacg ttttttttct

162901 catctcttat gaatctgtcg accaaaataa atatattcaa atttaaactt taatataata

162961 gaaataagaa ctatgatttg cagccagacg gtattgtatt tgattttttc tttacaacga

163021 attatataac tacagtagca tctgaccacg gtcttacctg aagtagggtt gccacctttt

163081 cggatttcac atgagttttc ggaccagaag tttgctttcg ataatagtgg atggtgtgaa

163141 acaatagtta aaggtaatta gtctcagatc gacattttgt tcaccattag tctttccctt

163201 tattccgaaa aatttataaa atactacgat taatgaggac caataagacg tcgattatac

163261 gatactggag gacactcgga actaaataag atagaccagg ggtctcattg gactaaatta

163321 tgtatcataa gaagtaccag gaacaaggat gttctgtatt ttggaatata ttttatgaat

163381 gtaccagtac caaatttaga aagtacaaca gtaataataa cgctgattct cgttttggaa

163441 tagcagtttg agtctacttc aatgaaagcc agctaatgtt aagttatatg tctaaagact

163501 agattagtat agaaatctat tccctctata gacagaaaac ataatgtata taaaacgcgt

163561 gttgaagata tatggatatg tatgcaaata cgcaggaaaa ctttaaaatc gtcaatattt

163621 cgtaaaagtg tgaaaaatca attacatcgg actgacgtca acacgtcggc tacgcgtaga

163681 aaacgtaggg atggggcttt gtaggttttg gcgtcaacga aacgaaacgg ttaattggct

163741 tttgtcaact agttgaaaaa ctagttattt gtgtcaacta gttaaccatt cattcaataa

163801 agaaagaccc aaagactaga gtcgataaaa aaatgaactg agattctcta tgtagatttc

163861 cgttgcattt aactagttga ttgaaaatca atcaacggtt gaaccgttgc cgagcgatta

163921 aaggttgatt cgactagttc gttgaatcca cgcccatcct caataacgtg gttaaaatat

163981 atataggtac gggctccaaa aactattact tggggtagac acatatatag agactccaag

164041 gaaccgcaat attagtctgg cttatagaag tttctctcta atctagttca cgctcgagtt

164101 tctcacttat gggaattgtc cctcgggacc atacagtgac tctcgcttat tgaggtttct

164161 cagtaatcga cttataaaag tgaatatcaa atctatgaag ttttttttaa aactttggtt

164221 ttactattgt gtggtgtaac cataagtatt tcgtattaaa ttcattgcga catttaaatt

164281 aaaataatta tcacaaaatt tccttggcct aaaataatta atgagtatta aaaaacactg

164341 aacgataaac ccacaataat tgcatctcat ttattctggg ctttttcaaa atacttgtac

164401 gataacattt ttaactctta ttcaaagaaa aacaatgatt taaacacata aatattaaaa

164461 catttaagta atattaaaac ttacaatggc aatcagcggt aacacttgta aataaaaatt

164521 atacaaccgt ttaaatacaa gattgttata attataaaat tgttaagcgc gtctagaaaa

164581 taaaccaatg gttcggtata attgtaagcg tctactccag acaaactcta aacaaaactg

164641 gtacatattg tcatccctct tatacccttt gaacataaag agacaacaag tgtatacagt

164701 agtacctgga taaacgagag tccaagggac cgcgatattt gtctcgcttc tagaggtttc

164761 tctctaattc gtgtatctcg cttatggagg tagctcgtcg ggaccataga atgactctcg

164821 cttatagagg tttctcgctt aaccacgtat gacaaaaata acaaaatttg accatattga

164881 acatgcagga agctaaaatt tcttaaacac attttgaaaa taaattataa ccactagtct

164941 ctacaacaat ggctagcctt tctattgggg tctattaaga agcatatctt ttagattcct

165001 acaaattcgt tagttaccag taaaccagtc aatccaaagc caacatttcg acttttcata

165061 tttcccgacg gatccctaat caacataaca caaatagtcg taacaattta cttgaatacc

165121 attcatatat acaaaggcaa aataattcac attaattaac aatacgggga acaaatcaag

165181 ctagtgacgt catcagacac gactgcaatc ttcaagagga caagcagaga atcacatgca

165241 caaaaacaaa atcaaaattt aaaaaaaaaa tcatcaatac ttacactaaa ccagagatac

165301 tccaccacag aacatacaaa aaacagacac gacagaaaca caaaaaaaaa aacaatctaa

165361 taaaacactg gcaggtaacc acgcggtttg cgtttctttc caacgcacga cttgccaagc

165421 acagcttgcc gtgtgctagg taaacataac gactagtcga ctgccgataa ctctatcgca

165481 acgataacga gcccgttagc cgagtaacgt aacgcggacc ggcgttacga catagattat

165541 gcatcgcaga cgtggtaaac cgttttacaa atacctgcat attctaccta aacgctactg

165601 ccataagtta atagtactga gggaagtatt tacctaactt aacatagaga aaaccttgtc

165661 actgttcctt tgtgatttgg cagtaacatt attaagaaat ctactatctg cttttgcaaa

165721 ttttgcggtt attattaaat catgtataaa acttgtccac tcattttaaa aactatctta

165781 atccgtagac caatcgttta tggcaatttt attaccgttt ataatacgaa gtaaaaaccg

165841 taaaataaaa tctaggacaa cctggcaata ttgtataacg attacaaata aatattggta

165901 atacatattt ctattcaaat tgccgtaagt gtaacaattg gctgacatag ttttctacct

165961 aattgccatt ctgcgggcat ccgtgatcaa tgacgtaacg tagttaaggt aaggaaaaag

166021 cgatcgagtc taaacggcgg cattggaggc cgtgtaaata caatttcatg gttcgtacta

166081 ttcgtgccgt gcagtgcgac taacttatct ggcccgataa gtagttctgt tatcgcagtt

166141 ctttcagaga atatgtaaaa cctacataga tataaaaatt acagcagcaa taaacaagaa

166201 tttgtagcgc ctagcccagt gagagaggca ctgttatcgg tcatttcagt caaaaaatat

166261 gacatagacc agggcttccc aaactgtgag ttgctccgtt ctggggcgtc gtgacattgt

166321 cccaggggcg tcgcataaat aaaaattata aatgtcctaa tagtgttctt tttataacct

166381 actagcagtt gcccgcgact tcgtccgcgt ggtgtttaat gttaaccatg aatcttatct

166441 tcggatagct cattgagtct aaggcaaagg tagctgctag gaagcccgga gtccttacca

166501 aggacttcac accgggacag cgttttattc tgtacatagc acatatctgg ccgtgtgtgg

166561 aatgctgctg tcacatctgg gcaggtgcat ccaaatacca actcgcaccc ctggattcag

166621 tggagaggcg ggccaaaaag tttatcggtg accaaaaact aaatttagtc aatattaagc

166681 actggcgtaa agtggcgtaa agtggccagt ctctcggtgt tctaccggct acatcttgga

166741 gggtgtgcgc aggaattaca tgaaataatt caccatcacc attctaccat cggacgttca

166801 gccgtacgac aggaatctga acactgaact gcatcatcta agtagcgtag gtatcaacct

166861 acattgtgaa aacgcaacat tatactttga tcgccaaact gttagtattt aaaacggaac

166921 actgtcgaaa tttttaacca tttttttcta tttgatgtaa ggtccaaaga ttttgttata

166981 ttttacggaa ttaaaaaatc ttgtctagaa caaaagttac agacaatagc ttgtaacagt

167041 aataatggat gcttcgaaat gaaccatttt caaaccccca tttcggattg gaaaaaatgt

167101 tatacttgct tgatatgtac tcaattaaat aattaaatat cagaactctt ttgcattttc

167161 ttccacgccc ggctcgtgag atgatttttc ggttaccttt ataacttgtg ttcgtgatat

167221 cttctaaact attcgtcaga tttaaatacg gttaaggacg attttaatct acattaaatt

167281 ctcctgataa caaacacatt tatttggata aggattaata tttaaaccaa aaaaaaaacg

167341 cggcaaatta agtgcatatt ttaattgatt tttataaaac aaaaagtgat tattgtgaga

167401 aaaccgtaaa agataaatat atgccatcgc tgactttttt gtagaacttt tttagatcta

167461 caaatgtctc atacatcatt tttatataac tcgagcggtt tagccagcgt aagccaatga

167521 aaaggaaatt taatgaaatt ttgcgatagg gtccagaaat gcgctgttta ttcctatttc

167581 ctaaactttt ctaaaaataa attatagcct atgttagaca caaagaacgt agctttctaa

167641 tagtgaaaga attttcgaaa tccgttcagt agattttgag ttatttcaat acaaacaaaa

167701 atacaaaaat acaaaaatcc aaaaatacaa agtttctctc tttataatat tagtatagaa

167761 aaagtgatta ttgtgagaaa accgtaaaag ataaacatat gccatcgctg acttttttgt

167821 agaacttttt tagatctaca aatgtctcac acatcatttt tatataactc gagcggttta

167881 gccagcgtaa gccaatgaaa aggaaattta atgaaatttt gcgatagggt ccagaaatgc

167941 gctgtttatt cctatttcct aaacttttct aaaaataaat tatagcctat gttagacaca

168001 aagaacgtag ctttctaata gtgaaagaat tttcgaaatc cgttcagtag attttgagtt

168061 atttcaatac aaacaaaaat acaaaaatac aaaaatccaa aaatccaaaa atccaaaaat

168121 acaaaaatac aaagtttctc tctttataat attagtatag attactaggc tgttactaaa

168181 attggaaagt atttttgggg gttggatcga ggggcttcgt ataaaaatga caggactaaa

168241 gggcgtcaca ctacaaataa atttgggaag ccgtgacata aacattaaag gagggcgctg

168301 agctatcata attaagtttc acttatgtcc gaccaagtgt ggcacttttt taacattgac

168361 aggaagacgt gtatgaagca ttgaatttat gtatagtttg ttatacgctc accgggtcag

168421 actaacttgt tgaggtatag ttaactaagc aaatacctct tagcattcac acggacgttc

168481 tttcaacgtc ggctacggcg tttttctaac gtcctcgctc atacgatcgg tgtaaaatga

168541 ctcttgtttc ttttgtccag ccctcaaaat atgatgcgcg ataaaaattt aaagctacgc

168601 gtgacgtcgg cgtgacgcga ccgcgaacat atcccgataa tccactcacc ctgatgaagt

168661 acccccgacg ggtctgaaac tagtctgtac ctacacggaa ataaacgaga gttacctgtg

168721 tttaatgtct cacaaaagtt ttaatagatt taaaacgccg tcgtgtgaac atggaactta

168781 aattgcgtta aaaaatcgct ggtgtgaatg cgggcttata ttacatgtta gttttgtctt

168841 gtttgtagct aatattgcag tacaataggg caattgttag ctgtgtgcat atttacacat

168901 tttgctgaca acctaacaat gagtatgtac cctcacatat attatagtta ttccaataac

168961 aaggtttttc agataacgta ttctaattat aatattaatt caatataagc cttcaaagta

169021 caattaacac taaaatattt tttataattg attaattaat aaaaaaaact atctaaaccc

169081 aactctctgg gttacgggcc gcgtgttttt aaaatatttg agttgttttt tatatctttg

169141 tagctattgt ttttcgactt cgtccccgta gaattaaaaa tatagcctat gttactcggg

169201 gataatgtag cttcctaacg gtgaaagaat ttttgaaatc ggcccagtaa tttttgagtt

169261 atttcagtac atacaagaat acaattcttt tttcttttta atattagcat ggagaagcta

169321 tcattagatt aaaaaacaaa gcagcctaag tgatcttccg gattatgttc tacgtctatg

169381 caaaatttaa tcaagatccg tagaacaatt ttggatatgt atgtagttcg acccatgatg

169441 gactccattt tccgtaactc cacgactagc gcgtattttg cgtcacttcc agatatcttt

169501 gaatttcgtc accgatatgt gcaggcatca cgatgttttt actttaacgt aagagggtcg

169561 gataaatatg tacatatggt acattggtag atgccgggat tcgaagacgg gacctgcagt

169621 ttacaagaca agagctaaac ccctaagcct tggataaact aactggtaat ttacgatcaa

169681 ctttagtcaa tttatattat ttattttagc cttgtttatt taataacttt ctaaaatatc

169741 aatagatcct attagtaata aaattacaaa acgcctattt tttgtatatt tgcaattacg

169801 gaacatctca aaaactagtt acgggaaaag tttagaaatt aattgtacca taactaggta

169861 tattgtatta tttaaactct aaatacctac ttaatccact tttgtattga ttttatttcc

169921 acacaaaagt gtggaattta tttttcaatt cactctatta tttttaaggc gccttataga

169981 atagcagagt gaattttcta atacaaaaca cggggaaacc gattgaaaaa tcatagggtg

170041 tcccaatgag tttgaacagt tttaacaatt gactttaggg caaattctag gattgagttt

170101 aattacaaaa tattcgaatt tgaatctagt atgaaaaagt atcaaactat agtctcattc

170161 aaactttatg ttggctcaca atttcacact gtcactgaac aaaacacaat tttatctatc

170221 tgtagggttt aaagggatac ggaggaagga caaacaattg cgaacattct ctgttcacgg

170281 tggtgtcact taagccggct acaaactgtc cagttaaaac tgatgtacac tcatcaattt

170341 gaactgcaca gacgaactta acagttctca gttttgggta cacacgtcag ttttaattgc

170401 gctgtcaatt gtacagttaa attaagcgtc tgtagccggc tacaattaca aagtactgct

170461 atatgctcag ctaagcacta gctgtgacaa agtttgcatc ttgtaataca cgcacattaa

170521 gtaatactac catgaagaga aaaattaggt gacttgtcgg aacaaaggag aaaaaattaa

170581 agtaaaaaat atatctattg tatactttcc taataaacaa agcgactgtt ttttgttcgt

170641 attttaatct taacgcttcg atagatcgcc tacaaacctg cagattccgg ataacgattc

170701 agaatatttc ataaaatctt aaactagaat atgattcaaa atgaaaaaaa ttaagccttg

170761 ttcgcaccag gataatattc tagccgagta gtaagtagca tttatccgtt cccatgattt

170821 actactgttc ggactcgttt acgcccaaac ctacgcacta ggatagtaat gtagtaacta

170881 aacgtatcct aacagtaaca ttttagtaga ttagtaaatc ctttattcgg attcttacta

170941 tttggtactc gactaaaata ctatcctggt gcgtacaagg attcagtgac cggagaaagt

171001 tattacataa ataattttgt tttgtctcta ttcttttaaa tataatgaaa gagatgacca

171061 atatgttttt aaacagcagt ggaaactcat agtagttcat gatgctaatg ttttagtata

171121 cggtaagttc acacgtaatt ccttgatttg ttcattattt aatcatacaa atgacttcaa

171181 cgattatgga tagcccgttt atattatcaa aagtaaacaa aatatattac aagacgatgc

171241 gtaaccacaa ggtctcatcc tgtagtattg atgggctgac agctcgagtt tttatccgaa

171301 acggataaaa acccgtatga ttagacaaac aaagtatcga aacgttttac tccaatttcc

171361 tcagtattgc taggatcaat tcttttttcc tttatgccat cagatcctgg tagacctata

171421 catacgcgga agtacctaat agttttgcat tcaaaagttg ttttcatcat taaattaatt

171481 accaaagcat gttagatata attttatttt tgttattctt ttattccaat ctcgtttatt

171541 aaaaaaaaca tgtttatgtt aaattacatt tatgaaatga cagatagttt ttatccaagt

171601 ttttggtaaa aatcacgccg aaaagatgga aattgagagt ttttatccgg atgaaatccc

171661 aacactattc cgtagtgatt ttttttattt tattatctgg ggtagttatt aaaactatac

171721 tcgtaaactt taaaagttta ttactcgcat attaaaactt tttgtttgaa aaggatctac

171781 tgtttgtttt tggtggatgt ttttggatag ccagggaaat ccaaagtatc tttcgattag

171841 ctgcaacatg tatgaaactg acaatgacat ttgataagtc atgtgcttgt tacgtgtgag

171901 atatcgaacg tcattgtcag atctacatgt tttgatgttc gctatcgaat cgcttcgttt

171961 cgaaaagttt caatttcttt gttaggggtt tctatattat agttagtaat acttacaaat

172021 ttaaacgagt gcaagtccaa ttacacaata acactaataa cactgtaaac aacctttgtg

172081 aatgatgaac caaaactgat tcagatcaaa tcgatgtacg ccattttata ttgaaaagcg

172141 cgggaaaaac gatccgtatg cggaagaagg aaggatgcgt ttttacaaag agatcgaaat

172201 taaacggtcc gttagtcaaa ttgttagcta cctttgttac aaattgtata gttttcatag

172261 tttacgtgaa tgaagggaaa aaaactttca tccgagttgt tttttttata ttggctgatt

172321 atctttcaat ttcctcgtgt cttgtccact ttaccgctgt ttttacgtcc cttcttgctt

172381 gttagtggac aacttaagcc ttgtacacat taggatagta ttttagtcga gtagcaagta

172441 gcaagtatcc gaacaaagga tttactaatc tattctacta aaatgttgct gttcggacac

172501 gtttagttac taaattacta ccctagtgag caggtttagg cttaaacgag tccgaacagt

172561 tgtaaattct ttgttcggat caatgctact tggtactcga ctaaaatact atccttgtgc

172621 gaacaggact ttaatcagtc tggggtgagt gactgtggtt cgcgcttttt taggacaaag

172681 gtttataaga aattataatt tagtgcttgt agaaaattct ataaaaaaat attgtcatct

172741 ccctgagcat ctctacatag tataaaacaa agtcgctttc tgtccctata tgtatgcttg

172801 aatctttaaa actacgcaac ggattttgat gaggtttttt tttaaaagtg attgaagatg

172861 aagatttaaa tgtataacta gcgacccgcc ccgtcttcgc acgggtgcaa ttattatttt

172921 ttatatacaa cgttcacagt ttttctgtag tgtatttagt atcagcatta gacgcgtgcg

172981 aagccggggc gggtcactaa ttctttataa aacatcagag acaatatcta tctactcgta

173041 tacaggtatc tatagacatg caatgataaa tgagagcatg ccctagcgtt acctggtctg

173101 tctacatttt ctaaactgtg tgttgcagtt aaatcttagc tataacatga accatccagc

173161 tccataaata atgtgacaga tctgaaatct agatattaag gtactcctaa aaacatcaat

173221 aacattagta cgagtaatat tcaatttaaa acgcctattc cgtacaaata caccgtaaag

173281 tgcacatcat gaaatataag tacatgcaga gactccatat ttcacaatgt gcatatttcc

173341 atgtgcagaa tgtgtttttt ttttatatta taaagtggca aacgagcaag cggccaccgg

173401 aaatcgctga attagcgaag cgaccgctgc ccatagacat ctgcaatcgc agatgcgttg

173461 cctaccctta atctacgcac gaagagacgc acagaaattt tatgcgtccc ctcccctgcc

173521 aaatccactt ccccttccca tcatttcctt ataagaaaaa gataggaagg gaaagtggac

173581 taaaaatagg cctccggcac cacactcatc agatgaaaaa cacggaattt ctgtcgtttc

173641 acgcctttct tctgtgtggt cgtggtattt caccgggcga gccggacaat tcgtgcgaag

173701 atcttaaagc gggctctacc actgtcaaga gattttatta ttgtgtttgt tactgtgatt

173761 ttattaatgt gaagacttta gttattcgac attattgatc actttaggag tttctggata

173821 cctgggggga ttacccaaag tacattcgct aggaagtgac tctatagcga acatcaaaaa

173881 tgtatgggac tgacattgac gttcgataag tcatgtgctt gtcatgtgtg aggtagaccc

173941 cctgatctgt cactttattt ttccagctat tactatagtt gtactatttt ttttccttta

174001 actattttcc tagcaattaa gcgctaactg cattagagaa tgtgtctatt tttgacacat

174061 gatagaataa aacgcgattt gcggtgcgat gtgaacgcta agttaccgag gtcagacacg

174121 atagtattgc ctgccttcta atcctcaacc aactcaaatt cactgcggct aagagccgca

174181 ttctgttaaa acttattcaa ttaaaattat acctagttat attgtcataa caacaaccta

174241 acccagaggg gtagacagga acaacggatc tctattcggc tcgatcctaa taaacctttc

174301 tcgcttcttc tacattcata catctacgca tatacgacgg ttcgcgttat cttaataccg

174361 gccttcttga gtaattcctc aatttgaccg gaataagtcc gcctcgaccg accccgtcta

174421 acactgccac tcactttagc attataaatc gtctgcgtta acctgcattc attcatcctc

174481 tccacatgtc caaatcatct aagcatacct ttttcaactc tacatacatc ttgttagaaa

174541 ccacattctt ccctcacaac actattccta attctattat tcacacttac acctatcata

174601 cttctccaag atctcatttc cactgcatta accttactcg catccttttc ctgcccaacc

174661 caagactcac tatattgtca tttgtactat aaaataaatt tataattaaa aagttactct

174721 tggtatcacc aacatacgaa tacagtatat tccagtgctg gtttactatt ccaggcgcaa

174781 tcctcttttg ttttctatct ctgtactggg acaatgtgaa ccgggtttaa ttcttcatcg

174841 aatatgtatt aagttcagac gaaagtgtcg taaatgtaat ttacacactt tttaaatcta

174901 tatatataaa agagtagagg ggtttcttaa aatcagcgca cagccaaaac tactaggtct

174961 agagacttga aattttgtac agaggttact gttactacgt agatgagcac taagaaagga

175021 ttttgagaaa ttcgaccctt aagggtccga aatgggtgtc caaagtttgt atggagaaaa

175081 atgattcctt tgttagaatt ggatgaaaat tagaatgagt gctctttagc atgaacaaat

175141 aagtttctca ttcaagattt ttacaatttt ttactttggg gggcaaaaaa ggggggtcaa

175201 agtacgaatt taaaaaaaaa tctcgcagcg taggtacgaa tcctagagac ctgaaatttt

175261 tcgtaggagt ttttgttact acgtagtttt gcactgagaa aagattttcc aaaattaagc

175321 ccctaagggt ccgaaacggg ggtccaaagt ttgtatggag caacgtgatt cctttgtcag

175381 aattgcatga aaattggaat gagtgctatt cagcatgaat aaataacttt gccattcaag

175441 gattttataa atttcttctt tgggggggaa aaaaggggga tcatatttca aatctagaga

175501 tatttttcac accttacatc agaattgcat gaaaattaga atgagagctc tttagcatga

175561 agaaatcatt ttggcattca agaattttac aattttttac cttgggagga gaaaaagagg

175621 ggtcatggtt ggaatctaaa aatattttca cagcgtagat ttgaaatttt gcataggcgt

175681 ttctgttacg acgcaaaagt gctctgagaa aggattttac gaaattcaac ccctaagggt

175741 ccgaatagtt gttcctatgt tagaattgcg tgaaaattgg aataagagct cttcggcatt

175801 aataaataac tttgccattc aagaatttta ttttttttta ccttggggga aaaaagaggg

175861 gtcatagttc gaatctaaaa aattttttca cagtgtaggt atgagtctta gaaacttgat

175921 attttgttca tgggtttcta ttactacttc aaacttattt tataaaggtt aaaataagat

175981 gaaaagcttt gtctccaact tctaaacttc aagcggacga agtcgctggc gaccgctagt

176041 gtctgataaa actaattagt gattaaacaa gtcattgctc tttcttggca cgaactgaaa

176101 tagcctaatt ttaaaattat accatcatca tcatcatcat cagatcacta tacgtgccca

176161 acgaggggct cggagactac cctatcttaa ggtacggcct aaggccttgt accaggacag

176221 tattttagtc gagtactaag taactatacg tatccgaaca gcgacatttt attaaaataa

176281 attagtaaat cctttgttcg gagacttgct acttgctact cgactaaaat actatcctgc

176341 gaaggctttc gtcaaccaag ctgagccagt gattagggga gaaaggggca acaagatcaa

176401 aaagatcaca aaaatcgata attaaaaaat agctgacaat cgatttcctg taatctgtca

176461 gatacctcat atcacgtaat acgtgccgtt gtcacgtaac tccgacagcg ggcgccagtg

176521 gaataacaat cgcggcattg gccggctcgc cctgacccca aggtcgcggg ttcgaatcca

176581 agagacatac gatgactaac tttgtacatc gtttatcaat aatttcattg taattatttt

176641 tttgcttatc tatactagga ttttaggaga gatttttttt taattttttc aaataccact

176701 caaatagagc gttttacaag cccacgatta aactccaaat tatacggtga tctcgtttaa

176761 ttttgacacc acctagattt taagcgacgc ttaaatttga gtggcgcttg atctttcagc

176821 cgttaatcca tttttgtact aacactctta ccctcgggct tcattagaca agttcgcgta

176881 aaaaacgccg atttaagtcc gtaagaccgt tttttgcgtg acatcggtct aatgaagccc

176941 tagggttata cagtacaata attattgtcg attaaacaca aattataatc tctgcattgc

177001 atttaaggat ctattattgg gttcgacatt tatagattgt aaaaaatata cctttataaa

177061 aataaaatat aaaagattta taagtctcag gaagacagtc tgtgattatt tttaaatgtg

177121 ataaaattac aataaaaatc ttttgtatat tcgaaaaaaa actacagtta tataaaaata

177181 gctgtattga atttagtttc aattttcaat gttacactat tattataata ataaaaacag

177241 tctaattcct ttgcataata aagtttttct acataatatt ggtttccaaa tataaaaatc

177301 gatctttatt ctagctgttt aaatggataa cctttgatgt cattttcact gtttcactgc

177361 acttttacgt gttgatagta agaagggaag aaatggaaga aggaagggga gacgcgtccg

177421 cgttccggaa agaaaagaaa gatggcggat tagtgaggtg cactattgcg ccgaaatgtc

177481 actggtcgca actagtgatg tgccgcaaca ctagctaaat taaacgttag acgctttgta

177541 aaatgactaa gaactaatga aacatatatt caaaacttat ttcttcaatt taagtattgt

177601 aaacataatt aatacataaa cgtacgcagg aattaaatac aaacatagag attgaaatat

177661 atagagaggg ataagtgacc aattaacgtc tctgagtgcg ttcaaatcaa atctattttt

177721 tttaatacaa agtggcaaac gagcaagtag ccaccaggat tcgctgatgc gttgtctacc

177781 cttaatttac ggaggagacg cacagcaaga ggagaatacc tcttcatatg cttctcgtcc

177841 cccaacaagc cacttctcat tcccatcatt tccttatgta agagaaagtg gactaaaatt

177901 aggactccgg caccacactc atctcaaaca cgggaaagca gccatttgac gcctgtcttc

177961 tgtgtggtcg tggtatttca ccgggcgaga tatatccaac aagtagtata ataactttag

178021 tacatattta tatgggtgtt acccattcat cgacctatct ataaaatatg tgtattcgta

178081 tctatattta tagtcataaa attcttacgt attcattacg caagcgtgac gtgtaagcgg

178141 gacagatgtg gatatttttg tattaccact catgaagcaa cacgcacgct accaacgcgc

178201 tccagtttac aatttatatg tctaccagcg cccaggcttc ggacgagttt ttttttttat

178261 atgagaaggg ggcaaacgag cagcggatcg cccaagttag ttaaaaacat ataaaccttc

178321 cttatgaatc actctatcta ttaaaataaa ccgcatcaaa atccgttgcg ttgttttaaa

178381 gatttagggt gagttgcatc aactagctat aaccataacc gaggtcgcga ggctggtcaa

178441 agttacggtt acgcatattt tcgttgcatc attttgacta tggttaaatt gacgtcataa

178501 ttaacgctaa ctctaaccgc tcaattttgg gcggttactg taaaagtaat ggttagtttc

178561 tgtcagtata tgtcaactgt cacagttcta cacgacaaaa taagctattt aaatctacaa

178621 taatattata aagagagaga ctttgtattt ttgtatgtat tgaaataact caagaactac

178681 taggcatata tatagaattc tttcatcatt agaaccatca gacaataata atgggtctaa

178741 atatattaga gtacatacag tacagtgtat aataatattg ctggtttatt attgtttaca

178801 gaaacaatgc tataacacca gaacagcaag tactaatgac tctagggttc tatgcatctg

178861 gatctttcca acgttgtatt ggtgacgctg caggtgttca taaatcttgt gtatgccgaa

178921 caatacaccg agtgagcagg gcaatcgctg gacttcgatc gtagtggata tctatgcctc

178981 aaagtagaca atccatggaa caagaggcca gaaagtttta tgcaattagt gcatttccaa

179041 aaataatagg cgctattgac tgcacgcata tatgtataac atctccaggc ggaagtgatg

179101 ctgaagtagt cattgttgca atggctgtac ttcataatat ttgtaggaaa gctagaaacc

179161 ctatgccata ttccacatgg catgaagacg acttgattgc ctcagatgaa gagaagttat

179221 ttagttcatc tttatcagag aatgatgaaa acaacagaac tttcttaatt aataattatt

179281 ttcgtagatt agtacatagt taaaaataaa gacagagttt gaaattatta tttttgtaat

179341 tttgcacgta aaatatctat ttctaaattt atcttcaaat ttatgctttt cttcattgtg

179401 gcgcattttt tccaacggca ttgaattcct cctcaatgat atgccaaagt ctttcctttt

179461 gctttgtatt tacactatcc gtcttcttac acattaaaat atctttatat ttctctacaa

179521 ttgcctgtag gcgattaact tcatctttac taaagtttgc acttctgtct ctgcaatcaa

179581 ataaccatta tgcaagtagt ctgggtgtat aggaaagtac cacagatttc tagaacttag

179641 tttgtattga agagaaatgt ttcacgaggg tttgccatat ttttttaata aaatccaccg

179701 gcgaaacttt atgatttaaa ttattttatt gacaacgaat gacatattaa ttcaaagttt

179761 gtcaatttct tgacactcaa ataaaagtgt tcccgttgta aaaaaattac ccgtgtaaat

179821 ctctgggaga aaaatccctt ttatcacttg taaccctttt aactttaact ttaacattag

179881 atgtatcggt gcaacgcttt tcagaatttg ttaaaattac tgtgtatgtc accgttataa

179941 ttaactgtaa acatagcttt aaccggtgat atgatgcaac tcacccttaa gcatacataa

180001 ggataggata gggacacaga aagctacttt gttttatact atgtagtgag tacttaaaca

180061 tatttggata agcgaagaat taaaattaag aaagacaatt ctgtacaaaa tgatacccag

180121 cacggcagct tttatatatt taatctcgtt ggtaacttaa atttatatat tcttaaactt

180181 acttaaactt tatcctaaac tatacatatt tacatttcac taaatatctt ttttggttcc

180241 tacatcgttg ttcgcttctt gacgttacat ttttcactcg ttattctcct gttttttact

180301 tatatttttt atttataaaa ggcaatggcc ttatggccat tatgccacca aagagatgca

180361 atttagactg aacattccgc ccaccaagaa ctttgttaaa gttcttaact aaactgcttg

180421 tctccgccca ccatgagata aggaggagaa ctgctgacgt tggatacaaa ctcaaaacac

180481 acataaccta acttcttaag cagtggactg tataaaactt caaaattacc atctaacctt

180541 caatatcaaa acacacataa cttaacttct taaacagtgg actgtatcaa ccttaaatta

180601 ccatcttaca tcaatatcaa tcaacatcaa aaactcagtt acaatttgct aatagccaca

180661 ctcaatatca tttggcttca attgctaaat tcgtcaacat aaaacattac atacaatact

180721 taaacacatc ataacacatc acataaactt gagcagtacc tacacttggc acaagccaca

180781 caaaagccac agaggcgagt ttccttcctc atagctacgg ttttccgaca tttggctgca

180841 cgttgttaaa tattgtgcga cttgctgata cttaggcctc atagccttag tctttttctt

180901 aggcctcaaa gcctcctagt cttatagcct catagctgct aatgttcatc ttgtcgttgt

180961 tacatcacaa ttgcttaaac taacataaac gccaatactt tgcgacttcc ctacttctta

181021 catcacattg tggtctttat ggtactatat ttattatgcc caagcatcct tctaaatgcc

181081 actccgaaat atggctgtgg agtccgttgt tattgacatc acacatattt cggagcgtgt

181141 tcacttgatg ccttggacaa aggatccatc ccagtgcctg tacgttctta agaccgctgt

181201 acactctagc tccggccaat ccgcgttgtt gtttcggtgg caacgggcta gtggtaaaca

181261 gcttgtatgt acatgcggtc gcactggaat atacctagga tcaaaacatt tccataatac

181321 accctagaca ttaatttact gttgctatct tatttatttt tatctaacca ataaacagta

181381 aaaattctaa ccttaaaata catcaataac ataacataaa cataacgtaa atattcataa

181441 gtcaattctt aacacaactc aaataacata acataaccac ataaaataac cctaatacaa

181501 ataaaatcat ttttacctta aacacaaata acttaaaatc aaccttataa caatacacca

181561 aaaaaccaaa atcaaatcgt aaaaataacc aaacattaaa ttattttaaa ttcaaaaaat

181621 aattccactc aaaataaaat gcacatttct taaaatataa ttcacttttt aaccgacttc

181681 caacagaagg aggttatcaa gtcaatgagt tttttttttt tatgtatgtt cggacgtcga

181741 gcagcgagtt ctgaaccgat ttacgtgatt ctttttttgc tcgatgcaaa atagctgcca

181801 tttggtccca tatttacgtt ttaaaaaaac tatttagtag tttttgagtt atttttaata

181861 aacatgaaaa aaaaatggga cttttttgct ttctcggctt acgtagccta aactataggc

181921 cccatgaaaa tttgggtatt accagatcgt agctctttaa atgtgctaat ttaaagtccg

181981 cgatggcata tatgtatctt ctatggtttt ctcataaaaa acattttttc aaaagaaaat

182041 tttactcgac tttggacgct attattagaa ttccctatga atccttatcc aaataaatat

182101 gttcaacatc aagagaattt aatgcagatt atagttgacc tttgaaggtt atcattttga

182161 ccaatacttt agaagatatg aaggttttaa gccacgcgga acgtgaaaaa ttacaaaaac

182221 gttccaaatt gagcgagacc agtgttttct tgccgtatac agcctaaact ataggaccta

182281 caaaaaaact ataatgtgcg atcaatacat agtgaaattg gctacatagt tgtctttggt

182341 ggcatttatc tataatctat gattgtatca caataatatt ttttttctca aaaaaaaatc

182401 aattaaaatg cattttgcaa gttacgacgg tattatttcg tattcggtat gaatccttat

182461 ccaaataaat atgattttgc gttagagtat taaacgtaga ttataattgc cctttacagt

182521 ataagattct gaccaattgt ttagaagata ttacataatt aaaacaaact aagcgcgttc

182581 gaaaatgcaa cagaagcgcg cgggcgcaaa aaatacttat atactcgcgg cgatgtaata

182641 tgtttgttgt aatagtattt acacgatata tctttcttga ctattgttta taattaaatt

182701 aagatataat aattgttgtc ttgtaggcca atcagataag gatggccata atatggagta

182761 catattaagt attttgttat aaataactgc gctgacgaag tctcccgcaa caactagtac

182821 tcatattaaa tagctttaat ttgtttgttt cacatttcgt agttataatt tttaaaataa

182881 actgtttatt tcatatatta agataataaa acacaacgga cagttataat gtatatggtg

182941 tgatagaaaa attctactgt agtatacaga acagcctatt aaagacacta aaaagcttca

183001 aataaaataa aaaaaactgc gtttaaaaaa accgccttcc aaaaccaaaa aaacatattt

183061 aaataaaaaa aactaaaaag tacaaaataa cgtagtatag aataaattaa ttttatacac

183121 ctcttatgca aaccattcag tcctttaata ttaataaaat actattattt gtgagctata

183181 tctatttatt gataggtttg aaggcggagc caagccttaa acaaaataaa acgtactatt

183241 atatacagct tacttactgt tatacaattt gtatactaga gattagccaa cgtaaacaaa

183301 cctaactcac agcgcatgta ggtgttatgt atccctatta tattttgctt ggctccgacc

183361 tcaaacctat caataaatag atatagctca caaataatag tattttatta atattaaagg

183421 actgaatggt ttgcataaga ggtgtataaa attaatttat tctatactac attattttgt

183481 actttttagt ttttttttat ttaaatatgt ttttttggtt ttggaaggcg gtttttttaa

183541 acgcagtttt ttttatcaca cactattgtt gctacgcaaa agcgcgcgat cgcgcggatg

183601 acgtcatcgg agcgctcgaa gcgctgcccc gtttgccgcc atcgcgccct cgccctgcta

183661 gactccgctg cgccggccgt tgttgcttgc cgcgctgatg ccataataaa tgccgcggca

183721 gtcttcttct ttatgcgctt ctatcagcga tagttctagc gcccaatact taatttcttt

183781 aaaatgtgct tctaacgccc caaagcctat ggcgttagtt ttagcaccct cacgttgttg

183841 actgctgcct gcactctctc ggtttgattg aattatattt acataccata cgttgttaaa

183901 tctcattact tcttcaattc aacgaacccg gtgagagatc tccgtgctgg ttcttcgttc

183961 ttcgctaatc cggctcgttt ggaccatgta caaaatgata cccagcacgg cagcttttat

184021 atatttaatc tcgttggtaa cttaaattta tatattctta aacttactta aactttatcc

184081 taaactatac atatttacat ttcactaaat atcttttttg gttcctacat cgttgttcgc

184141 ttcttgacgt tacatttttc actcgttatt ctcctgtttt ttacttatat tttttattta

184201 taaaaggcaa tggccttatg gccattatgc caccaaagag atgcaattta gactgaacaa

184261 attcaagact tagaaatgtt gatactttta tggggacgtt gacagttcag tacaccgtaa

184321 gatttggctt gtgtctacta agcaaaagta ttgctactta aaaacgtttt tatcgtaaat

184381 atgtttcata tagaattctt ttattaatcc gaacgcccac cgcgaaagag taaagagcaa

184441 tttatctttg ttggtgggga cacaaacatg cagttacgta tgaaagtggc agcttagcac

184501 tttactcttt tgcgctgggc gctcaccttt attactttaa aatatcataa atttaattcg

184561 ctctatttta gggatcaaca aattctgtcc ttaatgcctc gtacgcacca ggataatatt

184621 ttagttgagt agcaagtagc atttatccga acaaaggatc tactactgtt cggactcgtt

184681 taagcgtaaa cctacgcgct aggatagtaa tttagtaact aaacgtgtcc gaacagcaac

184741 attttagtag agtagaataa taaatccttt gttcggatac ttgctactcg actaaaatac

184801 tatcctggtg cgtacaaggc tttactgaaa ggccatctaa ataagtacac aaattgattt

184861 cgatctggat tcttgtaaag ggcactttca aacttttacg tacgagcata catagcaaat

184921 gattaactaa atagtgtatt acggaaaagc atattttccc aaattataaa agtctacgtt

184981 tacttaaatt atatactctt agtattaatt ataagacgga atgacctaca acactagttt

185041 agtgctgttt aaaataaata attatttaga taaaatcatt acaaatataa aaggaatcaa

185101 agataaatca atcttatttt ttacgtaata gagtacaaaa tagattgaac taaacatcgc

185161 ttacgctcgt tataacgacg tcgttgttta aaaataacta tttttcgtaa caataataaa

185221 tcatgacgcg tatacatcgt cgctccactg acctacacgc aataaaatga cacacccacg

185281 ccgcaatgga tatgctgtaa tacgaaaacg acgggatcca agtatcaacg tagatggaac

185341 gcttttgtgg gaaatttctt tgaaacgttt gcatcacaaa atgattgtat ctcaaaggga

185401 tatttttcga taagtattaa tttctaaata atgaatatgc ttcattgata tttgacttca

185461 taggagtatg agtcttatga catgttccac atttactttt tttgttactt caataatatg

185521 aaataaacct ttgacattgt ggcgataact gttatgtttt tttctatact attatattat

185581 aaaactcaaa aactactaag gcgatttcaa aaatactttc accattagaa agctagacta

185641 tcactgagta acataggcaa ttttttccgc gcggacaaag tcgcgatcaa cagctagttc

185701 tttataatga ataaaaaggt ttaaggtttc cacccatgat gggctccatt ttcagcaact

185761 ccacgattag cgcgtatttt gcgtgaatac acatatcttt gaatttcttc gccgatatgt

185821 gcaggttgca tcacgatgtt ttccttcaac taaaagcgtc ggtagtagta aaagtataat

185881 gaataatacg gttgctatct cactccttta tgcatactaa tagtcattgt tattttttgt

185941 ttatgttttt gtgtgtttca tactagtata ggccaaaaca agagcttcaa tgggaacagt

186001 aaatattatg ggtcttagta aatggtattt tactctacat tatatcatta ctggtttgat

186061 actgactagc gacccgcccc ggctacgcac gggtgcaaaa ctgatactaa acacactaca

186121 gaaaaactgt gaatgttgta tataacataa taaaaataat tgcacccgtg cgaagccagg

186181 gctggtcgct agttatatat ataaaccttc cttatgaatc actttatcta ataaaacaaa

186241 ccgcatcaaa atccgttgcg tagttttaaa gaatcaagca tgtatagggg caaatataaa

186301 aatcattatt gttttatata atagtgtaga cgatttgcaa caagcaataa gtgcaataag

186361 atgacaattt atttgtagtc atgtgtttta ctagacatta ttcttaatgc attatttgca

186421 acaaaattaa ctaatttgca gacatactgg cagaatacag ttattagaag taatgtattt

186481 ccttacatag tataaaacaa taatgctttt tttatgtttg tccctaaata tgcttgaata

186541 tttaaaacta tgcaacggat tttgatgcat tttttttaat agatacaatg attcataagg

186601 aaggttttcc atgtataata cattaatgtt tctatctatt taatattaaa attaatgaaa

186661 aacattgaaa gataaaacaa ctgaacaaat attatcttcc tctttaattt aaggttttta

186721 gctttgtcgg tggagtcctt tccaaccctc tttgtcctgt gccaacatct tgaccttctg

186781 atacgacacg acgtccactt tcttttttat ttggtctata taacttgacc tcggtctacc

186841 tctctttctc tttccttcta taaatgtggt tatcagatcg tcgtgtcgta tcagatgccc

186901 caatacatta cattatatta tcattctttg ataataagta ctttcactta ctttctacat

186961 tcatggcagc aaataaggct tcattataat cttatttatt ttactactgt ctaacaatgg

187021 tgtttaaatt aaaaaaaata tcaagttaag tgattataag gttttcagtg tttatgatcc

187081 ataattttga aagcctaaca aagttgtaag atcatttaat tatttttaaa tctttaatta

187141 aatataatat ctaatcccta aagattagat ttaaagagtt aagagtaatg cagtaaattt

187201 agtttgttta catttttttt gcaacaaata cattatggcc aatagtttta caaagtaggc

187261 acttctaata ccgtacgcta agtcatttgt taatgtaaat aaatatgtgc cgcgcgcgtg

187321 tatttatctt ataagccgtc atagatagaa gaagaagcaa ttataatcat aatcagccag

187381 ccgtcaacgc tgggaccgcg acccggcatc caaataaaga cataaaacta tgacatgtct

187441 accattcgac aaacactgac ctgttttttg catcaaaatc ttcaaagaag tcacattgag

187501 attccattca atagtttatt ataaaaaaac aacgaacatt cacaaattca gttcatttca

187561 cacaataatc atttattttt tacgaatttc tttttgtgac actcgcggaa accaacattc

187621 ggcccgaccg ctggagcgta acacaacaga ttatataaaa aatgtcaaaa aattatcaac

187681 atcatagata taataatttt gtaaaaaaat accactttaa ttcaggaaac aataagcaga

187741 aaataaaaca gataataagt taatatctca agattttacg atttacaatc atccgtagca

187801 ttaatttcta aggagtactt tctaaaagag tttttttaat ataagccagt cagtccgttg

187861 cttttattgc tggaattttg ttataaagaa cgctccctgt aatatgtagt ccattttcga

187921 catccaagga tttatttaag atccattata agtgaattga tatttcacat gttgaaaacc

187981 agttgcaaat aaccgaaaag gaaataactg gaataagctt tactgatgaa gaaaagcttg

188041 aaagctgtca agacaaggtg tctttgattt ctttgacatg ttgactttga caattacaag

188101 caatgacaac tgtttagaat agaaattaga aataaatcaa tgcacatgat aattgtattt

188161 tctctctaaa ataatctaaa ctttagtaga atataagttg ttaaatgtgt atttagttaa

188221 aataagttct aaaattgtta atattcacaa tgattaaagc catacttgtt tttaacaatc

188281 atggcaaacc acggttatcg aaattctatc aatatttcgt aagtaacatt gtaattccag

188341 tacttttttg acaatgccct gtttcccaac gtttattttt attcttttag aatgaggata

188401 tgcagcaaca gataataaaa gagacattcc aattggtgtc caaaagggat gataacgtct

188461 gtaattttct ggaaggaggc aggtgggtag taatcatctg gttactgaag ttgtggtcat

188521 ttaaaaaaat aattgtttat tttattttca gtcttattgg tggttcagat tacaaactaa

188581 tctacaggca ctatgccaca ctatactttg tattctgtgt tgattcttca gagagtgaac

188641 ttgggatatt agacttgata caagtaagta cactaacaat aggtagtagt tctattttcc

188701 acaactccac gacaagcgca tattttgcgt gacttcacac atatctttga atttcctcgc

188761 cgatatgtgc agacagcaca atgtcttctt tcaccgtaag agcgtctgat aaatatatac

188821 atgtgaaaat ctaaaaacac attggtacaa agcgggattc aaactcggga gctgcagttt

188881 acaagtcaaa ctaaacccct gagccaccga cactctagac acatatagga taacaataga

188941 tagattctgt aactaatgtc atcacccttt aaaatatagt aacagtttgc tcggaaatta

189001 gtaaaataga tataatagct tcaaataaga tcatattaat ggtacctggt ctagacctaa

189061 cctgtcaacc actggtctca tgtctttatt gatgagatgc tggacaaaga aagtatggtt

189121 gtgtgagaga agatatgctg taaaatgggg taaattcaga aatgacgact gatagggatg

189181 tatggagaag tacatactgt gccgaccctc gatagggaca agggcaggtt gaggaatgtt

189241 gaaatgctgt caacattgaa ggtacaagta tatttaaata ggtcccgagt tcgaattcca

189301 tgtaccaatg tgtttttcaa ttttcatatg tacatattta ctgacgctct taggtgtagg

189361 aaaacatcgt gatgtctgca catgtcggcg taggaattca aaggtgtgta ttcacgcaaa

189421 atacgcgcta gtcgtggagt tgcggtaaat ggagatcatg ggtcattggt gcaataaaat

189481 gtatgtggca aaaaacatgg ttttcaccaa ttaagactgt ttttactttt ggtacaatta

189541 tatcaacatt cccaggtctt tgtggaaacc ctcgataaat gctttgagaa tgtatgcgag

189601 ctggacctga tcttccacgc ggacgcagcg caccaggtgc tggacgagct ggtgatgggg

189661 ggcatggtgc tgcagaccaa catggcggat atactgagta gactgcagga acagaataag

189721 atgcagaaag ctgaggtatg cagatttgaa ctagtcacag ttacctaaca tatgcaaatt

189781 tattaattaa gtcgctattg ttcgcgtctt cgtccgcgtg gaattaaaaa aatgtagtct

189841 acgttactcg gggataatgt agctttctaa tggtgaaaga tttttcaaat gggaccagta

189901 gttgataagt tattacaata catacaaaaa tacaaatctt ttctctttaa aataaaatat

189961 acaaaaagat ggaaagtgaa caggattatt tacgaatata tacttcagtt ggtgtcttct

190021 atgtggtcat aaactggtct agttttatgc tatggggtag tggagtctac ctgttacgta

190081 taatatcttc caggcgggta tatccgcggc gccggcgcgc gccgtgtccg cagtcaagag

190141 tatgaacctg ccgcagcagc tgcgagacat gaagctcccc gacctaccgc aggctattag

190201 ggtaaggttt ttaataaatt tatatttaaa cactaagaag tatataattt tctaagcatc

190261 aatattgcaa ccttatttgc ctcattagac caatgtcacg cgaaaaaggc cttgctccga

190321 agtaaaatct ccgtttttat cgcgaactcg gtctaatgaa gccataggga aattgtagga

190381 gagttgaagg tcgttatatc gaatcttgtt tttaagaaat gtcaattaaa tagaagaaat

190441 gtactatttt tttttaatta tgtgaaaaaa ctgtcgtaag caaactatgt acggttttta

190501 ttaaagacta gctgttgttt gcgactttgt ccacgtggaa attaaaatat agcctatgtt

190561 actcgaggat gtagcttgaa atattttttg gcatcggcct tgtagttatg gggttttcaa

190621 tgcatataaa aatacaaatc tttcctcttt gtaatacagt tgaacctgga tgtcgcttat

190681 agaggtttct ctaatcagag tttctccata gaatgactcg cttatcgctc tcttatccag

190741 gtttgactat tagtatatat gtataattta acctaccctt aacctagatt taacctttta

190801 catcaacgta cctctaggac ctgaagttct gaccgctaca gcagggcgcg cccccgtcgc

190861 ccgccggcct cgccaccaac atactaaaca caatctaccc acacgacaaa tatcataatc

190921 tccatcgtca ggactttcct aataaattct ttggctcaga aacactgagc acgagattct

190981 cggagacgtt ctcggaaaat tatcaacaaa ctgaaggaat taattttcca aattatgaaa

191041 ctcataaatc cgaaggttat caagatttga gcggaactgt cgagagtgag tatagcgaga

191101 gaaagagaaa gaagaagaaa aaacaaggga agtgatagaa aaatatataa ataggctatg

191161 gcttcattag acaaatgtga tgcaaaaatg tcctcagccg aagcaaaatt accgttttta

191221 tcgcaaactc ggtctaatga agccgtagac tatacgaata ttagcgaaaa agttgttatt

191281 catcacttgt atgagatttt tgcagcgaca ccttcgccct tgataagcga gagtcaaatt

191341 gcccgctata tttgtctcgc ttatagaggt tccgcactaa cccaattctc gcttatcaag

191401 gttcgactat tagaatcttc accgctaccg tcacaatagg caagaacacg tctatagtta

191461 tatagaggta agtgccgctt taatgtgtta ttttaaaaat tcttatttta aaacatatct

191521 ttttaaaaag ctattttctt aaaagaaatg gtatttttca tattttataa tttgggtaga

191581 tcttcgaggt cgtcttatgt tattgattaa atgttagttt ttaaaagtcc agtttaatta

191641 atgtcttatt tagtgataaa atgatcacct agcagacact tagttaagat ttctcagtaa

191701 tcaagaacag tcgaatttga ataagcaaga gtcaaaggga ctttccaatt tagactgtaa

191761 ctggttaact aaatgtaatg aactttatga agcgtcaaca atacttcgaa aaattaaatg

191821 caaaaaataa ctagatacgt caaatccata catggcggcc atttttatat tggacatttt

191881 tagttattct tttaaaagtt gttaaatgta gatgtagtaa acccatgatg ggctccattt

191941 tccaacccca cgcaagccta agggttccta ttcatttaca attaaatgac ttgagaggtt

192001 aagttaatat ttaagcttgg ccacaaagat atattatact atagatggaa cattcgaagc

192061 ctaaaagtga ggcattttgt ttgaccttca gaggtcttct gtagggaaat ttcccctagt

192121 gttgaccttt acattacatt tgccgacata aatagttgac ggaatgtaac ctcttctctc

192181 atatttaaat tttatttcat agtatttact aaagttaaat attataatta atcgagacat

192241 ttttaaaacg ctaattaaac atttataaca accataaaac aaaccatggt ctttgttaaa

192301 acttgtttgg atttaaattg ttttaaataa aataataata attaaaaaca ataattaata

192361 ttttaatgaa tgttttagtt tattttattg tttatttaaa aaaaaaaaca ttttcccaaa

192421 ttttttcatg ataatgacaa gatttttatt ttttaataat ttctttcgcg gcacgcattt

192481 taacgtcaaa ttactatgga gcgcttcctc tatgcataat atctctttgc ttcgccagtt

192541 aaaatagtga aatctttacc ctaattatct tatatatcgg gtctattata attttggttt

192601 tgttaaaaca tttgttttag ccatttttac gtttttattt atgtgcaata ctagtttttt

192661 ttatattttg atgcattaaa tactaatagc aaatgaatat tatgacattc cttgaactta

192721 tgaagttagt taaaaatgtt aatgtataga aattttcaat ccgtgaaaaa ttaaattacc

192781 ataatatgaa attgttttgt ttagtaaaat atatattagg taattattat ttaatgtttt

192841 cccgtatttg agttcgagtt gatttgtgat ttttttttca aattatgaat ctattttgta

192901 ttgaaacgat gtctgtatat gtatgtgtgt gtgttccgaa gaattgtttg gaacgatccc

192961 tgccgctgag ttccgtcatc gtacgagtag acagagtgcc aacttccatc gtcaccacct

193021 cgatggttgg cattccacaa cgacgaggtt ttcgcgcaat tttttacctc gcacagccgc

193081 gttgtggaac actctgtcct cagcggtatt tccaaacgtt tacgacttag gggccttcaa

193141 gaaaagagcg tactcctccc tcaaaggccg gcaacacatt tgtggttcct ctggcattgc

193201 agatgtccgt gggcgtcgga ccaattaatt tagttgatcc gctgctcgtt tgccctcttc

193261 tcctataaaa aaaaaaaatg tataggttgt aagaaatata aatgttaatt atgcaatact

193321 ttgttttatt taaaacacta atttattatt ttagaataaa ttacaaaaaa aacttccgtt

193381 ttcatagtat gatttttttt cttataaggt agttcaacaa tttttttgca cgccatcttt

193441 ggatgtaagc aagtaacact atctcgtttt gaaaaatgca cacaaaattg tcatcgtagg

193501 tacgctattc gacaacagat ggcgttaaat cctttgtagt agctatttga cactagatgt

193561 cgcttatttt aatatatttc aaaaatgtaa aaattgggca cataaattga aataaaatat

193621 agttccacaa ttatttagat tattaactta ctgattactt ttaaaacgtg gttgaacatt

193681 gagggatata cgattggaag ctgaaccagt aatggcttat atttgtatcg aatcttcttg

193741 accaaggttt aaattggacc tagataaagt tacgatttac aggctatgct tgagtagaac

193801 gaatgcaaga aaatcaattt tggagttcaa ctaataatta aaaaaaatga attatgcaat

193861 gaagaacgaa acaacaaaaa tatatttgca agtgctaaac taaaacagta gttaattaat

193921 gttcttttaa ttatctttac aatgtaaaca gttcacacgt aagcttacgt gctaaactat

193981 ttacattcaa aagtaataaa ttaaaccggt cagtaaacac gactgacgtg ttgataaata

194041 aaatagcaga gcgcaggctt cgttcaattg cttatatttt attttaaatg ttgcttaacg

194101 tttatgtgtg tttttatttt cgtttacgat tagtttcact cgatttacga agttttataa

194161 aaatgttcat agatggcgtg cgtttttcat aaccgatttt tcggacataa atggtgcatg

194221 ctaataggtt acctagttgt ccaagaagta ttacgtatta tactatctta cttcttactc

194281 ttactaatat tatacaagca taggttacaa tgaatgtatg tttgtttgaa gaatctcaga

194341 gagtcgtaag gaattttggt gtaattcggt atagacctgg aataacatac aggctattta

194401 ctatgttttt tttagtttcg ggcagaggaa gtcgcgggca tcagctagta ttattatgaa

194461 atgtatcttt tgtgttttaa agagtaactg cagcaaagat tcgagaattg gagtcactta

194521 ttttccaatg accaccactt gataagccac cctgcaataa tttaggaggt tctttacaat

194581 ttacgttaca atttttgtat ttatttgtaa atatgctata aatgtttttg tttgtcatta

194641 ttccgacgag cactatgatt ttcttgacca catatgatta ctgttctgtc ggtttgttgc

194701 tcgcgattta aggtctgcgg tcgtctggac gttgttttgc aaaaagctgt catttcgcct

194761 tccggcctcc attacttgga ttataattta acaagacaaa taggatgtag ttagtagttt

194821 tcaaaccctc tttagtgtta tttaatgcat attttgaatg aactagagct tgaccacgaa

194881 tgcattttag accaccaggt ttcatcctgg cgatggctgg caatcctcaa ctgtgaggtg

194941 ttcgcaaaac tttctgcccc gcacagctgc gtagtggaat ggtctgtcct cggctttatt

195001 tccaaaccgc taagtctttg gatccttgaa gaagcgagcc tatctattcc ttaaacgccg

195061 gcaacgcatt tgcggtttct cgaaagttgc aaatgtccat gggcgtcgaa gatcacctcg

195121 atgatccagc cgctctgatt ctcttataaa aaaagatttt gtcatatatt gtaactaaag

195181 gaggccataa ttttaatatg aaaaatctac ttttaagggt tttgtaagtg cttgttagtc

195241 gtgtaattcg atgctgggat gttttgacgg taattttgtg gttgcagatg acgtccggtg

195301 gtcagccggt cacaacatgt cacttgcgga cattcggttt gcgaatatca aattattgtg

195361 acttgcggtc aatgtgaaac tttctaattg agagttcggt ctgtcctggt attcaaattt

195421 aaatggcttt agttacaaat atccgtcaaa aaaaataccc aagaaatcag agttaataca

195481 gtaacagtta tatattcgct tgtataaagc tttttttttt tatgaagtgg caaatgagca

195541 gagaccacct ggattcgctg aagtagcata gcgggcgctg ccaatagtta tatgcaattg

195601 cagatccatt gtctaccctt aatctacgga ggagaggacg cacaccaaga agattcttca

195661 agattcttcc tggcgtcccc tcccccgcca aatccacttc cctttcccat catatcttta

195721 taagaaaagg ataagaacgg aaagtggacc aaaatcaggc ctgtggcacg aaacatacaa

195781 ttacttccat ttgacgcctg ttttctgtgt tgtggtggta tgtcaccggg cgagccggac

195841 aattcgtgcg atgttattaa agcgggctct accactgtca aaatattggt attgcttcat

195901 tctcttcaaa caaaacacag ataggttggt tttgtactgt tattttggca gcggatagat

195961 aaagtatgta atctagtaaa gaacacattt attaattctt acaatacaaa agtgtatgtt

196021 cttagacgta aaatttattt ccgtgacata tccgtttcaa tgttcttaat aagcaaaaac

196081 taaacaagcc caactatatt cacaaacgcc gccacaacaa tttacctttg atatatttct

196141 taaggcgcat tcacacgcaa aaaaaccttg tgactctacc ccccgcaatc acacaatgtt

196201 ttgacatttc caccggtcca ttattaactg ttatttatag ggcgcggcga cttttacaaa

196261 cgcttttttt ctctcgtatc aatagagaac ttacctggac tttatcatat atcaaaattg

196321 ctttcgcggc gctttaaaac atgggtggtc gcgcaaagtg acaaaacata acaaaataaa

196381 cctttttttt ctttacattt ttataataaa tcgtatttaa cttaagtacg attacgttgc

196441 atgacttttg ataaattaat tttgcgtttc attaaagttc tgttttaatt aatttttgtt

196501 tggcagaatg agtttattgt tgtgggctgt ttaaaaaaat gtaactacaa ctagtaatag

196561 acgttcgcgc caaaatgaat taaaccattc cgtgtttcaa cgtaattaaa aatactaata

196621 cttaatcttg atttcttaat taaataacct ccttctgtta gaagtcggtt aaaaatatgt

196681 tactgggcta tttcttatac tttaattgca aaccagattg gtcggctttt gattatttct

196741 tatttaatac tggctatgaa acacggtcta aggtggtgcg acgtcattac taagatccca

196801 ctgctgacac tgctggacca gactgacatc ggacaggaag acatggaagg aactggagga

196861 ggctttcacc gctttcggcg gcccttacac agacgatcaa gaagacaagc actaacaata

196921 gtaatactat tttttaatac tattttctta tattataata tcaaacttta gtaaatacta

196981 tgaaattaaa tttgagaaaa gaggttacat tcggtcgact ttttatggcg actaatgtaa

197041 tttagaggtc aatattaggg gaaaatttcc ctatggaaga cctccggagg tcaaagataa

197101 tgcctcactt ttagccttcg agtgttccac atatagtata atatatcttt gcatgcaata

197161 ctataaaata caatttacac acgaggctaa aggaaattga tatccgatgc attaataatt

197221 aaaaaaaatt acatacgaat agtttttcct agaaacaagt atgttacgaa atattgtaag

197281 taaaaaaaac taattgactt gataacctac ttctgttgga agtcggttaa aaagtgattg

197341 gtgtttcatt gtaggtaatg aaacttcgac attaatgtac ttttttatat ttatcgcata

197401 tattgaaaca atccttgatt tatcgaacga aatgtatgta aaaaatataa tttaaattaa

197461 aatttgattt taataaacat aacctacgtt tgtagtgcga tccatgacga gctccattct

197521 ccgcaactcc acgactagcg cgtattttgc gtgacttcac acatatcttt gaatttcttc

197581 gccgatatgt gcagacatca cgatgttttc ctttaccgta agagggtcgg ataaatatgt

197641 acatatgaaa atctaaaaac ataatggtac atggcgggat ttgaactcgg gacctgcaga

197701 ttacaagtct agagctaaac ccctgagccg ccgacgctct ataaacatag gttactcgta

197761 caaaagaaaa taacgcagtt tttatttcca taatcagaag cgatgctatg tttttagttt

197821 tttcgttgag cttgaaatct tgaaattcgt tttattttaa taaataaaac atcattttaa

197881 ttacagagcg taaataaaca aagtcattgc aaaaatatta aaaaaacaat gtcaaattat

197941 aattagtata attgcaacac tcttttagtg tcttatactg gatcatcttt gtgtgaaaca

198001 ttaagggatt cccttagtag tcaattaaag attctgagtg ggcgtgtgga actatcatct

198061 ccccgtcctt attctactcc cttcgtgtcg gtgcaccagg gttttgtcct cctccacgtt

198121 atcctccgtc atgtcagtcg tcacaccctt gtttatcata tcacctttca cacaatacat

198181 ctcttcctag tccttcctct acctgtacac ctctatagta cttgacgctc gtaactgtaa

198241 gtttataaaa aaaagatcga actttgttga aatgatatta cattttaata atttataatc

198301 taccagactc tcataaataa taatgagacg tccaaacgac cgtggtgaag ttcagtttta

198361 tttcgtagct aaaaactaaa atatatagtc atccattcgg ccatagatta tgaagttatc

198421 cgacgaaaat tggccgaaac catagcccag aatttggaat agacttatga ccaaactttt

198481 taattatgca ggacaaaaac tacaggtttt tatttaataa tctacatatt ttaaaataaa

198541 ttaaaattca tgtttgtgta atattatgat tttgttccaa acgtggtatt tattcattct

198601 tactttgata aaataactca tttcttaagt tatgtgcata atattccaac tgaaactttt

198661 ttgctgtaat agtaaaaggc gataaaggaa gtatacatat caacggttgt aattatccgg

198721 tcatattttg tagcatatcc accctacatc tatgacagcg tcaaaaaaat accacagata

198781 attattattt tacggagcct aagtaaatta tatgtgttct gtgggagcta gagatgggac

198841 ttcggccatt ttgtcttgag ccgtgtcatt tggtacggtc tcaataaacc ggaccacttt

198901 atatcggatc tatataaatt attaatattg tggtcgacca ttttgggtta gagtgaaaaa

198961 gatggatgta atttgaatgc gttagaaaag gttgaatgtt atttttttta tctcttgagt

199021 gtaggggcgg cgttcgtttg tgattattac tgttttctaa ctatatggtg tttcgttaac

199081 tgattgtaaa taattattaa aaaatacaat tgccataaac ttaattaagc ctttgcttac

199141 gtttaatttt aattttttgc taatattaaa aatgaaaaag cagagaagga tgtctgttta

199201 aagatatttc aaaaaccgca gattgattca tgttgaaatt tagtacacat gtagaacagt

199261 ctagaaaaac aaataagcta tgaagttgtt attttaatta cacctgaacg gagtcacgga

199321 cgactgccag tatttcaatg tacatttaat tacaattatt cataaacata aactacacga

199381 taattgacta aaaaaaactc tgtaagcact atttctaaca agtcaattaa ctcgccatgt

199441 tacatagtga ttaatatcta tgcgaaattt atacattttt aattttaaaa aacaaacccg

199501 ctgtataata taaatgtaat agtttcgtct tgagatgacg cacacaggcg cagttacgca

199561 cacccgtatt gtaatgtttt tatttattac acggattatt agggttcagc gggttagtaa

199621 gaaaaacctt ctataatttt aatttaggca gttattttgg agaccgcacg tatagtgagg

199681 aggttccttt ctctgcagcc ctgaacactg tttttttttt atatatatga gaagggggca

199741 aacgagcagc ggatcgccta agttagttaa tccgacgccc acggacacct gcaacgccag

199801 aggaaccgca gatgtgttgc ttgaggcatc ggattccgca gctggtggtg cactattttt

199861 ttaatttact atatagtcga acctggataa gcgagaaact aggataagag agaaacctca

199921 ataagcgaga gttactgtat ggtccagagg gacgatttcc ataagcgaga aactcggatt

199981 agagaaaaac ctcaattagc aagattagtc tctgtaattc ggtcccttgg actctcgctt

200041 atccaggttc gactgcaata ttgttacgag cttgggtgtg agatagagcg aaaagcagcc

200101 ttagtatggt gcaaacaaca tttattgagc gacaaggtcc acacggcact ggctacttct

200161 tccgctgtcc tcttcccgga ctcccaatag cgccgctgcc tgcttctccg ccgtccccat

200221 cccggactga tttcatttgg atttggacgc ctttatatag gccgaaattc cgaatttgtg

200281 ttctttattt taatttaaat acatctattt caaaatattt cttcgaacct ctgtagtgtt

200341 gacactggca cttataatcg atttgatgac atactttggc gtatgccaat ttgactgttg

200401 acggccaccg tcgcaacatt atattattat gtcttgtaat atgtagacgg cgggattatt

200461 acggaacccg aattttaatt tttcaggctc gtcatttcgt gggatacgta gaaaaacacg

200521 taccgcttga aatctctgta actaattagt tcacaaaatg atacaccttt ttttcgctat

200581 ttcctttata ttctatgttt tgattttctt acactacggc tttattagat ttcttcggat

200641 ttttgcttcg gcgtatgtcg caaaatcggt ctaatgaagc cgttgggtaa aaacgtgata

200701 tctttgtgtc tatatttgaa aaaggttgta ttagaatgaa aaaataaggt acttttaatc

200761 gcgatgtgaa aaatataaaa aatataaata tattaaaaag agaattgtcg ccaaatattc

200821 ataaaggtag aacaaaagtt tttgtttttg tctaaaattc ggaatgtttt tttttaactt

200881 taaataaata cgtaatttac ttgtaataac agtattaagt gaggaaggta aaaacattta

200941 aaaatctaat ttgtataaat taaaaacaaa gtgcagtgac tggccgaaaa taagttggta

201001 aatattttat cgcgaaccgt cagcggtgtc gcattaaatt acaaaaaaaa caaaaaggtt

201061 tattgacccg tgaaatattt taaatgatat ttgtaaagtt ttgcaggcaa ataatttcac

201121 ttttgtgtag cttgtttgtg tagctatcgc tcgcaattcc cttcgaaaaa aacttagtca

201181 tgatatctta cttcttatct tatcttacta ataataaatg taaaatattt gaaaaaaatt

201241 atctccagag cggcagaacg gtctcgatga aacgcagata tcttctatat agaaaacagt

201301 ctggaagaag actacttact gtattttttt ttaaattctg cgcggacata gacagctagt

201361 actaaaataa atataaataa agactatata aaaattccat attcaaaatt cagtagaacc

201421 tcatcggtat ctcgagcaat ttttcattcg tgtctttaaa aaaggatttt accttttttt

201481 aaatgttttt aagcaaagtt ccaggtaact cgcggcaagc ttataccttt atgtttaaaa

201541 caatttattc tatactgttt caagttatgt tccatttaaa taaactttaa attgtgcata

201601 ataaagtttt aacgtctata ctgaaaataa cagaaagtat tgttaatctt tgttagaaaa

201661 tgtaaactgt tttctacaat aagacataaa agccacgaat ttcttcacat acatttgctt

201721 agtagattaa attagttgat gactttttta ttaataatta atctagattt aaatatgata

201781 aaggaattct tcctaatcta agtatactaa tattataaag tggaaaatat aaaacgaaca

201841 ccaaaaaaaa tacttaacac caaattgatt tacccaaaac aaatataata agaataaaac

201901 tgtaacaaat atatgacacc ctaaattaca attcatctac ctatttaaat cacgcttcct

201961 taagaaaaaa tattttcaca tcaaaattga taaattacca cctaatttcg accatcaaat

202021 ttttgtgctt ttataccaaa atatatttta agtcaaccta aaaatgtatg catataacca

202081 aataaactat aaagatacca aaattatatt atattatatt ttatccgtcc gttactgcag

202141 ggcacggaca cactcgctgc gccgctcttt gttattcgcg agctgtgatg ttgcgcttgc

202201 tgattgactg aagtgcttct aaagtaaaaa atatgcagat ctcactaata tacaagtctt

202261 tatatttaca ttactaatta tgattttttt agatcgtttc atttaaattc aagtcaatta

202321 aaaaccgcgt acctactgca taggttaggt taggcttaga acatgttgaa taatataaaa

202381 acaaacaggt aactaaaata aaaaaaaaac tattaatcga attctaataa actacaaact

202441 aagcttcata aaaaataatg atgtaagtct tttcaggtcc taagtatatt cgtatacgct

202501 taatccaaat atgtggtatg ccgttagtaa ttaattgaat aatattcata atgtttaacc

202561 attattgata atcagcaaaa cgagagctaa aagtaatcaa ttaatcacat tcaacgggga

202621 ttaagtatta atactgcaat aaaaaagaaa ctgcatgggt tgggttaagc ttagaacatt

202681 tttttttgaa attttatggc ctggtaacac ttagaacatt taaaattaaa tcaaagacta

202741 ggttatgcca ggtccaacat ttttaataat attaaaaaca caacaataag cgaaagtaaa

202801 aaaggatttt tttccttaaa attaattaag actccaaaaa tatgtttgaa aaaataagag

202861 tattttctat tatttaaaat taacaattgt gaatctgacc taacattcgt tgcccgctct

202921 gcgtgaagcg ccccttatgt cactcccaca tacatcagct aaaatagata ctaaaattat

202981 aaaatatgtg aacaataatt taaattggtt atagtttact attttaacac aaaaatatgt

203041 tacatatttg agtgaaattt tgcattaaat tgaggttata aattaggtgc aactttatta

203101 tttttgatgg caaaatgatt ttttggtgat agtttttcta ttttgattga aagactacag

203161 atattttggt ggtaatttta tattaaatgt ggatcgaatt taggtgatca tttacttttt

203221 ttgctgataa taagtaaata tttattttgg agatcaaatt tattattttt ggtgatcgat

203281 taattacaag ccatttttcc acgcggacga agtcgccagc aacagcgcta gatagtatcg

203341 aaatggcatg aacttaattc ttgtttcgaa atgtccggag tcataatcgg tttgagagga

203401 caatgacagt tttttataaa atcaggcgcc aaatgtaaca taattactta aagacactat

203461 tacgtctgtt atcgataaag gacaaatcag taacaatggt cgaaatcaaa aaggtccccc

203521 agtcccttaa aaagcccaag ggtcaagtga catccgtaaa aattgccgca attaaactgt

203581 agcaagaaaa agtcatttta aattctacat tcctaaggga cgttcgggaa acaatggacc

203641 attaggagac cgtagataag tatctaagac aacgcttctc aaactttaca aattcgatta

203701 ctaatgattt tttcattacg atttttatgg gctggaactt agaggtaaaa aacttagtaa

203761 atataatgtt ttacattaat taaataacga atattaattt aaatcgcaat ataattaaca

203821 aatgttagtt acaaatattt taacagtttt tttccaaagt ggactttaac cgctgacgta

203881 acgaccgaca aaattgacct taaaaaggac tttattaatt tagaaatcta gcttaataaa

203941 tacaaaataa ttaggttcat aaagtttgag tatcgctttt ggacgaccca attgtagatt

204001 cgtttttggt attttagaac gtttgtttgt ttgtttgttg tgaaggttcc ggcggagacg

204061 cggcgtggtg tcgatttttg cggctaacga tttggaaaca gcccgaaatc cagcaccggt

204121 ttattgagtt cacgattttt tttaaataat aaaaacgact tgattttatt atgtttaaat

204181 atttgtggta tttattaaga tgtgaattgt caaacgcgaa taaattaaca aaatctctag

204241 ggatataaaa cattttttaa cttgatattt tcaagtttta cctcgtaatt ttctgggaaa

204301 gctatttgct tgtcagtttt ttaattgtca atacaagttt taatttatcc ttaacgtttt

204361 aattaacact tagttcaata aaaacctaaa attcttttgg ccaactttgt gctgtaccat

204421 aaatgtaaat gtaattgttg ttttgtaact tttatcgcag atgaaataaa aattatataa

204481 ataaattaaa gtattactat aataattcgc ctcccaaaaa caataaaagc tttatacgaa

204541 aattgcgtgt ctctgacagt tttagtttaa tgaaatggta agattttttt aaataaaata

204601 tcggatacca gcatcaaaat ttttcaaatg acatctcaaa tatgtcatgg cttttgactt

204661 tttttttaaa aaacaatagc gcgcgaattg agaccgcaca cgtcgaaaaa cgttcatctg

204721 catatttcgc caaataacac gtaccccctt ttatttattt tatagtgcct gtaaagcttt

204781 ttctacatac gcttaacaaa tgtctacttt gcaataatag atatatctat gtaatggttt

204841 ttcagtctta attcgatcct tttattcttt tccgaatttc tcaccctatg gcctcattag

204901 tccgagttcg cgataaaaac ggagtaacgg cgaaggaaaa tctccgttct aattaagccg

204961 tagggtcatt gcctactgta aaaaaatcaa ataacattaa gaacaatatt tgcacagagc

205021 tttttcaaat ctacgacaga taaataatta ttcccgccat tcgtaacgtc acgctgacat

205081 taaaataacc gccttcctgt tgccatagat aaagggttta aatcgctttt ttattttacc

205141 agtagaatag gattcggcat tagggagaaa agccaatcct tatttaatga caaccaaatc

205201 gtaattcttt catttcaatt cgagtcacag agtgttttag gagcggaagt gtcgaacttt

205261 tttagtataa tcgacccata atgctacttt ataacatccg gttaggtatt ttgtaataga

205321 atatagtttc ggtcgagtaa ttttccgaat atgtaaatgc gagcataaat aaagaatttt

205381 gtagtgtaca aaatgatggt tgttgattat aatcttttat tgtttgttaa taaataagca

205441 atttatttac tgttcgacat atctttagtc ttatattata tttatttatc aatctcgaat

205501 cgcgtttact aacattacat aaatttaaat tgaaaataag aattcatcta catttattac

205561 aaacgtcggc gaacgaaaaa gaaaatgtcc cacagagtac acaaaaaaac catttcaaaa

205621 tggacccaag ccgggactat tgtaaaaggt aactaatcca ctttcatgcg gtgttgccaa

205681 agtgttgcct ccagcgacga acgttcggat tttgaaaata ttttgacatt aatgaccgcc

205741 tttgtgagcg gccagtgacg ttcaagtaaa gttttgtcga tggtctttgg ttttttttta

205801 tttttttttg cgaaagattt gggttaagca ttgaagcgtt gtaaagttaa ctagttttgg

205861 gtgttaactt tttttctcga tatcgtatca aaagttatca caggtatcgt tatcttttat

205921 aggatatttt atttgatata tattttattt tgctttaata gatttgttat tgtctgtttt

205981 tcgagatgtc gaacacaaga aacattataa attaaaaatt aaactataat agttatgagg

206041 tactaataaa tgaaaaagta tgtatagatc attataatta taattatttt taaacttact

206101 cattacaaat gtgatagtat gtacatataa cgtgaccata ttttttgtta ctcaaaacgg

206161 gacaactata ttatttggcc ccacatctag aagtagggga tgcaaactgt ggtgaaaatt

206221 ttaaacgaaa ttagtcaaca aagcgcaagc gataacgttt tcgccactct cgtgcaccag

206281 gctatcaatg cgtcgatgcg gtcttttttt tgtgactgaa ggtcactgtt tgcctatagg

206341 aggcatttac tgattcaccg ggcttgagat ggcagggcgc agatggcgct tagcctaaac

206401 ctcgtcgatg cggtctcgcc ctcaccttat aatgtactgg tcggctcaac tatcacaatt

206461 gggtgtttga cactatcaaa aatctgctat ggattgtgat catcctatga gataatagtc

206521 taaaatggct tatttcgata tcaatcaatc aaaatagtgt atttttcgaa tatttcacga

206581 tataaaatac aagatttatt ttgatttatg acatttcatc cgcggcatga aacgccttgt

206641 gatttgtcac ggcaactgtg acataacttg ctcaatccaa acgtaaacaa agattccgca

206701 attgaggttt cctgattttg atgttttttg gaatcgatct gtgatttctg gaagctattt

206761 atacgtatac ttaattaata cactacttta taaataagtg gtttgaattg ttttctaaat

206821 aaaatggaag caaattttgt tcgtgccgat aacagcaacc tatcgctcat agacgcatgt

206881 atggttagac atttttttaa taatagttga tgctgattat catggttcag aactgaaaaa

206941 tattaaaaca gcaatgtaaa tttgaaattg attgtttaaa atcccctgga ataattcatt

207001 tattattttc ctaaaatcta atcttacttt aggtttgttt tgtcttatta atgccagtcc

207061 cctgtttctt ggttattgtt gttgtttcac aggtctacaa gggaatctta tggagactat

207121 gtgtgtttgc cgtacaccag atagttcatt gtttggtgcc ataatgggtg caatattact

207181 tgataccata gcgatgaaga gaggtagaaa tttagaagta tctgtccgca aggcagtgtg

207241 taaaaaacta aaacaaaaaa acacttcaag tggccattat ctttgcaaag agaattcttt

207301 agttgctgct tctcatgatg gttgtacgga atgcagttgt agaaataaaa tgcccaccaa

207361 caaactagtg gacttgctaa tttgggcatt ttaaattgtt aagaggcaaa aactgttgtt

207421 cgtccgtgta catacaagga cttcataaat gtgtttttac tccctgttta cattgtaacc

207481 aataggttaa cacctattta aatgtgttac attggcggta tgttgcacct gcataacgtt

207541 tatttttttg taacacgtgt ccaaggccgg ccacaaagac ggtcaaagtg tcacatgcga

207601 tcaccgcagg aagtggctgg caacttacaa aactttagcc acccgggcat tttttgacaa

207661 aaaaacttct ataatttatt gttctacata gatatgaaat gtgacagatg caaggacgtt

207721 atatactaag aggactgcgc acttcatagc aagaaaacgg tccgaggaga gccgcaaaat

207781 atgaagtagg ggaacctgtg gtaccttgga catggttgta cctaagacaa tcgtttattt

207841 taaaaatatc ttgcgctgta aatcgatctt gcctatggca tttaaaacat cgtttgtcaa

207901 ctcccgcgtg tagttattta ctatacgtcg gcaccgcgtc agtgtttatg taaaaggtaa

207961 ttattgtttt tttgcctaaa aagtaagata actagataga actttatgcc aatttattta

208021 acacgatatt tatttctcaa aaaaactatg attgcaattt gttcttgatg tattcctcta

208081 ataaatgctg tgttgcttgg acagttttcc cttttttctt cgggaactta aaactctcaa

208141 ctaaacaaaa aaatctatca tttgatatat gtaatgtaat aagtaattaa ataacagatc

208201 tttacacatg tgtatatttg ttcaatcttg acctcaatat ttatttttaa aaaatcaaaa

208261 tgtaaaaaac tgtccaaggt accacatgtt cccctacgtt gtcccgttct aatagtgtac

208321 ccataatttt aataaaatcg tggacattca caaaacactg tacatgaccc ttaaattccg

208381 tacttggtta agagatgaaa acctctacta tgataggaat gtttatttgt gtctatcaaa

208441 gctttgatat tttcatatgc ctattgtgac gaggaagaaa ttaaagatca tatttatgtt

208501 aaattctttt taatgttgaa tggccacaaa atacaaagtc aaatcgctac aaacaattta

208561 ttacaggttg atagactata aatgctctga gtagttaaag ttttagaact tgaaaacatt

208621 atcgacttta atttcaagta atatttataa ttatcttaga tttacaaatg tctctgtaat

208681 cgatttcatt tagattcaac cttaccgaag cccttcttgt aattaagtca gtagtactaa

208741 tgttcttaaa gcacaccttt tgatgacctt gcaagttttt catgcactat attgagccat

208801 ttttgcctta caaatctgtt catttttggt aaagctaaaa acaaaaaata tgcataggta

208861 aattcatctg ataaacaatt tgtacgaaaa ttcttcattt accgtagtct agtggtcacc

208921 ctacttgttt atcataacaa agttgttagg cttgaaaaca aaccactaca aacttatttt

208981 ctacatccac gaacagatca ctttaacacc atactcagcg aaaatgtagt cgaattcgtt

209041 ttattttaac aattaatcta tatatataaa agagtagagg ggtttcttaa aatcagcgca

209101 cagccaaaac tactgggtct agagacttga aattttgtac agaggttact gttactacgt

209161 agatgagcac taagaaagga ttttgagaaa ttcgacccct aagggtccga aatgggggtc

209221 caaagtttgt atggagaaaa atgattcccc tgttagaatt gcatgaaaat tagaatgagt

209281 gctctttacc atgaacaaat aagtttctca ttcaatattt ttacaatttt ttactttggg

209341 gggcaaaaaa ggggggtcaa agtacgaatt taaaaaaaaa atctcgcagc gtaggtacga

209401 atcctagaga cctgaaattt ttcgtaggag tttttgttac tacgtagttt tgcactgaga

209461 aaagattttc cgaaattaag cccctaaggg tccgaaacgg gggtccaaag tttgtatgga

209521 gcaacgtgat tcctttgtca gaatagcatg aaaattggaa tgagagctct ttagcatgaa

209581 gaaatcattt tggcattcaa gaattttaca attttttacc ttggggggca aaaaagaggg

209641 gtcatagtta gaatctaaaa atattttcac agcgtaggta cgggtcctag agacttgaaa

209701 tttggcatag gcgtttctgt tacgacgtta aagtgctctg agaaaggatt ttacgaaatt

209761 caacccctaa gggtccgaat agggggtcta aagtttttat ggagaaaagt tgttcctatc

209821 tcagaattac atgaaaattg gaatgagagc tcttcggcat taataaataa ctttgccatt

209881 caagaattta ttttgtttac cttggggagc aaaaaagagg ggtcagagtt cgaatctaaa

209941 aatttttttc acagtgtagg tatgagtctt agaaacttga tattttgttc atgggtttct

210001 attactactt caaacttatt ttataaaggt taaaataaga tataaaactt tgttttgaat

210061 ttctaaactt caagcggacg aagtcgctgg cgaccgctag tctgacataa ttggctactg

210121 acatatcaac caccataatg gacagttgac ttttgtagtg aggcagttca acaacatatt

210181 tggtacccat aaacttggta atattttatt attatctagt tagtttgtaa tagtgttttt

210241 aaatagtttt cagtgttctt aaatgggaaa atataaaacg accaccaaaa aaaataaacg

210301 taacaccaaa ttgatttacc caaaacaaat ataataagaa caaaaaaaat aaaactgtac

210361 caaacatatg atactctaaa ttaaaattca tctacctatt taaatcacgc ttccttcaga

210421 aaaaatattt tcacaacaaa attgataaat tagcacctaa tttcaaccat caaatttttg

210481 tgcttttata ccctaatata ttgcaagtca acctaaaaat gtatgtaaat aaccaaataa

210541 actatattga taccaaacta tattatatta tattttatcc gtcagttact gcagggcacg

210601 gacgcactcg ctgcgccgct ctttgttatt cgcgagctgt gacgttgcgc ttgatggttg

210661 actgaagtgc ttccaaagta aaaaatatgc agatctcact aatatacaaa tttttctatt

210721 tactttacta attatgaata ttgttttaaa tcgtttaatt taaattcatg tcataaaaaa

210781 taatgatgta cgtcttttca ggtcctatat tccgtatacg taagcgtata cacgcttaat

210841 ccaaatatgt gttacgctaa tggtaattaa ttaaacatta ttcataattt ttaaccatta

210901 ttgatatatg aaatcattgt tgtttagtaa ctttcattgc agatcaaata aaattgaaat

210961 aaataaataa tcagcaaaat gagagctaaa agtaatcaat tggtcacatt taacggtgat

211021 taagtattaa tactgcaacc ttgttaccga taaaagtaat aaactgcaac aaaaaccaac

211081 ctgtgtgggt tgggttaagt ttaggatatt taaaattaaa tcaaagacta ggttatgcgt

211141 gaaacgcccc ttatgtcact cccacataca tcagctaaaa tagatactaa aaaaataaaa

211201 taatttaaat tggttatatt ttactatttt agcacaaaaa tatgtaacat atttgagtga

211261 aattttgcat taaattgagg atataaatta ggtgcaactt tattattttt gatggcaaag

211321 tgattttttt ggtgatagtt tttctatttt gattgacaga ctacaaatat tttggtggta

211381 gtttttatat aaaatgtgga tcttaaggtg atcatttact ttttttggtg acaattaata

211441 actattttgg agatcaaatt tattattttt ggttatcgat taattacaag cctttttaaa

211501 ttatatatat gtatacgaac aaaaggaata tgtacttatc taattcagta agcaatttta

211561 aaaccactaa atacgacttt tttgtcagaa cccaataatt ggaataatat ttaattttgt

211621 atcaaatatt ggacacttta tctttgatat taaatacttt ttacgacaaa cttccatttc

211681 gcggtcataa atttgtttac tgattatgat acttaaatct aaacagacta taatggaagg

211741 acgtatataa cgaaaacatt tgttaaaggg tttgtatttt tagtgtaaaa aatatttggg

211801 aaattgggaa aattctaaac gataatgaca acactagaaa gggacaacgt acttcatatt

211861 ttgcaactct tcttggaccg ttttcttagt atgcagtgcg cagtccctta gtataacgtc

211921 cttggacaga tgtgattttt cgcgcgcaat agtgatgttc attttacaaa atctacatgt

211981 ttaatttact gtctttcttg ttgcccgtaa ttcagctatt ttataaggtc attaaaatcc

212041 atataattga ataataataa gttttgttct acaaattaat gaaaaattca ttttcttaaa

212101 agcgagctgt tgcaactact gcagatttgt tttcttattt ctgaagttat tggactcgat

212161 ctacgctgat taattttaga tataaatttt gtttttggtg ctttaaaaaa atctacatca

212221 ctggtttgcg tgcaaatgaa tttatggcgg gagaaatgcg cacgtttttc aactctccaa

212281 aggcacgcaa atattaaatt tgttctgtaa ataaaaaaaa aacataaaag actaacaagc

212341 ggacaaacat cacctgtcaa ccgcaaactg accatcgtaa tgacgaccct acaatacaca

212401 atatggtatt gtctggccca tgcaccagta tacttagccc tccaagttct ttaaggtaac

212461 tcaacagtat ggctcaaatt acacatataa ccagcaacca agacctctga ttccgtttcc

212521 ttttgttgga gaccgaaatc tttttagact ggattttaaa ttgccgtaat aaatagacct

212581 aagtttaaga aaaataataa gaaagcttga gttcaatttg taattgtcgt gtatattttt

212641 gtttgtacga aggcgcgtgc tgagaaaggc ggtgtattgt tgtgttttgt taatttttag

212701 ggtacaaagg aggatgttgc ttttgactga ttgtttcgaa attgtaattt gagccggcta

212761 ctggttttga ccagtttttt tatataagta acctttgctc aaaatgtatt ctacacgaag

212821 ttgtacatac atggttaaat ctaaggcctg atattaataa taaaaaacaa gaataacttg

212881 atatgattaa ttttaactat tttggattac gtgactgcaa ctacaatttc cattttaaca

212941 ttttcaatga actcaaaaaa taataattgt tcagccgtaa acagtaataa aaagcaatta

213001 tttattggca tgctgttatc tttcccccac ggagtattcg tgcgagtgaa ttaatttccg

213061 agttctgatt ctggcaaccc cagccaccct ctataaaggg atggacgacc ataaacggat

213121 tatggcttgt tgtgagatta ggtttgttta ttcaatgttg tgacaaggac aattgtttga

213181 ttgtcaagag ggtgctgtgg tttatatgaa gatcattttt aatgttccac actaggacaa

213241 taatgttgtt taacattgga tactttattg aaaacaagta agatcaggtc taaaataacc

213301 gtcagtttta actagtttag gtttatattc aggtaagtag acaaaggtag aagctgctcg

213361 atgaaaaatg gcgtttaaaa aaaaagcatc tgagtatttt ttaaaaaaac agaatattat

213421 actaagtaag tattatacga tataacatag aaattagtaa cgttcgtgga aaaatagggt

213481 tgctattttt ttctatcgca gaattttgaa aaagttttta ttttattaat tttagaccat

213541 ctaattagtt cataataccc atatttaatt ctaaataagt atgtttattc aacaatttgt

213601 aataatcctc tatcatattt cttcgtttct actcccccat gattcgaagg cgtgaacttg

213661 ctccccgtga acccccggga gatgccgcaa tgatttgagt tttctttaca aatgcgatat

213721 gattgatcaa agcttgtgaa ataaatgaaa tatgtactac attgtctatt tatgaatgat

213781 ttccttttag gataaatggc aaagaaaatg gtaggtatac agtttacatt agcaaatcaa

213841 gaatgtctat tcttaatcct aggatcaatt ttaataactg ttgtttccga aaatttaaac

213901 taaaccaaaa tatcactcac ataaatgtag ctaagtatat agattataat ttgaaaaaca

213961 caattgcaac ctttgtcgtt tcaattaaat aaatgctcga agcccacctc ctttgagccg

214021 tcacaggcgc aaaaagaacg cacattcagc caacagccaa cagaccggcg acaaaggaac

214081 gatcacaaat tacgtacgca atttacatag aaaaagtgac aacgaacgac gcggaacatt

214141 tgttagcccc cattgtattg taaagtatta tatcctcgcg ctttttcgcc ttatttatat

214201 ttataaatgt tcgtatttgg tacgtactaa caagatgttt acgcgcggga gtgtcaaagt

214261 gcccgccaaa atcgacttat agggatgtgc cacggtacca aaattatctg tttcatctta

214321 aacttatgct tcagtaattt ttgtctccgt ttctagatgt actcctttaa aaacaaaata

214381 tttatgacag tggtttattc tgcataggaa tttcgcgtct atttgttagt tgctatgccc

214441 aatcgctaaa taaaaaatta aggtttcctc aatatgcgca attttatttg tgacttcata

214501 aatattacgc attattaatt ttattatagg caataatatt taaaacatcg tcatcttaca

214561 tccctagtgc catagacagc gaattttttt tacacttttc ttatttcggc aaatctttaa

214621 cgtacagcga tgcggtaggt gtgagccgat tgttgttgaa atgtctacgt aacttcttag

214681 caaagtgatg ttattcagta agcttgtaat cgatgctttg atatcaagaa ttgtggttta

214741 acgtaacgta aaattactac ggcgacgata gattagtatt acagtaatgt atcctatcgt

214801 atttgcatta ggcgctcttt tgagtttatt ggattaaaga aatttctatc tcgtagggat

214861 tttataagga tttttttagc aaaatttctt ggaacgtata tgtagataga catgttatag

214921 atgtgtaact accgtaatcg tcgtgtaagt gtaactagtg acatatttta tacgaatttt

214981 ttaaattgta tttatacaaa ttcgccatta aacgctgtca gacgcaagaa attcataaaa

215041 tcattttaaa agaaaatgga ctaatatatc ttaagataga agaagtgaac tttattacat

215101 ttgggcgctt tgtcagcaag tgggtccaat aacgcgttac ttttggctca actccaataa

215161 aggcgggaaa aaactttgct ggatcttgaa accacagaca agtgcttgta agcacatgtc

215221 acattcaata tggccgttcc gttatacaga aaaaaatctt tattgccttt ttcacactat

215281 aagtttattt acttgtattt gtatacagat aggggttttg tgtaattgaa tttatatttt

215341 gtatttcact acacttatgt acgttttata aaagttttaa atcgtataga aaatacaagt

215401 tgccagtcta taacaatcaa tacagcgtct tacgagtatt ttactttttc tgacacactg

215461 tcagcatgga acaacattga tgcaatggtt acagcatttt atttcattta ttaaaaaaat

215521 aacttttgtc tcacaaaatc ttgaaaattc aatcatccaa attggacata aatattaaca

215581 aagtggattg tacagttcta tagacgatat tttacgctct taaatcgaat actcatttaa

215641 ttgacacgta taaaacataa aatatcagta ataaaattgt tcccttcgaa atataatcgt

215701 gacatgacaa ttttacgacg ccatcttgcg cctaacgacc tttgattggt tatgtttcgg

215761 agaatttatg tgatgctgga tgttattgtg tggtttcagt tagtaaagca ttaacgatgg

215821 aaaaggaaat ctaatatgaa tgacgaatga acgatttgta ataactagaa aacaattttc

215881 atgttgtaac gtggaaatat tttctcttta ggggctcaaa gtaacacata ttgaaattat

215941 ttatataatt actttttgta tgcaagtctg ttacgatttt taacagttat tattatgttt

216001 gtaactaatg tgcacaacag gtgaccttta tttagggaac gtcaaaacca tttttctctt

216061 aatttaacat ttaaagtctg tttttgttgg ttcgtgttta atttaaaatc tactttatct

216121 gatctcgaat acaaatgacc aatcaacgtt tttaataaac agcgaacccc atgccatcat

216181 attacagaga aatacaattc cccatatcaa aataacatca tcatcccgac tacaaatcat

216241 cgcttgaaaa aaatctattg tagcgccctc gtggctccat aacatcccta ataacatttc

216301 gaggtcccta ccacaaagta cttataaaaa cacccaataa catgcgttaa tgtggccgaa

216361 aacacgcagc tgttgtctcg atgaaaaaac taaagtccgc cgccattggt ccgcttggga

216421 tggcgggaca ttagccgcat ctgtcagttt cattttgtct atggctgggc gtattcagca

216481 agaacagttc catccatatg ttgttagata tttatgtgaa aatattgcaa taaacgttac

216541 gatctcttac ctgatatttt atttcatttg atataaaagt taatgaacac tttcagcagt

216601 ttagtattga taatgtttat aaatattgtt tttaagttgg cagtaccgac taaaaggaaa

216661 actggttatg aagctgtaaa atgatacttt gttccaaaga tatattatac tgtagatgga

216721 acactcgaag cctaaaagtg gggcattttc tttgacctcc ggaggtcaaa cctagggaga

216781 tttcccttag tgttaacctc taaattacat ttgccgacat aaaaagtcga ccgaatgtaa

216841 cctctttcct caaatttaaa ttttatttca cagtatttac taaattttaa tattgtaatt

216901 aatagagaca tttttaaaac gctaattaaa aatgtataac aaccataaaa caacaatagt

216961 cgttgttaaa aattgtttgg atttaaattg tttttaagaa attgataata attaaaaacg

217021 ataattaatg ttttaatgaa tgttttagtt aattctactg tttatttaaa aaaaaattaa

217081 aaacacaatt tcccaaactt gggatttttt ttcatgatac tggcaagttt tttttttaat

217141 aatttctttc ttggcacgct ttttaacgtc aaattagtat ggagtgctcc ctctatgtat

217201 aatatctctt tgctttgttc ttttaatatt gaattcaaat caaggcatta caagtatgcg

217261 ctaacattat tttcaaattt ataaatctat ttaaaattgt ataagtaggt acaaatatta

217321 acgcgtataa acgcaaaaaa acgttatgaa gtggcaaacg agcaagcggc cacatggatt

217381 agttaaaata gcgaagcgat cgttgcccat agacatttgc aatttcagat gcgttgcctc

217441 cccttagtct acggagaagg gaaagcacag caagaggtta attcctcttc atttgtgtcc

217501 cctcccccgc caaatccact tccccttccc atcatttctt tataagaaaa ggataggaag

217561 ggaaagtgga ctaaaattag gcctccggca ccacactcgc cagacgaaac atggaattgc

217621 ttccatttga cgtttgtttt ctctaggtga gttgtataga caatcttaac tttctcgtct

217681 gaaataagat gttaattgtt tggggccccc tataccattt atcttaatag gtaagtacct

217741 agtactcaac aatttgtact cgaaaggtta ctaaagtctc taacgtagtt ctatttatat

217801 ttcttcacaa acttttacaa tgaactacaa ccgaaagcct atagggttgt ctataaacca

217861 aaaaaactct tcacctggta cggtaactct gatgtacgac caaactgaat cgcggcgtgt

217921 cttatcacga agtacgccgc gcacttgaca aatatcaatt tatatgttaa taccatgtaa

217981 ccagggttgg cacgttaaac acgatatgca gtactatggt aattttaatg caattcaaaa

218041 tgattgagtt tccattgtat aaatgctaaa gtctttaatt tgaattgcag aatggagata

218101 cgtagtaagt cactaattag tattcttcat attagaaagt ggccgacatt aatgaccaga

218161 aaacctagta agtatacaga ataatatggt atgtgtgtag tttttgcttg acttcacaca

218221 tatctttgaa tttcttctcc gatatgtaca gaataagtaa tcatgtacct taggtaacta

218281 tatccgtttt taaagtgaca gataaaaaag aagataaatg tttgaaaatt tatattatgt

218341 tataaatgat tgtagtgtca accctagtta gtaattggca tacaaaggaa tgtgccgtaa

218401 atttgaaaga ggtttaatta agtacatgtt ataaaaatac attttgttga tttaaaatta

218461 ctgcaataat ataactattt aaaataacaa cgtcagctta attataaagc aaggatgaaa

218521 atataccgtg tcaaacaaag tgtgtttctc ttggcagggg ttattatcac agtctctaga

218581 gcattatatg tgaaatcaag cgttttaaga taagttatgt tgactatgta ctcttaagct

218641 cttgcttgct aatgcttaaa agtgctcgtt gaggctaaga agccgcgcgg aaaattaaag

218701 tgtctggaag gcaagtttgc ctcatccaaa gtggctgcct ccttacaggg aatactgtca

218761 tagtcccttg agcattatat gtatgtgaaa aacagctatg tgaatataag ttaacttcag

218821 taatatttac ttccacttta acgcgtgatt gcctatttgc ttatagtatt gatacgactc

218881 ctatctaatc atatattaat aaagcttgtt aataagtaag gtctatcgcg tttccgatcc

218941 cgatgcaaaa ttgcttccat ataaacggcc gtaaggtatt ttattggaca ataaatgcat

219001 cgtatttggt attgaatgtg ccattttgtc ttttaatatc ttgtagcgag aattgaataa

219061 gtaacattta tttacattgc acaggtgaca cattcttttt aaatctttga aaatattaac

219121 ttttaactat taaccttttg acagccaagc aaaaacacca aaagcctgtg agccaaggct

219181 caaaatcgta agaaatcact aaaatatcaa tagtgcttgg agcagtcatg tcgataacgt

219241 gcaatatatt catatatttt aacgactatc taataaaaaa ctttcttaga tgtaaattta

219301 tttttatatt taatgttcta cctacgaaag tcacaataaa tgtcataatc tagactttaa

219361 aagcagtaga tacaaatttt aaattcaata aaatcgatcg aacaaaagtt atggaacagc

219421 actatcccag cgatgacgat gtccgtggtc agaagtatgt gcgacttggc tccctgggcc

219481 aagcatttta aaaatgcacg caatacacgt agagatgaaa ctgaagttaa ttttgtttat

219541 tgacattcga gtttcaaaac cattgtcaga aaaagtataa tatttgtaaa accggactct

219601 ttaccgttaa tattaagaag aatttagtaa aaaataaata agtaaatagc acttatgtat

219661 agatgtttca taaagtttac ttgattgaag acgtcattat tcaaactgtc tatattaaat

219721 attctgtctt gacttacgac gtgtttataa atattagttt atgaagtctt tatacagcat

219781 ctatcctata cctacattta ataaagttag tactgacact acgaaaatct aatttaataa

219841 ataatttcag tctgtctggc cgcaactcac ttttgaacac ttcacttttc acaaaagtga

219901 aaccaaacga ctaggctgat tttgaaggga ctctcactga aagacatgtg atgcgtgcga

219961 gaatacgatt gcaatttttt cctgacgaag ctgccggtct actgtttttt ttagcacgcg

220021 gcatgcaccc tgggaattcc atcgtcagca cattcgtaaa tgcagtgaaa caaaatgtac

220081 tatatgctcg cacatatcag acacatttca gtgaaagttc cataaaaatc ttgcggtagg

220141 caccatcttc gtcatttcta aaaaaagtac tctataatat tctcgcacac atcagacacc

220201 ttccagggta aatcctaaat cccgtgaaaa tttgtttagc cattgcgaag ttatcccgga

220261 acgaacgcgg tatacgaaca gacagacagc taaaagttaa aaaacaaaat tttgtttgac

220321 aatacattat gtgtatgaag tgataatttc gtttcgttcg acaatatcgt atatattaga

220381 ttcgatatcg atatcgtata catacacaca acaagcctat ttatacggat ggaatatgaa

220441 taattttcga gtaattcaat gtacctttcg catcactagc tgtcataaag tgtcacaact

220501 agctatatac ttttagattt ggtgtttaaa attaccaata agggttaata atgattatgt

220561 catgttttgc gagggcatgt ttttcgtcac aaatagaaga ccgcggctca cagagcactt

220621 catcgtttgt cttctatttg tgaccgtagc tgccaaaagg ttaacctttt aacctaacgt

220681 ttttttttac gccgatggac atgtgcaata tcagaggatc cacaaatgcg ttgccaacct

220741 ttagcaggtg atacgctcgc tatttgaagt cgcctaagtc gtaagtttaa aactgatctc

220801 tacagatgga taggctcttg taaatgatag cttctggatt ccctctaaac gcggatgttg

220861 ataatactgg cagtttatct ttattatttc gagtcctcaa caccgctaca aaatggtaat

220921 tcatgcacat ctaccgccca tgttaaccga gggttccttc cgcaaaacag gaatacagac

220981 tattttccag tcgaactata attcagaagg tattttcaca agcggtcacc tctatccccg

221041 cagccttaat aaaagagcgc ttatgtatac catagctgac cacgaaaaaa gaaacgaaaa

221101 gaaaaatcga tctaaacgac ggtttttttc aaatcagcgc acagccaaaa ctactgggtc

221161 tagagacttg aaattttgta tagaggttac tgttactacg tagatgagca ctaagaaagg

221221 attctgagaa attcgacccc taagggtccg aaatgggggt ccaaactttg tatagagaaa

221281 agtgattccg ttgtcagaat tgtatgaaaa ttggaacgag tgctcttcag catgaataaa

221341 taagtttgtc attcaagaat tttacaattt tttactttgg ggggggggca aaaaatgggc

221401 atgaataaat aactttccca tttaagaatt ttacaggttt ttactttgtg gggaaaaaag

221461 gggggttaaa taaaaaatta aatgacggcg tagttacgag tcttacaata gaaacttgaa

221521 attttgatca agggttcctg ttaccacata gactagctcc tagaaaagat ttaacgaaat

221581 tcaacctcta agggtctgaa atgtgggtcc aaagtttgta ttgagcaact tgctttcttc

221641 ttcagaattg catgaaaatt ggaataaatg cttttcagca tgaataatta acaatgccat

221701 ataagaattt ttcaattttc aaaaaaactt tgttttgatc ttctaaattc cacgcggacg

221761 aagtcgcggg cgaccgctag taggtacata atcccagtgg tgttttctct aatcggcggg

221821 aatggagttg accacttttg aaaataagct ccaaaattat taaagtattt ccttgttcaa

221881 gatttttcat cataaaataa tccgattttt gtattttctc cgtgtcatag atacgatttt

221941 atagtgaaaa cgatcagatt tcaaagtctg cgaaaggtat tgtttacggt ttgaaatgca

222001 aactaagtaa agaaaaattc aatatatttt caagcgctat tcacatagtg ctcaatacac

222061 aaaacctatc ggatatctaa cgggtgtaaa atagttggtt ggtaccttca tattttgagt

222121 ttgatctttg agttttccta tttttaaatt agttctttta tattttacta attacatagt

222181 ttacgtaaaa ccaacatata attatttaat aattttatta cagaaaaaga aaaaaactaa

222241 accatgtggt atactggcaa ttgaactagc tattaaaaaa actgaaagac agtagatcct

222301 ttttaaatct tacgttttaa taagcgtgta atgaatttta aaacttaata cgttaccatc

222361 aaagagatat tatacataga gggagcactc catactaatt tgacgttaaa aagcttgcca

222421 gtatcatgaa aaattttccc aagtttggga aattgtttta tttattttgt gttatttttt

222481 taaaatttaa aataaacagt agaataaact aaaaaaatca ttaaaacgtt aactatcgtt

222541 tttaattatt attattttat ttaaaacaat ttaaatccaa acaattttta acaaagacta

222601 tttttgtttt atggttgtta tacattttta attagcgttt taaaaatgtc tctattaatt

222661 acaatattaa attttagtaa atactatgaa ataaaattta aatttgagaa aagaggttac

222721 attcggtcta ctttttatgt tggcaaatgt aattcagcgg ttaacactaa gggatatatc

222781 cctacatttg acctccggag gtcaaagtaa atgccacact tttaggcttt gagtgttcca

222841 tctacagtat aatatatctt ttgttaccat actaaaaaat aaaataactt tactatggca

222901 ggatgcatga ttttatatta tctaattaca gtgcattcaa atgtttatta ctttttaatt

222961 aaagcaaatc catatgtcta attgggcccc taccctgctt tgtaaaccga aggaggcagg

223021 gaagcagaga tgtgggtaat ttgtgctagt tagtacattg cgccccactc gccccacttc

223081 ggtgacgtaa acaagagtta tgatatccag aaggattcac aagagggcgc tgtggacgat

223141 ttatttgtat gctctgtgcc tgaacatgtt ttgaaattag aacagctgcg gtttgttttg

223201 tttgatagac tattttcggc cagtttagca atgctcacga taaaaaataa aaaattatat

223261 ttttcaactc gaataagaga gtagactttg aatattaatt gtagagtgtt tattacatat

223321 attgtatata tattttttta ttagtaggca atattaagta tgaggttaga cctatagtta

223381 ataaaaataa tggaagtggc acgcgtaacg atcccaacgg tggaacatat cctttttaca

223441 gtaaaggttg aaacaataca aacaatagac taaaagcatc aacgcacgca cgacacaatg

223501 cattaaagag gacattgtaa acaaatcaat gaacaaattc gcgacggtgt aaacagatca

223561 ttgtctgtgc tcaggtgact tgcaacgaca attcttaact ttttctgcga cctgtcaaac

223621 ttaacgtcaa tattttggcg cgattagtga cgcagcgggt taacatgtaa ttggttattt

223681 tcatttaaat caaacaaaaa tatactttaa gtgttagtaa taaggttgtt ttcgattgcg

223741 tgttatgtta agcaaccgta gaatgcctaa taagttacgc tcggagctaa tgaattgaat

223801 ttaacttcgc tgcgactgtg ggttttctta gaaagtccga caaagttatg tttacggttt

223861 gaaaataatt attgtcgatg gtttacagaa tttaaaagtt atgaattgtt aaaatttggt

223921 gtaaacctta aagttgtgat tttgtttgac gtgtagcttt gtgggtaaat tgattatcag

223981 gtgttttgcg atttgtgacc gttatggtct cattttgtaa gggttttgta tgtcatttga

224041 cttattgtag atatttttgg tatgtccgcg gcgagtatga aattgtattg tattgttaac

224101 atacaatgtt tacaatagta gaaatattta tttcgtggtt gagggtttta tgaaaagtca

224161 tgtgcaaaag tttagtatac tattaaaaat ttgaatttag ggcaccgata ctataatgaa

224221 aagagcgcgt atagcacttc tttctggggt tgaaatcttt ctgaaactaa aaaaaagttt

224281 taaaaagggt atttcttcaa aatatatttt taaatatttt caactttttg tcaccggaat

224341 attgtttata aaaaaatcaa ttttatttct tttgcttaag ataaacccca tgcggctaac

224401 taattacgtt acatacaaaa tattggtgaa agcaaaccaa agactgccat aaaggtgttt

224461 ttaaaattat ctcaaaataa agctaacgct tatgacagcc ggaagcggat gatgtagcgg

224521 gtaacggata gcgttttagc ctttttttag ttataataac atgaaaagaa cttgtaaaag

224581 ttaattgatt atgttgtata tattttttgt tttataaaaa ctgttacaag attagtaata

224641 ggttttttgt cgtatataat tatcagtgtg tctcaacagg ccacataacc tctcggtaaa

224701 cgtttcctaa tagtagtaat agtagtagta gctacgttcc cgcaactcca cgactggcct

224761 gttttgcgtg agggaaagtt tggatcagtc cagccagctc agctctccaa agaaccccag

224821 cagccctttt atgttgctag cgatcccggt gtgatgcgat atttaatctt gtaaaaccgt

224881 gccctaacaa tgatcagaag aggttagcgc ggcatgtggc caccactttg aaaacgtcca

224941 ttgtgttagc tgtcaaaata tttttacttt ttttaatgcc gcatctatag gtcagaatgc

225001 ccgttcggcc agcgccggcg tatatcaatt tgaactaaaa attgtttgaa tcagtacgtt

225061 ttcatatgtt ttggtgtttg aaacttattg tctaaagttg atgttagttc tataattata

225121 atgtgtgttt aattttaaag gtgtaagccc gcagagacac acacaggcgt ttaaatagaa

225181 caaatgcaaa tgcagccgtc gctattatgg cgaatccatg cttgtttgtc actttataat

225241 acaaaacata atatagaagt atgtacaagg tttctctgta ctacttactt acttaaagtc

225301 ctatatcccc gaagtgggcc agctccagcc gccggctcgc cagatcgctc tgtcctgggc

225361 tctgcgtttg atctcgggcc acaacagccc gatggccttc atgtcggcgg acactgagca

225421 tcatggtttc tctgtagtat gtaataaaaa atcttgcctt tcagcctcta aaatgtgtct

225481 aagaatgggc tagttgaaat cgttcctctc aattatcacg cttacatctt gattgccgga

225541 atgtcgcttg acgtgtataa taacaatgcc aaagtgtgta attgcaatgt cgacatttca

225601 tcacaccagc acttcgagct ctacagctag tttttttaaa catttcaaag tgaatgaaaa

225661 cggctggaaa agaaaggtac tgttattttt aattttatta attagttttc gaggcggaaa

225721 gatacactgc cttctgggga aaaaacatta ttaattactt acaaaattat ttaacttaag

225781 tataataact atccatttat taaatatgat aagaatatta ttttttttta tcaaaacggc

225841 ttaacggatt ttggtgaaat tgcatattga tgtctataca tctcatctat aggactattc

225901 ctacaaggat attaaggcac ttttttgaca gtaacaagag gggctcctgc aaatttttag

225961 gcataacagt aggtaaaaat ttcctaactt ctttcaaaat tcctcaaagt catatcacaa

226021 aatataaata agagattttc tacatatcac aaaagtgccc gttgtaacgc ttactattta

226081 aatatatgta atatataata cacacaaatt gacacagctt taaaattata catgaaaaag

226141 gtaaaagtca taatctacat aataaaagag taaactgacg caactatcaa cgcacagcct

226201 aaactgctgg ttctagagac ttctattttt gtaatgaggt ttctgttact acgtaaaagt

226261 gccttgagaa agaatcttac gaaactggac ctgtaaaggt ccgacatggg ggtccaaagt

226321 ttgtatgaag gaaagtgatt cctttgtcag aattccatga aaataagatc gagtgatctt

226381 cagcacgaat aaataacttt gccattcaag aattttacaa atttctattt tggggaaaaa

226441 agtgggatta aagtttgtac gtaaaaaaaa ttttcgcagc gtaggtaatg agtcttagaa

226501 agttgacatt ttgcacatgg gttaccatag gtaccaatac ttcaaattta ttttataaag

226561 gttaaaataa gatgaaaaaa aaatgaactt tgttttgaac ttttaaactt caagcggacg

226621 aagtcgctgg tgaccgctag tttttaataa aataatgttc ctctcgagta atcgcacgta

226681 agcataatga taccggaggt cataatggaa gcaaattaaa ttacatcgca cacggatttt

226741 tctggaattt caaaattata aaaatggcaa tattatcgtg ggctgcagcc gttgtctgat

226801 tggtcaaaat gtgacgctat caagatgaca ttgttagttc acatattttc cgagtgccat

226861 agtatctttg tagggctata gaacacattg tggaagaaca cattggctac ttgctttttt

226921 tagttccacg tcgacggggt tgcggaagat agctagtgat ttagataatt cttaaagtaa

226981 ttaccaaata tatattatac tgtagatgga acactcgatg cctaaaagtg aggcattttc

227041 tttgacctcc ggaggtcaaa tgtagggaaa tctccctaag tgttaacctc taaattacat

227101 ttgccgacat aaaaagtcga ccgagtgtaa cctcttttct caaatttaaa ttgtatttca

227161 tagtatttac taaagtttaa tattataatt aatagagaca tttttaaaac gctaattaaa

227221 aatttataac aaccataaaa caaaaatagt ctttgttaaa aattgtttgg atttaaattg

227281 ttttaaataa aataataata attaaaaacg ataattaccg tttcgatgaa tgttttagtt

227341 tattctactg tttattttaa aaattaaaaa aaaacataca atttcccaat tttgggaaat

227401 tttttatcat actggcaagt ttttttttta ataatttctt ttttggcacg ctttttaacg

227461 tcaaattagt atggagtgat ccctctatgt ataatatctc tttggtaatt acctcgaatc

227521 ggacgtcaat attaattcaa aggtactcgt attttaccca taactcataa gtaatttaaa

227581 ctataattat ttattttcaa aattaattaa aacaagagac cggtcaatga cgtagtaatt

227641 atgataacag tacctttaaa cttatatatt ttgagtgtta caataaaaac tccgtcgcgt

227701 cctaataaga aaattatagt gttgccaggt aaaagcttca tttattcgta attactgcac

227761 atcaggttgt actcaacgcg aaataagttt tgatcactaa ttgcaaatat ttcatacaat

227821 aaaaatacgt attacaagag taataaatgc tcaaatcgcg tgttttgaac gtaaaggcaa

227881 agagatatta tatatagagg gagcgctcca tactaatttg tcgttgaaat gcgtgccgag

227941 aaaaaaatta taaaaaaaaa aaatcttgcc agtatcatga aaatttataa tttataaaat

228001 aatctataga aaaactagaa catttattaa aatgttaatt atcgtttttc attattatta

228061 tgttatttaa aacaatttaa atccaaacaa gttttaacaa aggctatttt tgttttatgg

228121 ttgttataaa tttttaatta gcgttttaaa aatgtctcta ttaattataa tattaaactt

228181 tagaaaatac tatgaaataa aatttaatta tgagaaaaga gattcagtcg actttttatg

228241 tcggcaaatg taatgtaaag gtcaacacta ggggaaattt ccctacggaa gccccccgga

228301 gttcaaggaa aatgcctcac ttttaggctt cgagtgttcc atctatagta taatatatct

228361 ttgcataaag gtatacttcc actttcagtt aacgctaatt gaatgtaaaa taggtagcat

228421 tattttgcag tgttaagtac atgcgcctta taagatgatg tccttcttaa ttgtattttt

228481 aagtactagc tgtcacccgt ggcttctcct gcgggaaaat ataaaaaaca tattgttcta

228541 ctatttttaa tttttgtatg tccaaaaggt actatttaat acagttagtg ttcctaacat

228601 tactaatatt ataaatgcga aattgacaat ggatggaaat atatgaaagt atcttcagaa

228661 cagctaaacg gatgttgatg aaatttaaca gtgatgtaga acataagagt atggaagaac

228721 acattttttt ttataataca aagctgcaat cgagcaaatg cccaccaaga ttcgctgtaa

228781 ataccgaagc gacctctgcc catagacatc tgcaaaacaa caccacactc accagaccgt

228841 acacggaata gctggcatgt gacgcctgtc ttctgtgtgg tcgtggtatg tcaccgggcc

228901 agccacacaa ttcgtgggaa gatataagcg ggctctacca ctgttaaata gactacatag

228961 actacttact acttttttat tccacgccga caaagttgca caaaagctat ttttttatac

229021 atgaaaattc accatttaat tccacgccga caaagtcgta caaaagctaa tttcttatac

229081 atgaaaattc tcattaaata catcatttaa acggtagtct atttaaataa catgttatca

229141 gtatattaaa aaacaaaagt cacatatttc cagacatttt tgtagacggg ttacgcttaa

229201 tttggtaaac aatgtcctac tttatacaaa caacaattaa atgctcaata catgtttaaa

229261 attaaattca atttcgaacc aattaagacc ttcgtcagaa atatttttaa ttttaataca

229321 taccagtgtt tggcgtaaat tttttaacga ttacttaaag aaattgaatt ttaatacttt

229381 gaatttaagg ttataatttg tattcttcgt gtatccgaac ggaccacaca cgtcgatgcg

229441 gcgaggcttt taaaaaagga acgcgccaac cgcgcctcgt tcgttacctc atcatcggtt

229501 tcttttttca attttgtttt ttttttctca acccgcggcg taggctatac atagagtgat

229561 ccacacacgc acgcctccta tgtgttgcca gctaaacgtt atactcgtaa gccgaataat

229621 gttgttgtgg ttgtattatt actggccgat tcgggcgagt tttcggtctg tagtataaat

229681 tagtctgttt tattagtccg ccgcttctgg tgcgccgcgg tgtaggtagg taggttttta

229741 tagtttttta aatatcctct taagggtttc gctctttgaa gattagtttg ttatcgaccg

229801 gtaataaagt aggtgagggt atagcttact ttgagatatc tttttttatt gaaacccaaa

229861 gaggtcggag gtgcgtaccg ttggggatga ttattgtcgg taggcttgga tcaaatgatt

229921 attttaaaaa gaatacataa gtagtgactt ttacattaga tattttatta acagtgtctt

229981 aatgcgtaac acatactacg tattacaaac tacgcattgc tacgaacttt tctgattgtc

230041 taaatttaat tatacagact gaaaacgaat aataatgtac aaaaataatt gaagaaaata

230101 acgggaattc ttcatcacgt ttttttttaa atattacaac tagctgttgt gcgcaacttc

230161 gtccgcattg aacatgataa tatagcctat gttactcaga ggtaatgtag ctatctaatg

230221 gtgaaagaat ttttgaaatc ggcccagtag ttcttgagtt atttcaatac atacaaaaat

230281 acaatgtttc tctctttata atattagtat agatgtggca aacaagcacc tggattaact

230341 aaaatacaga tgcgaccact gcccatagag atcggcaatt gcagttgtgt tgcctagcct

230401 aaaatctaag gagcatacag caataggata attcctcttc ctatgcgtcc cctttcggcc

230461 aaaacgactt acccttataa atgttttttt tagcttgagc tacgagagtg ctacgaggtc

230521 tacagaacta ataacagtga tctagaaagg gaaaaaaaaa tctacacgtt ttattgtggc

230581 ctgcttagtc tccaatcaca tagcgaaaga tagtatgtag gtacctacga ttctattaaa

230641 aatgtatgta tgttcatgta attgcttaac caaaatgcca tccttccgcg taaatgttag

230701 tttaacaccc tctcttcctt ttttaacaca aataaattta tgataaggac atgtatcaat

230761 aatgagaaca gatttattta tttcaaataa ataatctaat tattatattg aagttttatt

230821 taaagtttaa gttgggttcg tatcataaaa ttcgattcag caatttaaaa acgtaaagca

230881 aatacaacaa tggcatttct aatcgcggcc agcaatataa atattcattg cgagaaatcc

230941 tcaaccgata aaaaataaac cgttaaaatg cgttacggtt atatcaaatc gaaattaaaa

231001 ttttgcttag gaaaacggcg ggaaattaat atggccgctg gagattctgc gcgggaaaat

231061 acaataactc agtaatttac aaattgccta cgcattaatt taacaaataa ataacggatc

231121 aaaatctatt tttaattata aaaaattttg ctgtaaccct caagactgat gcatgatagt

231181 taagaggtat atgatattaa agttttttaa atgacgtgat tttctaaact gaaacgtctg

231241 tacacaatca tattttcacc cttccattgt ttaataactt attgtcaaag atatattata

231301 ctgtagatgg aacactcgat gcctaaaagt gaggcacttt ctttgacctc cggaggtcca

231361 atgtagggaa atctccctaa gtgttaacct ctaaattaca tttgccgaca taaaaagtcg

231421 accgagtgta acctcttttc tcaaatttaa attttatttc atggtattta ctaaagttta

231481 atattgtaat taatagagac atttttaaaa cgctaattaa aaatttataa caactataaa

231541 acaaaaatag tctttgttaa aaattgtttg gatttaaatt gttttaaata aaataataat

231601 aattaaaaac gataattacc gttttgatga atgttttagt ttattctact gtttatttta

231661 aaaataaaaa aataacacac acaatttccc aaccttggga aattttttca tgatactggc

231721 cagttttttt ttttaatttt ttattttttg gcacgctttt taacgtcaaa ttagtatgga

231781 gtgatccctc tatgtataat atctctttgc ttattgtatt aaaatccggg gccaagtttg

231841 tcattatctt ctgaaggaca catctcgtag aaaggtacaa atgtagtaca tagtccgaaa

231901 gaaatcatag actattgata attttatttt taattccgcg cgggcggagt gtaacaggta

231961 cttaccacat tactccaagt gtgtgcagct acgtggtcgg taattttaag aatacagcaa

232021 cacaactcac gcccgtaacc cagaggggta ggcagagaca acggaccttc atttggcacg

232081 atcctggcac acctctctcg cttcttctac attcgtacat ctacgcatac acgacggtta

232141 cgcgtactat taataccggg cttcttgagt aattcctcaa tttgaccggt ataagaccga

232201 atattttggg aatcaactgt ttttatttca tttacaatgt tggtatgtgt ttagtagttt

232261 gtacaaccga tctttacgct tcaatggtca cccttgtcac gtatcacaaa acatttgctt

232321 ttttataaat ggtaatgtaa acaagctttt ataggaacaa tttaaattaa attactgctt

232381 tcaccgacga cagacttttt agggcgactg tatctatcta tcaaatctaa cttaaatgta

232441 agaaatactc acttgtaaat attattaaaa agtctatttc gtttatgagt ctctggtact

232501 acagctttgg aaacgtaaaa tcccaatact tgcaaaataa atatgaaaat tgttccttca

232561 gtccttaagt gaagtgaaat ttggtcacca tacccagcaa tccaggacca cacacgtagc

232621 ttattaaaac aggaatcata acaacagtaa ttatattcag ggcttataat ttcgcgcgct

232681 tgtgagccga gcggcggaaa aaaaacaata ttaaaacata accttacctt attaatgtgt

232741 caacaactca atttatggcg ggaactcgac ggtgacgccc tctatgcagg aaatgtccgc

232801 caataagaag caagctgttc aactcgtatt gaacgccaat acatctaact atctccttct

232861 atttagctaa taaccagctt tagatggact attgtgttct atagttttct cttgaacacg

232921 tgtaaaaatg ttctaagtcc agggcgcggt ggtccaatgt aaattggatg ctcaatgttc

232981 aagttgatat taaaagtttt ttgaggcaaa cgataatgag cgctaggcca cgatgacatt

233041 attaatttat gcgtcggcgt gctttattta ttttgaatta gattatctgt aattatttgg

233101 tatttgagac ggtctgttta ttatcttcta ggtatattta attgtatcta atatacctat

233161 ttattttatt acatctaaat attctggtgg ttacattcga atgcgtgata attacaactt

233221 atttaaagaa tcgtttaact agattatttc gaaacgaaac tatttcttgc tgattttatt

233281 atttctgcat gtaatctggc tggatgtcga atgtcgcggt gacgacgtga tgtgaggtta

233341 gataatttgt attgttaggt aatttgtatt aacatgttaa gttatgtctc gccacactga

233401 aatagtaaaa ttaagcttca aatccgatcc agcattcaat aattgataaa atttaaattt

233461 cccagaagac actttaataa tagaaaaaaa gaagaaaaat aataagtcgt ctgctttatt

233521 acttttcttc tcatcttacc tagtctagga actgcattcg aactagatta aaacaagaat

233581 gttttttttt gttattaatt ttatacaaat atccatagtt attatgtgtt caactgataa

233641 aagatatacc ttcatccctc ttattgtctt ggaataaaca gagataagca ggcaggcagg

233701 gcaggatttt agtactggaa aatagataag ataaaagagt acccggcaga ttaaaataga

233761 tcccaatatt tagtaaaaac agcatacaca ctaaataaat ctgtatctat taagaattat

233821 ttactcccac ctctagggcc actctccatg taattatagg cctaacaaaa aatatatttc

233881 gaggtcggcc atcttgtttt cctttgggca cgtcattacc tcgaaatgcc ggctgcgttt

233941 aaaaacttaa tttcgtcagt cgttagtgtg cgaacgtttg ggccgtacga gggatatagt

234001 aatcagacgg gattgcctta atgtactttt ttttcgatcg aatttaaacc gaggcaaatg

234061 tactaggttc acacgcgcat agattaaaaa taaatccatg ctttatggtt agatttattt

234121 gagttctaga agataccgtt tattctgtta atgattaaat gaaattaaaa cacaataaca

234181 ttattattaa gaacccaatc ctaaattaaa ccggttctaa aaattaacaa atttacgacg

234241 gactgtgaca aataaattca cattttcctt gaaattgttg cttaattttt cgtattcaaa

234301 tataaatttt ggcgcggttc cccgtcgacg ttccgaaacc cgcgtcgtca taaacaacat

234361 attccacaaa cttacgaccc gattaataca ttcataaaac aactgtctct ttttaataca

234421 tacagaacca tatatccgtc tctcgttatc gttttgaaaa tgtaacactg tgcgagcgca

234481 tgaaattagt gccgcttttt catctcccgc cgcggcgcga tttgaatttg gaagcatttt

234541 tttttcatcc aacgccgaag gacgtcgaaa gcgacggcca tgttggccgg ttttgttttt

234601 ttttattttc taattatcaa tgttccaagg caaagatact taaagccgct ggtcttctgt

234661 cttctttttt taaagaagaa atttatattt ttcggtagaa tgtcatttgg gtaaattgcg

234721 attgtgtggc tgtatcgatg attaccgaac cagctacagg ctgttgtcta aatatttaat

234781 tatttttaat ttatactaat attataaaga ggaaagattt gtatttttgt atgtattgat

234841 ataacttcaa aaatacagga ccgatttcaa aaagtctttc acacacatat tttataatac

234901 tgcgcagacg aagctgcgag caaaagctag taagaaaaaa ggagctataa gaaaagctaa

234961 taattacatt aaaattattc ataagaaaaa tttacctaac agcaatatgt cctaaatatg

235021 cagatgaaag aacagactac taaactgatc gagtaggatt tttataagat tatatgaaaa

235081 gaaatataaa agtattttta cgtttatact aaggaaagat gcaagtaaaa actcttcatc

235141 gaacggctca agaaaacctc tttacatcac aatcctagct tagccacaaa aatgcaacga

235201 ttaaaaagct acttaataaa aaatagacca cgctgtatct ctcgtttgaa ttttttcctc

235261 gatttcctta aactttgaga ttattatgtg tgccggcagc agatggcctt tacctttttt

235321 tatcttattt ccctttaaga cgccgagacg gccgtttatt aattgcttca gaattagtgg

235381 cgtttgatta agatctggca actttttaag attagttacc tattttggta gttttatttc

235441 ggtcttgaga tgtttagata ttttaagtgg aaatcgattg gtggattagg ctggttatat

235501 aaatagaaat acatatagtt tgtttgattt ctggaattta ttgtttgggc atcgtaagta

235561 tgtggtattt ttgattatcc aacttgaaat aatatacatt gtttgaagct attgtgattg

235621 taagagacga attaataacg actaattagc taaaatagtt atataaatat aactatttta

235681 gcaatatata tatctatact aatattataa agagagaaac tttgtatttt tgaatttttg

235741 taattttgtt tgtattgaaa taactcaaga actagtgggc cgattttaaa aattctttca

235801 ctattagaaa cctacattat acccgagtaa cataggctat attatttaat tccacgcgga

235861 caaagtcgcg cacaacagct agtctatact aatattataa agaaagaaac tttgtattca

235921 tgaatttttg tatttttgtt tgtattgaaa taactcaaga actacaggac cgatttaaaa

235981 aattctttca ccattaaaaa gctatattat ccccgagtaa cataggctat attatttact

236041 tccacgcgga cgaagtcgcg ctcaacagct agtatattat aaagttatac aaggcgcagg

236101 ttaggccgca tatggaatac tgctcgcatc tctgggccgg agctccgaaa taccagcttc

236161 ttccatttga ccgcgtgcaa cgacgtgcga cccgaatcgt cgacgaccgg ggtcttaccg

236221 atcggctcga ctcactggcc ttgcgaaggg acgtcagttc cctttgcatt ttctaccgcc

236281 tgtaccatgg ggagtgttcc gaagaattgt ttggaacgat ccctgccgct gagttccgtc

236341 atcgtacaag tagacagagt gccaacttcc atcgtcacca cctcgatggt tggcattcca

236401 caacgacgag gttttcgcgc aattttttac ctcgcacagc cgcgttgtgg aacactctgt

236461 cctcagcggt atttccaaac gtttacgact taggggcctt caagaaaaga gcgtactcct

236521 ccctcaaagg ccggcaacac atttgtggtt cctctggcat tgcagatatg ggcgtcggac

236581 caattaattt agttgattcg ctgctcgttt gcccccttct cctataaaaa aaaaaaaaaa

236641 agagagaagc tttgtatttt tgtatgtatt gaaataactc aagaactacg ggaccgatgt

236701 caaaaattct tccaccatta gaaagctaca atgtccccga ataacaaata atatatatta

236761 tttaattcca cgcggacgaa gtcgcgcaca acagctagtt aataaatata aaatttacta

236821 gaaaataaac attatattga tgtaattgtt gcgttcacca caccttcagc tatggtaggt

236881 aaatagaggg cattataaag ccgcgcacaa agcaggggcg ctggaggcgg tacattaggg

236941 gcccattgta agcacacctg gcctctccca accctgatag ggtcaccatg atggtgtttc

237001 aaggattgta tccgggttta caaccagaat acatatgata gagcatttga ctgttgtgtg

237061 gtaaaaaata gttatactta caaattatct acttttagct acgtaaaagc tgttgccatc

237121 tatttacctt ctaccaagat atttttagta agtataatat tgaattggat tcaatcttta

237181 acaaagcaga ggcaaaatta actaccaata ctaatgctgg ttttcggtga actccactcg

237241 ccaattaacg aaaccgctta gattttttcg taatttttat tacattatca actgttctct

237301 aacattcggc tcaacacaaa atcattcttt ctctgaaatt ttgtttaatt tccaaagttc

237361 ccagtatcat atcctaggca aactccttgc ctaccaaaga ggtcggatac ttcaaacttt

237421 attataaaca gattgtcatt gtggatctat ggatttacga atatttttgg tcaccgtaca

237481 caacccctaa gtaacgtgat catatttcat aatctgctaa ccatttttct taatctattt

237541 cgtaggtgtg tatttagccg gcgggcggcg ggcggcgggc ggcgggcggc gggcggcggg

237601 cggcgcggcg tgtttgcggg tacgcgccga attatacgga ataataaata ttatgatgaa

237661 tacccgcact ttggacgctt atatactata ataattatta cataagccgg aagggtacta

237721 tgagagaaaa tgtataatat atatcctctt ttttttttaa acttcaggaa atgcatttag

237781 gcacctctac gccgggcgtg caatggcgta gagagtgtgg gactcgccgg ccgcagaagg

237841 tgaccgtaat acccactaaa acctgacggc cctcacacgt attacggcgg ggtcgtgggc

237901 actgtttaac gtccacgaca atgtatccta ttttaactta ctgttttggt aaatcttttt

237961 catattaggt actagctaag aacaacgtaa cttcgtctgc gcggaattaa aaaatatagc

238021 ctatgtgact aggggataat gtagctttct atgagcgaaa gaatttttga aatcggccca

238081 gtacttctta ggttatttca atacatacaa aaacaaaaat gcagaaatag aaactttctc

238141 tctttataat attagtatag atagtacaaa ttacaacaca aaagaatatg gtcagggtaa

238201 accgacaatt tgaaaatatc cacaaaccgc ttctatattt taacatttgc tgcacacgcc

238261 aaactgtctt cccataccta aattacaaaa gtcttacgtg catttcatta acaaagtaaa

238321 attagacctt atttccatat catcatggtt taattttaaa tgattttgtt gttagaaatg

238381 ttatatttat ttagaaaagt tgacacgcaa cggacgcccg cttaattcgt agcagacaag

238441 aaatgggcgc gcaacgaaac gcactaaaat aaaatgttta tttgaaaagg tgataactag

238501 atatttattt atctcctttt acatactgag ttatacgctt attgaatttc ttaaagattg

238561 acaagaagta gtagtagtag tagtagtagt agtaggagct acagacgctc agttttaaca

238621 gcacaattaa aactgcatgg cactacactg tttaaaaact ggacgtttgt agcctggttt

238681 acgcaaagct atacaaaact gcaatttttc tcttaaaata gcaaaccagg atagtattta

238741 ccaaacatgt ccgaccaaca gcatttaagt agagtagatt attaaatcct ttgttcggat

238801 acttgctagt tgctactcga ctaaaatact atcctcgagc gaacaaggct ttaaagatta

238861 atataacagt gagcttaccc aacatgcttt tcgatcgacg atgtcgctga tggaatcgac

238921 aaaaatgtat gaaactgaca ttgacattcg ataaatcatg tgattgtcac gtgcgagata

238981 tcgaacgtca atgtcagttt catacaaatt tgtcgattcc atcagcgaaa tctccgatcg

239041 aaaagcgtgt tgggtaagcc cacagatcag cgaagcattt tttattgata gtgtaatata

239101 agaagtcatg tagatatcaa gacaggacaa aaaagggtaa tccacaactt gttacaacta

239161 gacattagga aaatctactc tgagtctgac ccatgtccgc atgtaatttg ctacaggcat

239221 ttaagtgttt gaaaatgctc catgcttcat aaaattcaag actgttgtag cagaaaacat

239281 tgaaatttct attataaatt aattccttaa ttaacaatgt aattgccttt ttaataaatt

239341 atcatagacc tagctgcggt atatactaat tatgccttgc tttaggtcac atgaagttag

239401 gtctcgtgta aacaattctc ttgaatattt aataatcttt attaaatgga taggtcgtta

239461 taataatagc ttttgcttcg tttcgactta actttgttgc gattacgcaa gttaatatta

239521 ctttcgtgta accctacggc ttcattagac cgaattcgcg ataaaaacgt acttgtccgg

239581 aaggaaaatc tccatttaaa tcgcgaactc ggtctaatga agccgtaggg taatggaaaa

239641 aaatccgtgt tccgtccatc gagtgtattg cccgagttct aattttagtc cagtctttct

239701 acctttcttt tttgaatagg caaacgagca gcggatcgcc taagttagtt gatccgacgc

239761 ccatggacac cttcaacgtc agaggaaccg caaatgtgtc ccgcccttga ggaagcagta

239821 cgctctacct gttcttataa ggaaatgatg ggaagagaaa gtggacctgg caaggagacg

239881 acataggaag ccgattaata ggcaaattat gtgcaattgc aaatgtctat gtaaagcggt

239941 cgcttgacta tttcgcgaaa tctaggtggg cgcctttgcc aatttaaaat ataaaaaaac

240001 acgctatagt tagaaccaaa atggcggaaa ctatgtagct ataaggtacc tgtccatgta

240061 tagacgtcgg tgaggatccc tttataattg tctctgaaga catgttatta tggtagaagt

240121 cgacttgtcg caaagtaact accgaggttg tgtaatgagg gttgtctatg gtaatattta

240181 acttgtatac gtgactttgc ctgtatttta taactattaa caaatggtaa tcaatgtctt

240241 gaaaaatatt attgtatttt acgggactat caactatttg actgtttgag ctccgtaatc

240301 cattgattgc gcatgaaact gtgaaatatc taacgatacg atagtgaaaa ccatgaaagt

240361 ttatagttat gtaaagtatt atttgttgta gactaggtga ccctatagtg gtactcttat

240421 tttaaactga catccaaagg aaagaggtta agtcaatggt ttttttgcat gtgtaagtat

240481 tactctgcga tttatactat cattttgtaa tgttccgttc aactgtattt actttatttt

240541 ataagaacac gagcgacccg ccccggcttc gcacgggtgc aaatcggtat aatgctgata

240601 ctaaatactg gagcgtttgc aatgtaagcg ctaaaaatgt gttaatttac gacatcatac

240661 taaaaacctc taaatgatca gtgttactct gctatattgt gaatgtaata tacatgaaaa

240721 ccttccttat gaatctctct aatctattaa aaaaaccgca tcaaaatccg ttgcgtagtt

240781 ttaaagattc aagcatatat agggacaaac ataaaaaagc gttattgttt tacaatatgt

240841 aaggattctt atgataatac agacttgtta ttaaacataa tgaacgagtt caattattga

240901 tatttgattt tgactgatta attccatcca gctatcataa aggtcaatgt actgtgttag

240961 tttaacactt ggtcagaaca gaacaataag gcaggacagg acacccttct agatcgatcg

241021 gtgacttcgt atgctcgtat taaaaaaaat tatctatgtc tttctaataa tgaaagaacg

241081 tttcaaatca gttcaggggc tacccaaagt acttttctat tttattttct atttgcgatt

241141 tcgctgatgg aatcgataaa aatgtatgaa actggcattg agattcgata agtcatgtgc

241201 ttgtctcgtg tgagatatcg aacgtcattt tcagttacat acatttttga cgttcgctat

241261 agaatcgctt cgcagcgaat ttactttggg taaccacccg tttcggagcc tagttcaaac

241321 aaactaggac agaataagtc ctcgtgtgaa actatcatca ggcgttgatt gcctaatcaa

241381 taagcaactt aatgtcagtt taatatattc ataatgatgt ttgtatagcc accgctccgc

241441 gcgtgtgtca tttcgcaaca aatatctaaa tatcattaca ttattatgca aattgcccag

241501 gatttgtatc gttgattatg tgattataac gcagacgtca atttcaatta taacattaat

241561 cttggcggga gatttgacat ttagctgtca cttgtcggtg tatatggatg aaggcaatta

241621 ttctatattt gcttcatata tattgtggca aacggggaaa gagacaccgg gattttctga

241681 aatagcggag cgaccgtttt ccctagacat ttgcaattgc agccgacact tgatctacgg

241741 aagaggagac gcacagcaag aggataatac ctctttctat gcgtcccctc ccctgccaat

241801 ttcccttccc atcatttcct tataaggaaa tgatggaaag agaaggtgga ctaagattag

241861 gcgtccggca ccacactcgt cagacgaaac agggaatagc ttccatttga cgcctgtctt

241921 ctgtatggtc gtggtatttc accggacgag ccggacaatt cgtgcgaaga tgttaaagcg

241981 ggctctacca ctgttaaaat ttatgtgtat ttttttcata ataatgaaac gatgtaattt

242041 ccgacattct ttaggttatc atataaacaa acaaaagata ggctattagt acatccatta

242101 tattaataga aatacttatg tgaattgaaa taaaaggcag tctacgaggc tgatgaaagg

242161 ggatgaataa taaataatat gagacaggaa acatcaggag acagcgcgcg cattgttcaa

242221 gtaatattgc tacattagac aatggtggac agaataaata tttatttgtt aattatgatt

242281 gctggcgttc tatggtttgt tttttataaa cgagcaaata acaaagtatt cttatctgac

242341 caggaacgct gttgtcatgg ttgtttctgt tggaaaatat ttaaaatcta gacaagaaat

242401 gaatcttgtg gaaatataag atcaaaggac gctggtaaaa aattgtaatt caaccgggtc

242461 agacaagttt gtccgactat gaggtcatga aatagatttt caattggcca cagcaagtga

242521 ggtataaaat ttgtttataa gttctgcgag ctcggctcgt ttaaggcgaa tgctttaggg

242581 ctctgcgaaa ttatccaatt tgcttaagaa cagtaatttt tcatcgttcg gcagtccatc

242641 gccatttttt ggcgggaact gtcggttatt aatcaattcg aggttaagtt tgaaacgatt

242701 agttccttta tggtgataat tttaaaacat taatttattt attcgtgtca aaccgcttta

242761 ttccgaaacg agaatcagcg ttattactgt tgtactttct acgtttggta ctggaacatt

242821 caaaaaaatt attccaatat acagaacatc cttgtttctg gtacttctta agatacataa

242881 ttgtcctcca gtatcgtata atcgacgatt tattggtcct cgtttatcgc agaagtttat

242941 agtttcttgg ggataaagtt taaattattg tttttggctt ctacgttaat tagaatagta

243001 caatatattt ttataattat tttattataa ttaattgttg ctacctgaca tatcttgcct

243061 tcggttaaga ttactaacgc catctagtat gtattattgt acaacttctt aagaaaattt

243121 gtttctaatt tcctgcgttt actttgttct taacttttaa catctaactt tttatattta

243181 tattttttct tatcaattta aaatgataca aatgcaaata tttttccgta agttttaatt

243241 gctaccgtat acatggaact ttacttttaa acatatcaaa gtgtaaccag gctgcattta

243301 atgattaata aagtgtaact gcgccttaac ggtgagcatt ggtcgtaaag tgttgccaca

243361 acacggggac cacacacggc gcagcaccgc cagacaggaa taacccttcc gctctcacgt

243421 gtcggcaact ccaacacaac tgttgcaact aaaaatcttt acgttactga taggttattg

243481 aaacaataaa caaattgcag atttttctac aaagcttcca aactccaaaa tactaacttt

243541 cgttatctaa tgaataaatt aacccttaaa tgcatcttgt tgctaaatgt atacatgtgt

243601 ttaaagaaaa atatgcaaaa gacattatgc aagtttcggc agcaaatcta ttttttgttg

243661 acatttctat ttagcggtct actaagttta cagagttata agaagcgtct ccgtaattgt

243721 ttaatcattc caggccgcaa taaaaaacca tgcatttaag ggttaaatat ggaccaacta

243781 gcaaaaacga aaaacaacca agacacgcac cttctacgat taagcacttt gtttacaata

243841 caagtcctaa acttctatga ataaggacat gtttattgtc caggggggta acctaagtac

243901 atgcgctaca aagcgttagc gaacatcaaa ggtgtatgga actgacgatg acgttccata

243961 catttttgat gttcgctaac gcttaacgaa ttatcgaaat tcattgtcag tttcttacat

244021 ttttatcgat tccatcaacg atatcgccaa tctaaagaga cgttgggtag ccgcccaggg

244081 cgattagtca acgtggattt gtcaactatt tatttgttag tttttgtaga aagagaaagg

244141 tatagaagtg gtgttagcct atacggttga gggacaaaac atgccttaga ctacgtcata

244201 cgtatgtcgt ggtgcatcac tttacggctt tgaaccaata acatcatgtc tcaagtcttt

244261 gcctgggacg tatgaaaaat tagtttccca aaaaatacgc ttggagcgct acaatactat

244321 gtgatatgtc acatttgttt gggtcttggt cctggaccca aacaaacaaa atcgcatacg

244381 gagaaaggga ccacagatct atatcactgt ctttgtcacg ttacgtatat ttcgtataaa

244441 tgtgtcacag tcgctgtcag tttgacatat gtcatgtctg atctacttct tttaatctat

244501 gttagtttta cttatcgatt tgacaacacg taatattttg taaataactt attcaatcaa

244561 gttacaatat tatttacgat aaattatttg tactaaaagc gtattacatc tattcccgga

244621 gcaagctatt aattctccat taagctaagt tcgatagtgt taaataaaca caaatagata

244681 aacaataaac aatcgtggaa acaaatcgta cagcgtcggg cgtcgccgct acagtggcgc

244741 cctcgtacgc gcccgcgcaa gcactgattg acagttgaca gctatttagg cgggaacagg

244801 gctaccaaaa actataatgt atttttaatt acactttttt aaattttaat taacattatt

244861 gtttttaata aagcgctatt tgctcttacc cgatttgaca aagtaccaaa tgggtcataa

244921 atattccatg agaatttgga agattttata attatgttac aaattaccaa agatatatta

244981 tactgtagat ggaacactcg atacctaaaa gtgaggcatt ttctttgacc tccggaggtc

245041 caatgtaggg aaatctccct aagtgttaac ctctaaatta catttgccga cataaaaagt

245101 cgatcgagtg taacctcttt tctcaaattt aaattgtatt tcatagtatt tactaaagtt

245161 taatattata attaatagag acatttttaa aacgctaatt aaaaatttat aacaaccata

245221 aaacaaaaat ggtctttgtt aaaaattgtt tggatttaaa ttgttttaaa taaaataata

245281 ataatgaaaa acgatcatta acgttttaat gaatgtttta gtttattcta ctgtttattt

245341 taaaaatgta aaaaaatcac acaatttccc aaacatggaa atttttttca tgatactggc

245401 aagttttttt ttttttaata atttctttct tgtcacgctt tttaacgtca aattagtatg

245461 gagtgctacc tctatgtata atatctcttt gcaaattacg ttacaaatta aattctgtat

245521 ggctgtatat tatttctccg ttagagacag aaataaggag attacaactg aagattaact

245581 ttatatttta ataccacaag atgcatcaat gtagtaatta caaaggattg taaagagatg

245641 ggcaaactac aaatgtaact gtatgctaat cggtaaccct acgtgagttg agcgacagcg

245701 gccgtcggct cgttagcgcc gccatgttgg agttttttct gttgtttctc attgtcaatt

245761 gtctaggcaa tggcgcctcg cccgttctgc caccggtaat ctgtttgttt agattatttc

245821 ttttctattt agatcctcta ttaaaattaa tggcaatgtt taaattaatt gctattttat

245881 tttgttgttt acttataact actatacttt acccatgtca cgcataaaat ggccttacgc

245941 aaaagaaaaa tctccgtttt tatcgcgaac acggtctaat gaagcccaaa tacctagtat

246001 ggtacttata acgacttgtg tttatggtaa ttgttttttt ttattgcgta ttgaaattct

246061 ttattcgcat atggacatac tgatgcagta agtcaatttt atctgctaaa ggatgtttaa

246121 tgtatatgag atgtttatat tacatgtttt aatttcgtaa tgaaatgctc accacggggg

246181 aacaatagat caagcactcg caatcaccat aagtgcaagt gtcaagatgt ttttgtgctc

246241 ccccagacat gttcgtgtta acgaatgcag taggttaaca cttacgctaa tacaaagtag

246301 gttaacacct acatactaat gaactgttat tttaaatact aggtttacaa tttatcttgt

246361 tttaattggc ttttccataa tataaaccga caattcaccg gacccggtgg gcaaaagtga

246421 aactacgtta tatttgctca ctagcgccat tttatgcagt tttctatgtc catatcgtag

246481 gtccattgtt caactaagtt gcaatctata ctaatactat ctgttgttcg cgacttcgtc

246541 ggtatggaat taaaaaaaat gtagcctatg ttactcggga ataatgtggc tttctattgt

246601 tgtatgtatt gcaataactc tgtaacgatg ccgttaattt ctagtcatca ttgtggctta

246661 aacctccatg atggctagaa attataaaag aggtcaaggc actggctatc tttttctaat

246721 atttttaaaa ttcaaaatta ttattccatt tttcctggtt tttaactctt ttcggaacac

246781 taagagctaa gctactgaag ggtgaagact tgtatcttca gtatttatca tttaataatt

246841 attactaatg ttacttctat aatttacaaa attattatat aatgccattt ctgtaatgga

246901 agtttttcat tattattaaa taagtgtgaa aactacaaac agtgagaagt catttattca

246961 tatctgttcg gtacagatgc actacagttt gtaatttcgg acataattga attttggcgg

247021 gaacgggtac atgtaggtag atgctacgct acggttgagc gcggggctcg cggcttgtgc

247081 ggggagtgag gtcgtcggtc aaagcagtca cggtcagcgc acagtcgctg gaggttgcat

247141 tacggttcgc gactacggaa gcggtgcatt ttgacttttg gactgggaac atttttgacc

247201 tcactgactc ggacagaatt actaggcacc tgcagacggg aaacagtgca tcgagtgcac

247261 ttaccaaaag tgccacttta tccattttca gtttcagggt aggttgtgaa ctaggacccg

247321 aacctgtatt tgtagtgcac aatatccaaa tgatttattc ttctattctg cgacgtacct

247381 aagatgaact ccccccgtgt tgctctaccg gtgcatacac ttggatacct cgtcgcaact

247441 cgagaactac tcggccgatt tcaagaatct tttcaacatt agaaaggtaa ttatgcccga

247501 gtaacatagg ctatacctaa cacattgacg aaatcgcggc caatattaac ttaacacaat

247561 ttaaatccaa acaattttta acaaagacta tttttgtttt catgttgtta taaattttaa

247621 ttagtgtttt aaaaatgtct ctgtggcctt ttatatcggc taatgtaatg taaagggcaa

247681 cattgaggga aatctcccta caagtgaccg gaggtcaact aaaacgcctc atttttaggt

247741 ttcgaatgct ctaactatag cataatatat ctttgaacta aactaacaga ctttgtaaac

247801 atagtgttac tcgttaaaac tagctttttt actctgccga gcattaatgc gtttaacagt

247861 tccttttaaa tcgaaataaa ttataaaaca cgtttacacg gtgtttagat catttaagtg

247921 attactcgag ttaagttgcg aattctattt aagtttaagc aaataagaca tttatgttaa

247981 tagattaagc tatcaattgg tagttctagt tgattcactc atctatttac ataattataa

248041 tgtttttgcg tatcgttgaa agttagttag gttgtcgtga tattcgtttt ttttttaatt

248101 gattattcag taggtaattg gtttcatact tgaattgact cctttaatta caaagtaata

248161 tgtacccact tgtttgttgc ttttaattaa aaagcatttg aaccttcata aaattaaaac

248221 aataattaaa ttaacgaagc cgtcgaaaaa atgtgcaatg tttttcttat cgcgtctcga

248281 taaataaggc aattccgaac gtgaaatgta attgcacgcc ttaacattcc tcgtcctgtc

248341 tctgcctacg cttattattc gtaatttcaa tgcgctaaga catgacacat ataattatgc

248401 aacgtgtcaa aaatcgccca ataaaacacg tagccgcgcc ggcttaatgg ccgcaataca

248461 acttaattat accgctatta tagtaacaat ctgtaaaaaa atcaagtttt cgaacgcgcc

248521 cgttcaataa ttcatcaaaa tggcgtcccg tctatcaata taataattct gtcaaagttt

248581 aattgaaatt ccgtaccgcg gccgcgcggc tcaaagcggc gcgggcgcgg gcgcgggcgc

248641 gcagtattat tgatacatta cgaggccgcg aaagagccat agcggcaatt tacaactgaa

248701 ttacaaatct ctgcctggcc cactaaatac acaagccggt taaatcaatc gcgctcgacg

248761 ccgtctccct ctaacgcggc ggacgcgcgc gggagcgagc gcgaccaaag acgcgagcat

248821 ggcgctgccg aacgatgaaa agcgccagcg gtcgccgcct ccgagcacaa aggggaaggg

248881 aaaaaagctt gtttgttcgg gggcgtgtca ccgcggcccg agcgggacgc aggccgcagt

248941 cgctcggccg ccgccgccgc gagcgcagtt tcacgtcact gtcaaactca aattgacgtt

249001 tcaatctgac agccgccgcc gtccgccggc cgacctccgc ggacacaaac aaaacgaaat

249061 aacgacgaac aagttgacat ggaattaaga aatcatcggg gactcgcttc aagaataact

249121 cgataaatat ttatgccaca tccagagccc gcgtcgaccg cgccacacat tcaaataatc

249181 gtcgccggcg acgtcgatgc tcatgtttta atgaatttta atcgccgacc tgcatagtaa

249241 ttgaagagca ttgtgtcccg aagaagggcc ataactaatc aaacaaacaa cagatatcgt

249301 gcgaggagat ggctttcgag gatcgctgta gccccaacca ggcgacgagc ccgggtccgg

249361 tgtcagggcg cgtgccggcg ccgcacgcag agggtcccgt cggctgccgg ccgcccagcc

249421 agtacacctg caccaccata gacgcaaggt acgaccgcgg cactcccaac gttatgatca

249481 aggttcagcc gagctcgccg ccgcccagcc ccgacccgca ccacgagatg gagtaccagg

249541 gctactacag gccggagacg ccggatgtca agccggtcat aaatctggac gacaggttcg

249601 agccggacaa gcggcggccg cagccgacgg cgccgatcgc cttctctata agtaacattt

249661 tacacccgga attcggtctg agcgcgattc ggaagacaaa caagatagaa gggccgaagc

249721 tggcggggcc gaatcacagc atgctctaca agccgtacga tttgtcgaag cagaatgagt

249781 acaagaagta tagttttgat tacttgaagt ccaaagaggg cagcgacttc tcgagcgtgc

249841 cgccgctggg ggggctgcgg cagacggtgt ctcagatcgg ggaggaggtg aacaaggaga

249901 gggaggcgca gaaggcggcg gagcagaggc gtccggactc ggccagctcg ctggtgtcgt

249961 cggcgtcgag cggcgcgccc tctacctgca gcacggacgc cgccagccag gcagggggca

250021 acccctcgct ctggcccgcc tgggtctact gcactaggta cagcgaccga cctagttccg

250081 gtaagttttt tcatatagta ttttacttct attgtataat gagaatataa ctgttagttt

250141 acaattacta acttttatac gtttaacata actagtataa tagcacaatt ttgtaattca

250201 ggaacaccta ccaattacat taaattcaat tacaaaaata tgatttctaa caatgcaaaa

250261 aaacgtaaac ggcattgata ataaagaggt aaatatgacc tacaaacctc atttcgttat

250321 aaaacagtta atagttgcgt tataactgtt tataattaac gaaacgttat tattgtgtta

250381 aatcgtgcgt ttacactgca tcctggcaca cgcccttttt ttgtcttggc gtggccgtcg

250441 gggcattgca ccaagaaggt tatgtgattg ataccgttga attatagtta tagataagtt

250501 acagaaggta gggaactccg agactcaagt tgttaaaggt tcaattcaaa actggacaaa

250561 agtatttcta tctttttttt aaagagaatg gaaggaatgg tgctattttt tacaaccttc

250621 atggacctac gacatggact atttatttca aaaatatgaa cggtaattta aactaaaatc

250681 ttagtaacct acagccaatt ttatttattt attaactcat tgaaagaata gattaaatct

250741 aattagataa ctaataagaa ctttaaaacc aattggcatc taacctccaa aacataattt

250801 cgattcgatc gatcttgaat gatcgattcg attcaattta ccttatcatt ctaattttaa

250861 aactggccgt acagcaaaac aattaccacc gtttaaaaaa acgttcaaat gtcgtaataa

250921 aaaaacaaag agtgatgata aacagctgtg ttccgccgcg ggtgtggcgc aaccagccaa

250981 taccactagg cgttttaaaa cacgcgcatt ttgaaaacct ataaaaaaca taatttaaag

251041 tatattaact atgatgtatt tcttactttt gcattaatat gtgcggcgct tgaagtatta

251101 ttttttttac ttttcaacca ctgcctaaaa tggaattaaa ttaaacggtg aatttgtata

251161 atggcgaacg gggtggatta aaagtcactt tttattttac aaaaaactac atagtttata

251221 tctaatagat aattctataa aaatatagag ttattaacaa gcttttcaaa cctatgattt

251281 gtatgtttgt gtttaatgag tcatttgtgt ttaggcgttt taaaaaccgt gtgtttccaa

251341 agcccgcgtt tcaacgtcta gtgtaaaccc gatcatacgt tcgcgtgaat aggaaccaat

251401 taattgctcg gcgatttcga ttgtttaatg cggtattaag tacgattaat tgttacgtct

251461 gtgtatgata tagtgttatc cgtttcgtgt tttaactgaa aatgttagat gtgctcattt

251521 tttaccgagc attcagattt gatataaagc gctaatagtc tacctactta aatagatgcg

251581 aaagtctaaa tgcctgcttc tacgtaggac gaaaggtaca acaaagactg gaagaaaaca

251641 acgttttttt ttaattacgc gtggacggag tctttccaga aagctagtag attataaagg

251701 acggcacaaa aatataagtg gccgacaata accacgacaa cgaaatcata tcaaaaagca

251761 ttaatattca ttatttggga atttcgttct aatttaattc tttttgtgtt atttagaagt

251821 tagttgtaag aaactggaat gtttttttaa tctgcatagt gttgaaactt gacgattgaa

251881 gagttcaaaa ataagcttgg aattttaaac gttcgagaaa cagtttagca agtataatat

251941 gccgttgtca cctctaaaca gctgatgttt tgttattcaa atacgtgtta gtctttttga

252001 tgtttgcatc ccgggtgcat caataaatta ttaggcaaac ggtgtattat aaatctgtca

252061 cattaattta attttatctg tcatctgagg cacagactgt aaaggaacgc gcgcaaatgt

252121 ttgtttttta tgtttttttt ttaaatcgtt gcggctcgag cggccagcat tagttttatt

252181 tttttttatt tgaaatattt gtttaatata aacaccggta gctgttttat ggaaaagcaa

252241 cttcttataa tattgtgaaa aatattttta caaggatgtt tgtagtctca aaattattat

252301 ttcttatcta taaactctct tgttttatat gcctaatgtg tagattataa aatgtaagtt

252361 attcatcaca aatgtaatta cattaggttg gtaacattga atacatcaaa gttccaaggg

252421 ccatcaataa cgtttctttt cacctttatt actctgccct taattaattc aagtcaaatt

252481 agactgacta taaaatgcat cgtaaagtat tccttcgagc ttaagattgt atttttttta

252541 tttaatcaag ctagggccgc ctttgatatt tacaataaaa aaaaaacaaa gttctatggt

252601 cgcaaaaaat aatgcagcga cttcgaagta aagaataaat aaaatgtaat cattctcgga

252661 aagtgtacct atgtgttatc tttaaggttt ttaaaaacca tctgaaacgt tatttggaga

252721 ggccatgaag cctcatacaa tacaagcttg atctattttt tccgtgccaa tttaaaaaat

252781 attaaacatc tttaggtcca ccgacatttt taaaaatacg ttataacgat cgaacttcat

252841 atctttctta cgtaattgca tttcatcgct aaaattgtaa ttataagaaa aaaccttgca

252901 attttataag ttgtaaataa atcattggcc gaaaaacgac ttgtcgacgt cgataaaaac

252961 ttcgagatga cagatgtcaa tttgacacgg cgccatttta atatcgcgag taccgattta

253021 tcggaaattg ggtttcggtg attattctgc aatcaactat agaaaaaata ttgatgttat

253081 attcattttt aaatggtaga agcgcagtta ggtttattaa ctagctagtt ttgcaattaa

253141 caacctaatt aagttttaaa ttatgttttc agcgctgatt ccagttactg aatagtctta

253201 taatctagtg tttatggata ataattaagt aattattatt gctacgtcta aatgtatggg

253261 taataaaaaa acaggatatg cttgtcagta taacgataca aactggcatt gcaaaaccga

253321 taaaaataaa attcaataac caagtaatat cgagtttaat tttttcttca cgtcagcttg

253381 ttataaagga catgacagga aaatatagac aaaagcaaac ccggcggtag ggtgcagttt

253441 aaattcactt atcgtaaaca cttaggccct tactgcaatc attttataac gagtcgacct

253501 gtttattaat tctacacgcc gccgatgact aaaagaaaaa aaaacatttt tagtctgtgg

253561 cggtaaaaaa atcaattgac aacataatta cgtcccgcca tcattatttt tcgcgttgta

253621 caattttttt attaaaaaaa gcacgcctct tttgcacctt ataaatgtcc tcatgggaac

253681 cgaatttaat tgacgctagt gaaattaatt aacaactgat gtaataatcg aatactcaat

253741 tacaacggca ttaaggttca aaattgaatt tccctctgtt ttgagagtgt aaattataac

253801 ctagtcacaa ttcatcgaag aaacaggtga aataacgaaa gaatttagtc cctattattt

253861 tgtgcgatta tcgtgttttt ttcgcacggc agccatcttg ttcgtcgcaa tggcggaacg

253921 cccagtggca aggtttaaaa aggtacattt ggcgggttcg ttgacacggt tatcggctgg

253981 cccgttttgg cgttttgcca cttaattggc tctaggcgtg aaatattgca tcttatttgc

254041 ttaatggcta tgatggatgt cgcagcggcg tttcattatc acattgttga atttaatatc

254101 gtaatttctt ttaaattggt ttgtgtcatt tcattatttt ttaattggca attcgtattt

254161 tcgatttcat tgacgtttct ttcatttaat ttcggttaaa aataagtatg taagtttata

254221 ctactgttat tataacttct tctgacgtaa aagtatagta agtaagtagt gatagttaaa

254281 atgttcaatt ctagtaattt ttaagtctat attccagagg tgttcatcag gcataatcaa

254341 gccatcaaaa gtacatcaga aaaagaacag tttaaaagtc tttaccaaac atgtatttag

254401 atttggtttg taaatgtaca cttacgtgga caatttttac aagttttttc ttggttaaat

254461 tcagaagtta actattaaaa gtccggtttc cctcagctgt ataagtgcaa ttgccattga

254521 ttttaaatgt tgaaaatttt attatattta aagccgtaca accgcacgta gagaagttaa

254581 ttcataagta aagaaacccc taacgttgaa gtatgagaaa tattctaata ggttgctgta

254641 agcattaagt aactctgagt ttggcagtta gagaaatagt agtcttagtc aattaccaac

254701 gtgactgtac atgccgtgta ggcaggatgt tttcttttta attaccactt ctcgctaact

254761 gtgccaattg ccggtgcaaa gaaccagtga tgcgccattt aattgtcgtt aatcagtgtt

254821 tggaccaaac gcaaaaaaat atacatcgat ttttcgaatg cggatcgaac ggacgtttaa

254881 ggactgctag tgatgtgcgc ttgatttttt ttttagtatt ttttgtcgat aagaactatg

254941 ttttttatta tttaattgtt gttgacaatc gacatttaga aggttcaata acccctgtca

255001 ataatgagat caatttcacc taaattataa ttatggttaa gcaacctttt cctatttact

255061 aaaaacgcta agcgtcagtt tttactcgtg tacacaaaca aatctcagtg tatccaaaga

255121 gaataaaact agtgacagta ttttaataca tttcgacatt gctgctacac aatcacttgt

255181 acgaaacgtt gtgaaactgt aactcgatat gcaacaaaaa acaatagaag gaaataagtt

255241 ttcaatacag taaaagtgcg ggcgtgcacc ggaaacagta accgctggaa aagattcgag

255301 gtaacaacac aattactgta tcgaccacac ctcgccacgc cggtgttacg tgccgatttc

255361 ctactaatat tattgactca tatttaacct cattgatttg agaaatcatt gcgatataat

255421 ttaattaatc ggtatctatt ttcagattaa tctaacaaag atattttttg tagatacaag

255481 catcaaaaat tatctttgca gaagggttca gatctgatcg gtgcatcttg agtttataaa

255541 ttaaataatt atttaattta atagaaattt tatggttttt gcgtctgtaa ttacttacag

255601 acgcaaaaac cataaaattt ctattgagtt tatttatttg agtttattta agtcattgct

255661 tgttttaaat ctggtggtat ataaaatatg ttactatatc tttcagttag ccgcgcagct

255721 aatctttaca atgttggcaa cactgatcta acgacttttt gcaattgtgt caacacaacg

255781 taaattgttt agaaacacgt ccactctatg aaccatgcgt tatgcattag atacttctta

255841 cacttatatg ttataaataa aagagcgagt ttacattaac attttacctt ttgtagttta

255901 aaatgcataa acaataatga ttttatttac attctatcga tatgtcgatg aacatctcta

255961 gttgtttgtt aacctttgcg gttataattt cgtttttgta tagttccata aatagacatt

256021 gaatctaaat aatcgcttaa gttttatcca ctttctgtag atatctattg taatatatat

256081 aaatgtacta tttaataaaa tcaaaatcat gcgtcaaaag catgcaataa tgggtttttt

256141 attcttattt ttatagatat tggattaaga ttacaaaagt agtattaaat caattacttt

256201 ctctagatat tttgcggtaa tgccttacat ttctcaaacc gtcgtacaat aattaaatat

256261 aataattcca tataaaagta tgtttgtttc gaatgagatc tagtttaacg aaaggaaatt

256321 aatataatta ataattcatc gataagcggc acatcactaa tcgtagttac cgagaaacgg

256381 ccaccgtcca tcgcgctttt tgctcaaaag aattcgctaa taacttgatt tctactgttt

256441 aggtggcccc ttgctttttc aacgtttctt ttaacagata tgcttacgtt gtaagggggc

256501 aataaattgt cgtatttata gttccacatt gttcctgact gcagggcagg tattccggct

256561 cctgcacgca ctgatcatca gggttgacgg ctatctctaa tttcacatcg tccaaaaaac

256621 aggaaaatat tagacattgt tcatgtcaca tttgaaaagt atttttctag ttagattata

256681 gagattagag tgaaaaattc tttcaaacca aaatctgaat gacaaaaaaa actatttatt

256741 tttaaaagtg acacataata aatataatat cacataaata ttaaatgaga gatgaatctg

256801 tagtcttttg taagttatgt aatatgtaat aagaaataaa ataattaata tacgtgacaa

256861 ccctatacct aatgctaatc cactcatatt aaaaccctaa gaccataaaa gtaaagtctc

256921 tttttcaaca aaacgtatac cgtgccttat tggttttggt taatcactct tcagtcgata

256981 aacacgggag tgtttgtttt tttatcgata ttcgtttcaa caaacttttt gtcattttta

257041 ttacttggta catcactatt attgttttga tctcgtaata aaaataccag taaggagtta

257101 tacatattat ttgtataaaa aataaatatt ctacgcaatt actaacgtat ttttttgtat

257161 taaaagctta ataaatacga tacttctgag atgtcgtaaa ataaaacgac tataccactt

257221 ggattctctg aaatagcgaa gctaccgttg cccatagaca tcggctatta ctgaagtaac

257281 ttaatcgacc gaggagacgc aaagaaagag catatttacc ttcctaagcg tcacctgctt

257341 cacctaatcc actttatcac cctatcatct ctttataaaa aaggaagagt ggcaccacac

257401 tcctcaggcg aaacacagaa ttatctccat ttcacgccta tcttatgtgt ggtcgtagtc

257461 cacagagcga gccggcccat tcatagaacg tcaaacatgt atgaaactga cagtgttgtt

257521 cgattctcac tcgtgacagg cacatgaatt atcaaatgtc attgtctgtt ttctatattt

257581 ttatcgattt catcagcgac atcgccgatc gaaaactact ttgagtaggc ccctggctcc

257641 aaaagtcttt gtagtttggt tataaagaaa aatgttattg aaaaatctca atcttatggt

257701 acgtttttat taatattaaa ccacccatct ttacctatgt tttcttaaat tgtttcttat

257761 acaagtttta tactgctttc atccttgtaa tctttcaaca cgagtcccca cgaaatagaa

257821 aaaaaaatca atccctacaa aatgcttgca caattacctt acacacctca gcgaaatatc

257881 aaacaaagca atatacactt tataccttgt taataaaatt caaaatgctt tactacgata

257941 aaggactcct ttaataaatt gcttgcaaaa gaaaaatgac cgtatacacc gggaataaag

258001 ctcaaattgt attgaacgtt aacaaatgtt tgtattgagc gaagcaaacg cactgaatac

258061 gtaatgagac gtggatgtat gagggtaatg taataggacg cggcattctg atagaattaa

258121 aaaaggtttg agaaattgat ttacgtttaa tacacgatac acttaataaa atagtattat

258181 aagtcagttt atacaaataa atctatagaa acaaataaat ttataatgat actagcgacc

258241 cgccccggct tcgcacgggt gcaatgctga tactaaatac actacagaaa aattgttgta

258301 tataacagcc atactaatca taattgcacc cgttcgtaga tttaccatag cagcgccatc

258361 tattgattac ttactcaacc cagtcgaaag gtatcgacat ctgttagaat catttggagt

258421 taacagataa ttagcaataa attacaataa aattgcgact ataatttgag atttaaacta

258481 ttctatctct caagttggat cgaactgcac atggtttagg agtccattga ggacaaacat

258541 tgtgacacga gatttatata tattaagatt tgattgttat ggacagctaa aaaaaaggtg

258601 tcgacccatg atgggctcca ttttccgcaa ctccacgcac gcatatgggc acacatcggt

258661 ataatgctga tactaaatag tgcagcgttt gcaatgtaag cgcttaaaat gtgttaataa

258721 cctctaaatt atcagtcata ctctattcac aatatattga gaatgtatta tagatgaaaa

258781 ccttcccgca tcaaaatccg ttgcgtagtt ttaaagattc aagcatatat agggacaaac

258841 acaaaaaagt gctattgttt tataatatgt aaggataagg atacttgcat caaggaatat

258901 accgtatcca taaaaagcaa cgttcagttt cacgcatcgt cgcgtttgaa acaatcacgc

258961 ttgccgtagc tacacatgtc agaatgtttg aatagattac ttacgaatac tgtaccacca

259021 cgaccattat catttggagg tagacagaag cggatatgca atttatacag taagacgtcc

259081 gaagacattt tatgcactga gattgcttat ctatgtattt agtgtcttgt agtgacttat

259141 tttcctacat ttagttattc ctggaaacta aaaatataaa tacagtcgta taacttattt

259201 atcaaaacgc atacatcaaa ttcacacaca atcggcgaga gagcggcaat taaacgtccg

259261 ccatttttcg tttgcaattg ccggtccgac ggcaaaaggt aacaacctaa ttattaatta

259321 attaccacaa ttccagaaaa taattattct gtaacgacat tatttagtag ttttttgtaa

259381 cctttactac tttggtagca tcaggtagga taatgataac gactgctaat tttattgctt

259441 cgtgaatgtc ttctatgatc ttttgtttta cctttcagtt ctagtaacag ctgatctacg

259501 agaaacactc agaatcgatt aaattcttgg ttcttttaac ttcagccgta tttcttgtaa

259561 tgcttacaat actctgtgga attgttttgc cttatacgaa cttattggta agaagtttct

259621 ttgttcgctc gtcgtttgtt tttagaaact gttttaattg gctaattgct taatatcgtt

259681 ttgataatta acgataacta agttttccac ttcgatttcc actaaaatcg tactgtcggt

259741 gcggtttatt gcatttcgaa ttagttcgta tttactagtt gagtaactgt cctaattttt

259801 taacaacatc cgtatgttgt tggggttttt ttgttttaat tatgccttgt taatggaaat

259861 atgaaatatg agtagtttta ttaaatattt tgagatagga atcttttaga agtaacatat

259921 aaggcaacac gcaatactac gatttcattt agtttgcgtg cgcgctgcgt gcggtaatta

259981 ttatgtcaaa cgaaaatagt aattctaatg aaaattcagt cctacataat taattatact

260041 tttaaaaagt attccgttta aactatccct aaagccttgt tcgcaccagg atagtagtca

260101 ggatagtatc cgaaggattt actaatcagc tccacaaaaa tgtttaatta aaaaaatact

260161 tgctacttga ctaaaatatt agcctggtgc gttttacaac gtagtaaaag gctgaattaa

260221 tgtagtgata attttcttat aacaatgtaa ttgcagtttt ctctaactaa tattataaat

260281 gcaaaaatgc aaaatgactt gtaatctgca ggtcccgagt tcgaatccca ccatttacca

260341 atatgttttt ccattttaga atttcatatg gacatcttta cccgacgctc ttacggtgaa

260401 ggaaaaacat cgtgttgcaa cctgcacata tcggcaaaaa aattcaaaga tatgtgtgaa

260461 agtcacgcaa aatacgcgct actcgtggag taaaggaaaa tggagcactt catgggtcga

260521 actatactat acttacttgt ttaggacatc tctagtgtac gataattaga ttttgagtca

260581 atgtttcaac agtggaaaca actatgaggt gtgaaaatcg aatagtgtaa attgtatgca

260641 ataatgtact aagtacagta atgaaagagt acaattagta atacaccaca gagcagtgta

260701 aattgaatat tagaagtgca gtaggtaatt agcacattgt agcctttctt tactacttcg

260761 ttagcatgta atatattaat taacaagcaa acgcattacg cattacttac ttttgagacc

260821 gaaaaagtca gatgtaaaat agaattaaaa atcatcttca tcctgccctt gtagagggtc

260881 ggcacagtat gtgctactcc atacttccct atcgccgtca tttctaaatt tacccctttc

260941 tttaatataa tttaaaatat cttctacata attaataaaa caattcagac cccggcataa

261001 ttaaaccaat aatttaaaca acatcaacta tttttcccgc gcaaaccaat aattgttgtt

261061 tcggcattta gcgcacttta cacagagccc gctggctgcc attacgttta acgctgccaa

261121 ttacaaaagt aatgaatatg tcccgacgct cgctatgaat aaattatcat cctcatttgt

261181 ccggataacg tccgctaggg ttgcgcagga atgttaatag ctcaaagttg ggctgttagc

261241 cgcgaattat gtccgcctgg tacttcacgt ctctgtttct actggtttgt ttggcgaagt

261301 ttttaaaaat atagttatat ttacaagttg aatattaagc cggctacaga cggccagttt

261361 ttataattaa aacagggcag ttctatcata ttctaactgt acagttctca attttgccta

261421 cacacgtcag ttttaactgc gcaatacaac ggtacaggta tacctgaaaa tctgtagcca

261481 gctttacgct agattaaaga ggcttttaga ttaattttaa taagacttaa accattcatt

261541 aaagatatgt taaattaaaa gagtttgatg actaagagcc atttgtctcg tttccatttt

261601 gaaaacttat tgcagatgac tttgtgcagt cacttcacta tttttgttct acaaggtgac

261661 agttagccgg tcattttaaa aggaaaatga atattttatt agcgaaaata aatcatctgt

261721 gaggggtaat tcatttcggt caattaatga gtgcatactt ggtcgctaga tctcattaat

261781 taaaattcag aacacataaa agaatgtgtg cgcggcgata ttttccagtg ccagcctaac

261841 ctaacacatt acggctcgtt acgaaaaatt ctctggcaaa cgttttcgag ggactttgtt

261901 taattaaaaa cacgaataaa aaacttactg cccgttttat cgggtgataa acgagcgttt

261961 gaagcgatat gttttcggtc aaacgaaaag aaaccttgct ggaataactt cagggtttgt

262021 ttttcaacgc aataaagtat cgtataacca aacatttttc aaaatctaaa agatttgtgt

262081 ttacaccatt tcctgttcta ttcttaaaac gataaaactc aatgttcgtt atgaacgtga

262141 tgcttgcatt ttacgatgca caagcgattt gataccgtaa tacgaattac gtaagatcta

262201 aaataactga aagcgtttct ttaaaaaaac gttgacgtga ataaaggatt aagtcgcgta

262261 agtgccgttt atcaaatgtg cgtcttatgc taatgtggta aggtgtgaaa tcctttgcga

262321 taaagcatcg aggaataaat tcagtagtac aggaaggtag agaagtgttt atagttgcta

262381 ttttcctctg ctgatagcat ttagcgttgt gtggatttat tgccgcgtac agtcgcacgc

262441 taaactgtct agatcactat aaagtgatta cgggtgattt aaaatggtac agaataaaat

262501 gtacaacatt gttgagattt gaaaacaata tggttttttg aaataataat catgtatctt

262561 gagaagtgaa ttgttaggta ctacctacgg taatattaac agtttagctt tggtaatttt

262621 taactaggta gctaatgaaa aaataatagg taaataaaca gaatcatttg gctgctagta

262681 gtagttcgac ctatgatggg ctccattttc gcaactccac gacaagcgcg tattttgcgc

262741 gacttgacac atatatttga atctgctccg atggtttcct tcaccgtaag tgtgtcggtt

262801 aaatatgtac atatgaaaca cgaaaatcat tggtatggcg ggattcgaaa tcaggacctt

262861 cagtttacaa ggcaagagct aaaccgctga gccacctacg ctcttcattt gacagttacg

262921 aatactaatt cggcacatag taaaaataca gattcacata gcaataaaaa aataagaaaa

262981 ttgtttaaat taaattaaat tattaatatt gagggtagtc gacgcatctg caattgtaga

263041 tgtctatggg cagcggtcgc tttgctattt cggcgaattc aggtggccgc ttgctcgttt

263101 gccaatttgt aatataaaaa aaaactacaa aaaatacaag atttttataa cttaattaca

263161 ttaaataatt aagcacaaac ttccatgctt tagaagtaat tacgtcactt ctatacacgc

263221 aatatggcgg cagaacaagg gttatgtata ttactctatt atactcctgg ctgggtctaa

263281 ttaggtctat gggcgtgccc acgcatgctt actggtgtga tatttaatgt accccttatt

263341 aaatttatgc aaagcaaacg taaaatgaat ctttttacca ttattaaaag tctcaattgg

263401 attaacacag ttttttagtt ttcctatttt ttacaataac ctttttcagt gttaattgcc

263461 attttaattt atttatacag tcaaacttgg ataagcaaga tctggataag cgagaattat

263521 ctaaaagcta gtcatacaat gatcccgaca agctacctca attagcgaga aaattgaatt

263581 ggaacttgga aatagcgatc ttaaaatagt agctattgtg ttgtgaatat tttaaattaa

263641 acttatattt aaaaaaaatc attaataatc attttttaaa tcttgtaaaa cccttacgat

263701 acttttgcaa ttaaatgttt gtttcatttt tttataatat aaagtggcat aagagcaatg

263761 gacatctaga ttctgtaata gcgcagtgac cgctgcccat agacatctgt aattgaagat

263821 gcgtttaccc ttaatccacg gaggaggaaa cagcaagagg ttaattcctc ttcctatacg

263881 tcccctcccc cgaattgccc ttcccctcct acgtttcatg cttacaaccc ataaaattaa

263941 agattatcgt tcaaaatatt caactataat aaattaatct atcatttagt aataaatgac

264001 gttattacgt tataaatata catttattat tattcatgcg gtagttataa tgtgattcca

264061 tagcttagat aagcttatcg atccaatctt ttggagtgta tttggagtaa aaaaataaaa

264121 atatatttat caatgactat tttttcttta cctaagtaat aatattgttt atatataaat

264181 atagtctatt tattatattt taaagtaaaa tagcatgatt agaaataata acttttttta

264241 taattgaagt tacattgacg tgctcgactg tacttaccag tagccgtaaa taattactca

264301 attttaaatt ataagttaaa acacctttaa atacatcaac aaaaatctac attttcacaa

264361 gggcgctttt tcaacgcgcg ctaaataaac tttgaaatag aacaaataca tttccatgta

264421 cagtctaacc tagataagcg agaaacctct ataaacgaga gtcattgtct ggtccgaaac

264481 aagagtcatt ttacagcgat cgtattagcg agggcgttta aaaaacgcca acaccgacgt

264541 tagaaaagcg tccgcgtgaa gcaactgtct ccaattggct tactcgccac ttagaaacaa

264601 atttgctttg ttggtctcag aaaacacgat aaagttaagt ttcctcttca ctagcggttt

264661 tatctttcaa ttactgaaca ttgttttctt tgtccttagg tcctagaagt agaagaatga

264721 aaaagcagat gaattcagaa gagaagagac cgagaaccgc cttcagtgcg tctcaactag

264781 caaggctaaa ggtataaata gtttgcttta acaactttaa ttagagcgac agtggctcag

264841 tggcagtaat aagagccttg ttgatttaat aaaaaaaatc ttttgaattc aatcaataac

264901 tattactgaa gtatgcggga tcctgcggga tccagctgga ctgaatttaa tccaataaca

264961 atcgtgcggg aacgcaaaaa cttgaaaaat tgtcgcttaa tttattaaca actagctatt

265021 acttcgtcag cgctaaatta aaaaaatata acttatttta ctcggtgata aagtagcttt

265081 ctaacggtga aagaattttt gaaatcggcc aagtaatttt tgagttattt taagacatac

265141 aaaaatacta atctttataa tattagcata gataattcct agtctctttt cataaatttc

265201 tttactaaat aactgtttat tgtttaatca caaaacatct gccgtttata atgttcctaa

265261 atgactaatg aagttctttt gttcttagca cgagttcgtc gagaaccgtt acctgacgga

265321 gcgacggcga caggcgctgg ctgcggagct gggcctggcg gaggctcaga tcaagatctg

265381 gttccagaac aagcgagcaa agataaagaa ggcctcgggt cagaggaacc cgctcgcgct

265441 gcagctcatg gctcaagggt tatacaacca tagcaccgct actgagagtg atgaagatga

265501 tgaagaaata agtgtcactt aattagtgat ctgaactata tctgtacatg ataagacgca

265561 aatccgcgat attagtgtct aagcgacttg cggaatatgg acgtaatcaa cgcgatgttg

265621 atacaatgta ttgcgtaaac catttacggt cgaatactga aacaaatatt aaagttaaca

265681 cttaaaaaca gacgttatgt atgtaatttg taatttaaag gaagacacac tgcaccagtg

265741 gtagagcgcg ctttaacatt atcgcaccaa ttgtccggct cgcccgctga cataccaaga

265801 ctacacagaa gacacgcggc aaatggaagc tattccgtgt atcagtctga ctggtgtggt

265861 gccagaggcc taattttagt ccactttccc ttcctatcct tttcttataa ggaaatgatc

265921 ggaaggggaa gtggatttgg cagggaaggg gacgcctagg ggggaattat cctctttctg

265981 tgtctcctcc tccgtatatt tatggtaggc aacgcatctg cagttgccgt ctgtgtgtcc

266041 atacaaaata tattttgaaa gtgctctgtt gttcgggcac gttgatatat tttgtattga

266101 tacacagacg acacgggccc gatcgggaca tggtgtaatg tgagccattt ttaagcgtgg

266161 tcttataatt taagatatgt tcaatattga aatgaagttt cagtattcgg tcattagata

266221 aacttgatct ctatttcata caatttaact atccccttct tgccaccatt tgacgtatgt

266281 tccttttgtt aaataaatta attttaatta tatctattaa tttacttcaa attatagttt

266341 aaagttaatc taaagtaaaa aggacgctgc tatttatttt acatctcata tttcattaaa

266401 aacataatct acaagtttag tctattaaaa aaatgtgcta aaaatcatcc tttttattct

266461 accctacggt ttcataagac tgagtcttcc ataaaaattt acgccgaagc aaaatgtccg

266521 tttatatttt gaactcggtc taatggagcc gtaggtttaa acaaaaacat tactatgtac

266581 agccaaatct ggattagcaa gaaacctcta taagagcgac aaatatcgcg gtcccttaca

266641 ctctcactta tcgaggttcg actctattaa tacaagttat taggcattgg aaactattgt

266701 ataagaatat tgttaaagaa ccgaaacata ataaaatgtt tcaatggaat tgcacattct

266761 gtcacaagca gagaggagaa aataatagcg tctttagaca aattgtagaa aaaaaaacaa

266821 taaaaaaatc tttcaaacac attattccaa aaaggtgcga tatagtaacc aagtagttaa

266881 taagtacgta gattaaataa taattatttt agtttgtaat aacatttgta aatattaatt

266941 tttattttat ttaaaaatca tttttctaat tttttgttaa tttattcgaa actagccctt

267001 gtgcgcgact tcgtccgcgt agagttctat aatatagccc atgttactcg gggataatgt

267061 agctttctaa tggtgaaaga atttttgata tcggtccagt agttcttgag ttatttcaat

267121 acatacaaaa atattaagtt tctctcttta taatattatg tttattcgaa ataaatctca

267181 aaaacaatgt ttggacgaat gagatattta ataacatttt tttggaatta ttaatgtcaa

267241 ttttcataat tacgaaaatt aaatttcaaa gcttttcttg tagtctgatt aaaaaactaa

267301 cttaaaactt tgtaggttgc tttaaaacaa taacagtgga ataaatttaa aaagtgtttt

267361 ttccacaaaa cctttataaa taaaaataaa ttctatgtca tatttatgtg cgagtaaaag

267421 aattgtaatt cttacacttg agttggcacg tggtgcggat aagttcacta gtcccaacgt

267481 gccaactcaa attagctcta taatttcatg tttcgtgtat ttttttggct ccagtcatta

267541 tcttcttcca tttataataa gttatatgta aatatttcaa attttatttc atagttattt

267601 ttgactgcgt tagttgtaaa tgtaaattaa aaataaatta tattatctat ggtttaatat

267661 tgtctttaat ttattaccaa cacaaaatac aaaaacataa aagtaaagac gtatttgtat

267721 ttctcttttt tactttagtt gtagtctggt ctagattcta ataaatattg ggctaaatat

267781 tcatctaaag taagaaatag aaaacttacg aatcttaaag taaagccttg tagtcaagta

267841 gctagcaatt taataactaa acgtattcga acagccacat ttaagtagag tagattataa

267901 atcctttatt cggatacttg ttacttgact atcctggtgg aatcaactta ttagagattc

267961 tcaaagagct agatatcctt gtgctataag cgtaaatcac ttaagacacc atcaccatca

268021 ccatcatcaa catcatccct acagagggtc gtcaccgtat gtactccata tttccctgtc

268081 atttcttaat tcactttttc gccatatctt ctttcacaca atccatgcat gccttctttg

268141 gccttcatca tttgtatccg tccacattga atttattttt ttattcattt aagacaccat

268201 cttcatttga ggctctgcag cggcggatgg gcctgggcct ggtccagtaa atttctccaa

268261 tttgctctac tttgggcctt ttgcttccct tccacctttg catccgtcca cattgaattt

268321 aattttttat tcatttaaga caccatcttc atttgaggct ctgcagcggc ggatgggcct

268381 gggcctggtc cagtaaattt ctccaatttg ctctactttg agccttttgc ttcccttcca

268441 cctttgtatc cgtccacatt caatttaatt tttaattcat ttaaaacatc atcatcatcg

268501 gaggcactgc agccacggat gtatctgggc ctggtccagt aacatttcta caatttgctc

268561 tactttgggc cttttgctac cagcccgccg ccgatattaa aatcacgtta aacgtcatct

268621 agccatctta gctttggcct accacgtgct ctacgctcag ccggcttact ctccaacaga

268681 cgtttgggaa ctatccatgc gtgcgacatg tcatgctagc gtagccgtcc aatttttcat

268741 ttaatatgct attacataat tgaatgacgt atttaatatt cgatagtaac ctaaaaatag

268801 cacaaaatac gtagaactac aacaaagacc tttcaattta aaaaatatgt aacattttcg

268861 cgctgtttta cgacaaacaa tttcgttgaa ttccctctaa ttatgttcct ttgctatttc

268921 ttattccagc tgtacctggg accatttgtt tcaaggaatc ttatggtata cgtgttaccg

268981 tcagaatgta aactctgtaa gctttgctct cgtctagaca taggcaaatg tatgtacaaa

269041 ttgttcagct tcagttaaca ctcgttagtt tgtaatctta cgaagacaaa tcgtcaatta

269101 gtaacctcat atataaccta tgttatatat taaaaagcac caatagactc gtccaagagt

269161 taacatattt gacatatggc cgaatgttca aataccactc aaaaatcgtc gtttaaaatg

269221 taattgtcgt caaaattaaa cgagatcatc gtagtagttg gagtttaaac gtggacttaa

269281 cacgtaatat cctcttaaat atttgagtgg catttgaaaa ttcggccgtt aatttaacaa

269341 gctatttaaa atgatctcta ctaaggcact ggttaattag gtgaccagta tgacttcata

269401 cataccaaat tcacaatttg tcggtagcat acggttacgg cgaatgcaaa aaagcttact

269461 agtaggtcgg gcaaagtgtc taggaatgtt cccaggcgtt tccagtattc agcgccgttg

269521 ttaattagtt tcttagtact tgtacacgca agtgttaatt gcacatacat aaagaaatat

269581 atctaaaata taaatagagc attttaatag aatttaacta ttaaattgtt aaaactttcg

269641 tgggacatac ttaacgtgac cagacatcag gtcttgaacg ggactgtccc gttttacagt

269701 catgtgtccc ggtgtacaag gaataatctc agggacgcca aattgtcccg ttttcggaaa

269761 ccgagccggc cggacccggc gtggcggtcc ggccgccgca ccggggctga acgcgggcgg

269821 tgttcgagag cgcattgtcg gtcgacgcga caacaatagc cgggagcacg atagtggcga

269881 aaacgttctt cctttttgtg tactagtttt ttctttcgcc ctgtttacga cattttagag

269941 atgggcaaaa tacgcgatta caccgcctcc cccccctaca tctgtttaag ttctacatct

270001 gtgcagaact tcagccgttc ttgctttact atccttcaaa caaacacttc atttatgtaa

270061 attgtagtaa tcacaatcag cctgaactag taggaggggg gcgagtcgag tccgtgcccc

270121 tggcgtcgag ttcagaggct ggcaaagttc acattttgta gagaaatgga taactcataa

270181 tttacctaac tttcacaata gaatatcaga tacgatacgt cagacatcag ttgaaattat

270241 aaggcatttt tacatttttg ccccctttaa acagtttgta catccgggac agttaatcca

270301 tactaatact agctgttgtg cgcgacttcg tccgcatgga attaaataat gtagcctatg

270361 ttactcaggg ataatatagc tttctaatag tgaaagaatt tttgaaatcg gcccagaagt

270421 tcttgagtta tttcaataca aacaaaaata caaagtttct ctctttataa tattagtata

270481 gattaaaaag agacaaactt tgtatgtatt gaaataactc aagaactact gggctgattt

270541 caaaaattct tccagcgtta gaaagctaca ttatccccaa gtaacatagg ttattttatt

270601 taattccatg cggacgaagt cgcgaacaac agctagtaat gtatcacagt tcacaagtct

270661 atgatatctc gggctcatta gtacgaatta agcgcgctaa agatgcataa atcaagccgc

270721 gccatctcgc gtatgattta gtgactacat tttagaccta ccgggaacca tgctgagatt

270781 caactaattg ttctgctgaa ctcactcata tgaagctttt gatctaattt tgacgactag

270841 atattttaga ataaggttga cattctttga attacctaaa tatcgagaca tcggttattg

270901 ggttcggata tcaatcgaag tactttaagt gccaatagcc taatggtttg gggtacggac

270961 tatcgaacta gcggtcgcga gttcgattcc caaaattcga ctattgtcat cggccatatt

271021 agtttatagc ttgttagtac aagctttttc atgctgactt ggaatggtta aaggaaatat

271081 taataaatta acaataataa ttgtattgta gagaaactgt atttttgtat gtattaaaat

271141 aactcaagaa ctactgggcc gatttcataa attctttcac cattagaaag ctgcattatt

271201 cccgaataac atattattta tcccgaataa cagctagtac ttttataaaa aaaaaacaat

271261 tggataaatg gatacctcta aaatgtctta acggatccca atgcacagat gtaaaaaaca

271321 gcctggaaga acacttgcat tactaagttt ttttttaatt gtacgcggac gtagtcacag

271381 cagctagtga cattgttgct atcttaactg tgtagtctgc atactgtcac aagtattgat

271441 gggcagacgg cacgagtttt tatccgaaac ggataaaaac tcgtatgatc aggaaaacaa

271501 agtatcgaaa cgttttactc caatttcccc agtattgcta aggtcaattc ttttttccct

271561 ttttgccatc agatcctggt agacctatac atacgcggaa gtacctaata attttgcatt

271621 cataagttgt tttcatcatt aaaaaaatat taacaaagca tgttagatat aattttattt

271681 ttgttattct tttattccaa tctcgtttat tcataaaaaa acatgtttat gttaaattac

271741 atttatgaaa tgacagatag tttttatcca cgtatttggt aaaaatcacg acgaaaagat

271801 agaaattatt agtattcatc cattttgatt ttttatccgg atgaaatccc aacactagtc

271861 acaagataaa tacttgacat aacctaaaaa ctgcaaaaaa caaaagacgc tcagtccgaa

271921 tgacagacaa ttatgatcac gtttgatgta caaccgacat agccacaaaa caacaacaaa

271981 atattccaaa cgggcaaatt cacaatgaac ttaattaatt ttactgcaaa gaaaggcgag

272041 gggagcgcgg aatgttgtcc tctatacggt ttcgacttaa aaatgacata aaaatactta

272101 catggacaaa tatgacaatt gtttgtaagc ttattaccga aattgcgata aatgtcacat

272161 aacaaaacat aattgacacg aggaaatagc gggaactttc ttattttgaa agttaaaaaa

272221 ttaacaagcc cctttgtacg caaggcaacg cgaatctgac attttagtct ctaaacctat

272281 ggcttcatta gacagagttc gctatcgcgc gctttttatc aaaacgaaga ttttgctacg

272341 gcgtaaggtc gttttttgcg tgacttcggt ctaataaagc cgtagtgttt gtcttttatg

272401 ttacgatctc atagaaacaa gcatatgtaa agcccttttc gcatcggata gtattttagt

272461 cgagtagcaa gtatccgaac aaagtctact gtactaaaat gtacttctta ggacatgatt

272521 tgttactaaa ttacttgcta ctcgaataaa ttactattct agtacaagct tagacaaata

272581 cctatacgta tataaatcat actgctctat cagccgtcat atttaaattt attccttttt

272641 ttgcaataat cgaatcgatc aagtcaatag aggaaaataa ccctctacta tttctaacct

272701 atctatggca taaatgattt ttttttcata attttgctag gaaaaaaata attactcatt

272761 ccaacaatac aagttgttta tctagctcga ccatttatgg acccaatttg actttcattc

272821 taattcaatt gtatttttaa ccttttcttg tgtaaatttt ccttgaattt caccgcgttt

272881 gtaacaaaaa gtgtgcgttt ctgcaatgat ggtcactgaa tagtcattcg attgttattg

272941 cacacataat tcatttaaaa gtcaaactaa actgtaaaaa tgttaattca aaatacggat

273001 aatgaaaaaa atcgtgatgc aacgtgcaca tatcggtgaa gaacttcaaa gatatgtgta

273061 aagtcaccca aaatacgtgc taggcgtgga gttgtggaaa atggagccca tcatgggttg

273121 aatcttacct taataaaaaa tagtttcaag atcgtatatt ggtatcggct gtgtaaaaat

273181 aaccaaacca aaacttcaaa gccaagtaaa agcgccattt tgaaattttt tcttacatag

273241 atcgcttaca taggcttcaa tagaccaagt tcgcgataaa tatggatttt ttccatcggc

273301 gtaaggctgt ttgcgtgaca tcggtctaat gaagccctag agttagaaac agcgtgaacc

273361 atacaacttc taagtaccag ggtgacataa caccgactgc atgatcataa ctcacaagct

273421 atgacatgta aaattggtgg ccaattagtg gcacgagcga tacgtctccg caagcgacgc

273481 aaaacgtccg ccgacaaatg taccgtgtta gtttttaact gtttattcta taaatagttt

273541 actaattagt tacagccgta aggataattt gtatgattat ttatatcatc ctagtgactc

273601 tgggattagt gtttttacat ggcaagatgg caaacgagca acgataattt aacaaacgcc

273661 caaagtatgt acagcaaaac caggataagc gacaaacctc tataagcgag tcattctatg

273721 atcccgacga gctacctccg taagcgagaa actcggatta gagaaaaagc tctttaagcg

273781 agtcaaatat cgcggtccct ttggctctcg cttatccagg ttcgaaagta attagtaata

273841 tgagtgtggt catatcattt aagacctaaa tcagtgagct actggtttaa ctgtttaaac

273901 ttacgtttta ataggcatgc cgagaataga tcagtagcca ccactcagta tggataggag

273961 cagataccta aaattggggc agctgaacct tggtatgcga gaattagcaa ctgaggaact

274021 accgataata tcacgtgaac tcggtctcga tgcagtctta gtccaagaac aatttcctcg

274081 aaccgaccga atcgtgcaaa cgtgcaacca gtcgggtgca ggtatctttc ttgccaacaa

274141 ttccacaagt ttctgcgtac ttcaccaact ttgtaacgac tactgtgtgg tgggtcactt

274201 cgctgaattt aatatctttg tggtgtcgga catttacggc atttagatgt cgtgattaac

274261 aacttaaagg gaaaaaaatt tacttggact cgattctaat gcacattctc cactttggta

274321 ttgtgactga aggcaatatg tagggcgagg tccagataca gaacaccgtc gtaatcagat

274381 ggaagcattc ataatagcta gaggtctaca tataataaac gcacaaggtc agcccgcaac

274441 attcgctggt gaaagagggg aatccaacgt agacttaact ctggcatatg gtggtgtcaa

274501 cgttgaaaag tggaaagttc acgactgcgc tagttccagc gaccacagac tcataaccta

274561 cgagatccat actaacagac aacagcctga tactgagcca gctagggatc ctccgcgctt

274621 tcgggactgg gatgtggact gggtgcgttt ccgtgggtta atccaattaa gattgggaag

274681 gatgaatgta aaccggccag catcggaaat agcagaggat ttttccaacg tcattaccca

274741 cacagcgaag aatgtcctag gagaagtgcg ccctaggaac gatcgcggtc atgaatggtg

274801 gacgcctgaa ctggataagc tgcagaaaga atattgcaaa acctgaaggg actggcaaag

274861 gaagagaaga atgggaggta aaatggaaga agcggctaga gaggtatacc actgtgtgag

274921 agtgagatat acaaggttaa tggtggagac acagctcgcc ttcttccgta aacgcgctga

274981 gtctgcaaac caagatcctt gggtgcaagc ttatcgattc attaaaggta aaggccggac

275041 ccacagtaat gttattaacg ctaccgccta ctcgcggggc cactcggaaa cagtggagga

275101 ggctatgcgc cacttaataa cttctttatg tccagacaat gacgttagta aagacacgga

275161 atatcaccgg ttggtgcgga tggtctccac ctttgtgccg tctgggcggg atgtgtctcc

275221 accatccgtt gaggaattgg gagatatagt caaggctctg cctaatacgg cttcgggtct

275281 tgatgggata agctctagaa tcgtgaagaa tgtgtggcgt gcggcaccaa aggaattcca

275341 ctttgtgttt gctaaatgta cgaaagaggg tgtgtttccc aggatttgga aggacggtag

275401 gttgattgta gtaagtaagg gtaacaacaa accgccaacg gatcccaaag cgtatcgccc

275461 tataacgctc ctcccaattt taggaaagat tttggaaagg gtactactta gatgtgttcc

275521 ttctatctcg cgtgggatca caatgtacca gcacgggttt ttttcaggtc ggtccactgt

275581 tacagcgctg cgatcaatgc tcagcaccgc gaggataact caatcaacct atgttcaggc

275641 tattttcttg aatatctctg gtgcctttga caacgcttgg tggccgatga ttctagttaa

275701 ggcaaaagga ggtggatgtc cgccaaatat tttccgaatt atcgccgatt atttcacctg

275761 tcgcagggtt gggctttttg taggggacca gctcgtatgg aagacttcca ctatgggctg

275821 tccacaaggt tcggtgttgg gccctacact atggaacctt ctcatgaatg atcttcttct

275881 gctcccactt cctgaaggtg tgactatggt ggcgtatgcg gatgacatat caattctgat

275941 cgaagctaat tccagagcta gaatcgaatc caaggacaaa gctacgctga ctcttgtccg

276001 ggactggggt gaacggaacc gcttggactt ctccccacca aagtcaacaa cgatgacgat

276061 aaaagggaag ttccagcgac caccggtcat ccgttttaac ggtgtttcga tcagaaacgt

276121 aaggtcagca aagttcttag gggttgttat ggacgctagc ctttcattca ctcagcacgc

276181 gtcagaaata ggcgaaaaag cgacgaaagg ttttgggaaa ctgtcgcaca tctcgacttc

276241 atcttgggga atccgttacc ctagcttaaa acttttgtat aaagttatct atgtccccat

276301 tctaacttac gccgcagaat gctggtttga acgagcaaag atgttcgcag tgcgcagcgc

276361 tctcctaaga tcacagcgaa attcgcttgt cctgatcacc aaagcgtata ggtcgaccag

276421 cacggctgcg ttgtcggtgc tggctggtgt tctccccgcg gacctggagg tcaaccgagc

276481 tggtttagtg agcagggcac atatttcatt tataaatatt gttattagtg aatttaacgt

276541 aacgttaaac gtaaaaagat gcataattaa catccgatgt tggctcgaaa cttgttggtg

276601 ctgacaccgg acacgtttag ttactaaatt acttcctacc ctatcctgtt gcaaactaga

276661 ctttaattac gtgagtgcag tcgtttttat tcactaataa gctactagtt ttagctacat

276721 tgtaagtaat cgatattatt ttaagtttca caaaagtctg tcttccagga aacgcgtact

276781 caaaaaattg aagaccacaa aaccgcaccg acatcggata tttccttaac gtaacaaata

276841 ctcataaaaa aaaacaaaaa aattaagtac attcgcatga acaggttcgc ataaattgta

276901 ttttgcgatt gttttatcca aataaacgtt tcaatgataa ttacttagct ttttttcttg

276961 attaatttaa ctattctcta ttttcccaat actgttagtc tttggcatat ggctcatttt

277021 catccaagac aaaataagag taataacagt ctttcataat tggcagcctt atttttttag

277081 gttattatgt caagattgtc agataaaatg aaattatata aaataagcgt aaccaatcta

277141 tatctatact aatattataa agagaaaaac tttgtatttt tgaattttgt atgtattgaa

277201 ataactcaag aactactgga ccgaattcaa aaaatctttc accattagaa agctacatta

277261 tccccgagta aagccttttt cgcaccagga tagtatttta gtcgagtagc aagtagcatt

277321 tatccgaaca aagaatttag aaaagagatt aggggctacg tttaataaaa tgtataatac

277381 aaacaaagag atattataca tagaggaagc gctccatagt aatttgacgt taaaatgcgt

277441 gccacgaaag aaattattaa aaaagaaaaa tcttgccagt atcatgaaag aatttcccaa

277501 gtttgggaaa atgtgtgtgt ttttttttaa cttttatatt aaacagtatt tttttttcga

277561 gtcgctctcc gttgtatgcg gtcaaatgga agaagctggt attggggtgc tccagcccag

277621 aggtgcgagc agtattccat gtggggccga acttgcgcct tatacaattg aaggcggtgg

277681 tccgactgga aatactgctt cgcgcgactg agcacaccca gctttttaga ggccagcttg

277741 gccttgtctt ccaaatgacc gcgaaactga acgtcactcg atatgtcaac gccgagaata

277801 ccgatactgg ctgcggcaac caggggagtg ccctcgaaac gaaggtctac gacaaaggga

277861 tcttttttag cggtaatggc gcatacttgt gtctttttgg gattgaagtc gacaagattt

277921 tgacgacccc agtccgagac ttcacccaat agggtctcca gtccagacac aagtttggtc

277981 cggttctcgt aggcagtagc ccgagaaacg ttagcacggg ccgtgtacga tgcgtcaccc

278041 gtgctatcat ctgcataaca atgaatgttg cttatttgca acatgtcatt gatatgcaga

278101 agaaacaggg taggcgacag cacacagccc tgagggacgc cagcattaat tggtttgggg

278161 tcagagcatg caccgtcgac aacgaccttg atgctccgac cctccaaaaa actggcaatc

278221 caggtgcaga gtttctcggg cagcccgaag gaaggcaact tcgaaagaag tgctttgtgc

278281 cacacccgat caaaggcctt ggccacgtcc aaactaacta ccagggcctc acccttgccc

278341 tcgattgcct ccgaccatcg atgcgtgagg tactcaagaa ggtcaccagc tgagcggcgc

278401 tgacggaaac cgtactgacg gtcactaatc agctggtgct cttccaggta cctcatgagc

278461 tggcaattga tggtcgtttc cagacttttg agaacaaagg ggtaatggcg atcggtctgt

278521 agttggacgg atcggagcta tcgcctttct ttgggatcgg gtgtattatg gcagtcttcc

278581 aagacttcgg gacgacgcct aaggaataaa ggtaatataa ctaaaacatt cattaaaacg

278641 ttaattatcg ttttccatta ttataatttt atttaaaaca atttaaatcc aaaaaaaatt

278701 aattagcgtt ttaaaaatgt ctcgattaat tataatatta aactttagca aatactatga

278761 aacaaaaatt aaatttgaga aaagaggtta cattcggtcg actttttatg tcggcaaatg

278821 taatttagag gtcaacacaa ggggaaatct ccctacgtga gacctccgga ggtcaaaaaa

278881 atgcctcact tttaggcttc gagtgttcca tctatagtat aatatatctt tgaatacaaa

278941 gttaataatg taactttatc aaaaattcta atcaacacaa cgatgattga aacattaagc

279001 atagataatt tatatcgttt aaagttaatc gaaggcgacg atgcggtggc tagacgttaa

279061 aacacgacgc gcgtaaatca cgccgcgttt gacaggcgct gtcagtgacg cactaaaacg

279121 gccatcttgg atttgaactt tcatgttaaa cttgtcttgt ttgtttgtat gtgatagatt

279181 tatattgaga aatatagtat aaactttata ggcctctagg ggatgtggat atggcggggg

279241 aggagacgtt tagcaagagg aattatcctc ttgctgcgcg tttcaaattt tataaaatac

279301 agccaaacag ccaaaaatca ctacaagcga gtcattctct ggtcccgacg agcaacttca

279361 taatgcgaga aactcggaat agagagaaac ctctataagc gatacaaata tcgcgttccc

279421 ttggactctc gcgtatcgtt tatgtattta tattttaatg taacttaaac agacacaagt

279481 cgatttttaa ctttaccatt ttcataagag actattttgg gacagacatg cgaagtcggg

279541 ttcacaagca tgtctcagag atatctatcg atgacaagtt ggaagtaaat ccgtccacac

279601 tgaacattca tcacgctaag tgcttgctta acaatattta tagaaaaaca ttttcgcgcc

279661 atttttaaac gataaataag gttataaaga tggctgctta tttacaaaat aacttctgta

279721 ttgtcacata tttgttaata tattgtgtta tctatatcta tactaatatt ataaagagat

279781 aaactttgta tttttagatt tttatatttt tgtatgtatt taaatacgag taactcaaga

279841 actacttggc cgatttcaaa aattctttca ccattagaaa gctacattat ccccgagtaa

279901 cataggctat attatttaat tccacacgaa cgcgaacaac agctatttaa tgataattgt

279961 acaacaataa tccgagagct gttagttgtt ctaagtcaat caaaatggtt cgtgaccaca

280021 ctataagtga aagcaaacta aacttatacg cccaacatgc tcctaattgg cacaacagcc

280081 cctaacaatg ctttactatt gcctacgtca acatttgaac tcaaacatcc acacaacaac

280141 attacattta cacctctaaa aataactctt caaccaaaaa ccaaacgata acatacttgt

280201 ctaaccgtaa atatatcttt gtacaccaac atgagatcta caaagttaat tttgcggttt

280261 ttaaactgtt ttaaggtata gttatttggt tggatgtctg taaagttaag tttgtagtgt

280321 gttatgaaaa actataatat ttattgagta cttatgttga tcatgatcag tacacttcgg

280381 cggtaaacag ttttggtgat aatgagagaa ctgtaacaga aagcattcct ttttttagtc

280441 actccccgtt attccgaaaa acttggaact aaataagata gaccaggggt ctcattggac

280501 aaaattatgt atcactagca acctgccccg gcttcgcacg ggtgtaaatc ggtataatgc

280561 tgatactaaa tactgcaacg tttgcaatgt aagcgctaaa aatgtgttaa tttacgacat

280621 catattaaaa cctctaaatt cttagagtta ctctactata ttgtgaatat attatacatg

280681 aaaaccttat gaatcactat tcaaactaat ctattaaaaa aaactgcatc aaaatccatt

280741 gcgtagtttt caagatttaa gcatatatag ggacaaacat aaaaaagtgt tattgtttta

280801 cttatactgt gaaaggataa gaagtaccag gaacaaggat gttctgtatt ttggaacaaa

280861 tattatgaat gtaccaaacg tagaaagtac aacagtaata acgctaattc acgcaaaacg

280921 tcaacgaaaa tatttaaatt aatattttta caaactaaat atttcactac gacacatggt

280981 ttattaatta taaaaacgtt taaaaataaa aataaaatca tttcccggtt agataatact

281041 cgtttttgat gaaaaaagaa taaaataatt ttcataacct ataacgataa ttgaagaaaa

281101 ttatcaaaga tggcggatta acaaatataa caaacaggga attattagta atttgaaatg

281161 aaaaacggta gaaatatgga tgggaaaaac gaggattccc gggtttcggc accaaaaaag

281221 atgtggcgtc acggtgaggc cagaaaggag aagaattaaa agggttgagg acgccatcgt

281281 cttgatagaa tgtccgtgga taagaaacgg cgaacggaca cgggatgaga tggctacgtc

281341 gtatttcgaa gttcgttcct agaaatgagt gcgggactta ccatttaacg atgggcagct

281401 gacgcacatc ggagccggcc tgctgctcca ctgcatcatg ttgatgcccc tctgtgcact

281461 ggtaactcac tcgcggatac ggaggaacgg atatagggag cgaactcctc ctcgcggttg

281521 ctgcttctag actatctgct cgcggaacat cccggatgcg ctatagggcg cgtccttcct

281581 agttcgaatt tcgaacgcga cgcaacaaat agaggaagaa aaaactagag gaagaaaaaa

281641 cgaaggaggt cgtataacct tataatagat agaaagaggt cgcatgtgaa cgaatggaga

281701 agttcatctc tgaacgagta tgtggtcgat acaatccccg agtaacaaag gctatgagtt

281761 ttatttcaaa aaacagctag tttactaata taataaacta aaagtaaagc cttgtacgca

281821 ctaggatagt attttagtcg agtagcaaga atccgaaaaa aaggatttac taatctattc

281881 tattaaaatg tcgctgttcg gacacgttta gttacaaaac aataccctag tgcgtaggtt

281941 taggcttaaa cgagtccgaa cagtagtaaa tcctttgatc ggataaatgc tacttgctac

282001 tcgactaaaa tactatcctg atgcgaacaa ggctttaaag atgcaactat cacgctctat

282061 ttagcataat atacgtatta ctacgtaaaa aaatgtatac ttttttacta agaaaaaact

282121 aatttaatta atactataat aattagtaca taatttataa cctaactttt ataaaaatgg

282181 cgggaaatca ttcacaaaac gcgcgcaata tccaacaaga ttcacataag aatttgagtc

282241 agaatgtatc ttcgccgttg ttaatgaaat gcccgactgc cggggaccac acacgacgct

282301 cgccacagat gacgagatag tatggataag actatgaatt tatttttgtt tcttaatatt

282361 gtccatggtt taactataaa taaataaata actcatttaa ttcgtatgaa aacattaatt

282421 tatacagtaa tggtgaaaca gaaattacat cctaaaagca aaaaaaaaac aacaattata

282481 attaaaaaag aaaaagtaaa aatgttttct atggataggg cctttgatta gctattccta

282541 taaatgtatt tatgtttctt attgttgtat actacttaat ttattgttta attaagcagt

282601 cttaaaagta gacgcatttt tttgttttta tgacttacgg cttatgtaac agcgcttaga

282661 tttttctaaa aggtgaagaa tttgcagttc atctatattt attagtatgg ataaccttgt

282721 atttatttac cgcaatatag tataattttt aatgaattaa tctttagaga aacagaaact

282781 gtactattaa ttttgatatt tgcttttggc ttagggtatg tccatactac atcccgggag

282841 caataagcga gcaatattaa taacactaat aacgtttgca aagcacgaca cgagaccgac

282901 acggggacga cacgggtacg tcacgagatt gacacgagga cgatatgagg acgacacggg

282961 gacgatatag agacgacatt gatgccgtat gatgatactc taggttaatt actttcagtg

283021 aactctaaag ccttgtacgc actaggatag taatttagtc gagtagcaag tagcaagtat

283081 ccgaacaaag gatttactaa tctattctac taaaatgtta ctgtttggac acgtttagtt

283141 actaaattac tacccaagtg cgtaggccta ggtttaaacg agtccaaaca gtagtaaatc

283201 ctttgttcgg ataaatgcta cttgctgctc gactaaagta ctatcctggt gcgaacatgg

283261 ctatagcgcg taagcgtaca taacttacgg ccggtttcaa tatgcaaaca taatccgatt

283321 ttattaacca gcagttgatt tacgagtaag acctatttgt ttgaagtata taaaagaaaa

283381 ggaaaattat ttgacagttg tcaacagaaa ttgatgtttt tctaaccctg cggcttcatt

283441 agaacgtgtt cgcagaaaaa acggaaaatt tgcttcaacg tatgcccgtt ttctgcgcga

283501 gatcggtcta atgaagccgt ggggtaatga ttttaaatca aacatcaatc taattttgaa

283561 atgatctgac gttttgcgat tgttgctatt ggaacaccga ctgtcactac tctggacatc

283621 gaataaaatc ttggattgcc acaatgtcaa atcgatggtt cgatattgaa accggccgtt

283681 aatctatacc aaagatcgac atccgtgtaa ctattttaga tatacataca aacaaaactt

283741 catccatcaa aactttctca tctatagtag tatttaaagg tttgtctaac aatgctttta

283801 tgtgattgta ttgtaaacaa aataagatca acatctgagc caattagttg aattctccaa

283861 gtcgcaattt tgtaagaatt gaagcaattt caaacagcaa ctaatgttat gcaatttaaa

283921 caaatgacta cacgattaat atcaggtttt cattgctgaa attcatgcat aaaacatatc

283981 gtcatccctt tcctttagta gtagtagttg gacccatgat gggcttcatt ttccttcact

284041 ccacgactag cgcgtatttt gtaaaagaaa cgcaacattc gtgctacata tacagggtgt

284101 cccaaaattc aacgataagc cagtgccagt gcatcaccag atcggtacct gcatagggaa

284161 aattaataaa aaaaatctaa ctcttttagt ttaaaagtta tgttaactta tttgaatttt

284221 attgctcttt gatacccgac cccattcaaa tagccactgt ctcttgagct ataaagatat

284281 ccctgctttt ttatttgtaa tgggtagctt agtaacatag ctaataattg cagttgaaaa

284341 cgggacctaa atatctgtag tttaagtaca aaaaaataat tactttaact gtttgcttga

284401 aattttcgaa tattttaaca tttcatcaac tttgtcatgc tgccgtgaaa aaaaaatgta

284461 tggagggtac cgtggcatgt tgttacaaaa gttggcgcat ctcatcaaga ttctaaaatg

284521 gtacaatgtt atgatagaaa catgcaagaa ccctgttaat atttttttca agtactgaaa

284581 aggattattt tgcatagttc caaagaactt tactaagtgt tcaaacaagt gttttaacac

284641 tagaaaggcg gccggtgtca tttgacccta atttgaaatt gaaacgctga taattttgtc

284701 cctatttgtt tatacaggtt aatatttcgt gacttttaat gaattacgat tgtaatgcag

284761 acaaaaatat ttagactgta ttttgtttat agaaatatga attaaaataa catttaccga

284821 caaagacggc gccagtcatc tgacaccttg acagacttat gtattgttat tgtgtttagg

284881 ataataatgt ttactttaat tttattgaga tatcgttttc aacgtataca cacataatta

284941 agattatatt ttgggtgttt atgagttttt gttgtgtcaa tgtttctatt tatagttcat

285001 atttctataa aatgttactc tgtatacttg tgtaaaacaa atgttgatat gattacaatt

285061 gttggtagaa aaccaattag atgttggtaa tgaagatgct caatggaaaa cgaagttagg

285121 gatagaaatt caaatacttc tgtagcctat tagtcacgtt tagtgagtaa caatgccttt

285181 agctgctacc gaacaacgaa ctggtaaaaa tcgttctggt gacgaactgt acctggtgtt

285241 agtacaccag attataataa gagctttcat aataaaacta tataagcctg tggttattgt

285301 agaaatatat acatacttga aaatggatca taggaggccg ggcagaagtc acaatttgtt

285361 agggtgcgat cttattgcgt gtcaatacat atttttgttt ttgtgttcac ttatctaatg

285421 ttattctgga gtcaatgtct aaatgttaaa actttatatg ttcttataaa taatcaaaaa

285481 aggctcaaag aatacttttt gtttagctat tgtaggtagt actcgaggca catttcaaag

285541 aaatccacta ctgaaactat actgtaattt ttatcacttc taaatgctgc tgcaggtgaa

285601 atgccagtag tcatcataga cattgtaaaa ataagagaaa caaagattgg tactcttcag

285661 aaggtctaac taaggcagtc gcaactaagc aaatatgacg atactgtata acttcaaata

285721 tttatttttt attgcaaatt gcataattat gaaaaatttt atatttatct gtaatcggga

285781 atacatttct tatcacctac aattattttt tttaatttga atataaacat atacataatt

285841 agagcctttg ttaggctgaa gtgtcatatg accatcggcc gtctttctag gtagcgcgtt

285901 tttcccgtct ttctagtgtt aagttacaga ttcattacct ttatttacac gttttggggt

285961 acggtacatt tttgtcgctc gctatacaat cgcttagtac cgaatgttag gtaccgccgc

286021 tgtcaggtta ttattaggtt tcctaaatcg atacactcat ggcgccacta gcgcagtact

286081 gtttacactg ccacaaagtc ccccggaaat gatcgccgaa ttcctttagt taacaaaaca

286141 ttgtacgtac agacaaacac ttacatcaat caaattatcg aaacgcgcat aaaagttatc

286201 agagatccac ttttatgaag aggcgttaaa caaatactag gagaacgtac ggataggaat

286261 accgtttaat ctatactaat acttgctgtt gttcgcgact tcgtctgcat ggaattaaaa

286321 aatataacct atgttactcg gagataatct agctttctat gcgtgaaaga atttttgaaa

286381 tcggcccatt agttcgtgag ttatttcaat acatttggag ggacaaggat gttctgtatt

286441 ttggaataag aaattacaac ggtaataacg ttgatctctt ttcggaataa agcggttaat

286501 agtttctaca gtagataaaa attacatata aaactgctaa acaaaactcg gtaacgtaat

286561 ttcagtccgt gagttctcta ccttcgtgta tagagcaaca caatcattgc ttgagatcaa

286621 aaaatgtatt ggtgcaaagc tcttggacta ttaccagact gtataccgcg accagcgtta

286681 ttgatacttg aattaattaa ttttagattc gtatttttta acttatgcgt tttcttaata

286741 agaatagagt taagtattat tatttagtaa tatgtagtag ctgaaataaa taaaatattg

286801 agaaaattaa cacgtgaata ttattaaaat aaaaaacaaa tatgaagacc tatttttttt

286861 ttcaattcgt ctttttttac attttagttt tctttaattg tctttatatt tgtcgaaata

286921 gaatttaagg cgcgcggaat aatccatcat gcgtatagca agcttaattg attttgatac

286981 tcggaacatt tactgtaaca tgataattga caacatctaa tacaatcaaa taaatcgaag

287041 gcgaaaaata cgaagataaa aaataataaa aaacataaaa attaaacgat caagaatcac

287101 aatacgtaat cagaaagcat tgtgtgaata ccgcaccgcg acttgggtgg tccgaaagta

287161 tccagtcgcg ttgtcccgct cgcacgcgta cgatataata ataatctgtc cttgtccaca

287221 attcggtttg gctggcaaag gctatcgaac gtagacaggt cacgcgctta attaataact

287281 gaacggtcaa attagttcta tagtctatga agtatattgt catatttttt tacaaatatt

287341 aaaatgaata attttttaat agaattaaaa aatatattca acgatattta atatcagttg

287401 ctaaaatcta tttaaaaaat attattttaa tttgcattgc ttatgtcagt aattccaaaa

287461 ggcgctaaac aaacataatt taaacgtttt ttctctattt aaaaatgtcc atagacataa

287521 aagctgatgc cttgtctata tcaatctatt catttcacta tagacctttc ccgtgattct

287581 accatgaggt ttttcagcga cttttgctgt aaaaaataat tttgttctta tttataagta

287641 cgtatatact tattaatatc atttcatata cttaccgcta aaacacatat ttacaataat

287701 aatattaaaa ataccaacaa ctattatata aacaacaaca taatcacgcg cgtaccccag

287761 aggggtaggc agagacaacg gacctccatt tggctcgatt ctaacacacc ttagacaaga

287821 caggcctgaa atgaaagcaa ttccgtgttt cgtccggtgt gtgtggtgtc ggaggcctaa

287881 ttttagtcca ctttctctac ctatcatttc cttataagga aattatggga aagggaagtg

287941 gatttccggg atttgggttg catgggaaga ggtattgtct tcttgctgtg cgtctcctcc

288001 tccgtggatt gagggtgggc aacgcatctt caattgcgga tgtcaataag caggggtcgc

288061 tttggatttt tcagggaatc ctggtgacca cttgctcgtt tgccactttg tattataaaa

288121 aaaacctctc tcgcttcttc tacgaactat tatatgcgat gaaaataaaa gattaagtaa

288181 tagttttgta ttttttattg ccttatacaa tcaaatatgt tcgtgatccg gggatcgatc

288241 cgagttgaga tcaattaggg cgatacaacg ctgttttgac agctccctcc tgtcatgaca

288301 gtttgacagg tgtcgtgaca gacgcgttgc gcgtgtgagc aattgcaccg tgactgaagt

288361 gatatgacta tgatcattgt tgcggttgaa aaagagttta ataattccgc cctcagtgtt

288421 gtcatccctt agtgtagatt taatatgaga aatcttcgct aagtgctctt tgtacacaaa

288481 gctatgcata cctcaagtat ttgtccgatt tataattgtt acatattact ttggtgatgg

288541 agtttgaaaa taacatgact ccaagtcttt ttaacacata accaacaatc acttaccaac

288601 ttagaatttg caatacataa taaaataata ttttgcacat acgtatttta cgtagggacc

288661 gtcaatatag tttctaaaag ttggggcatt tttacgccat tatcttaaca tctaatgaga

288721 ataattaatc aaaaatcgat gtttttatta cagcccatat ttgcaactac aatagttaac

288781 taaaatcaaa tgacaaattc aaaacaaatc gatcaaaata aagggtaaac aaatgagaca

288841 aaaagtccga tgccggtgtt agttacttac acattatcct gattaaaatc taaattgtaa

288901 attgatatat ttaccagcga ccacccatct cggcgggctg tttatttttg tttgcgcatg

288961 cgccgcgagc tgtgatagat agccgggcac cgattggctg tcgattaggc cgccacagat

289021 aaaatactat tttataatct gtgcaaacta tcgccccgaa tatattatac aggttacaaa

289081 gaataggaag ggtcctataa atgggctgaa cgtagaaatt ttgtcaaagt agccagatat

289141 gtaagaagtt atttctaata acttggatcg tcaaaaatag agttagtaac taaattaatt

289201 gaacaaaata cctttctaga catttattaa agattagtcc aataattaac gtatttagcg

289261 tagaaaataa tcaaatagtt ttttggactt gtcatgtatg aaataaaagt ccgatttaaa

289321 aatattaagc ttctcacagt ttaggtttga ttgtttctct caagatgtaa agagagtgga

289381 tataacaaaa atgaaaatgt cttgtcctat tattaatagg ttattaatac ctacttaact

289441 ttcaagaacg tttaatttta atttttatct gtgcgaatgt gactatggcg gcggtagccc

289501 tgcgcgctga ttggccggcg cctcgcgcct cgcgcattct attttcaaaa ttatttcttg

289561 cattgattcc gttcgatcgc ttttaactaa ttacaaatat tacactgcga ttgtttccat

289621 aaaatgcaag cgaatttaaa ctttattgcg aagcaataag tgtggtatat atttccttga

289681 tagtaataaa cgtttgttaa attagatttg tattttcaaa tgtttgttat tattattgat

289741 aggttaacag acatggcgcc gttatttagt tctgtcagta attattgaaa tagatcattc

289801 gtctgtattt attgaggcga ttagattaaa ttattacaaa cggacactaa taaagtaatt

289861 atcagtggcg aagcgtccat acaacacgat tcctaccggc ttcccttcgg cttacctttt

289921 cagatactaa tatataaaac tactattcta cgttgtctaa gtattgcaag agatttagtt

289981 acattgcgaa ctttatacaa aacattcttt aatatgcgtc acaaaacacg caatttacgg

290041 atcataactt tacaatcgta atacgcgtgc cgaggtagta ggaggcgggg taactcgctc

290101 ataatattgg gaatttaaat tattaaaatg taaagaattc gtaacggata tacattggca

290161 acctctctac gaaataacac cttatttgtt ggtcgttaag gatgagtttg gcttgaagct

290221 ttccacttcg aaataagccg aatgtctcac ggcttctaat gcttattttt tgtgtgttaa

290281 ggtttacttt tgcgtgaagt ttgtcgacca accagagtat ttttttgcga catgcaggcc

290341 ggtcatccga ttgttttttt attgttatta cgcgtgtttg gcaacggttc gtatcttaat

290401 aacgtgagtg tatatttact aataataatg tctcacggaa gtttaaacag tgaaatagtg

290461 atattttatt tcctaagtgt tgtgtttaag aagtatattg cgtatctttc atcgttaagg

290521 tttgtttgtt tgtttctttt tgtttcacgt aggccagagg ttaggttagt aatggcggaa

290581 ttagtacaaa taggttgttg ttttacagat tacctattaa atgagttaac tttgttttca

290641 aaacaagctt tcgaaactaa aaaatatata attaacggtg gacgggctcg cctcgatatt

290701 aaaattgatt atagacgtta taaactgtaa acaacgcaac gtaaacacca tagtccatac

290761 aaaacatttc gccgtaattt tggaaaggtg aactaacaca cactaattta aggaatgtaa

290821 tttatttttg acaagatatg acgttgtgaa aattcacgcg ttagacgttt tttttggtcc

290881 ggctttacct ttctttttct tcaccccacg ccactggtaa ttatgtatat gaatgaatta

290941 aatatagcca tttagatttg cataagatat tttaacaagt ttatcgtata atcacactaa

291001 tattataaac tctaaagata cgatgtattg atggatgttt gtttgaagat atcgccaaaa

291061 ctgcgtaacg gatttcgatc aaattttgca gagatgtaga acattgtcta tgttgcgtca

291121 ggaaggaagg aagaggaatt ggcaatgctg aggacgacac gtcttggggg aatgtacttg

291181 gaaagggaaa cggcaaacgg acacgagacg aagtctaaag gcttcgttgt agtttgggat

291241 tcgttccttg aaacaaatag gtgcgggact ttcctctggc gacagctggc acaccttgga

291301 gtaccctgta gcaacgcgat aaggagagca atcttcacct gggggttgct gcttctcgat

291361 tcgttttacg ttccgcattc gcgatcgaac gcttcgttct tagttcgtat ttcgaactat

291421 aacgcaaaaa atagacaaag aaagaacgaa ggagaaagcc ataactttac aacgaaaaga

291481 ggttacatgg ggacgtaaaa agaagaaggt cgtatatgat acggacagcc tggaagaata

291541 catgtatcta ctacttattt ttttaattcc acgcgaatat agtcgcggga aacatctaga

291601 aagcataact ttgaataatg tttttagtaa aaagcttaac aacaatgttt gcttattagt

291661 gtggtcaaag tatacaaaat taactaaata ggagtattag agatagatgt tttggagatt

291721 taagacccag tagtataaat gtataaagaa tatacgtttt tattaaggtc tgttttctaa

291781 ttatacaaaa atcaagtttt aggacccgtg tcctgatttg actaaaaata tatcaagggc

291841 gtattcgcct tctctattgt ggctgagact aattttacat aaacttatgt tcgttctaaa

291901 ataacgttaa attaagaaac aaatctacat caagacacac aagaataacg tcgccatgac

291961 gaatagaaac aaactgccat gagagctgca ccccctttgc aaatgctctg atttgacaac

292021 ttcttcattt ttacatttcc tggaataatc cctaatacaa gcttatctag gctatcatta

292081 aaattattta aaaacagcta aaaaaattat ataactttta atgtttttaa aaagtgttag

292141 aaagcagact ttagtagtaa aagtcaaaag aaattctgct acttaccgta aatctttcag

292201 agtctaactt taagcgctgt ttgcggtcta aaacctattt agtgcttaca cctcggcctg

292261 tgtgtggcct agacgctaac ccgtttacgt tcctatattt ataaactaaa ctaataatcc

292321 aacgtaatgg ggaccgcgta acaccaaaaa atgcagtcct gtacagctca aacgagattg

292381 gctctcaaat taagccaaat aaatatggac cagttaacga aggaattgga agcgatttaa

292441 gggtcatgtt atatcggtcg tgtaatacgt aagtctgcga cgtgggtggt cggccatttt

292501 tatttaaccg tcaaaaggtc attcacccgg tcgattactg tcgtcacacc tgtgaaggtc

292561 ttttttgtcg gatacagttt tacatcttgt tattaataac agattttttt tattttcgac

292621 aacaatgaga ttgttgtcat ttgatgaatg tttttgactt gtagtcgttg ctgtaagtac

292681 agacttgcag ttgaatgttt tgataagcaa attatgcaat aatataatat aagcaataat

292741 ataatattaa gataagcagg tatctttaac tgacttccaa cagaaggagg ttatttttac

292801 aaaattattc actgaaccgc actaattaga tatacaactg ttttgaaata tttggtattt

292861 tttcatggtg taaaaaaata aaataaataa attattattt aaaaagttat taagaaattg

292921 tgtattgtga cagctaactt gtactaatac agttgaactt ggataagcga gaactggatt

292981 agtaagaaac ctctatatgg gagtcgtggt atggttccga gggactagga ttagaaagaa

293041 accttatcca agttcaactg tattagtaca agttaggtgt ctcaatacac aatttcttaa

293101 taacttttaa ttaataattt attcattttt tttacaccat aaaaaaccaa atattttaaa

293161 acagttgtat atctaattat tgtagtaagt atatatatat atacttaact accattaagc

293221 atttttaagg aatgtttgtg tgtaaaaata atttagatat tcctgataac tttaaaacgc

293281 taaaatggcg tgaaaatatt tgttttttat tggcagagaa gcaataacgt ccgataaagg

293341 gtgtgtagag tgccctccat ggagtgtaaa aatatcacga cttgcgccac acttcgtcag

293401 gaattttggg gcttcccttc ctttaatagt cggtttacat gcacgtgtaa atttataatc

293461 tcaaagtaaa cctagaaata gtattaatgg cttaggggtt aagcacttga cgtgtaatct

293521 acaggtcttg ggttcacacg cacctagcat ttcaaaataa cttatatcta gaaatagtat

293581 gagttgctta ggggtttgtt ccctgccatg tattcgtgtt tcatatgtgt ttcctgacgt

293641 tcttatgatg aagaaagaca ccgtgatgcc tgcacatatc ggcaagtaat ctgcaatttc

293701 taatttcctt tttattctaa ccttttcctg actcttactt acttattatc gcgttgttat

293761 ctaaatagaa tattctataa tctggaaatc tatactaata ttataaagag aaaaactttg

293821 tatttttgat tttttgtatg tattgaaata actcaagaac tactgggccg atttcaaaaa

293881 ttctttcacc attagaaagc tacattatcc ccaagtaaaa taggctatat ttattaattc

293941 cacgcggacg aagtagcgca caacagctag tataataata aaattataac ggtcaaatgt

294001 taaaacgcaa atcaaattta agcactgttt aaaatctatg tggcgtctaa atcgagatcg

294061 tgacgatcga agatcgtgga catggcatgt aagacgctct taaatatttg agtggcgttt

294121 ataaattcgg ccgtttgact atctaattca agcgctgcat gtaaccccac tctaatctaa

294181 aggcacgctc ggcgcgtgtc gcgacatccc tccaaattaa ctttgttttt ttttctgtcc

294241 gttactattt gacaatggat tggagatgct ctcaggatcc ttgtgttgag tgtactcgat

294301 atttgacgat gaaattaaga ttctgtactt aaacttagtt ttaaatagaa tttgagaaag

294361 aacttttact tatcgcttta aaaactaagt tctaagtaat caggttctga tttatgatac

294421 gatttgaaaa tcacgtccat tttaggttta tgaagatcag cttatccatc ttaggcggtc

294481 ctaagagtcg acttcagcaa agtgaaaatc gatgagttat taagtatatc atagatgaga

294541 aaaaaaaaat ctaagtttgt cggtcacgaa tgaattctga ttgtcagggg gagcattgag

294601 tttcttgcta ctaccattat aaagctccaa atactattaa aagaaaatat taagactttc

294661 atatgtagac atttacccga ctctcttacg gtgaaggtaa acatcgtgat gtctgcacat

294721 atgtaaagaa attcgaagat atgtgtgaag tcacgcaaaa tacgcgttag tcgtggagtg

294781 gaggaaaatg gagcccatca tgtgtcgaac tactaccact actattaaaa gaatatttaa

294841 atacgcattg gtttagtatt ttagtagcag tgaaaattta aagtaatatg tttgacaggt

294901 tacatcgatt taaacatgca aaacaaaacg ctgcacgtct tccgaacccc tttctatcag

294961 cctgacggga gggggcttga agaattttat tagaattttt gcggcggcgt ctcgaatcgg

295021 gaattcgaga tcacttcgtc gcgaagaagt ttaatttagt tgcgtttgtg cgcgtgtttt

295081 tataagtaac tttagatagg ggctgtttga gaggggtgac aacactatga tagattcctg

295141 ttgcatttag gttaggttat tcataatgac tttatacaat aactagctat tgttcgcgac

295201 tttgtccgcg tggtattaaa tattatagcc tatgttactc ggggataatg tagctttcta

295261 atggtgaaag aatttttgaa atcggcccag ttcttgagtt atttcaatac ataaaagtat

295321 tattgtctta gcatatgcta agatagatgt gtggtgtgac aaaaatggat aaagttaaga

295381 atgtgtatta gacgaggttt gattaataaa aagtattgtc gaacgttttg tgtttctaaa

295441 ctgtgatatt atgatgtaaa atccttaaaa aataaaatgc gtacaaaacg tttttagtct

295501 tacctattta gtaatatgtt ctgttgtgaa aatgataaag taatgcttaa tacattttag

295561 ttatgggaat taattgaaat aacagtactt atagtttttt tatttctttt tattattatt

295621 tttctttacg accacttcta catagtttaa aagttgacaa tattctgtgg aaacaatcca

295681 acacatttat acattaataa ttttcaattt taataataat aattttaatt tgatgttatc

295741 acaggttacg acataaaact gcaaatggtt agtagaatac agataaaaat acacatgtca

295801 aaattaatac cgcgaaaatg aaatgacatt tgtttttttt tacaaaagcg gtggaaggtc

295861 tctctagaat acagccaaac tatttttctc tactaatact gctacattgc acaataaata

295921 acttaataat attttgttat tctaacctct aaaatatatt tacaccatac tctatacaaa

295981 tcgagtggga catagcagct aaattgaatt attaacaaat tgcttaactt actttagact

296041 cgttgttact aagtggataa tggcaaggct attaaataat tatttctatt ttctatgcga

296101 tggtttaact tcgacgcccc aacggccggt aattggtgta aggcaaaatg gtccacatta

296161 caatgttctt gcagcaaaca agatggccgt tttgacagat ggataggagg tatagtccta

296221 caagggtatt atggcacttt tttgacattg acagggaggg cacctacaaa gttttagcga

296281 taacaatagg taaaaatttt ctaacttctt tcaaaatccc tcaggtgcct ttccctgatt

296341 ggttaaaatg tgacgttgtc aagatgagat tgttagttca catattttcc gagtgccaaa

296401 atatccttgt agacctatac gactgttcta tgaaatagat taaaaatatt ttgattttta

296461 aacagatatg aatttaataa tttatttaca attttctttt catttcttta atgacttaat

296521 tagtaattaa tttacaaaat tatgcatcag cgtggttgac tatggcctta tcaccccttt

296581 agggtaggct ccgagctcct tagggtatag tgagctggtg atgataatga tgatgcatct

296641 gtgagttttt actaccgaaa ctcttgtttt attaaacctt tcatgtcata aatgctattg

296701 tcctctcttt aaacatcaaa aaatctacaa aaatcaaacg attatacaaa attcattgta

296761 ttagtattat taaatcgaga ttcgctaata aatgaaatca tccacactca tagtcgttta

296821 agtattactt aaatagtcgc ttaatgtaga tttttgatac taaatctaca ctttaatacc

296881 ttcagtgcga cggttgctgc tgcaaaataa ttggacacta agagcatctt ttaacgtagc

296941 cttaagactc aatactgaaa cgtcacatgt gtaacagcct cttaaatgtt aaaatcccac

297001 ttaaactcca aattacatac aaattttgac gtttgtttga ttataaacgg cactttatta

297061 tttgtttcaa tattgagcct ttagtatgat aaagttcaag attagtcact ctaagtatga

297121 catttagttt attaataaga gaaatctgca ctaagggatt gtataagcag atattaaaca

297181 ctctgagtgc gacaatatgt acataaatac aaagttaaag ggtggatgaa tattcctacg

297241 agtattgcag aacagacaga taaaaagaga agtccatata aagtggtcgt gttgatttta

297301 ttctttaact cgattttaac agcgggtttt gtatacgctt tatgcatcct taaatacatt

297361 gggcattgtc actggacatt tgttaattaa gacaatttca aatttgacca atattatggg

297421 cgaaatgtga tttttttttc ccagttcggc gtattaggtt gttaaatgac ttcgtccgag

297481 taatccgagt ttctcgcaat ggaggtagct cggcgggacc atagaatgac tctggctttt

297541 aatatttctc gcttatccag atttgattgt atttgcagac aatttaataa gctagtcagc

297601 aaagagatat tatacataga gggagcactc catactaatt tgacgttaaa aagcgtgcca

297661 agaaagaaat tattttaaaa aaaacttgcc agtatcatga aaaaaaatac caaaattggg

297721 aaattgtgtg atttttttta atttttaaaa taaacagtag gtaaaactaa aacattcttt

297781 aaaacgttaa ttatcgtttt taattattat tattttattt aaaacatttt aaatccaaac

297841 aatttctaac aaagactatt ttcgttttat ggttattata catttttaat tagcgtttta

297901 aaaatgtctc tgtttattac aatattaaac tttagtaaat actatgaaat aaaatttaaa

297961 tttaagaaaa gatgttacat tcggtcgact ttttatgtcg gcaaatgtaa tttagaggtt

298021 aacactaagg gaaaactccc tacgtttgac ctccggaggt caaagaaaat gcctaacttt

298081 taggcttcaa gtgttccatc tacagtataa tatatctttg ctagtcaggt tctaaataga

298141 ccggagttca cgtatgttcg gtgacatctt atcccaaaca ataaaaggat catttcgtct

298201 tcattcctcg acgcgagcgc gctcttatga caaaaaattg tttcaactca actttgattg

298261 gttcaagctt tatgattttt aaattgctaa tacttttatt ttattttaat cttacaattc

298321 aaggttataa agttatatct gtgacagcga tatagatttt aatctttgag aattcagttt

298381 tttattctgt gacagtatta gtggcacata ttgattggga aaaagggttt tctggattta

298441 aaaagctttg ttgtgccaaa attacttaag ccggctaaag acgcacagtt ttaaccatac

298501 tatttgactg cgtagttaaa actgacgtgt gtaggtaaaa ctgagaactg tagagttcga

298561 aagttgtaca gctcaaaaac tgaacgtgtg tagccggctt tatattagtt aaatattttt

298621 cagtaaaatg gcaaacgaag tatggctact tcaagtcgcc aaaatagcga agtgatcgtt

298681 gcccatagac atcaacattt gttgatagat cgacgcagat aataaaatgt taccatagat

298741 aagcaaaaag gctgagattt attattaagt tcgtctgtag aaattatata caaaaagatt

298801 ttccctagaa atataaataa ataacatatt acgcaaactc gtagtagaag gtaaatatat

298861 acctacagtg tcgaatgttt gcaaatgcac taactacata ttagcagtac tttctccaat

298921 attgacagag aaactggaca gtatgaacgt gacaagtcaa tttgaaggta aatattgacg

298981 tacaagacaa cgatgacgtc atcttttgta cagtgtacaa aaatttgaac agtaaaatta

299041 ggttaagaat cttgagtgac ttatcgaatg tcattgtcgg tttcatacat atttgtcgat

299101 tccatcagcg ttatcgccaa ttaaaaagta cattgggtag caccctgtac ctttgaccaa

299161 tagtagttaa aatatgcaaa caaaaggcaa tattgtttct aagtatatga ttaattacaa

299221 atgactgata gtttctagaa tttcgttttt cgtccgatga gcgtagtgcc ggaggcctca

299281 tttaaatcca ctttcccatc ttacactttc ctataaggaa atgatgggaa agggaagtgg

299341 aatttccgtg ggagaggacg catatggaga ggaattatcc tctttctgtg cgtcttctcc

299401 tccgttaagg gtaggcaacg catccgcaat tgcagatatc tatcaaaaat gatatgcata

299461 agcaaagtag ttgatctaag tagtattttt gcattatcgt acaatagttt agtaaagggt

299521 aatagttcat attcaaatac gttatctaag taaagttgtc acgattctcg tgattgttag

299581 caatttgtcg cagttaaaac ttctgtagaa acatgacact tttcataatg atgtagttat

299641 aacgtttttt tcccaaattc tatgaattca aatattataa tttctaaatg tttaatatta

299701 tggtttttat tatagttata ttttcggttt tcaatatttt aactacgtta aaacattgat

299761 cgtttagtga ttttatcaag atgtaattct acgatcgaaa gtttttaata aagagtccaa

299821 caataggagc aatagtgtca agtattcgcc gctgttaaat atacggattt taatattatt

299881 tttatctttg ttacataaaa aaaactttcg gatcaagaac atttcatcca cccttaccaa

299941 tatctaagca actagaaact taacaaaaca ctttcaatgt ctattctgta gttccttttc

300001 tctttctctg gccttcatct ccagttcttc ctcttccttg gtgaggggca ccgtgctatg

300061 attgtatagc ccttgagcca tgagctgcag tgcgagtggg ttcctctgac ccgaggcctt

300121 cttgatcttt gctcgcttgt tctggaacca gatcttgatc tgcgcctccg ccaggcccag

300181 ctccgcagcc agcgcctgcc gccgccgctc cgtcaggtaa cggttctcag caaactcgtg

300241 ctgagaataa aagaactaat aagtatgctt gatttatgtc ttctaagctg aaataattat

300301 aagacgtcca taaaacagtc tactgtcttt tatgcccgag atcaaacata gagcccagga

300361 tagagcgatc tgactgctcg ccgtggacgc cctctaccct atttcgagga tataggacat

300421 taagtcaagc agcagatgct agaagaagtc tactccatgc ctacctttag tctagctagc

300481 tgaggtccgg agaaggcagt cctaggtcgt ttctcatcag aagtaggtcc ctccccgggc

300541 ggcttcttcg gtcttctcgt ccgaggacct gggacagaac agaatatgga atcagacaat

300601 cctaattaca atgatagctt cgtcgtgcag gaaatgaacg gattgaaact accagggaac

300661 cataatctta cagaagattg gcttgaagaa gtaaattatt aaatctgtac ctttttctta

300721 cgtaaaaagg aataataaag cctagctccc accgtaaaat agtaatgagc ggcagatggc

300781 gagctgagcg ggctcaaatg tgcttgcgtc accaccaaca aagagctgag ctaagcgagc

300841 acaacggcat aacgctgctg tcttatttga cacagctcgg ctctttttga cagatcttat

300901 ttctctttgt cggtggggac acaagtatgc agtttcgtat gtatgtggga aatggtgccg

300961 cccagctccc gctcagctct ttgctctttt tcgttgggcg ctcaccttaa acgtgcgaca

301021 tgtatgtgta aataattgat aaaagtcttg tttcttataa aaaaaaagac cgcaaacagt

301081 agccgccagc gtcccactgt taaagaccta attacgttgt ctttttttaa taatgttaaa

301141 taaaatatga ctttatagta ttaaaaataa agtttgtttt gcgttgttta tttaagtatc

301201 catgtttaaa catggtattg acagaattat tttttgttac tttaaaagtt ttggtatttt

301261 acgaagtatt tttgtatctc tttctttttg aaattagtag ttttatgaga atttttaagc

301321 agtttttatg gacatatgta ctctaaaata gtttaatcga gattaatttt acctgatttt

301381 aagtatcaaa taactataaa acggtggcaa ataataaatc cgctgtaact agttatccgt

301441 ccgcgtgaaa ttaaaaaata tatcctatgt tactcgggga taatgtagct ttctaatggt

301501 gaaagaattt gtgaaatcgg cccagtagct cttgagttat atcaatacat acaaaaatac

301561 aaatctttcc tatttataat attagtatag ataaattacc ccagtagcat gataattcct

301621 taatagtatt aaaacacagt acaaaatcac acataaaaat acaaacataa tattcaaaaa

301681 cacaaaaaca cacaagcaac aaaacaatat gcacacaatt tacacgttca atactacaca

301741 tcctttttta cgtcatgggt ttttgtagca ccctgtatta tattgccatc tctgtcgtat

301801 ttattaaaaa aaacagagat agctgttata cgcaacagtt gaaaatcgta gtagcattga

301861 acgtaggcaa catgttctaa tgttgaaaca acacaaaaac atactcttat ctctatattt

301921 ttaagtaaaa tgagcaggat gactatgttt ttatacatgt aaacagcagg cagtaaaagc

301981 agtcttcatc gttgataccc gagtcaagcg ggcgatgcaa tatcttaacc aattcacatt

302041 ggctataagc gattgtttgc tataaaattg gtcaaaatat tgcaccggcc gcctgatcgg

302101 cagctgcact cctatgttgt cctggtacca aggttggcaa ggttggcact cagagtgatt

302161 ctaagggaaa cattagttag tgtaaacaaa gacgacacta agggccgaat tccgatacgc

302221 cgcttataat ttaacagtcg ttattcgtaa ggaattttca tgacgctgtt acatatttat

302281 atgacgattc aggattcggc catattaaaa gagggcctag cacgaatatt tgcgagaagg

302341 tatctgtaac cctaagactt cagtcacgca aaaaacgacc ttaggccgaa gtaaaatcca

302401 ttgtttgcgt gaactcggtc taatgaagga taacgtcata aaaaatctct ctcgcttttc

302461 gtggtaagcc ctctagaata gtaaacataa acgaagaatg ttaattcaaa aagcttcaca

302521 acacaaaata taacacaagg atgcacaaaa atgtacagtc gaacctggat cagcaagcta

302581 caagggaccg cgatgtgact aatacttata gaggtttctc tctaatccga gtttctcgct

302641 tacctcgtaa ctcgttgcaa ctatagaatg actctagctt atcaaggttg gactgtaatt

302701 agatcccgaa atatcatgct aatagggtaa gcagaccaaa atgaatagta gtatttttgc

302761 cctatagtcc ctagtaccta atccactttt ataaaacaag caacttttat acccttattt

302821 aaagaatttc tgcaaacgaa taaaagttta acaaaattaa gttatttata gaaatgcata

302881 aagctatttt aaatttaaaa aaaagtttaa ttataatttt aactaaatac cgtctaaagc

302941 cttattcgca ctaagacagt gctttagtcg agtagtaagt agcaaatatc cgaacaaagg

303001 atttattaat ctactctact aaaaattttg ctcttcggac acctttagtt actaaattac

303061 ttgctactcg tttagagcgt ctgtggctca ggggtttagt tcttgacttg taaattgcag

303121 atcccgagtt cgaatcccgc cataccaatg tgattttcga ttttttcata tgtacacatt

303181 tacccgactc tcttacggtg aaggaaaaca tcgtgatgtc tgcacataac ggcgaagaac

303241 ttcaaagata tgtgtattca cgcaaaatac gcgctagtcg ttgtgcggaa aatggagccc

303301 atcatgggtc gagcttacag tattctacta cttgctactc gactaaaaca ctatcctggt

303361 gcgtacaagg cttaatagat gacatatgca acgaaataaa acgattgaat catcaaatta

303421 tcaggaagta taaaaatcaa tcattataat ttataatgta tagaacggca tttactaatg

303481 taaatatttt aaacaattca aataaaacct gtttatttta attaaagtat aaattacaag

303541 taggttttca ctgtaaatta tcgtcatccc gttcattcta tacggaagga cagagataac

303601 aatataaagt cgtacttgat aagcgagact ccaagggacc gcgatttttg tcttgcttat

303661 agaggtttct cttttatgga ggtagctcgt cgggaccata gaatgacgct cgcttatgga

303721 ggtttctcgc caggtttgac agtattgttt actgttgtcg atcagtgaaa gcctacggtt

303781 agaataccag tttatactag ctgttgccct cgagtttgtt cgcgtgggtt aaaataatat

303841 agaaaaacat gtcaatgaca cccgaggata gtgtaaccaa cagtaaaatt caaatctgtt

303901 tattagtttc gtagtttatt caacgctaac aaacaatcaa atctatcctc tttaatgtgg

303961 gttttctatg taaatacatc tcataagcct ctagaatgtt acaaataatt ttggttgtca

304021 acacaattcg actgtttgta ataattatgt tgttaagtat tgcattaaat ggtaattaag

304081 ccaccaggtg tcgcttacaa ttatgtatcc aagaacaaac tgttaaaacc attagacctg

304141 gttctcgata aaaacgaact aaagctgaag taaaatctcc gatttatcgc gaactcgata

304201 taatgaagcc gaagcatagg gaatgaaatt atatcagatt tatttcgatt atcaaatatc

304261 atttttatac aatttgtgtt aaaaagaaat ctatctatgt acgtataggt aaatcaattt

304321 attttaaaac acacgtgcct ttctctatgt cacagataat aaaaaaaaac ccgccaaaat

304381 agacaactcg accaccaaca aatgcggtac agatatcatt aaaatacaga taaaaaacta

304441 ttcaaaatat ttgacgttta atcgtaagct tgtgaccatg ttttgacaga tgaaaggtca

304501 gtagcctttt aacttttttt gaccaccaaa gtgataggct ttttgtgtca gccgtgactt

304561 ttgtaggtct aatttgacgt ttttgtgaca tttgtcacgg tggtatactt ttaaagtgac

304621 aaaaacatga agtcgctact catttcgtga gatcttaatt atattggaaa ttatgttgtc

304681 accaaattgt tatcatttaa aacgtaactg ttttaagacg ttacatcttt gttaatttta

304741 actaacagac tgtaaactaa taaaatatgt ttataagtta aataataaaa tattaaaatc

304801 tttttcaaaa tcgcgaattt tcttttagtg gagtagcaag tcatttagta actatacgtg

304861 tccaaacagc aacattttag tagactagat tattaaatcg tttgttccta tacttgctat

304921 ttgtactcga ctaaaatcct ggtgcgatca aagctttagt aatactcgga attgaagact

304981 tttaccgttt taatgaaata taaaattaat atataaaata aatgggtttg tctcacgttg

305041 ctgccgtgag ctggtcccgt acttacttct tcctgaaaca aaagagaaaa caaaacatta

305101 gaacatacac caaacaaaga aaacaaacaa acatcgtcaa aatatccatg taaaatacag

305161 attattaatt ataaaaacaa taatatcaaa tcattgactt aattaatctg gaaaataata

305221 gaaattataa aacaaaatgt cctaacaccg acaatattta ttttcttgca attgtggcgg

305281 gtaacaaagc ttgtatagtt cataaaaata agtttatgtt tcgcaggttt tacgttaaag

305341 aaacatctaa aaccggctac agacgtttag ttttaactgt acagttggac tgcgcagtta

305401 aaactgacgt gtgtatacaa agctaagaac tgtacggttc gatagtgtag ttcgacgtgt

305461 gtacgtcact tttaactgta aagatcgagt ttgcgggtaa aactaaacgt ttttacccgg

305521 cttaatttta actaccaagt taaccatcct atacaaaggt gtaaacagcg aacgaacaaa

305581 caaagccaaa gggaattaag ccttgttcac acctggatag tattttagtc gagtagcatt

305641 tatccgaaca aaggatttac tactgttcgg actcgtttaa gcctaaaact agggtagtaa

305701 tttagtaact aaacgtgtcc gaacagcaac ttttaagtag aatacattag taaatccctc

305761 gttcggatac ttgctacttg ctactcgact aaaatactat cctagtgctt acaaggcttt

305821 aacgttagtt ttttctattt aaattttata gtgcgcgcct aaaagtagaa cacgacgtca

305881 gaccacccag taaaaagcga ataattaaga cattagacct cgttacatta tcttttaact

305941 aaggctcttc gcccacgagg atacactctc ggatacattg ctttacaagt tcgtatacaa

306001 gattcgacgc aaatgtatta tattcagcaa tccttaaaat cacaggattg taactatttg

306061 ttattttaaa aaactggtag taagcgtcta gtttctatga ccgatttaaa tgtcggcgac

306121 gcatgccggt atctaaaact ctatttagca accaaaggta ccacaaagtc tagggatcgc

306181 agtccaaaat tagtaccaat aaagtctatg ctctcagtag gcttgtatct atcaaggacc

306241 aggaataaag atcttctgtg ttacgccatg gaattttctg aaagaataag acgttcatta

306301 tctaactcta acgtagaaag taccatactg aagttatttg atttagaaac gtaaccctgc

306361 gttcacacaa actgtccaac gtctgggttg tgcgcttttt taaacgctct cgttcataca

306421 atcactgtaa aatgattctt gtttcgttag actagcgttc aaaatgcaat gatacggtac

306481 aaaaaaccgc cgtatgatac atttttttta tctcaatgct tttctagcgc gcgttgaaaa

306541 agcgcttttg taaatggggt gtaagccgct tagagattaa aatcgctgta taagcgttgc

306601 ctatcgtcgt gtacggatgg cctaagttcc atttcggaat tccattactt gtagagacct

306661 cgtttcgggg ttttaatgac cttattagtg tagaactgtg gatatcgttc ctacaaacat

306721 ttgatgaatt ttaaatgatg ggaaacaata attaaaatag ctttataacc cctagatgag

306781 gcagataaag aagttttaat attaattttt gtttttaatg ttacgcttgt tttataatct

306841 gtcttttcct ttcagaagtg tatccatgat ttctaaaatt aaagacgtta tataaataaa

306901 tagctgttgc tagcgactct gcgatgaatt aaaaaatatc ctatgttact cggggataat

306961 gtagcttttt aacggtgaaa gaatttttga aatccatctg gtagtttttg agttatttaa

307021 ttaaatattt agtataatag gtgtatataa cctatactaa tactaactaa tattataaag

307081 agagaaactt tgtatttttg tatgtattga aataattcaa gaaatactat agcgatttca

307141 aaaattcttt caccattata aagctacact atccccgagt aacataggct atattttttt

307201 taattccggg tggacgaagt cgcgaataac agctattatc taatataccc ttttactttt

307261 ttcatataaa ttaacagttt tatatttact cgtatttata atttaagtct ttattatatt

307321 aattacaggt cgatgtttgt ttaaatcttt tgattggttt atagtttaca gcggccgtaa

307381 acgcggctac atcaaagatt tcacacgagg gtagagaatt acggtagagc agttagcagt

307441 actaacattt aagcacaatt attacctact ctaaaactcg attgccgtga tacggcagtc

307501 tgaactcgaa gtaaataaaa gaccaaaata agttaagaaa gagtgaaggc atattttaac

307561 agtagcagaa cccgttttag catcacatag aaggcaggct tcacatggaa gctattccgt

307621 gtttcgtccg atgagtgtgg tgccggaggc ctaattttag tccactttcc cttcctatcc

307681 ttttgttata atgaaatgat gggatgggga cgccgatttg gtggggaagg ggacgcatag

307741 gaagaggatt tatcctcttg ctttatttct cctcgtccgt agaataagag taggcaacgc

307801 atctgcaatt gcagatgtct gggcagcggt cacttcgcta tttcagcgaa tccaaatggc

307861 catttgctcg tttgtaactt tataatataa aaaaaatgtc ttattttatt tagttacaag

307921 atataaccaa atatttggag aatttgcttg ttattttgtg taaaaataca aaattatctg

307981 ataataagta atatcaatac aataaccaaa gacgtaataa taaaaccatg acttgtaata

308041 attctacaat agaaaataac gtgagtatcg aataataaat cgatattttt ttcaatcacc

308101 gtgtaatcaa aatcaatata agccaaaagt tataaagaac taaatcaata aacgtacaaa

308161 aaactaataa aaaattaaag caaattcatc tcaagctaat ataagattta tcagttctta

308221 ataaagtcga taatcattta aatatgatat aattgaattg ggcattttat cagtaaagag

308281 taataatagg tctcttattg gaggcaaatg tcttcattac aaaacttcgt tggttgcacg

308341 aacgtgttaa aagacccaac acggcttcga acaaggagta tttatatttc gttatattac

308401 agtacaaata tttaacacta gctattgttc gcgacttcgt ccgcgtggca ttaaataatc

308461 tagcctatgt tacttgggga taatgtagct ttctaatggt gaaatatttt tttaagtcga

308521 tccagtagtt cttaactttt aatacatata aaaatacaaa atttatctct ttataatatt

308581 aagaaatatt attaaatagt ccaagtcggt tgcccaaagt atcttcgatt ggcggtctcg

308641 ctaataggat cgacaaaaat gtatgaaact gataatgaca ttcgataagt cacgtgcttg

308701 tcgcctgtga gatattgaac gtcattgcca gttccataca tttttgatgt tcgctttgta

308761 agcgcatgca ctttgggtaa cccccagtca atttaaaata tatttttaaa gatacatttt

308821 actatccata tttttgacta caaatccttg gagtgccaaa atggtgaaaa tcaaaaagcc

308881 acaacatatc tagtctaatt tttttttcat atcgttctta ttaattaaat aataacatcc

308941 ttcaaaagct taaattacat aagaagccat gtaagcatat ttacccgacg ctcttaagcc

309001 ttgtacacac taggatagta ttttagtcga gtagcaagta gcaagtatgc gaacaaaggt

309061 tttactaatc tattccacta aaatgttgct gttcggacac gtttagttac taaattacta

309121 ccctagtgcg taggtttagg cttaaacgag tccgaacagt agtaaatcct ttgttcggat

309181 aaatgatatt gctacttgac taaaatacta tcatgatgct atataatact acaaggcttt

309241 acggtgaaga aaaacatctt aatgtctgca catatcgacg aagaaattca aagatatgtg

309301 tgaagtcacg caaaatacgc gctaggcgtg gagttgtgga aaatggagcc catcatggct

309361 cgagctacta cgtaagaagt ttttaaatta aataatactc catttaaata tgataaaggc

309421 aaagaaacgc tccaaaagaa tgcctaaaat gttaataggc gttttttaat tcacaactgt

309481 ggaacgataa atgaagatat tttgttattg attacggaat ttcttttatg acacacggtt

309541 aaaaagttac gtgaactaga acactgactt atttaataat ttttttttct ttattaaatt

309601 gcaaattatc caaatgtatt atatttatta gggcatttat agactcctgt agatttttag

309661 ttaagtttaa atacactcat cattggtaat taagggagac atttaataag gaccttagaa

309721 gtcagagtgc agcaagtcta gataaaatat ctttagaaaa aaatttaaac ttcataatta

309781 ttaaatgatt ttaggtctta attgcaatac agtcataatt ctccatacta agttacctcc

309841 ataagcgtaa aactcggatt agagagaaac ctctataatc gagagtcatt ctatgctcac

309901 cgcgagctac ctccataagc gagaagctcg tattagagag aaacctatgt aagcgagaca

309961 aatatcgcgg tgccttggac tgtcgcttag ccaagttcga ctgtatgttg acagttgtca

310021 gtgtacgtat gtcacagtag cgaattccga cctccacgaa actggatcga gtatgctaat

310081 tccgcggcgc tggtgtcgcg taaattacta tgttacgacg ttaatcgctt tattatctgt

310141 gccactctta atgaggcggc catgttgatt ttttgacagt tatatattgt tattaaaagc

310201 gacatttcgt tatgagaacg agttttttct tgataattaa tcatttttat gttaattata

310261 tttttaattg atagcaataa tagagatttg cttaatttat tttattcttt aactattctt

310321 tgataccatt taatgtaata tgaatttttg taagaattag ctaaatattt cttgataaaa

310381 cgtttataaa tatgtcgcaa tcagaagaag aaattagtct taaattcaaa tagcgtgttc

310441 gatgagtatt gacgttaatc agaagtatat cattgagaaa tgagagatcg ttactaaagc

310501 attgtttgta tggtaaatgg gtgatgagcc atgtatctga actaattata atgtgattga

310561 gaggtgcaga gtgtattcct tgattccgac atcacccagt gccgcccacg agtataataa

310621 cccatagtaa ttctgataac gtgtcttgtc tttaaatatt tcttatattg ttatacctta

310681 aattgtatat taattttgta atataaagtt atttgtccac agttttgacc gcacaagacc

310741 atttggtgtc ataaagcatg gcattgcagg gtaaccgcgg ctcgtaggca cgtcgccgtc

310801 tgtagcctca ctaacgtttt atgggcactt tgtggatagc atttacaatt tattgctaca

310861 aatatttatt tatatgtgga cattattata caaattaacg tttaaataaa atattaacgg

310921 aaaataataa tagaaactag taatgtccag gtatcttgaa aaagtgtagc ctattttact

310981 cgatggtgtg tttttctaac ggtgattttt ttttaaatca tcccagttat tgagttatac

311041 aaatctttcc tctttgtaat attagtacat atctacctat aaaataatat tattaaatta

311101 tcaatcttta ttgttattct aaccaaacta cactttatat ctatactaat attatataga

311161 gagaaacttt gtatttttga ttttttgtat ttttgtatgt attgaagtaa cttaagaact

311221 actgggccga tttcaaaaaa tctttcacca ttagaagcta tattatcccc aagtaacata

311281 ggctatatta tttaattcca cgcggacgaa gtcgcgcaca acagctagtt atattataaa

311341 gcggcaaacg agcaagcggc cacctggata cgcttataaa ataaagcgat cgctgcccaa

311401 agaccctgct gctgcctacc ctgaattagg aggagacgta tagcatgagg ataaatcacc

311461 tttccatgcg tccccgcttt cgccaaatcc acttcccctt gccatcattt tcttataaga

311521 aaaggatagg aagggaaaat ggactaaaat tagtcctccg gcaccacact catcagacga

311581 aatacggaat aacgtccatt tgacacctgt cttctgtgta ctcgtggtat ttcaccgggc
[truncated: 104,307 more chars]
